# NF-κB inhibitors, unique γ-pyranol-γ-lactams with sulfide and sulfoxide moieties from Hawaiian plant *Lycopodiella cernua* derived fungus *Paraphaeosphaeria neglecta* FT462

**DOI:** 10.1038/s41598-017-10537-1

**Published:** 2017-09-05

**Authors:** Chun-Shun Li, Ariel M. Sarotti, Peng Huang, Uyen T. Dang, Julian G. Hurdle, Tamara P. Kondratyuk, John M. Pezzuto, James Turkson, Shugeng Cao

**Affiliations:** 1 Department of Pharmaceutical Sciences, Daniel K. Inouye College of Pharmacy, University of Hawai’i at Hilo, 200W. Kawili Street, Hilo, HI 96720 USA; 2 Cancer Biology Program, Cancer Center, University of Hawaii, 701 Ilalo Street, Honolulu, Hawai’i 96813 USA; 3 Instituto de Química Rosario (CONICET), Facultad de Ciencias Bioquímicas y Farmacéuticas, Universidad Nacional de Rosario, Suipacha 531, Rosario, 2000 Argentina; 4 College of Pharmacy, Anhui University of Chinese Medicine, 45 Shihe Road, Hefei, 230031 China; 5 Center for Infectious and Inflammatory Diseases, Texas A&M Health Science Center, 2121 West Holcombe Blvd., Houston, TX 77030 USA; 6 Arnold & Marie Schwartz College of Pharmacy and Health Sciences, Long Island University, 75 DeKalb Avenue, Brooklyn, NY 11201-5497 USA

## Abstract

LC-UV/MS-based metabolomic analysis of the Hawaiian endophytic fungus *Paraphaeosphaeria neglecta* FT462 led to the identification of four unique mercaptolactated γ-pyranol–γ-lactams, paraphaeosphaerides E–H (**1**–**4**) together with one γ-lactone (**5**) and the methyl ester of compound **2** (**11**). The structures of the new compounds (**1**–**5** and **11**) were elucidated through the analysis of HRMS and NMR spectroscopic data. The absolute configuration was determined by chemical reactions with sodium borohydride, hydrogen peroxide, α-methoxy-α-(trifluoromethyl)phenylacetyl chlorides (Mosher reagents), and DP4 + NMR calculations. All the compounds were tested against STAT3, A2780 and A2780cisR cancer cell lines, *E*. *coli* JW2496, and NF-κB. Compounds **1** and **3** strongly inhibited NF-κB with IC_50_ values of 7.1 and 1.5 μM, respectively.

## Introduction

Endophytic fungi are rich in biologically active secondary metabolites^1–13^. Our previous investigation of the Hawaiian endophytic fungus *Paraphaeosphaeria neglecta* FT462 had led to the identification of a few phaeosphaeride A analogs including paraphaeosphaeride A, a unique γ-pyranone–γ-lactam–1,4-thiazine derivative, and we had proposed a biosynthetic pathway for paraphaeosphaeride A^2^. We also proposed an intermediate structure that could be generated from mercaptolactate and phaeosphaeride A, which showed STAT3 inhibition^2, 3^. Motivated by the unusual structures of paraphaeosphaeride A and the assumed intermediates, we reinvestigated the Hawaiian endophytic fungus *Paraphaeosphaeria neglecta* FT462 for mercaptolactated γ-pyranol/pyranone–γ-lactam analogs as presented in the proposed biosynthetic pathways of paraphaeosphaeride A and phaeosphaeride A.

**Figure 1:**
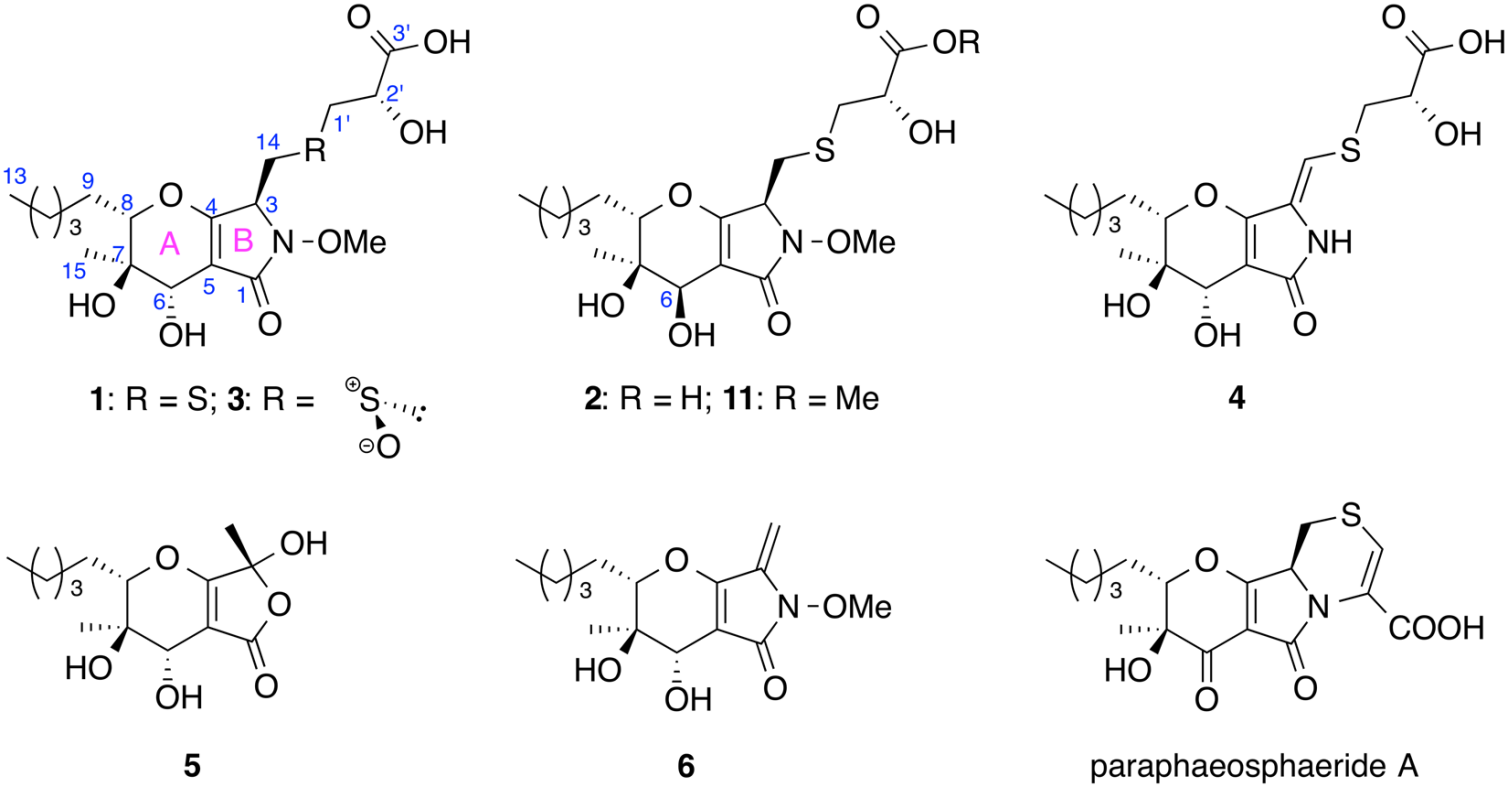
Chemical structures of **1**–**6**, **11** and paraphaeosphaeride A.

The fungus *Paraphaeosphaeria neglecta* FT462 was cultured under static conditions at room temperature for 30 days in a conical flask (1 L) containing 300 mL/flask liquid MDY medium. The fermented whole broth (6 L) of FT462 was filtered through filter paper, and the mycelia were extracted with 80% acetone/water followed by concentration under reduced pressure to afford an aqueous solution. The supernatant solution and the aqueous mycelia extraction was combined and passed through HP-20 eluted with MeOH-H_2_O (10, 30, 50, 70, 90% methanol in H_2_O) to afford five fractions (Fr. A–E). Fractions A–E were analyzed by LC/HRMS. Molecular ion extraction at 420.1–420.2 (positive mode, [C_18_H_29_NO_8_S + H]) identified two peaks (I and II) in fraction B. Besides I and II, one more peak (III) with similar UV to I and II was also detected, and it had the quasi-molecular ion peak at 436.1647 [C_18_H_29_NO_9_S + H], which was 16 units more than I and II. Fraction B was further separated by preparative HPLC and semi-preparative HPLC to yield compounds **1**–**3** together with compounds **4**, **5** (Fig. 1) and the methyl ester of compound **2** (**11**), which might be an artifact produced during extraction and separation. Compounds **1**–**3** corresponded to I-III, respectively. In this report, the separation, structural elucidation, determination of absolute configuration with chemical reaction and NMR calculation, and biological activity are presented.

## Results and Discussion

**Figure 2:**
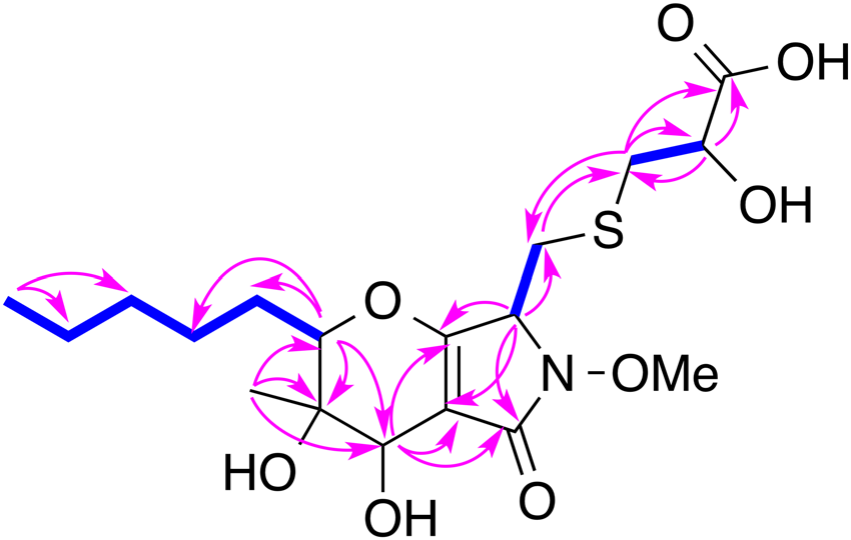
Key ^1^H-^1^H COSY (bolds) and HMBC (arrows) correlations of **1**.

Compound **1** was isolated as a light yellowish solid. Its molecular formula was determined to be C_18_H_29_NO_8_S based on a high resolution ESI-MS [M + H]^+^ of 420.1678 (calcd for C_18_H_30_NO_8_S, 420.1687), with 5 degrees of unsaturation. A detailed analysis of ^1^H and ^13^C NMR spectra (Table 1) demonstrated the presence of one methoxyl, two methyl signals, six methylenes with two connected to sulfur (*δ*_H_ 3.01/3.35 and 2.90/2.98, *δ*_C_ 33.6 and 38.4), four methines including three oxygenated and one nitrogenated (*δ*_H_ 4.42, *δ*_C_ 62.3), and five other carbons with no hydrogen attached, four of which are *sp* ^2^ hybridized, including two carbonyl carbons. The NMR data of **1** were very similar to that of phaeosphaeride A (**6**) except that the olefinic methylene (H_2_-14) in the molecule of **6** was absent in that of **1**. Meanwhile, two more methines with one oxygenated(CH–O) and another nitrogenated (CH–N) and two more methylenes connected to sulfur (2 × CH_2_–S) were present in **1**. One *α*,*β*-unsaturated γ-lactam (*δ*_C_ 175.7) and two rings (A and B) accounted for four degrees of unsaturation. The remaining degree of unsaturation was supported by the existence of one carbonyl group (*δ*_C_ 175.9). Therefore we could argue that **1** was derived from **6** (phaeosphaeride A) and mercaptolactate (HS-CH_2_-CHOH-COOH) as proposed in our previous publication^2^. The HMBC spectrum of **1** demonstrated correlations from H_2_-14 to C-1′ and from H_2_-1′ to C-14, and the COSY-coupled H_2_-1′ and H-2′ showed HMBC correlations to C-3′ (Fig. 2), which justified our argument. Hence, the planar structure of compound **1** was elucidated as shown in Fig. 2, which has the same planar structure as CAS# 1214709-65-5, but no reference was provided for CAS# 1214709-65-5 and its stereogenic centers were not determined.

**Table 1:** ^1^H and^13^C NMR Spectroscopic Data for Compounds **1**–**5** in Methanol-*d*_4_.

#	1		2		3		4		5	
	δ_H_, mult (*J* in Hz)^a^	δ_C_^b^	δ_H_, mult (*J* in Hz)^a^	δ_C_^b^	δ_H_, mult (*J* in Hz)^a^	δ_C_^b^	δ_H_, mult (*J* in Hz)^a^	δ_C_^b^	δ_H_, mult (*J* in Hz)^a^	δ_C_^b^
1		175.7		174.4		—		175.0		174.7
2-OMe	3.86 s	65.0	3.82 s	64.9	3.90 s	64.8				
3	4.42 t (6.0)	62.3	4.48 t (6.0)	61.8	4.61 t (5.5)	58.6		130.2		102.5
4		168.6		168.4		169.3		159.5		174.7
5		106.6		107.6		105.6		105.7		102.0
6	4.02 brs	66.6	4.17 brs	68.2	4.00 brs	66.3	4.13 brs	67.5	4.05 brs	66.5
7		72.8		72.4		72.4		73.0		72.4
8	4.10 br d (12.0)	88.7	4.04 br d (12.0)	88.3	4.15 br d (11.2)	89.1	4.03 br d (12.0)	87.6	4.17 br d (12.0)	88.6
9	1.60 m; 1.93 m	29.5	1.73 m; 1.80 m	29.0	1.64 m; 1.91 m	29.2	1.66 m; 1.86 m	29.1	1.66 m; 1.90 m	28.9
10	1.37 m; 1.60 m	28.1	1.37 m; 1.58 m	27.6	1.40 m; 1.64 m	27.7	1.39 m; 1.57 m	27.7	1.39 m; 1.58 m	27.2
11	1.35 m	32.7	1.33 m	32.7	1.34 m	32.3	1.34 m	32.5	1.34 m	32.1
12	1.36 m	23.7	1.34 m	23.6	1.35 m	23.4	1.34 m	23.4	1.35 m	23.1
13	0.91 t (6.0)	14.4	0.91 t (6.0)	14.4	0.92 t (6.0)	14.2	0.91 t (6.0)	13.9	0.91 t (6.0)	13.9
14	3.01 dd (18.0, 6.0); 3.35 dd (18.0, 6.0)	33.6	3.01 dd (18.0, 6.0); 3.29 dd (18.0, 6.0)	33.1	3.54 m	52.1	6.31 s	110.5	1.64 s	22.9
15	1.31 s	20.0	1.23 s	20.0	1.32 s	19.8	1.28 s	18.8	1.32 s	19.4
1′	2.90 dd (14.0, 6.0); 2.98 dd (14.0, 4.0)	38.4	2.81 dd (18.0, 8.7); 2.98 dd (18.0, 5.0)	38.3	3.08 m; 3.51 m	57.4	3.10 m; 3.27 m	39.8		
2′	4.34 dd (6.0, 4.0)	72.5	4.29 dd (8.7, 5.0)	72.2	4.40, brd (8.1)	66.9	4.29 brs	72.2		
3′		175.9		175.9		—		177.5		

^a^Spectra recorded at 500 MHz (but 400 MHz for **4** and **5**). ^b^Spectra recorded at 125 MHz (but 100 MHz for **4** and **5**). Data based on ^13^C, HSQC, and HMBC experiments.

Compound **2** was also isolated as a light yellowish solid. Its molecular formula was determined to be C_18_H_29_NO_8_S (calcd for C_18_H_30_NO_8_S, 420.1687) based on a high resolution ESI-MS [M + H]^+^ of 420.1677, which was the same as that of **1**. The ^1^H and ^13^C NMR data of **2** were almost identical to those of **1** except position 6. The planar structure of compound **2** was determined to be the same as that of **1** based on its 1D and 2D NMR data. We proposed that compound **2** was derived from the 6-epimer of **6** and mercaptolactate (HS-CH_2_-CHOH-COOH), which meant that **1** and **2** differed in configuration only at C-6.

Compound **3** was isolated as light yellowish solids. Its molecular formula was determined to be C_18_H_29_NO_9_S (calcd for C_18_H_28_NO_9_S, 434.1490) based on a high resolution ESI-MS [M − H]^−^ of 434.1503, which was 16 units more than the molecular formula (C_18_H_29_NO_8_S) of **1** and **2**. The ^1^H and ^13^C NMR data of **3** were almost identical to those of **1** except the side chains at 3-position, which had the same ^1^H-^1^HCOSY and HMBC correlations as those of compounds **1** and **2**. We assumed that the functional group between 14-position and 1′-position was a sulfoxide in **3** instead of a sulfide in **1** and **2**. Hence, the planar structure **3** was determined as shown.

Compound **4** was isolated as a light yellowish solid. Its molecular formula was determined to be C_17_H_25_NO_7_S (calcd for C_17_H_26_NO_7_S, 388.1424) based on a high resolution ESI-MS [M + H]^+^ of 388.1418. The ^1^H NMR of **4** was similar to that of compound **1**, except that the *N*-methoxyl group and the CH-CH_2_ spin system at 3- and 14-positions in the molecule of **1** were absent in that of **4**. Meanwhile, one olefinic proton at *δ*_H_ 6.31 presented in **4**. This olefinic proton was assigned to H-14 readily since it showed HMBC correlations to C-3, C-4, and C-1′. Compound **4** was the *N*-demethoxyl and 3,14-dehydrogenated product of **1**. Hence, the structure of compound **4** was determined as shown.

Compound **5** was isolated as a colorless solid. Its molecular formula was determined to be C_14_H_22_O_6_ (calcd for C_14_H_23_O_6_, 287.1489) based on a high resolution ESI-MS [M + H]^+^ of 287.1476. The ^1^H NMR signals in the 4-dihydropyranol (ring A) and the side chain at 8-position of **5** were almost the same as those of compound **6**, while the *N*-methoxyl group and the olefinic protons at 14-position in ring B of **6** were absent in **5**. Instead, a third methyl group at δ_H_ 1.64 ppm was observed in the ^1^H NMR spectrum of **5**. The signal at *δ*_H_ 1.64 ppm showed HMBC correlations to C-3 (*δ*_C_ 102.5) and C-4 (*δ*_C_ 174.7) indicating C-3 of **5** must be di-oxygenated, and **5** should be a lactone instead of a lactam. Hence, the structure of compound **5** was determined as shown.

**Figure 3:**
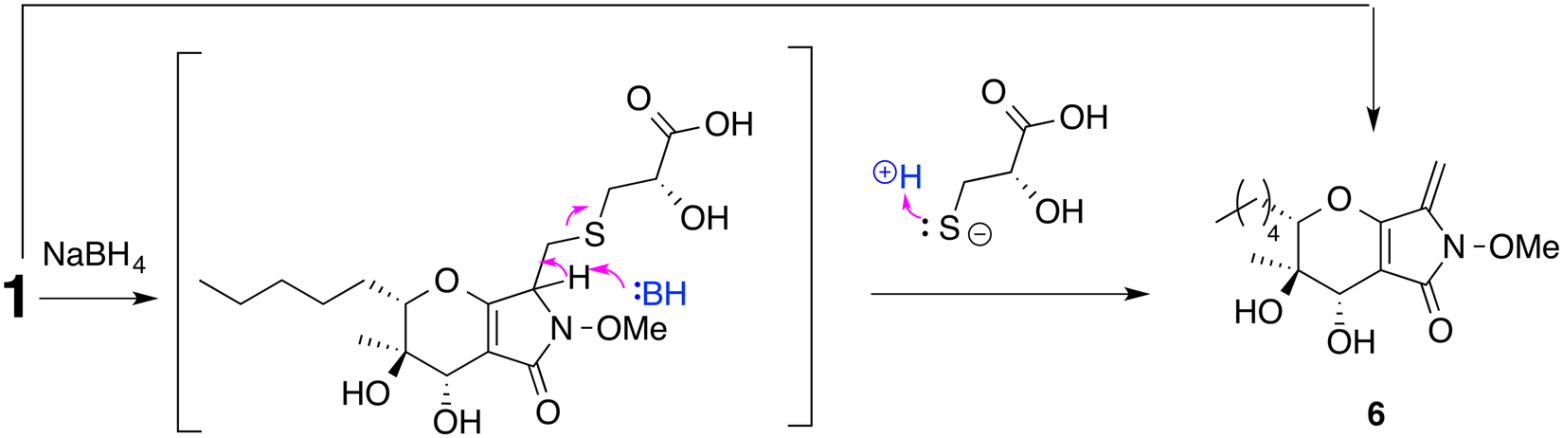
Conversion of **1** to **6** under basic condition.

**Figure 4:**
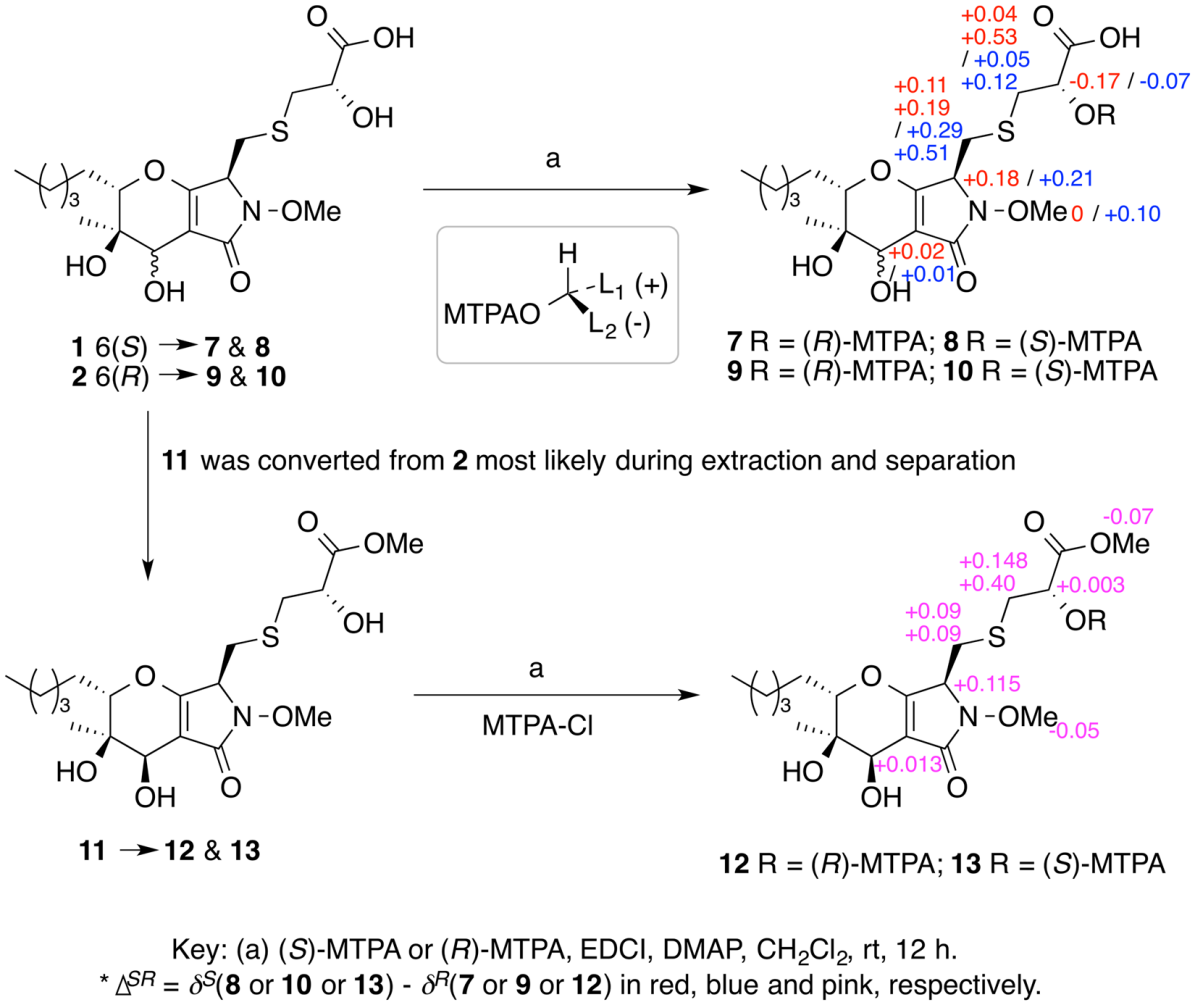
Reactions of **1**, **2** and **11** with Mosher reagents.

To determine the configuration of 3-position of **1**, **1** was treated with sodium borohydride^14^. We expected the double bond between A- and B-rings would be reduced so that we could run a ROESY NMR experiment, but instead the reaction yielded phaeosphaeride A (**6**) (Fig. 3), indicating that the configuration of ring A of **1** was the same as that of **6**. In order to determine the configuration of C-2′, **1** and **2** were reacted with *R*- and *S*-Mosher reagents^15^. Results showed that both **1** and **2** should have an *S* configuration at 2′-position (Fig. 4). To determine the absolute configuration unequivocally by the modified Mosher’s method, the ∆^*SR*^ values on both left and right sides of a secondary alcohol should be calculated and the sign distributions should be consistent. Unfortunately, the ∆^*SR*^ value at the carboxylic acid proton for either compounds **8** and **7**, or **10** and **9** couldn’t be obtained. Fortunately, we isolated the methyl ester of compound **2** (**11**), which was reacted with both *S*- and *R*-Mosher reagents to yield two Mosher esters (**12** and **13**, Fig. 4). Result clearly showed negative ∆^*SR*^ values for both H-2′ and the methoxy at 3′ position, and positive ∆^*SR*^ values for the others (Fig. 4). Hence, the configuration of **1** at 2′-position was determined without any doubt.

In our previous study, we determined the configuration at C-3 of paraphaeosphaeride A as *S* from experimental and calculated Electronic Circular Dichroism (ECD) analysis^2^. Given the close structural similarity of the former natural product with **1** and **2**, we hypothesized that the absolute configuration at C-3 should be also *S* in both newly isolated compounds. In order to confirm our deduction, we next carried out DFT calculations of the NMR shifts using the GIAO method^16, 17^. This approach has been extensively employed recently to settle the structure and stereochemistry of complex natural or synthetic products, and emerges as a suitable alternative to propose the most likely structure of an organic compound in a simple and affordable fashion^16–21^. Among the several strategies that have been developed to support (or reject) a given structural proposal^17, 22–26^, we decided to use the DP4 + probability, the method of choice for assessing the most likely candidate when only one set of experimental data is available (as in this case)^23^. This probability is an updated and improved version of the DP4 method (developed by the Goodman group)^25^ that includes the use of both scaled and unscaled chemical shifts computed at higher levels of theory. Thus, following the DP4 + methodology, we computed the NMR shifts at the PCM/mPW1PW91/6-31 + G**//PCM/B3LYP/6-31G* level of theory using methanol as solvent. Since the NMR data of compounds **1** and **2** were collected in CD_3_OD, here we also include the solvent effect during the geometry optimization stage. Confirming our hypothesis, the calculated values for the two isomers with the *S* configuration at C-3 (**1–3** ***S*** and **2–3** ***S***) (Tables 2 and SI) showed better agreement with the experimental data than those corresponding to the 3*R* counterparts (**1–3** ***R*** and **2–3** ***R***, respectively). For instance, the CMAE (corrected mean average error, defined as Σ_n_|δ_scaled_ − δ_exp_|/n) computed for **1–3** ***S*** and **2–3** ***S*** from ^13^C NMR data were 1.0 ppm and 1.1 ppm, respectively, lower than those calculated for **1–3** ***R*** (1.6 ppm) and **2–3** ***R*** (1.8 ppm). In a similar fashion, the CMAE values calculated from ^1^H NMR data were lower for **1–3** ***S*** (0.10 ppm) and **2–3** ***S*** (0.08 ppm) than those of **1–3** ***R*** (0.11 ppm) and **2–3** ***R*** (0.10 ppm), respectively. The isomers with a 3*R* configuration (**1–3** ***R*** and **2–3** ***R***) also displayed higher outliers (CMaxErr, corrected maximum error, defined as max|δ_scaled_ − δ_exp_|) values (7.3 ppm and 4.4 ppm, respectively, for ^13^C data; 0.50 and 0.24 ppm, respectively, for ^1^H data) in comparison with the corresponding values of **1–3** ***S*** and **2–3** ***S*** (3.3 ppm and 4.0 ppm, respectively, for ^13^C data; 0.24 and 0.21 ppm, respectively, for ^1^H data). The better fit of **1–3** ***S*** and **2–3** ***S*** was further corroborated with DP4 + calculations, where each isomer was identified as the most likely candidates in high probability (>99.9% in both cases).

**Table 2:** Scaled (δ_s_) ^1^H and ^13^C NMR chemical shifts of the more likely structures of **1**–**5** computed at the PCM/mPW1PW91/6–31 + G**//PCM/B3LYP/6–31 G* level of theory (solvent = methanol).

#	1–3*S*		2–3*S*		3-S*S*		4–3*Z*		5–3*R*	
	δ_H_	δ_C_	δ_H_	δ_C_	δ_H_	δ_C_	δ_H_	δ_C_	δ_H_	δ_C_
1		175.8		174.2				169.4		169.6
2-OMe	3.88	64.1	3.80	64.1	3.92	63.9				
3	4.18	64.0	4.27	62.9	4.32	60.7		129.3		104.3
4		169.2		170.1		167.7		159.2		175.5
5		106.5		105.9		105.8		104.1		104.0
6	4.18	67.4	4.37	65.6	4.44	70.7	4.48	73.1	4.27	70.9
7		74.9		72.8		72.8		73.6		73.4
8	4.26	88.7	4.17	86.9	4.14	88.3	3.81	87.1	3.96	89.7
9	2.16; 1.51	28.7	1.70; 1.56	29.4	1.85; 1.80	28.4	1.87; 1.67	27.9	1.87; 1.74	28.2
10	1.66; 1.25	27.2	1.61; 1.36	27.9	1.69; 1.30	28.5	1.49	27.8	1.50	27.9
11	1.23	32.1	1.30	32.8	1.30	32.9	1.31	32.6	1.31	32.8
12	1.34	23.8	1.38	24.4	1.41	24.6	1.37	24.1	1.33	24.3
13	0.92	12.6	1.00	13.2	0.96	14.0	0.98	13.1	0.98	13.3
14	3.18	34.0	3.12	33.7	3.32	56.4	6.24	117.4	1.67	20.6
15	1.44	18.6	1.28	19.1	1.21	14.1	1.15	11.8	1.21	14.3
1′	2.78	41.7	2.95	42.3	3.30	51.8	3.10	42.9		
2′	4.32	71.7	4.25	71.2	4.48	68.4	4.35	71.7		
3′		174.0		176.4				176.9		

**Figure 5:**
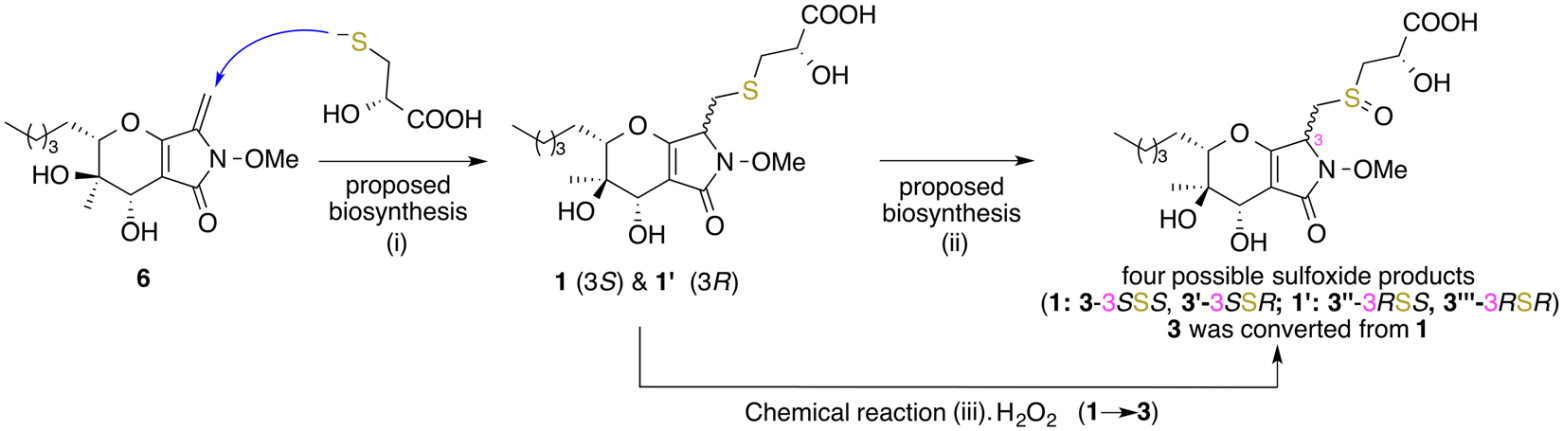
Proposed biosynthesis of two sulfides (**1** and **1**′) and four sulfoxides (**3**, **3**′, **3**″, and **3**′″) (steps i and ii). Chemical conversion of **1** to **3** (step iii) with hydrogen peroxide (H_2_O_2_). **1**′, **3**′, **3**″, and **3**′″ were not isolated.

Reduction of compound **3** with sodium borohydride also yielded compound **6**. As proposed in our previous publication^2^, nucleophilic addition of the mercaptolactate thiol to C-14 of compound **6** could generate **1** and its 3-epimer (**1**′, which was not isolated from *Paraphaeosphaeria neglecta* FT462 in this study). Further oxidation of **1** and **1**′ could yields four sulfoxides including **3** (**3**, **3**′, **3**″, and **3**′″, Fig. 5). From a biosynthetic point of view, **3** should have the same configuration at 2′-position as **1** and **2**. To confirm the above assumption, **1** was treated with hydrogen peroxide and *tert*-butyldimethyl silyl chloride^27^, and compound **3** was detected (Fig. 5). However, the configuration at the sulfoxide in **3** was still not determined. Once again, the two possible isomers of **3** (**3-S** ***R*** and **3-S** ***S***) were submitted to NMR calculation at the PCM/mPW1PW91/6-31 + G**//PCM/B3LYP/6-31 G* level of theory (Tables 2 and SI). In this case, the ^13^C and ^1^H NMR data pointed toward different directions, which is a common situation in the field of quantum calculations of NMR shifts^16–26^. For instance, the CMAE and CMaxErr computed for **3-S** ***R*** using ^13^C NMR data (1.8 ppm and 5.4 ppm, respectively) were slightly lower than those calculated for **3-S** ***S*** (1.9 ppm and 5.7 ppm, respectively), whereas using the experimental ^1^H NMR shifts the trend was reversed (0.14 ppm and 0.56 ppm, respectively, for **3-S** ***R***; 0.11 and 0.44 ppm for **3-S** ***S***). In this case, proton data was the most conclusive one, and isomer **3-S** ***S*** was identified as the correct structure of **3** in high probability (99.5%) after DP4 + calculations.

Two more peaks, each of which has the same molecular weight as that of **3**, were detected in the LC-MS of fraction B. Most likely, they are diastereoisomers of **3** with different configurations at the sulfoxide, 3-, and 6-positions since all the analogs that we isolated have the same configurations at 7- and 8-positions. We tried to isolate these compounds and determined their structures, but failed due to insufficient material and instability of these molecules. Nevertheless, it is worthy to investigate these compounds further.

In the case of compounds **4** and **5**, we concluded that they should have the same configurations at C-6, C-7 and C-8 as those of **1** since minor differences in the ^13^C NMR shifts were noted at those centers. Also, from a biosynthetic point of view, **4** should have the same configuration at 2′-position as **1**–**3**. However, as throughout this study, we were not able to unequivocally determine the configuration at C-3 from experimental NMR basis, and we had to rely on the computational chemistry assistance to settle this issue. Thus, the NMR calculations of the two possible isomers of **4** (**4–3** ***E*** and **4–3** ***Z***) and **5** (**5–3** ***R*** and **5–3** ***S***) were carried out at the PCM/mPW1PW91/6–31 + G**//PCM/B3LYP/6-31G* level of theory (Table 2, Tables S4 and S5). In the case of **4**, the 3*Z* isomer displayed much better agreement with the experimental values than the 3*E* candidate, mainly in the ^13^C NMR region, in which lower CMAE (2.2 ppm *vs* 2.9 ppm) and CMaxErr (7.0 ppm *vs* 14.2 ppm) was noted. On the other hand, the computed ^13^C NMR shifts of **5-3** ***R*** and **5–3** ***S*** showed similar match with the experimental shifts (CMAE 2.0 ppm in both cases), though the prediction of the ^1^H NMR data was improved in the case of **5–3** ***R*** (CMAE = 0.08 ppm *vs* 0.09 ppm; CMaxErr 0.22 ppm *vs* 0.25 ppm). The DP4 + calculations were in line with these findings, suggesting that the most likely configuration at C-3 of **4** and **5** is *Z* (>99.9%) and *R* (92.2%), respectively. ROESY correlations between H-14 and H-6, and H-14 and H-8 further confirmed the configuration of **5**.

All the compounds (**1**–**5**, **11**) were tested against STAT3, A2780 and A2780S, but none was active at the concentration of 20 μg/mL. The compounds were also evaluated for their activity against pathogenic bacteria, *S*. *aureus*, *C*. *difficile*, *P*. *aeruginosa* PAO1, and *E*. *coli* JW2496 (∆*bamB*). Only compound **1** mildly inhibited *E*. *coli* JW2496 at 20 *μ*g/mL.

Since phaeosphaeride A showed STAT3 inhibition^2, 3^, and NF-κB and STAT3 act as two major transcriptional factors linking inflammation with cancer progression, and they functionally interact with each other at many different levels, we tested all compounds for their ability to inhibit NF-κB and iNOS. When evaluated in a mammalian cell-based assay designed to monitor TNF-*α*-induced NF-κB activity, compounds **1** and **3** were found to mediate inhibitory responses with IC_50_ values of 7.1 and 1.5 μM (Table 3), respectively. When tested using the same conditions as the NF-κB assay, none showed toxicity (Table 3). In the absence of a cytotoxic response, inhibition of TNF-*α*-induced NF-κB activity suggests the potential of a cancer chemopreventative response. Compounds **1** and **3** also inhibited iNOS with IC_50_ values of 47.9 and 16.1 *μ*M (Table 3), respectively. According to the structures and NF-κB activity, it could be concluded that the sulfoxide (**3**) was more active than the sulfide derivatives (**1** and **2**), and the configuration at 6-position (6*S*) was also important for the NF-κB inhibition.

**Table 3:** Inhibitory effect of compounds **1** and **3** against TNF-α-induced NF-κB and lipopolysaccharide-stimulated iNOS activity.

Compound	NF-κB inhibition IC_50_ (μM)	NF-κB cytotoxicity IC_50_ (μM)	iNOS inhibition IC_50_ (μM)	iNOS cytotoxicity IC_50_ (μM)
**1**	7.1	>50	47.9 ± 0.03	>50
**2**	—	>50	43.2 ± 0.7	>50
**3**	1.5	>50	16.1 ± 6.6	>50
TPCK^*a*^	4.09 ± 0.12			
l-NMMA^*b*^			18.8 ± 3.4	

^a^TPCK: *N*_α_-tosyl-l-phenylalanine chloromethyl ketone was used as a positive control in NF-κB assay.

^b^l-MNNA: Na-l- monomethyl arginine was used as a positive control in iNOS assay.

*Paraphaeosphaeria* species produce different types of secondary metabolites, including macrolides^28, 29^, xanthenes^30^, isocoumarins^31^, hexaketide^32^, polyketides^33, 34^, and phaeosphaerides^2^ and paraphaeosphaerides^1, 2, 8, 35^. Paraphaeosphaeride A^2^ is the first sulfur-containing molecule (a sulfide) isolated from *Paraphaeosphaeria* species. In this study, we isolated four more sulfides (**1**, **2**, **4**, and **11**) and a sulfoxide (**3**) together with a γ-lactone (**5**). The configuration of these compounds was determined by NMR, DP4 + calculations and chemical reactions. Thio- or sulfide or even di-sulfide fungal metabolites are not rare, but sulfoxides isolated from fungi were uncommon, for examples, oryzamides D and E from the sponge-derived fungus *Nigrospora oryzae* PF18^36^, and cyclosulfoxicurvularin from the marine-derived *Penicillium* sp. DRF2^37^. The identification of paraphaeosphaerides E-H (**1**–**4**) confirmed our proposed biosynthesis of paraphaeosphaeride A^2^. So far, only a few phaeosphaeride derivatives have been reported, and phaeosphaeride A showed STAT3 inhibitory activity^2, 3^. All compounds were evaluated for their anti-proliferative, antibacterial, NF-κB and iNOS inhibitory activities. Since compounds **1** and **3** inhibited NF-κB with some specificity, they remain of interest as cancer chemopreventative agents.

## Methods

### General Experimental Procedures

Optical rotations were measured with a Rudolph Research Analytical Autopal IV Automatic polarimeter. UV and IR spectra were obtained with Shimadzu UV-1800 spectrophotometer and Thermo scientific Nicolet iS50FT-IR spectrometer, respectively. NMR spectra were recorded in methanol-*d*_4_ on Varian Unity Inova 500 MHz and Bruker 400 MHz. High resolution mass spectra were obtained an Agilent Q-TOF Ultima ESI-TOF mass spectrometer. HPLC was carried out on Agilent 1100 LC system using a Phenomenex Luna C_18_ column (100 mm × 21.2 mm, 5 *μ*m particle size), a Phenomenex Luna phenyl-hexyl column (250 mm × 10 mm, 5 *μ*m particle size) and a Phenomenex Luna C_18_ HPLC column (250 mm × 10 mm, 5 *μ*m particle size). Column chromatography used Diaion HP-20 (Sigma).

### Isolation and Identification of Fungal Strain

The fungal strain^2^ was isolated on PDA medium from a healthy leaf of Hawaiian indigenous plant, *Lycopodiella cernua* (L.) Pic. Serm, which was collected in the Mokuleia Forest Reserve in 2014. The strain was identified as *Paraphaeosphaeria neglecta* (similarity 99.8%) based on comparison result of the DNA sequence of the nuclear ribosomal internal transcribed spacer (ITS) with Genbank. A voucher specimen was deposited at University of Hawaii Cancer Center, USA (accession no. UHCCFT-462).

### Fermentation, Extraction and Isolation

The fungal strain FT462 was cultured under the same condition as that of our previous study^1^. The fermented whole broth (6 L) was filtered through filter paper to separate the supernatant from the mycelia. The later was extracted by 80% acetone/H_2_O 3 times, and the solution was concentrated under reduced pressure to afford an aqueous solution. The combination liquid of the supernatant solution and the mycelia extraction was passed through HP-20 eluted with MeOH-H_2_O (10, 30, 50, 70, 90%) to afford five fractions (Fr. A-E). Fr. B (1.78 g) was further fractionated by preparative HPLC (C_18_ column, 5 μm; 100.0 × 21.2 mm; 15 mL/min; with 0.1% formic acid in mobile phases) eluted with 20–100% MeOH-H_2_O in 30 min to get 30 sub-fractions (SF.1–30). SF.15 was pure enough for NMR analysis, which was compound **1** (22.12 mg). SF.16 (183.02 mg) was further subjected to semi-preparative HPLC (phenyl-hexyl column, 5 μm; 250.0 mm × 10.0 mm; 3 mL/min; with 0.1% formic acid in 25% CH_3_CN/H_2_O) to obtain compounds **2** (25.20 mg, *t*_R_ 28.7 min) and **3** (2.72 mg, *t*_R_ 31.3 min). SF.22 (37.08 mg) was separated by semi-preparative HPLC (C_18_ column, 5 μm; 250.0 mm × 10.0 mm; 3 mL/min), which was eluted from 40% to 55% of CH_3_OH/H_2_O for 15 min, followed by 55% CH_3_OH/H_2_O for 30 min, to obtain compounds **4** (1.25 mg, *t*_R_ 26.9 min) and **5** (1.32 mg, *t*_R_ 28.0 min). SF.26 (57.16 mg) was separated by semi-preparative HPLC (C_18_ column, 5 *μ*m; 250.0 mm × 10.0 mm; 3 mL/min; with 0.1% formic acid in 65% CH_3_OH/H_2_O) to yield compound **11** (8.7 mg, *t*_R_ 9.2 min).

#### Paraphaeosphaeride E (**1**)

yellowish solid; $[{\rm{\alpha }}{]}_{{\rm{D}}}^{25}$–32.6 (*c* *=* 1.4, MeOH). UV (MeOH) λ_max_ 216 nm; IR (film) ν_max_ 3390, 2956, 2927, 2859, 1698, 1655, 1439, 1378, 1266, 1259, 1229, 1091, 1019, 922 cm^−1^; ^1^H (methanol-*d*_4_, 500 MHz) and ^13^C NMR (methanol-*d*_4_, 125 MHz) data, see Table 1; HRMS (ESI-TOF) *m/z*: [M + H]^+^ Calcd for C_18_H_30_NO_8_S 420.1687, Found 420.1678; *m/z*: [M + Na]^+^ Calcd for C_18_H_29_NO_8_SNa 442.1506, Found 442.1493.

#### Paraphaeosphaeride F (**2**)

yellowish solid; $[{\rm{\alpha }}{]}_{{\rm{D}}}^{25}$–66.7 (*c* = 2.2, MeOH). UV (MeOH) λ_max_ 216 nm; IR (film) ν_max_ 3397, 2955, 2930, 2860, 1699, 1655, 1436, 1378, 1288, 1260, 1224, 1093, 1046, 1019, 924 cm^−1^; ^1^H (methanol-*d*_4_, 500 MHz) and ^13^C NMR (methanol-*d*_4_, 125 MHz) data, see Table 1; HRMS (ESI-TOF) *m/z*: [M + H]^+^ Calcd for C_18_H_30_NO_8_S 420.1687, Found 420.1677; *m/z*: [M + Na]^+^ Calcd for C_18_H_29_NO_8_SNa 442.1506, Found 442.1503.

#### Paraphaeosphaeride G (**3**)

yellowish solid; $[{\rm{\alpha }}{]}_{{\rm{D}}}^{25}$–27.8 (*c* *=* 0.36, MeOH). UV (MeOH) λ_max_ 217 nm; IR (film) ν_max_ 3362, 2956, 2930, 2859, 2819, 1708, 1632, 1579, 1440, 1379, 1367, 1130, 1087, 1023, 944 cm^−1^; ^1^H (methanol-*d*_4_, 400 MHz) and ^13^C NMR (methanol-*d*_4_, 100 MHz) data, see Table 1; HRMS (ESI-TOF) *m/z*: [M − H]^−^ Calcd for C_18_H_28_NO_9_S 434.1490, Found 434.1503.

#### Paraphaeosphaeride H (**4**)

yellowish solid; $[{\rm{\alpha }}{]}_{{\rm{D}}}^{25}$–31.6 (*c* = 0.22, MeOH). UV (MeOH) λ_max_ 202, 327 nm; IR (film) ν_max_ 3332, 2966, 2927, 2857, 2360, 2343, 1673, 1634, 1603, 1409, 1379, 1262, 1165, 1077, 907 cm^−1^; ^1^H (methanol-*d*_4_, 400 MHz) and ^13^C NMR (methanol-*d*_4_, 100 MHz) data, see Table 1; HRMS (ESI-TOF) *m/z*: [M + H]^+^ Calcd for C_17_H_26_NO_7_S 388.1424, Found 388.1418.

#### Paraphaeosphaeride I (**5**)

colorless solid; $[{\rm{\alpha }}{]}_{{\rm{D}}}^{25}$–29.4 (*c* = 0.17, MeOH). UV (MeOH) λ_max_ 232 nm; IR (film) ν_max_ 3362, 2957, 2931, 2861, 1744, 1681, 1600, 1422, 1378, 1305, 1176, 1126, 1068, 1035 cm^−1^; ^1^H (methanol-*d*_4_, 400 MHz) and ^13^C NMR (methanol-*d*_4_, 100 MHz) data, see Table 1; HRMS (ESI-TOF) *m/z*: [M + H]^+^ Calcd for C_14_H_23_O_6_ 287.1489, Found 287.1476; *m/z*: [M + Na]^+^ Calcd for C_14_H_22_O_6_Na 309.1309, Found 309.1290.

#### Methyl ester of paraphaeosphaeride F (**11**)

yellowish solid; $[{\rm{\alpha }}{]}_{{\rm{D}}}^{25}$–54.3 (*c* = 0.28, MeOH). UV (MeOH) λ_max_ 218 nm; IR (film) ν_max_ 3392, 2956, 2931, 2860, 2359, 2342, 1738, 1702, 1658, 1438, 1378, 1287, 1260, 1221, 1179, 1091, 1049, 1019, 924 cm^−1^; ^1^H (methanol-*d*_4_, 400 MHz) 4.52 (t, H-3), 4.37 (dd, H-2′), 4.20 (brs, H-6), 4.06 (d, H-8), 3.86 (s, 2-OMe), 3.77 (s, 3′-OMe), 3.29 (dd, H-14), 3.02 (dd, H-14), 2.98 (dd, H-1′), 2.85 (dd, H-1′), 1.84 (m, H-9), 1.78 (m, H-9), 1.63 (m, H-10), 1.43 (m, H-10), 1.40 (m, H-12), 1.39 (m, H-11), 1.27 (s, H-15), 0.91 (t, H-13); ^13^C NMR (methanol-*d*_4_, 100 MHz) 175.5 (C-1), 169.2 (C-4), 108.4 (C-5), 89.2 (C-8), 73.3 (C-7), 69.9 (C-6), 65.7 (2-OMe), 62.6 (C-3), 53.5 (3′-OMe), 38.9 (C-1′), 33.9 (C-14), 33.5 (C-11), 29.9 (C-9), 28.4 (C-10), 24.5 (C-12), 18.9 (C-15), 15.2 (C-13); HRMS (ESI-TOF) *m/z*: [M + H]^+^ Calcd for C_19_H_31_NO_8_S 434.1765, Found 433.1773;

### Reduction, Mosher reaction and oxidation

#### Reduction with Sodium Borohydride (NaBH_4_)

A stirred solution of **1** (5 mg, 11.93 µmol) in THF (850 µL) was added acetic acid (11.93 µmol, dissolved in THF), which was followed by NaBH_4_ (13.12 µmol) at 0 °C. The mixed solution was stirred for a further 1 h, and then for 8 h at room temperature. Then, the solvent was removed in vacuum, and the residue was partitioned between EtOAc and saturated NaHCO_3_ solution. The EtOAc part was purified by semi-preparative HPLC to yield compound **6**.

#### Reaction with Mosher reagents

Acylation of compound **1** with *S*-(+) and *R*-(−)-*α*-methoxy-*α*-(trifluoromethyl)phenyl acetyl chloride (MTPA-Cl) yielded 2′-MTPA esters **7** and **8**, respectively (Fig. 4). The same reaction was also performed with compounds **2** and **11** to obtain their 2′-MTPA esters **9** and **10** (Fig. 4), and **12** and **13**, respectively. The ^1^H NMR signals of the MTPA esters were assigned on the basis of their COSY spectra, and the δ_H_(*S*–*R*) values were then calculated (Fig. 4).

#### Oxidation with hydrogen peroxide (H_2_O_2_)

A solution of compound **1** (4.18 mg, 10 *µ*mol) in acetonitrile (500 µL) was added 30% H_2_O_2_ (2 *µ*L, 20 *µ*mol) and *tert*-butyldimethylsilyl chloride (1.51 mg, 10 *µ*mol) at room temperature. The mixture was stirred for 20 min before adding 500 µL H_2_O to quench the reaction. The mixed solution was extracted by EtOAc and solvent was removed in vacuum.

### Anti-proliferative, antibacterial, NF-kB and iNOS assays

#### Anti-proliferative Assays

Viability of normal mouse fibroblasts (NIH3T3) and two Stat3-activated cancer cell lines, MDA-MB-231 (breast cancer) and U251 MG (glioblastoma) cells was determined using the CyQuant assay according to the manufacturer’s instructions (Life Technologies, CA, USA)^38, 39^. Briefly, cells were cultured in 96-well plates at 1000 cells per well for 24 h and subsequently treated with compounds (20 μg/mL) for 72 h and analyzed. Relative viability of the treated cells was normalized to the DMSO-treated control cells^38, 39^.

#### Antibacterial assay

MIC values were determined against *E*. *coli* JW2496 (∆*bamB*) and other bacteria using the standard microbroth dilution method exactly as previously described^40^, which is based on the methods by the Clinical and Laboratory Standards Institute^41, 42^. The maximum test concentration used was 20 *μ*g/mL.

#### NF-κB assay

We employed human embryonic kidney cells 293, Panomic for monitoring changes occurring along the NF-κB pathway^43^. Stable constructed cells were seeded into 96-well plates at 20 × 10^3^ cells per well. Cells were maintained in Dulbecco’s modified Eagle’s medium (DMEM) (Invitrogen Co.), supplemented with 10% FBS, 100 units/mL penicillin, 100 *μ*g/mL streptomycin, and 2 mM L-glutamine. After 48 h incubation, the medium was replaced and the cells were treated with various concentrations of test substances. TNF-*α* (human, recombinant, *E. coli*, Calbiochem) was used as an activator at a concentration of 2 ng/mL (0.14 nM). The plate was incubated for 6 h. Spent medium was discarded, and the cells were washed once with PBS. Cells were lysed using 50 *μ*L (for 96-well plate) of reporter lysis buffer from Promega by incubating for 5 min on a shaker, and stored at −80 °C. The luciferase assay was performed using the Luc assay system from Promega. The gene product, luciferase enzyme, reacts with luciferase substrate, emitting light, which was detected using a luminometer (LUMIstar Galaxy BMG). Data for NF-κB inhibition are expressed as IC_50_ values (i.e., concentration required to inhibit TNFinduced NF-kB activity by 50%). The known NF-κB inhibitor TPCK was used as a positive control.

#### Nitric Oxide Assay

RAW 264.7 cells (1 × 10^4^ cells/well) were seeded and incubated in 96-well culture plates at 37 °C, 5% CO_2_ in humidified air for 24 h. Then, complete medium was replaced with phenol red-free medium containing various concentrations of the test compounds, followed by LPS stimulation (1 *μ*g/mL) for 20 h. The nitrite released in the culture media was reacted with Griess reagent, and the absorbance was measured at 540 nm. The amount of nitrite was calculated using a standard curve of known nitrite concentration versus absorbance at 540 nm. TPCK was used as a positive control. IC_50_ values were calculated using Table Curve 2D Windows V4.07 by AISN Software Inc^44^.

### Computational details

All the quantum mechanical calculations were performed using Gaussian 09^45^. The conformational search was done using the MMFFaq force field (implemented in Spartan 08)^46^. Given the high conformational flexibility of this system, we used a cutoff energy of 5 kcal/mol, yielding an average of ~500, ~40, and ~400 different conformations per isomer in the cases of **1**–**3**, **4** and **5**, respectively. Next, each conformation was fully optimized at the PCM/B3LYP/6–31 G* level of theory in methanol as solvent using Gaussian 09, followed by frequency calculations at the same level to determine the nature of the stationary points and to compute the thermochemical properties (at 1.0 atm and 298.15 K). The most stable conformers (up to 1.5 kcal/mol from the global minima) were next subjected to NMR calculations. The magnetic shielding constants (σ) were computed using the gauge including atomic orbitals (GIAO) method^47–50^, the method of choice to solve the gauge origin problem^16, 17^, at the PCM/mPW1PW91/6-31 + G** level of theory. The calculations in solution were carried out using the polarizable continuum model, PCM^[51]^, with methanol as the solvent). The unscaled chemical shifts (δ_u_) were computed using TMS as reference standard according to δ_u_ = σ_0_ − σ_x_, where σ_x_ is the Boltzmann averaged shielding tensor (over all significantly populated conformations) and σ_0_ is the shielding tensor of TMS computed at the same level of theory employed for σ_x_. The Boltzmann averaging was done according to the eq. 1:

1 $${\sigma }^{x}=\,\frac{{\sum }_{i}{\sigma }_{i}^{x}{e}^{(-\frac{{E}_{i}}{RT})}}{{\sum }_{i}{e}^{(-\frac{{E}_{i}}{RT})}}$$

where *σ*_*i*_^*x*^ is the shielding constant for nucleus *x* in conformer *i*, *R* is the molar gas constant (8.3145 J K^−1^ mol^−1^), *T* is the temperature (298 K), and *Ei* is the Gibbs free energy of conformer *i* (relative to the lowest energy conformer), obtained from the PCM/B3LYP/6-31 G* frequency calculations. The scaled chemical shifts (δ_s_) were computed as *δ*_*s*_ = (*δ*_*u*_ *− b*)*/m*, where m and b are the slope and intercept, respectively, resulting from a linear regression calculation on a plot of *δ*_*u*_ against *δ*_*exp*_. The DP4 calculations were carried out using the Applet from the Goodman group (at www-jmg.ch.cam.ac.uk/tools/nmr/DP4/). The DP4 + calculations were carried out using the Excel spreadsheet available for free at sarotti-nmr.weebly.com, or as part of the Supporting Information of the original paper^23^.

## Electronic supplementary material

Supplementary Information
