# Supplementary material for: NF-κB inhibitors, unique γ-pyranol-γ-lactams with sulfide and sulfoxide moieties from Hawaiian plant Lycopodiella cernua derived fungus Paraphaeosphaeria neglecta FT462
Source: Sci Rep. 2017 Sep 5;7:10424. doi: 10.1038/s41598-017-10537-1 (PMC5585240; doi:10.1038/s41598-017-10537-1)
Supplement: Supplementary file 1 — Supplementary Information [file 41598_2017_10537_MOESM1_ESM.pdf]

Supporting Information

NF-κB inhibitors, unique γ-pyranol-γ-lactams with sulfide and sulfoxide moieties from Hawaiian plant *Lycopodiella cernua* derived fungus *Paraphaeosphaeria neglecta* FT462

Chun-Shun Li[1,2], Ariel M. Sarotti[3], Peng Huang[1,4], Uyen T. Dang[5], Julian Hurdle[5], Tamara P. Kondratyuk[1], John M. Pezzuto[1,6], James Turkson[2], & Shugeng Cao[*,1,2]

[1]Department of Pharmaceutical Sciences, Daniel K. Inouye College of Pharmacy, University of Hawai'i at Hilo, 200 W. Kawili Street, Hilo, HI 96720, USA.

[2]Cancer Biology Program, Cancer Center, University of Hawaii, 701 Ilalo Street, Honolulu, Hawai'i 96813, USA.

[3]Instituto de Química Rosario (CONICET), Facultad de Ciencias Bioquímicas y Farmacéuticas, Universidad Nacional de Rosario, Suipacha 531, Rosario 2000, Argentina.

[4]College of Pharmacy, Anhui University of Chinese Medicine, 45 Shihe Road, Hefei 230031, China.

[5]Center for Infectious and Inflammatory Diseases, Texas A&M Health Science Center, 2121 West Holcombe Blvd., Houston, TX 77030, USA.

[6] Schwartz College of Pharmacy and Health Sciences, Long Island University, 75 DeKalb Avenue, Brooklyn, NY 11201-5497. Correspondence and requests for materials should be addressed to S. C. (email: scao@hawaii.edu)

Content

**Figure S1**	^{1}H NMR spectrum (500 MHz, methanol-d_4) of compound **1**....................................	**S4**
**Figure S2**	^{13}C NMR spectrum of compound **1**..	**S5**
**Figure S3**	^{1}H-^{1}H COSY of compound **1**..	**S6**
**Figure S4**	HSQC spectrum of compound **1.**..	**S7**
**Figure S5**	HMBC spectrum of compound **1**..	**S8**
**Figure S6**	^{1}H NMR spectrum (500 MHz, methanol-d_4) of compound **2**....................................	**S9**
**Figure S7**	^{13}C NMR spectrum of compound **2**..	**S10**
**Figure S8**	^{1}H-^{1}H COSY of compound **2**..	**S11**
**Figure S9**	HSQC spectrum of compound **2**...	**S12**
**Figure S10**	HMBC spectrum of compound **2**..	**S13**
**Figure S11**	^{1}H NMR spectrum (400 MHz, methanol-d_4) of compound **3**....................................	**S14**
**Figure S12**	^{1}H-^{1}H COSY of compound **3**..	**S15**
**Figure S13**	HSQC spectrum of compound **3**...	**S16**
**Figure S14**	HMBC spectrum of compound **3**..	**S17**
**Figure S15**	^{1}H NMR spectrum (400 MHz, methanol-d_4) of compound **4**....................................	**S18**
**Figure S16**	^{13}C NMR spectrum of compound **4**...	**S19**
**Figure S17**	^{1}H-^{1}H COSY of compound **4**..	**S20**
**Figure S18**	HSQC spectrum of compound **4**...	**S21**
**Figure S19**	HMBC spectrum of compound **4**..	**S22**
**Figure S20**	^{1}H NMR spectrum (400 MHz, methanol-d_4) of compound **5**....................................	**S23**
**Figure S21**	^{13}C NMR spectrum of compound **5**...	**S24**
**Figure S22**	HSQC spectrum of compound **5**..	**S25**
**Figure S23**	HMBC spectrum of compound **5**...	**S26**
**Figure S24**	^{1}H NMR spectrum of compound **7** (*R*-MTPA of **1**) (400 MHz, methanol-d_4)...........	**S27**
**Figure S25**	^{1}H NMR spectrum of compound **8** (*S*-MTPA of **1**) (400 MHz, methanol-d_4)...........	**S28**
**Figure S26**	^{1}H NMR spectrum of compound **9** (*R*-MTPA of **2**) (400 MHz, methanol-d_4)...........	**S29**
**Figure S27**	^{1}H-^{1}H COSY of compound **9** (*R*-MTPA of **2**)	**S30**
**Figure S28**	^{1}H NMR spectrum of compound **10** (*S*-MTPA of **2**) (400 MHz, methanol-d_4)..........	**S31**
**Figure S29**	^{1}H-^{1}H COSY of compound **10** (*R*-MTPA of **2**)	**S32**
**Figure S30**	^{1}H NMR spectrum of compound **11** (400 MHz, methanol-d_4)	**S33**
**Figure S31**	^{13}C NMR spectrum of compound **11**..	**S34**
**Figure S32**	^{1}H-^{1}H COSY of compound **11**..	**S35**
**Figure S33**	HSQC spectrum of compound **11**...	**S36**
**Figure S34**	HMBC spectrum of compound **11**..	**S37**
**Figure S35**	^{1}H NMR spectrum of compound **12** (*S*-MTPA of **11**) (400 MHz, methanol-d_4).......	**S38**
**Figure S36**	^{1}H NMR spectrum of compound **13** (*R*-MTPA of **11**) (400 MHz, methanol-d_4).......	**S39**
**Figures S37-42**	HRESIMS spectra of compounds **1-5**, and **11**..	**S40-S45**
**Figures S43-48**	IR spectra of compounds **1-5**, and **11**..	**S46-S51**
**Table S1**	NMR Boltzmann averaged isotropic magnetic shielding values (σ), unscaled (δ_u) and scaled (δ_c) chemical shifts of **1-3***R* and **1-3***S*	**S52**
**Table S2**	NMR Boltzmann averaged isotropic magnetic shielding values (σ), unscaled (δ_u) and scaled (δ_c) chemical shifts of **2-3***R* and **2-3***S*	**S53**
**Table S3**	NMR Boltzmann averaged isotropic magnetic shielding values (σ), unscaled	**S54**

	(δ_u) and scaled (δ_c) chemical shifts of **3-3***R* and **3-3***S*	
**Table S4**	NMR Boltzmann averaged isotropic magnetic shielding values (σ), unscaled (δ_u) and scaled (δ_c) chemical shifts of **4-3***E* and **4-3***Z*	**S55**
**Table S5**	NMR Boltzmann averaged isotropic magnetic shielding values (σ), unscaled (δ_u) and scaled (δ_c) chemical shifts of **5-3***R* and **5-3***S*	**S56**
**Cartesian coordinates and energies**	Cartesian Coordinates of all significantly populated conformers found for all compounds under study at the PCM/B3LYP/6-31G* level of theory	**S57**

^{1}H NMR spectrum of compound **1** (500 MHz in methanol-d_4)

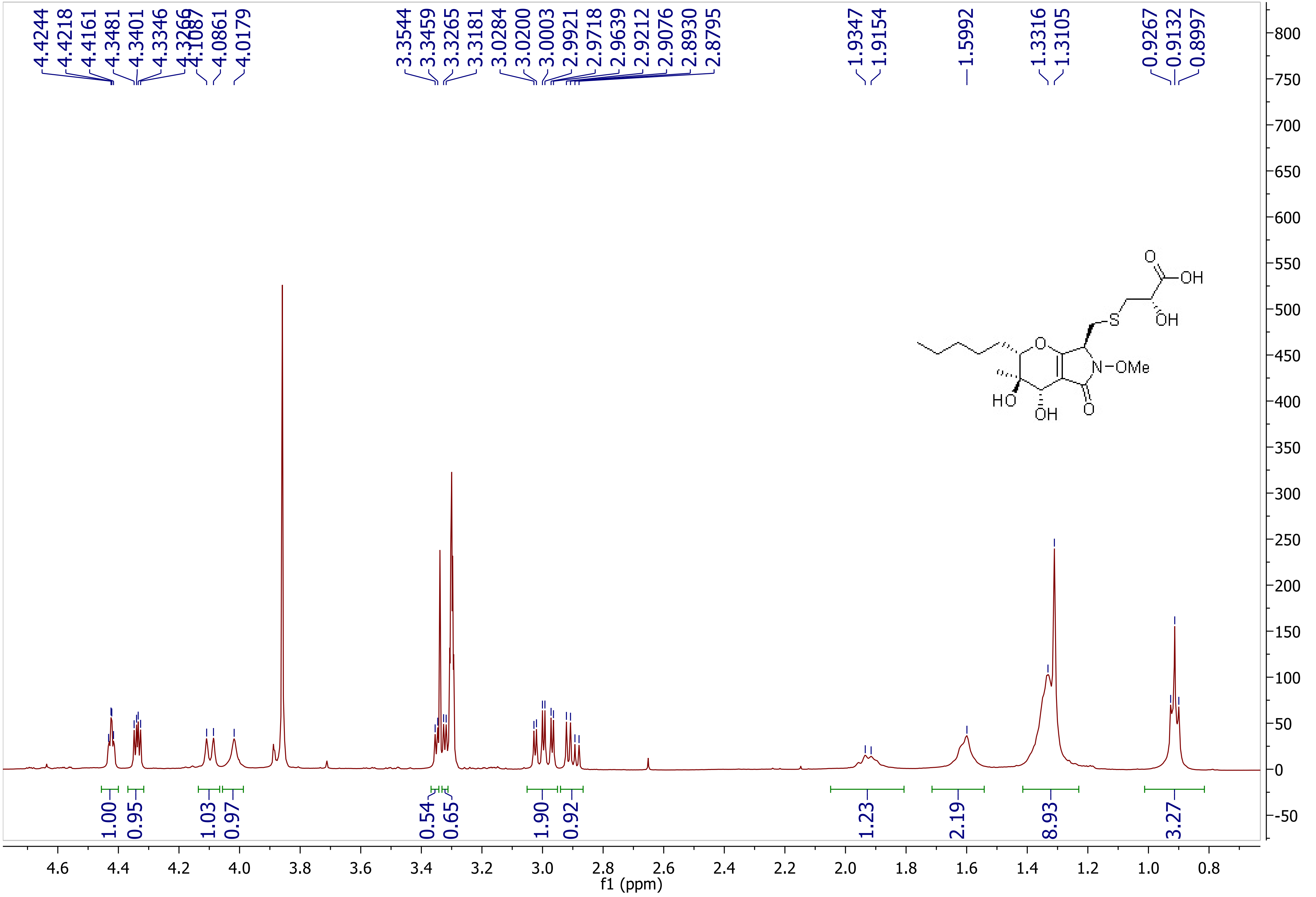

^{13}C NMR spectrum of compound **1**

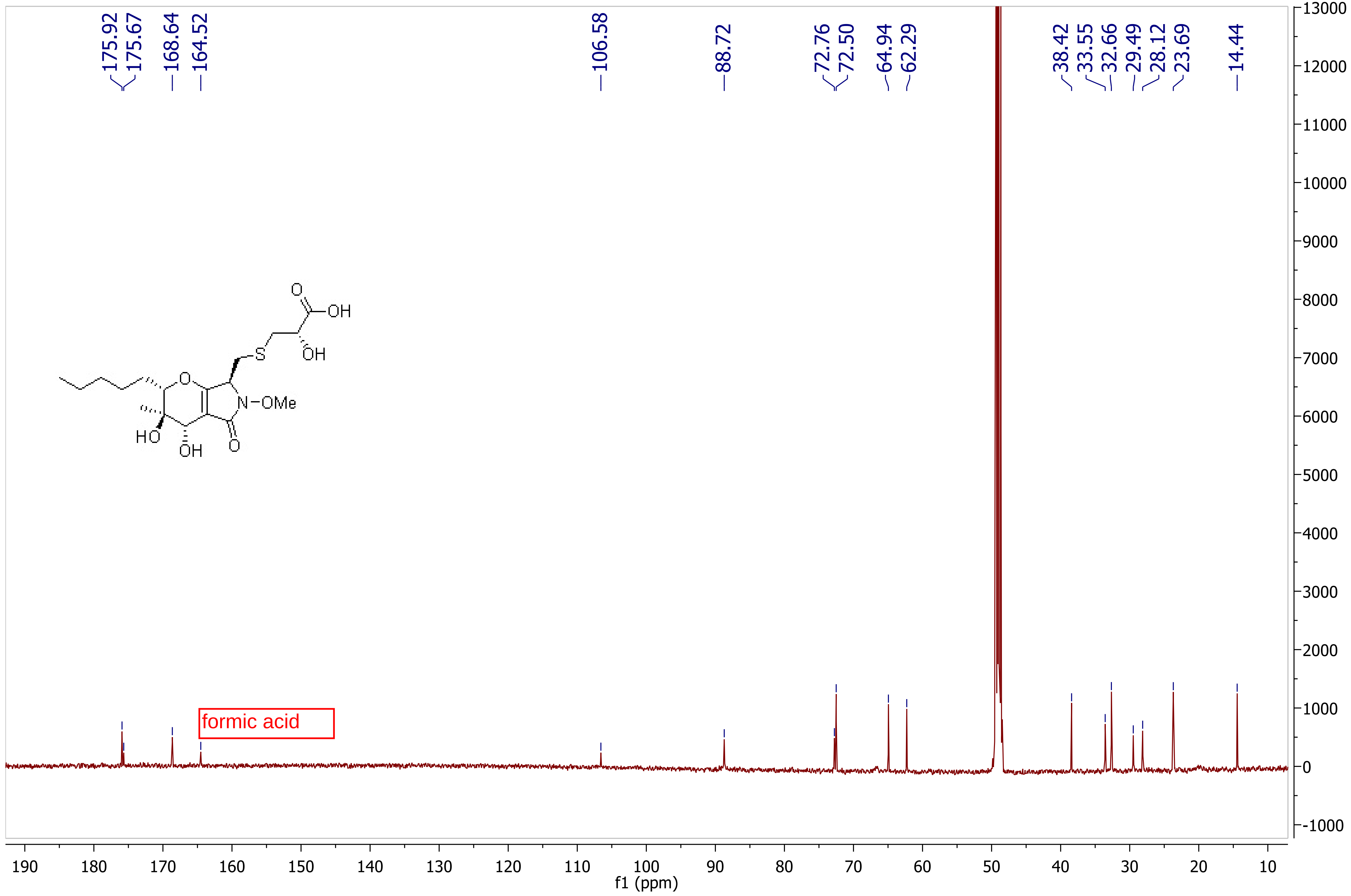

^1H-^1H COSY of compound **1**

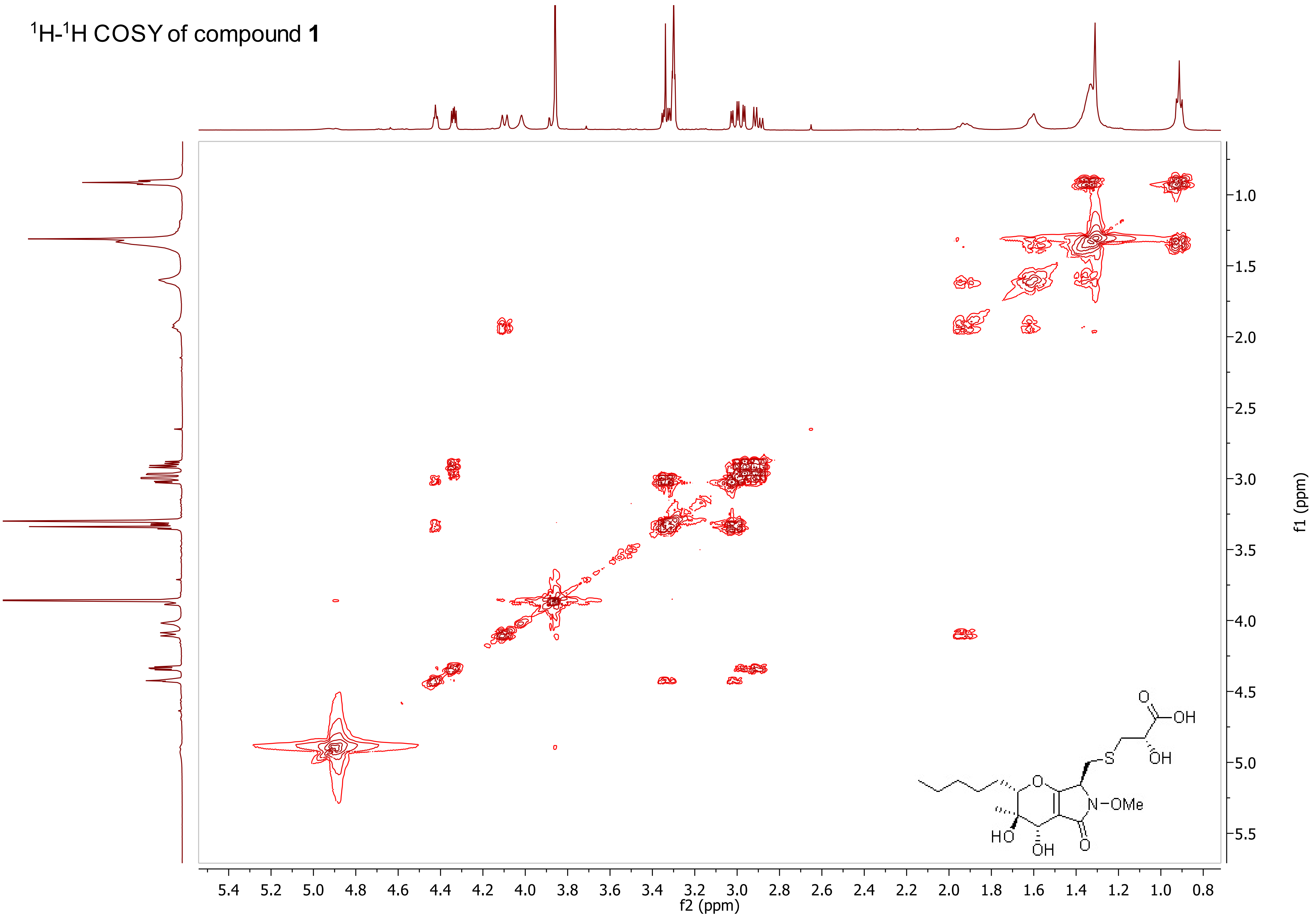

HSQC spectrum of compound **1**

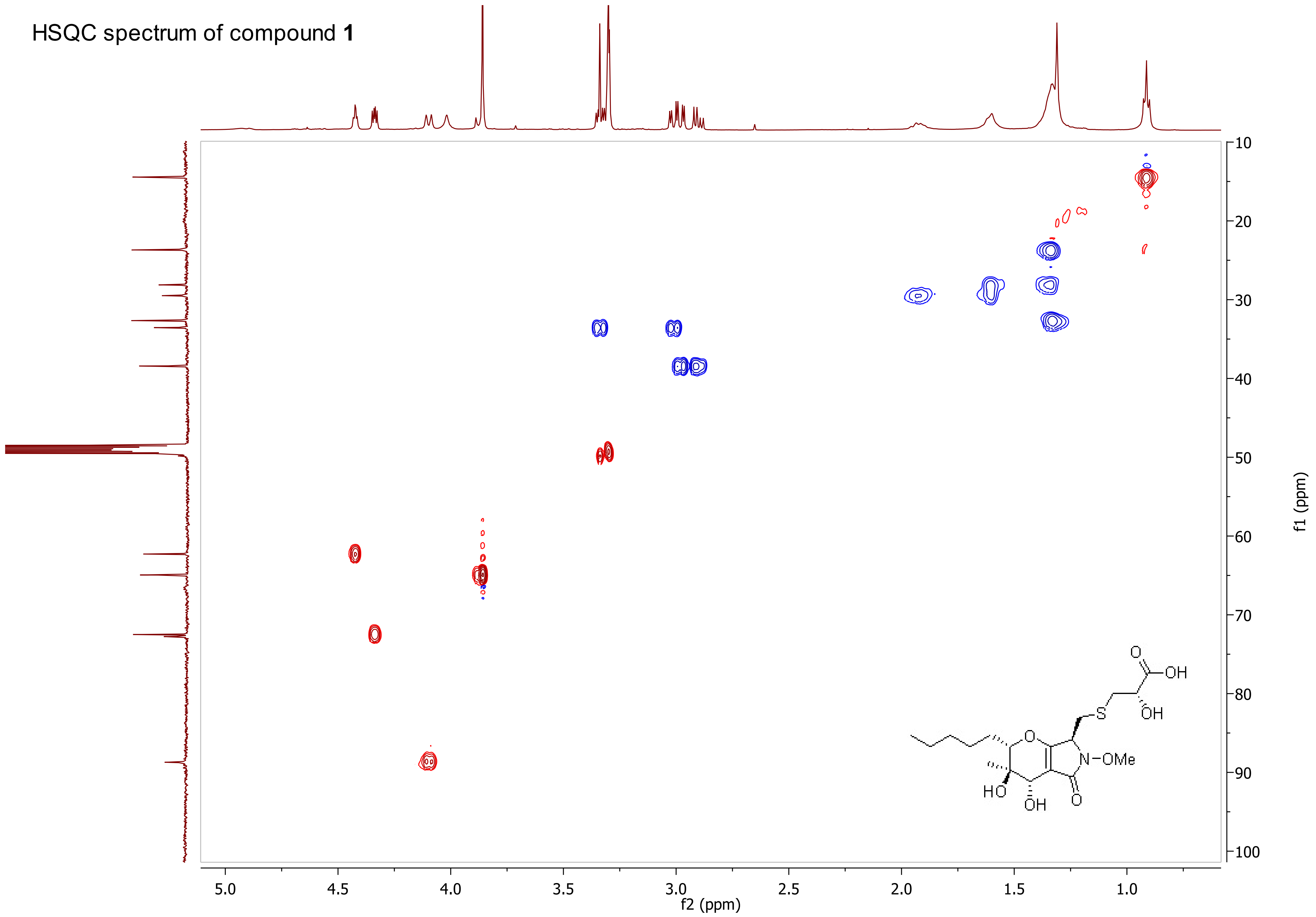

HMBC spectrum of compound **1**

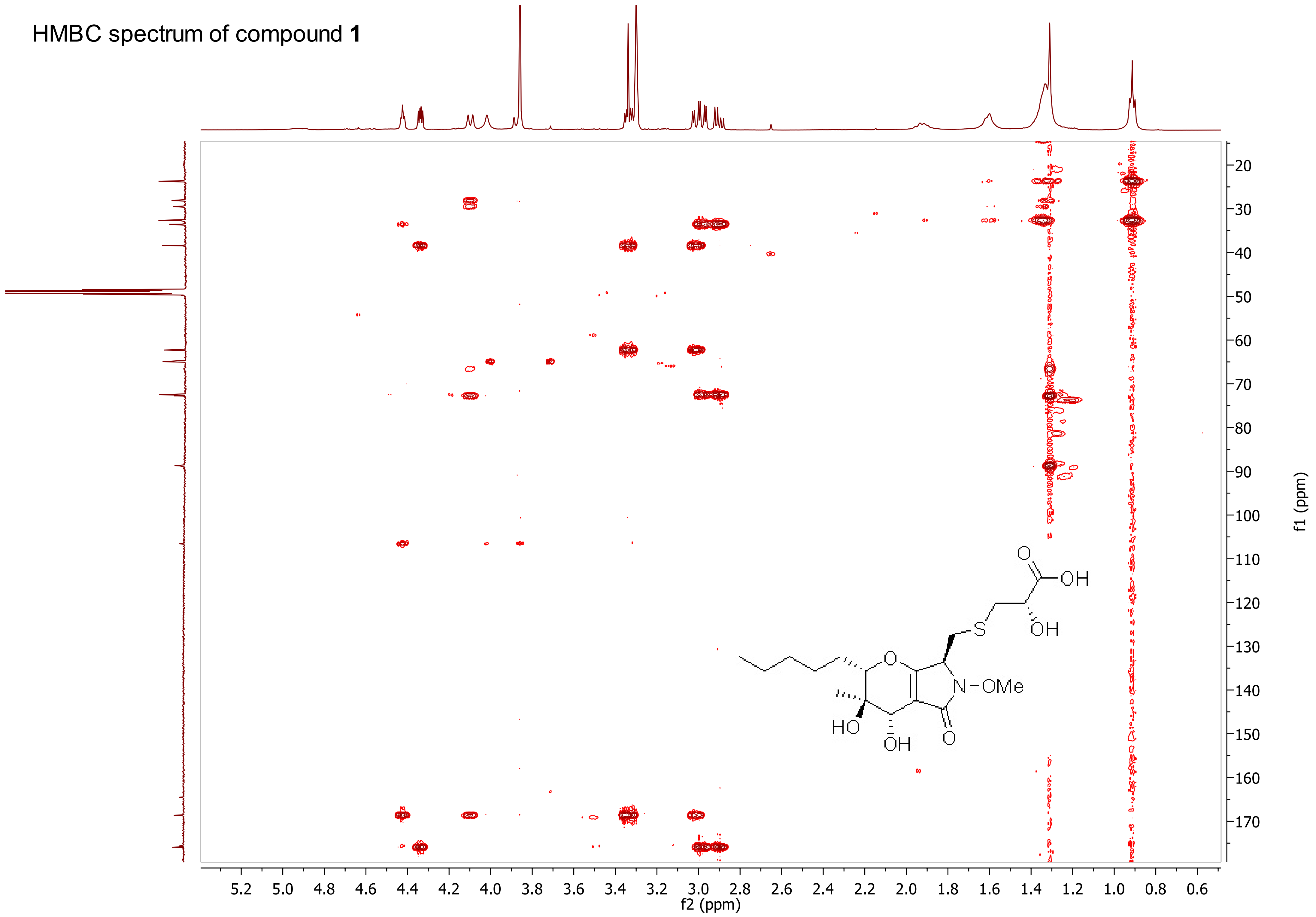

^{1}H NMR spectrum of compound **2** (500 MHz in methanol-d_4)

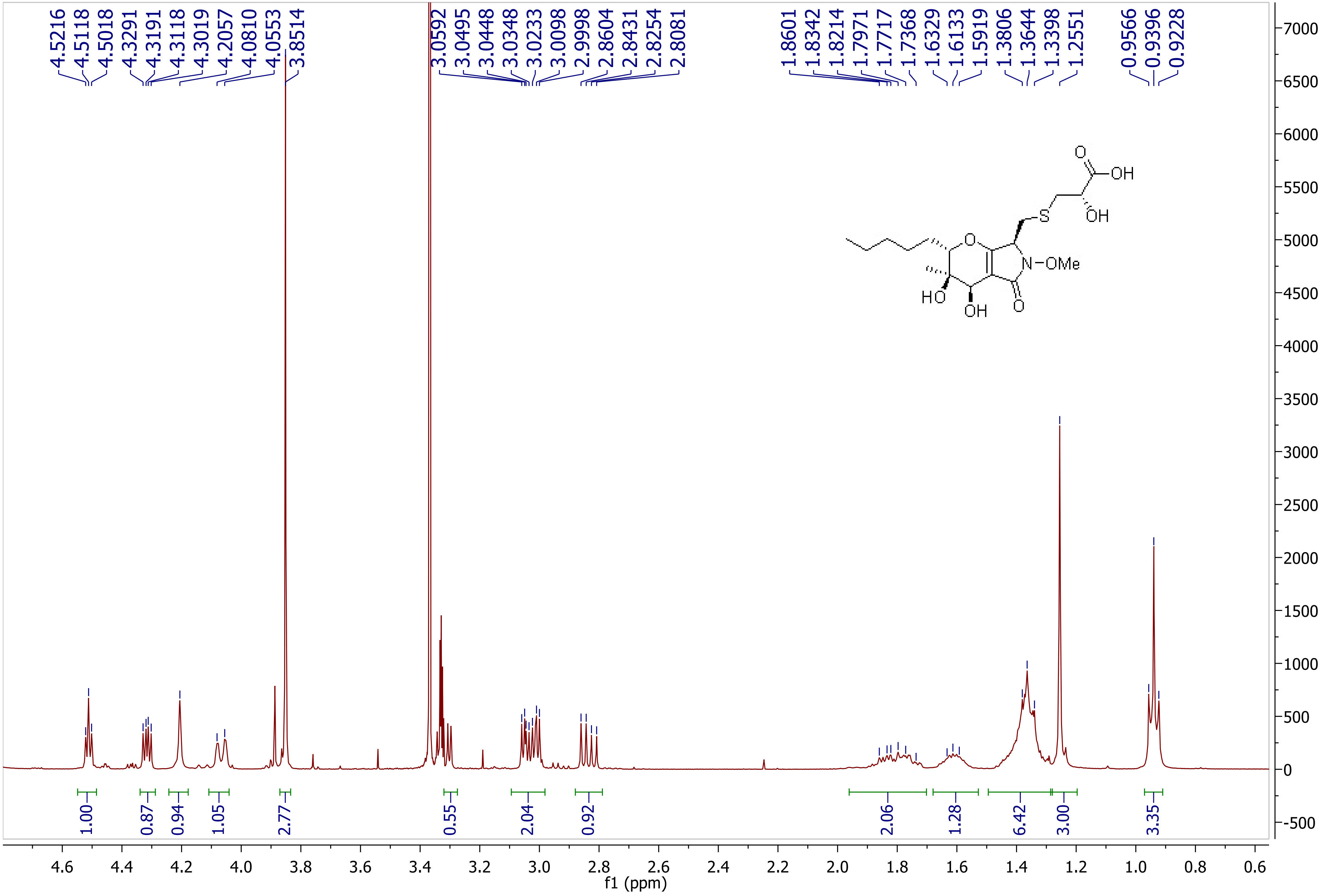

^{13}C NMR spectrum of compound **2**

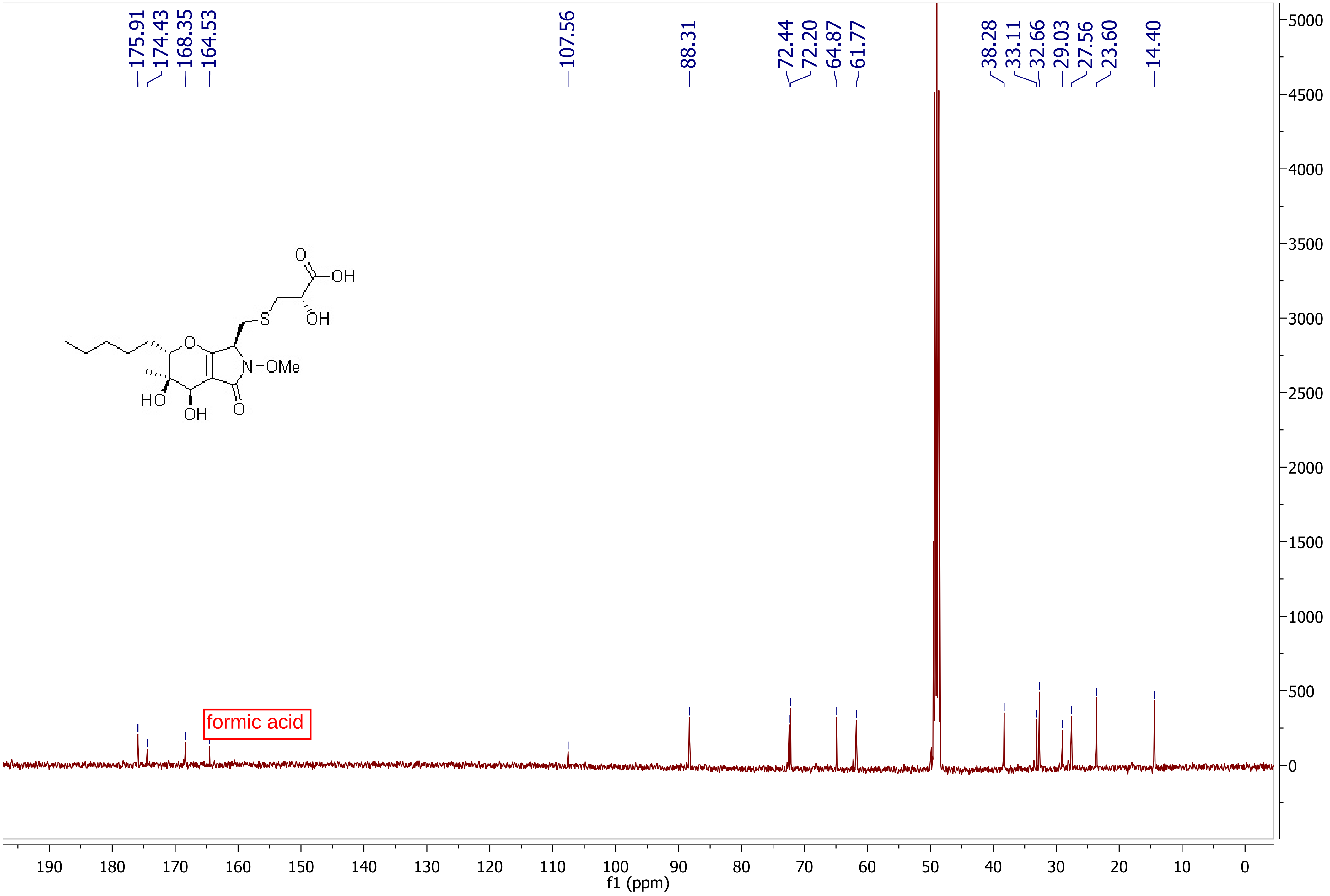

^{1}H-^{1}H COSY of compound **2**

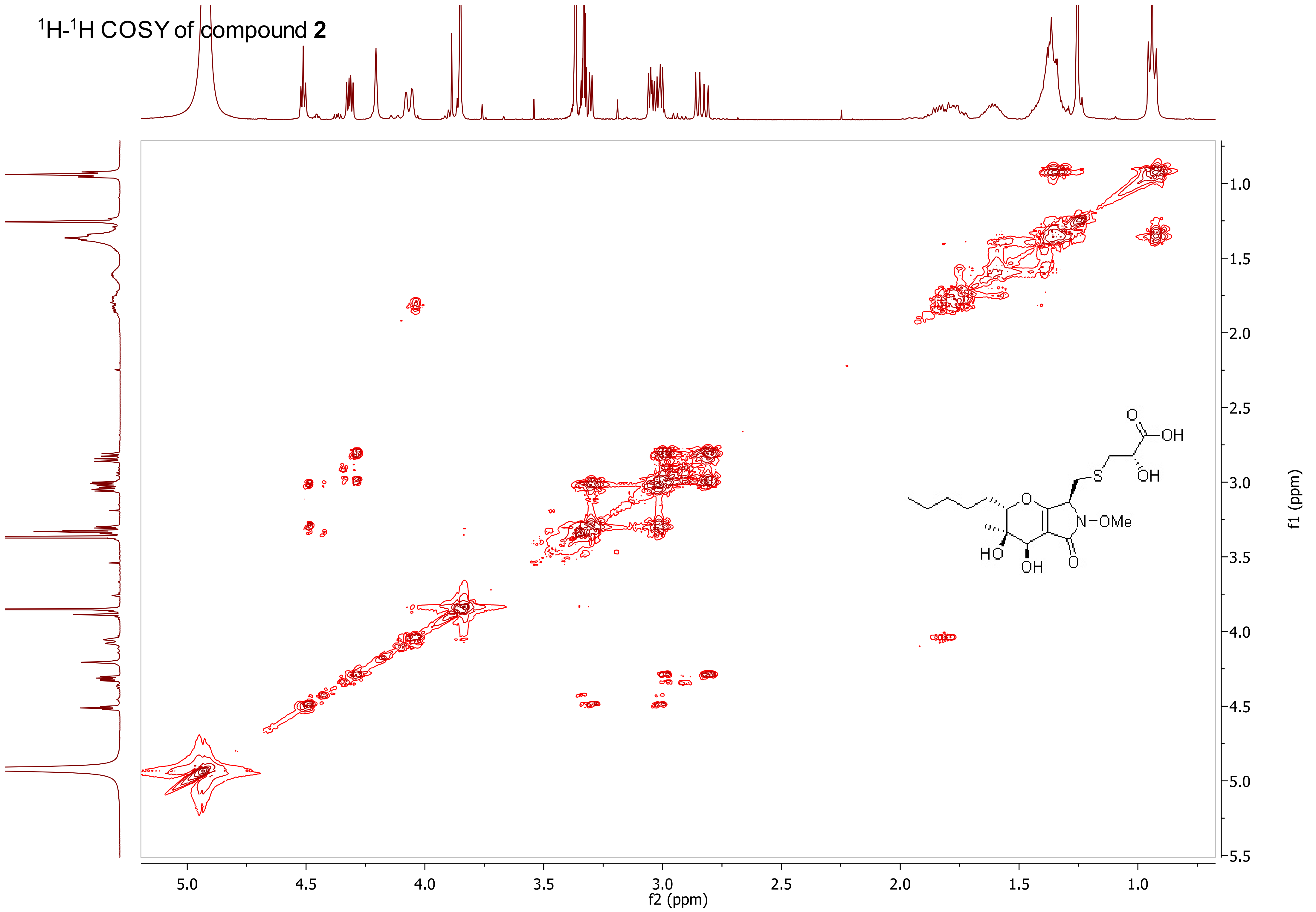

HSQC NMR spectrum of compound **2**

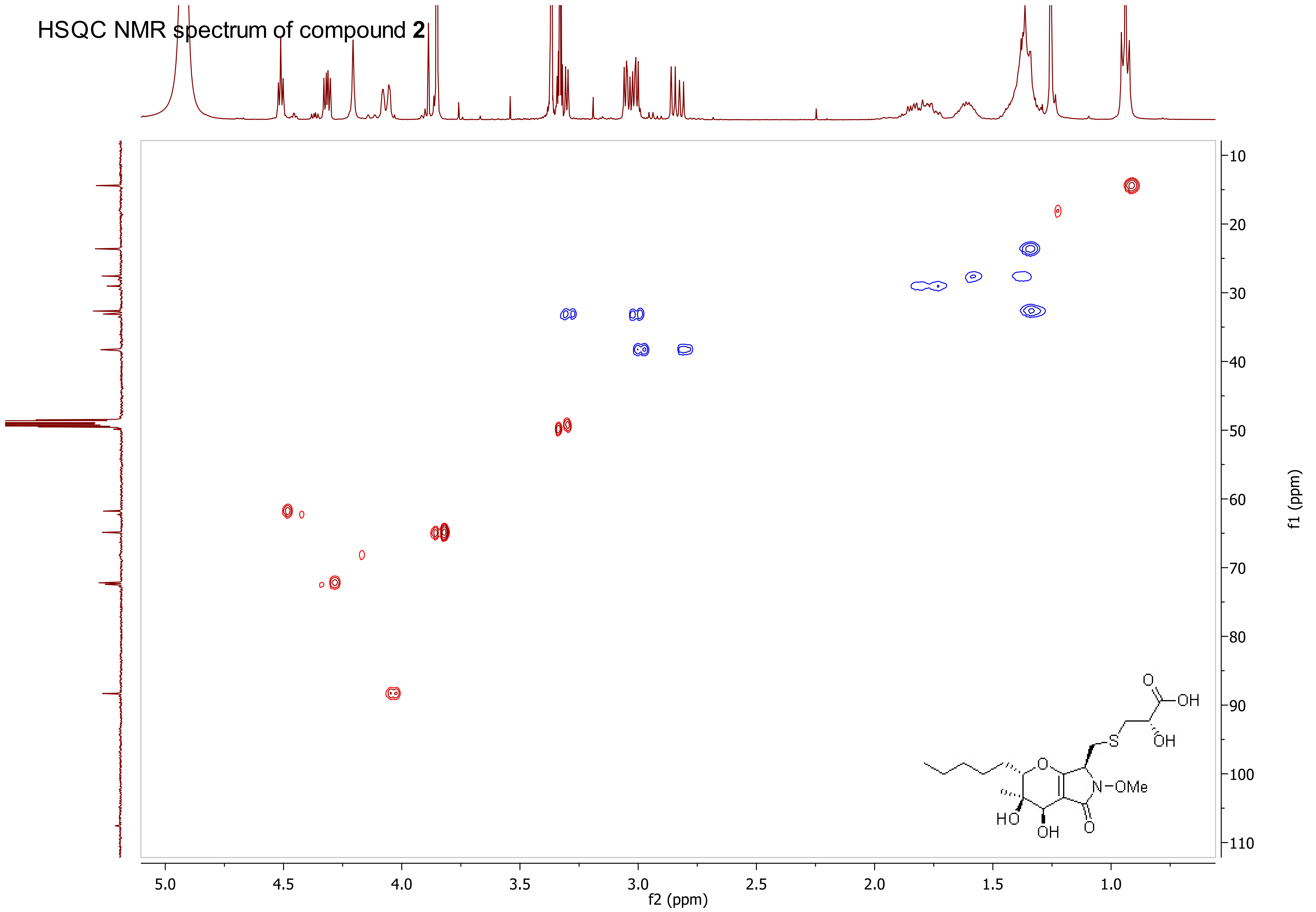

HMBC NMR spectrum of compound **2**

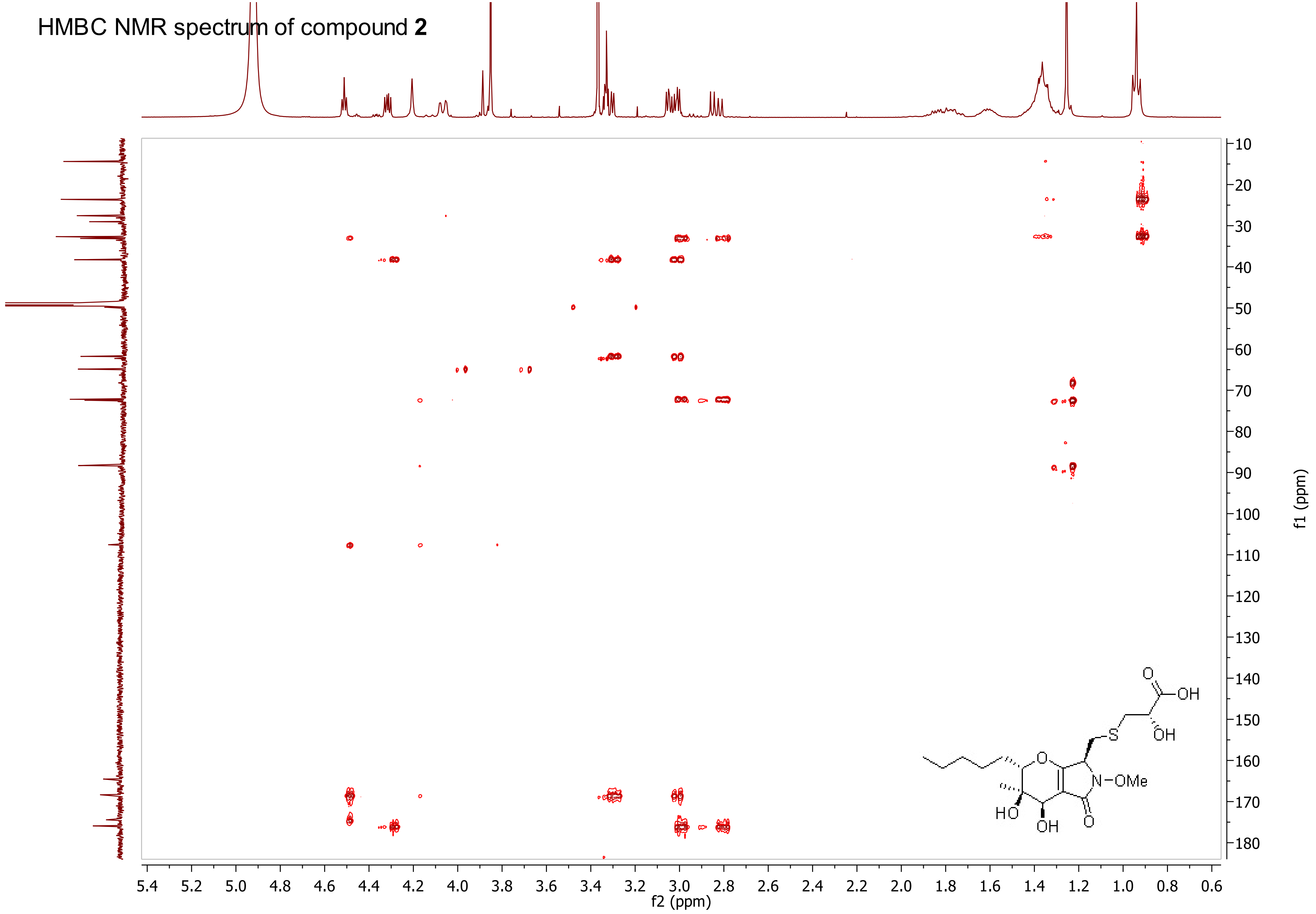

^{1}H NMR spectrum of compound **3** (400 MHz in methanol-d_4)

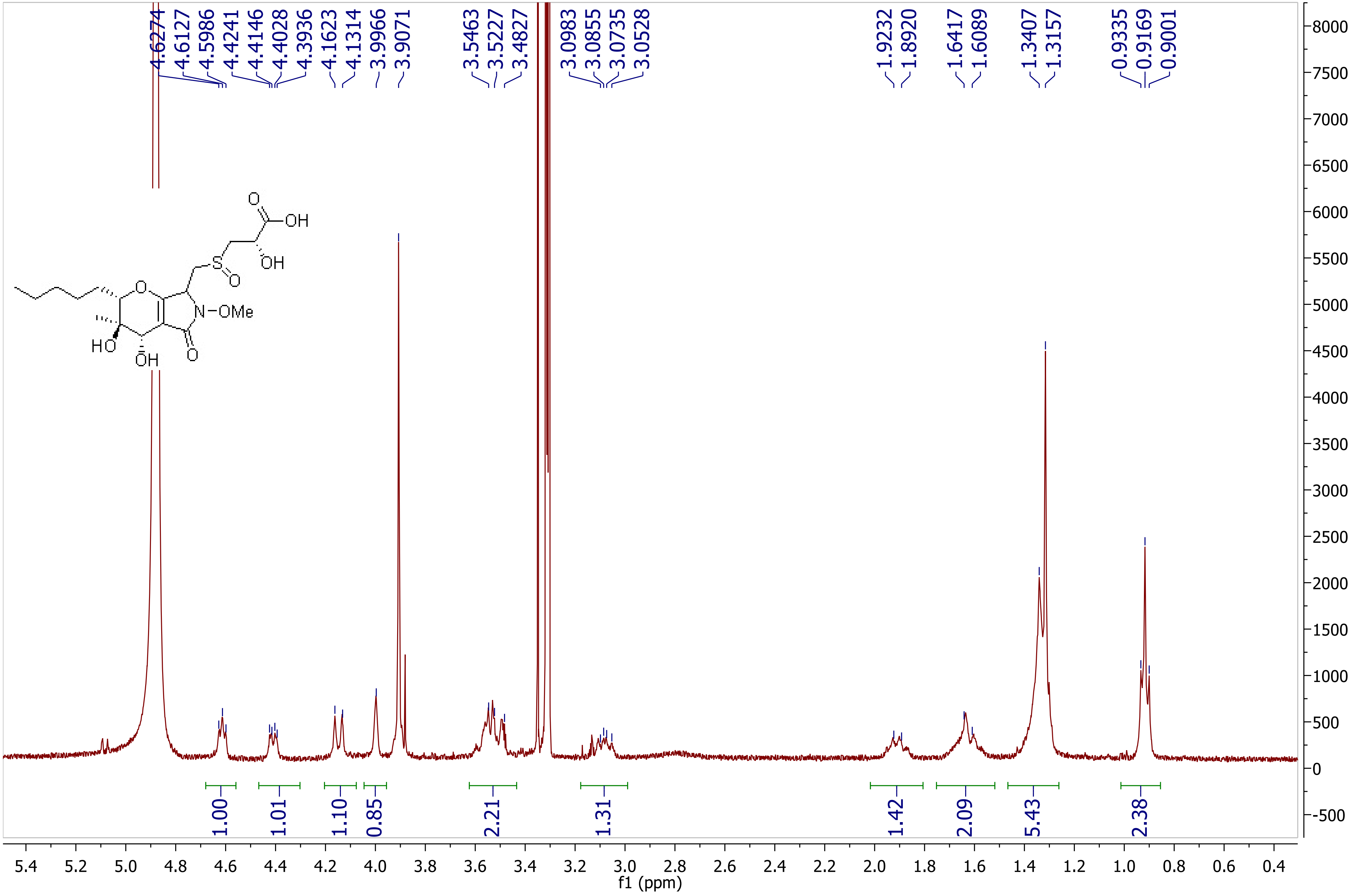

^{1}H-^{1}H COSY of compound **3**

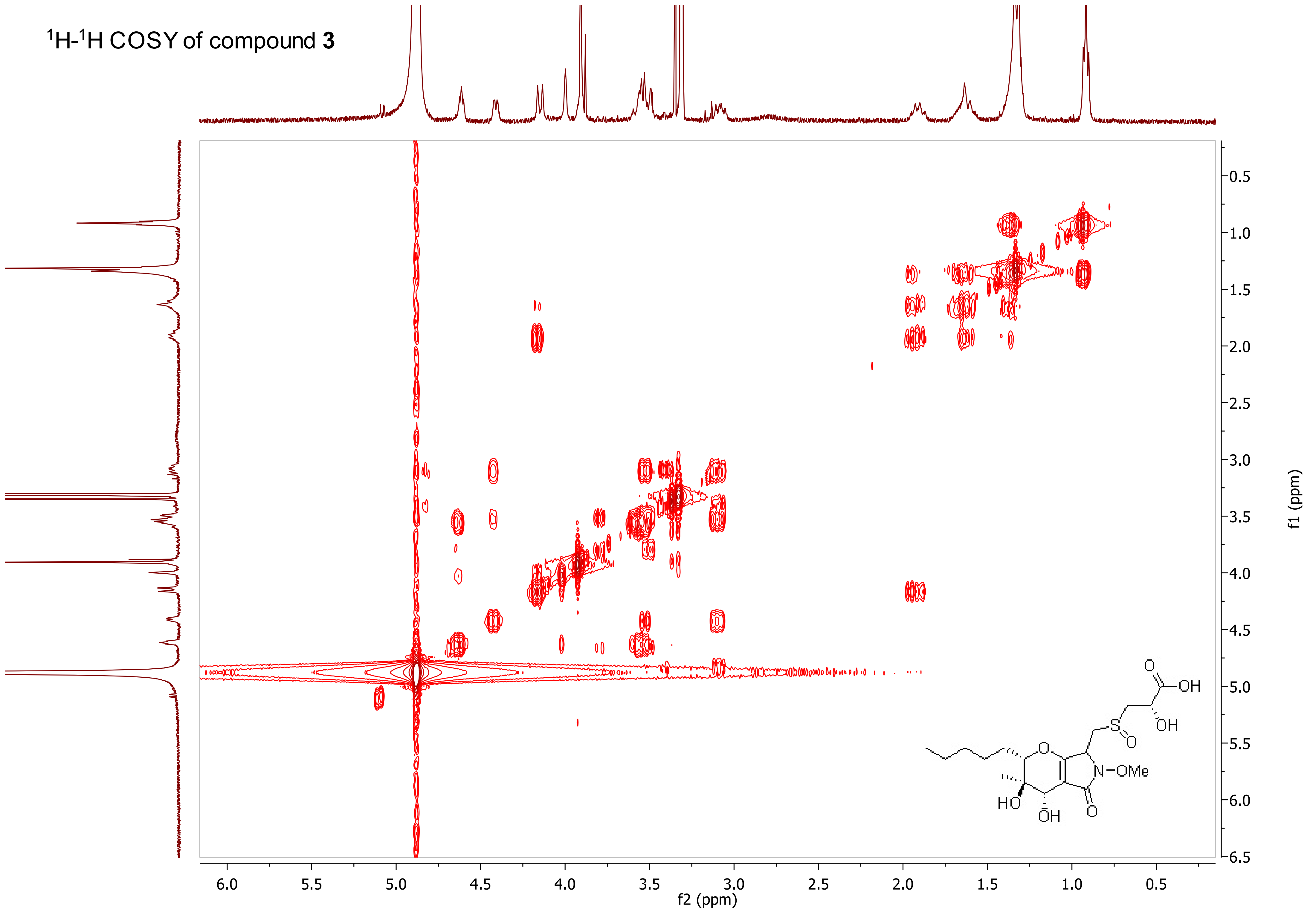

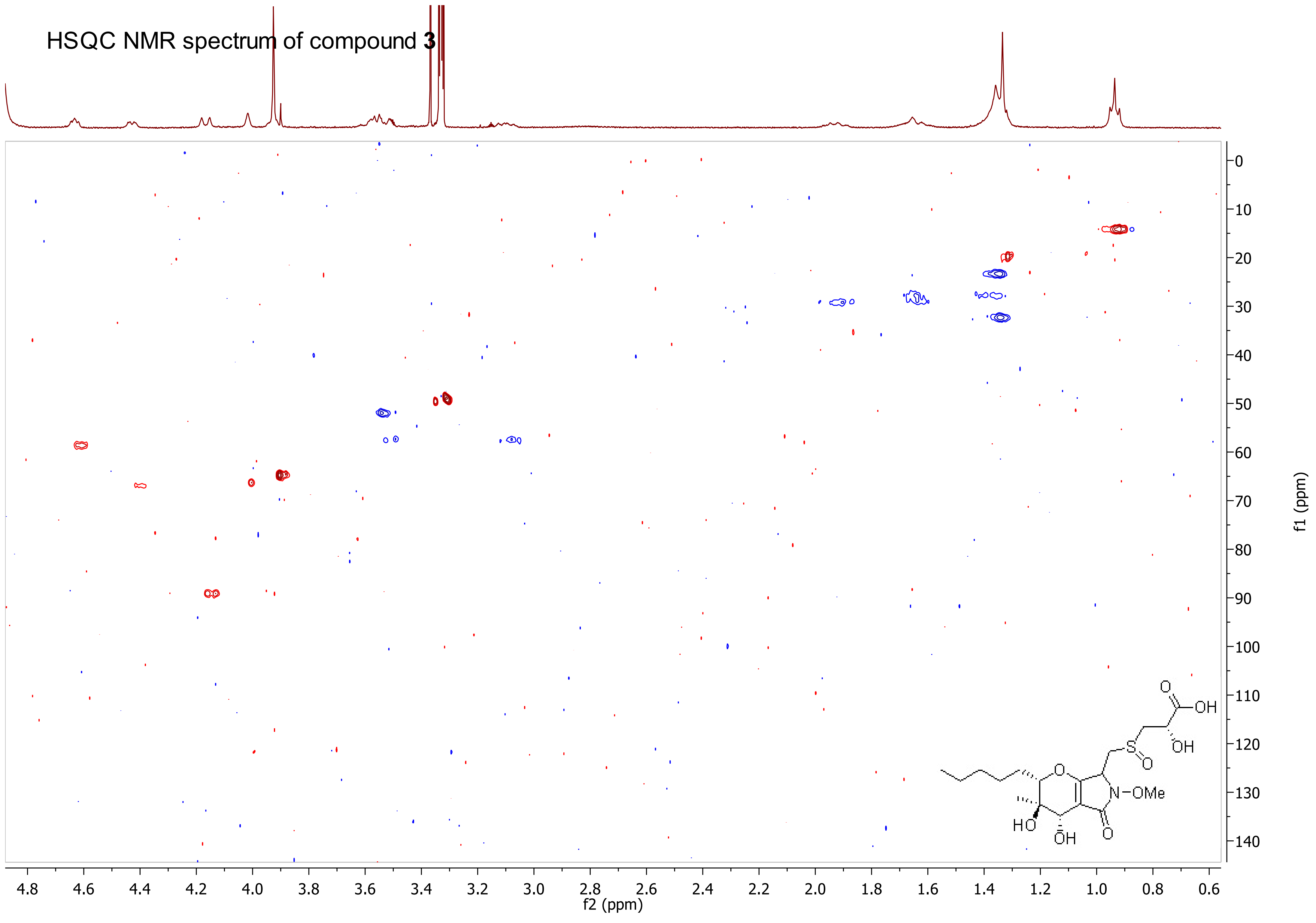

HSQC NMR spectrum of compound 3
f2 (ppm)
f1 (ppm)
4.8
4.6
4.4
4.2
4.0
3.8
3.6
3.4
3.2
3.0
2.8
2.6
2.4
2.2
2.0
1.8
1.6
1.4
1.2
1.0
0.8
0.6
0
10
20
30
40
50
60
70
80
90
100
110
120
130
140
O
OH
OH
S
O
O
N−OMe
HO
OH
O

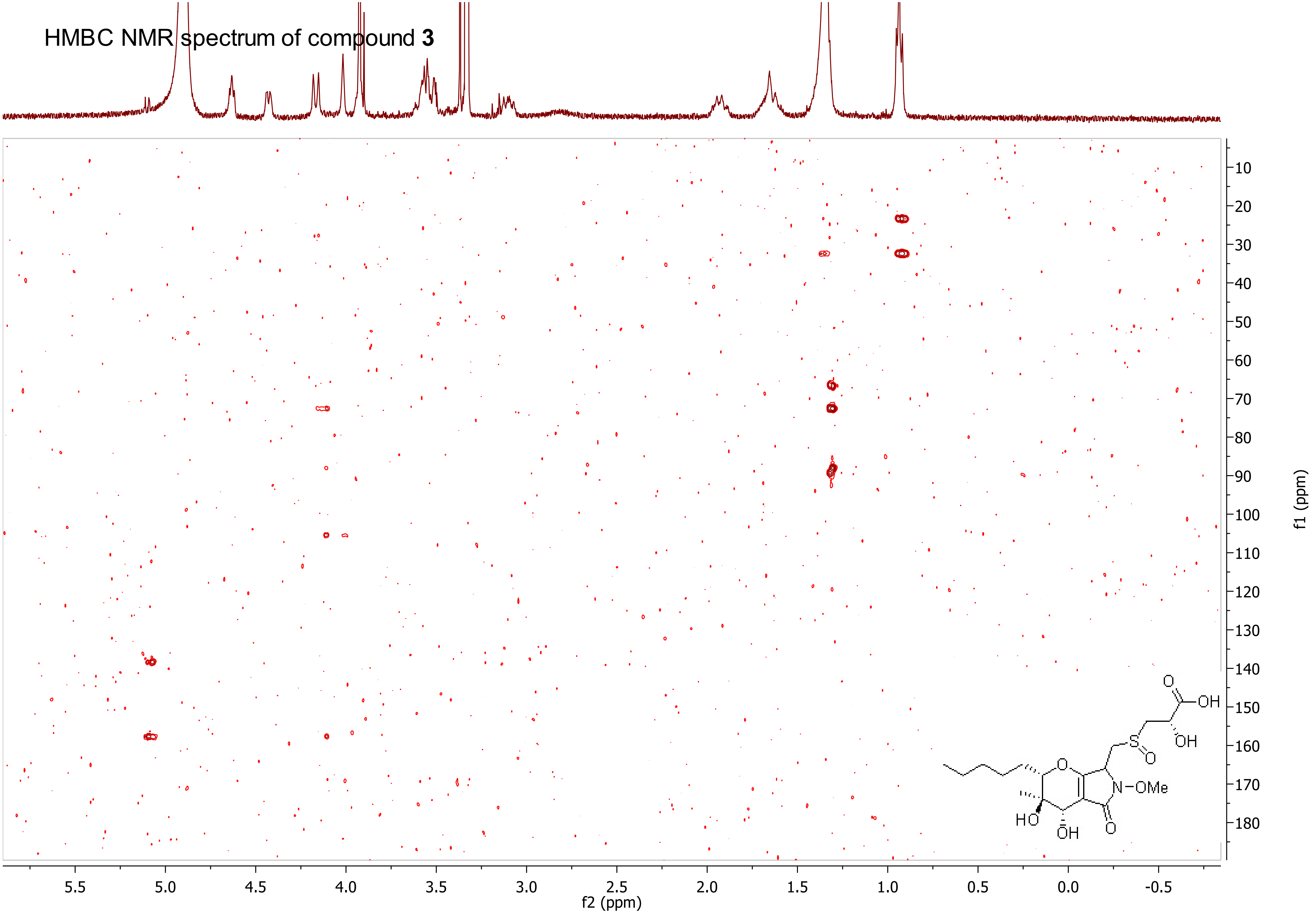
HMBC NMR spectrum of compound **3**
f2 (ppm)
f1 (ppm)
5.5
5.0
4.5
4.0
3.5
3.0
2.5
2.0
1.5
1.0
0.5
0.0
-0.5
10
20
30
40
50
60
70
80
90
100
110
120
130
140
150
160
170
180
O
OH
OH
S
O
O
N−OMe
HO
OH
O

^{1}H NMR spectrum of compound **4** (400 MHz in methanol-d_4)

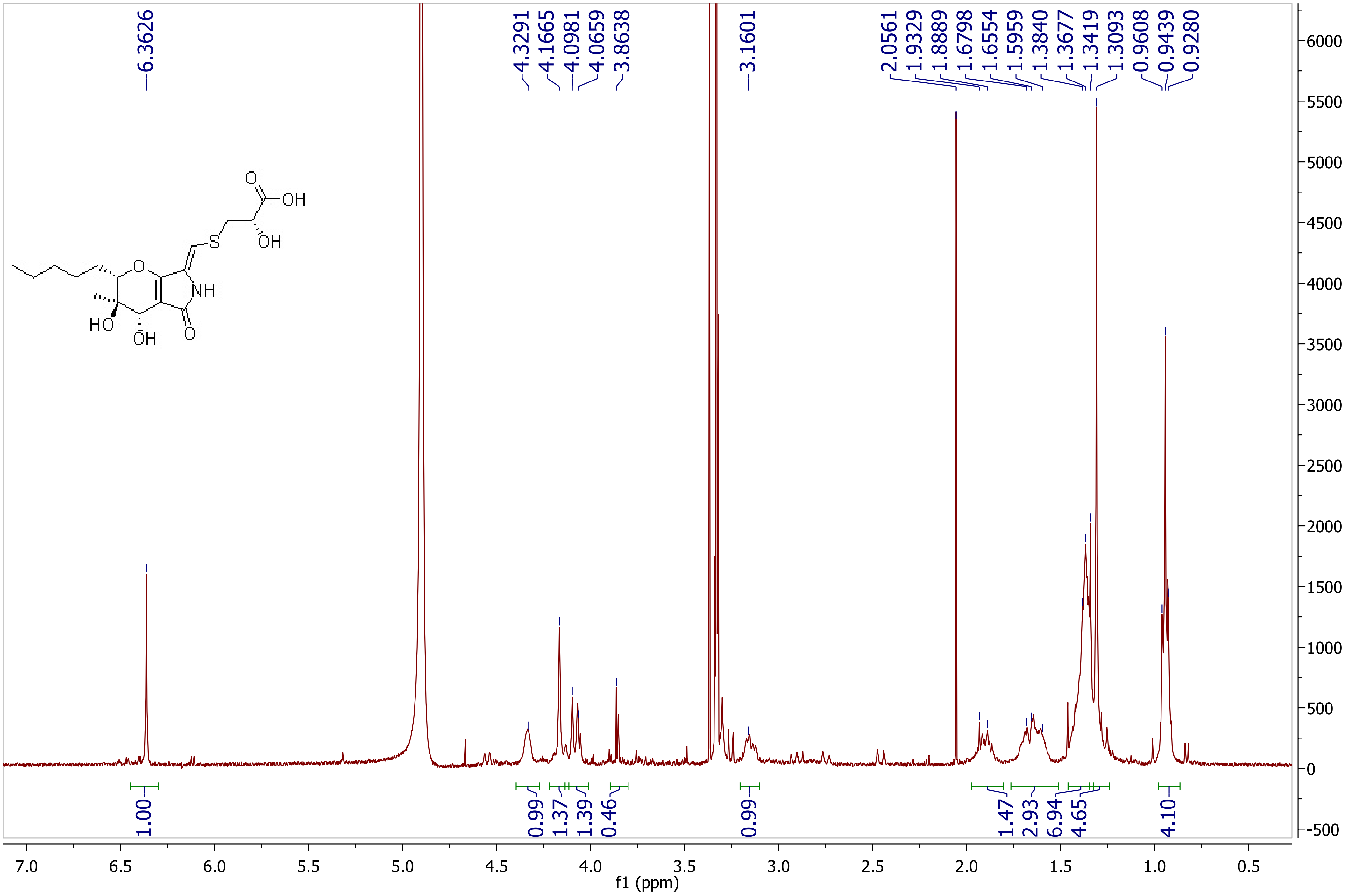

^{13}C NMR spectrum of compound **4**

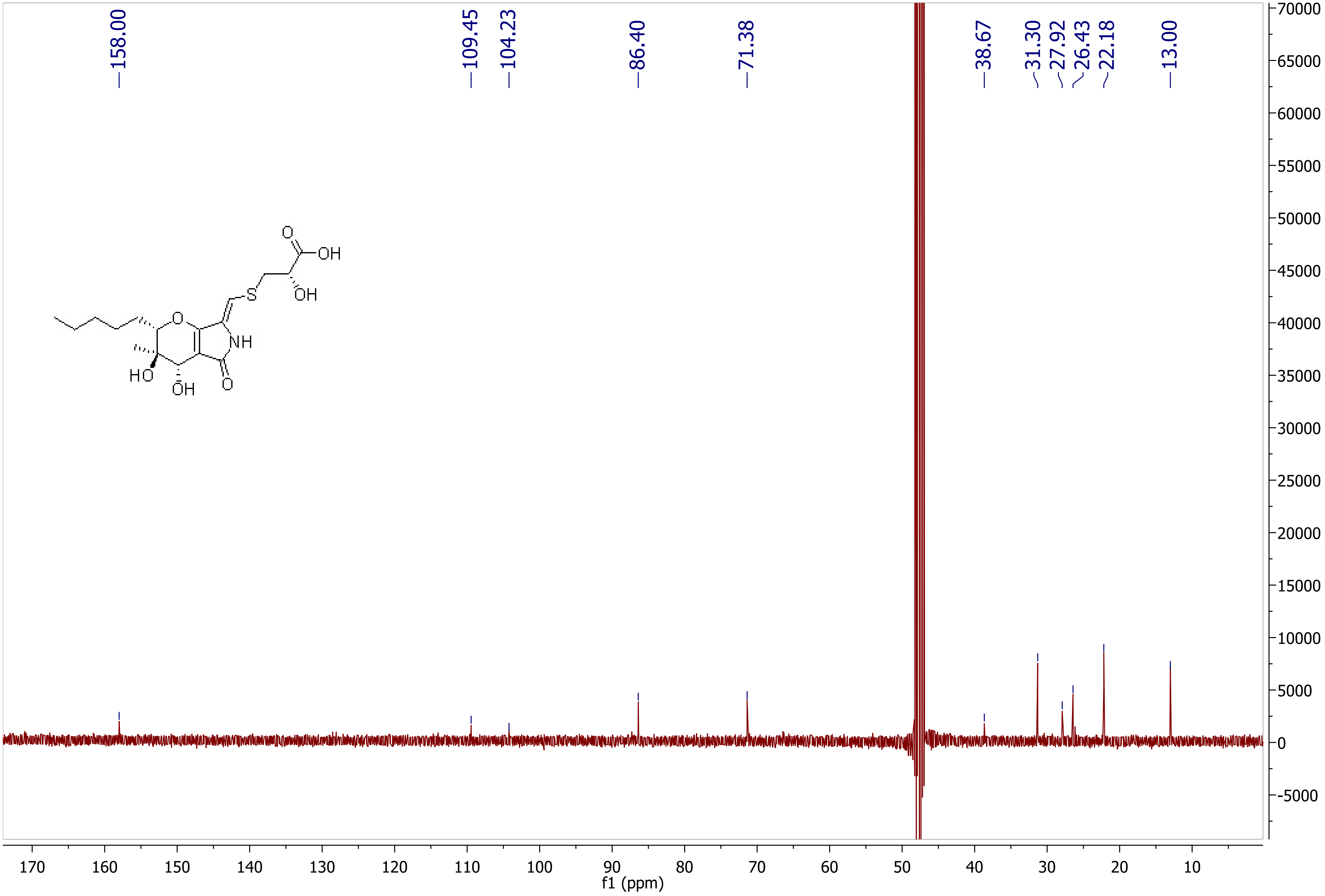

^{1}H-^{1}H COSY of compound **4**

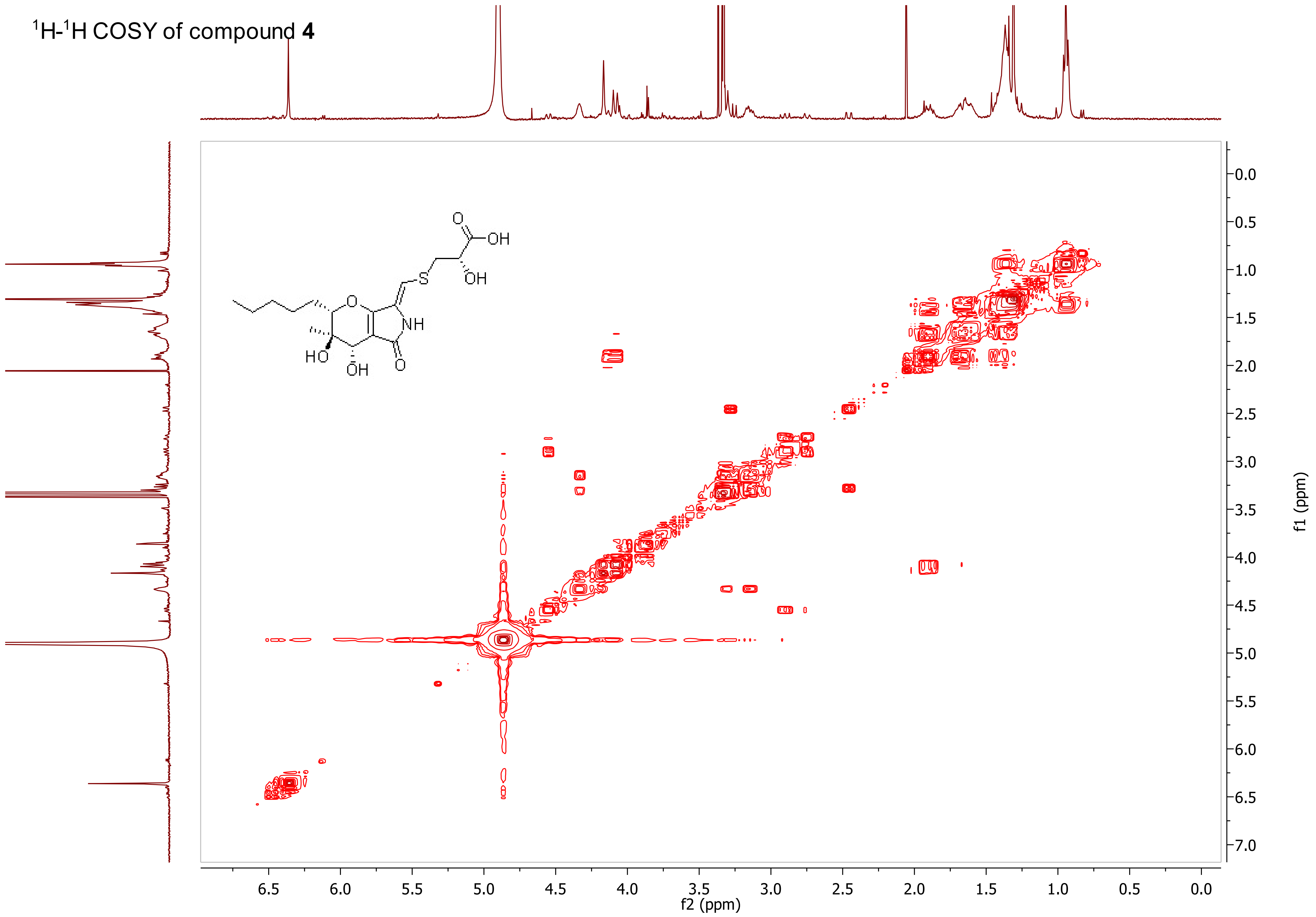

HSQC NMR spectrum of compound **4**

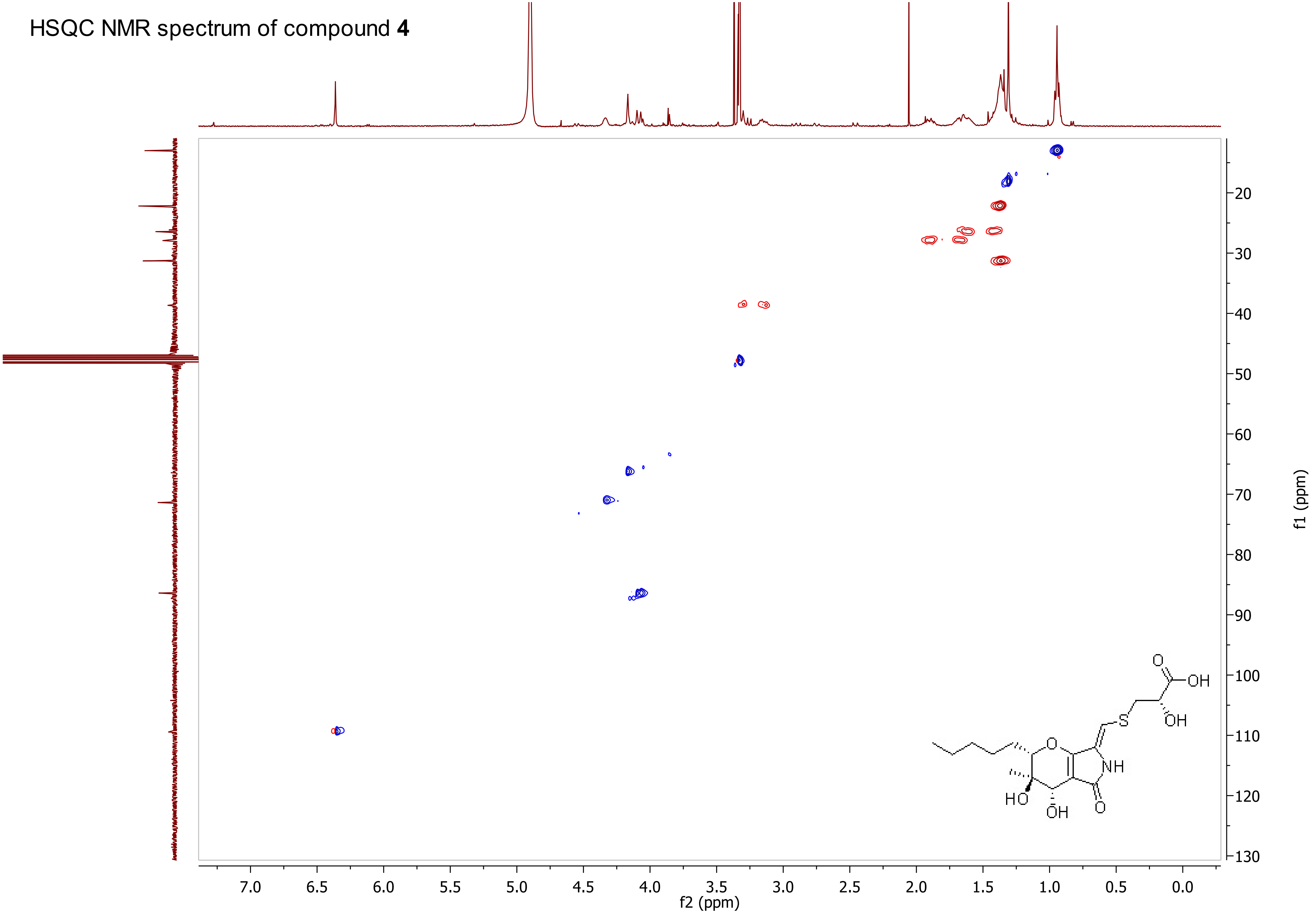

HMBC spectrum of compound **4**

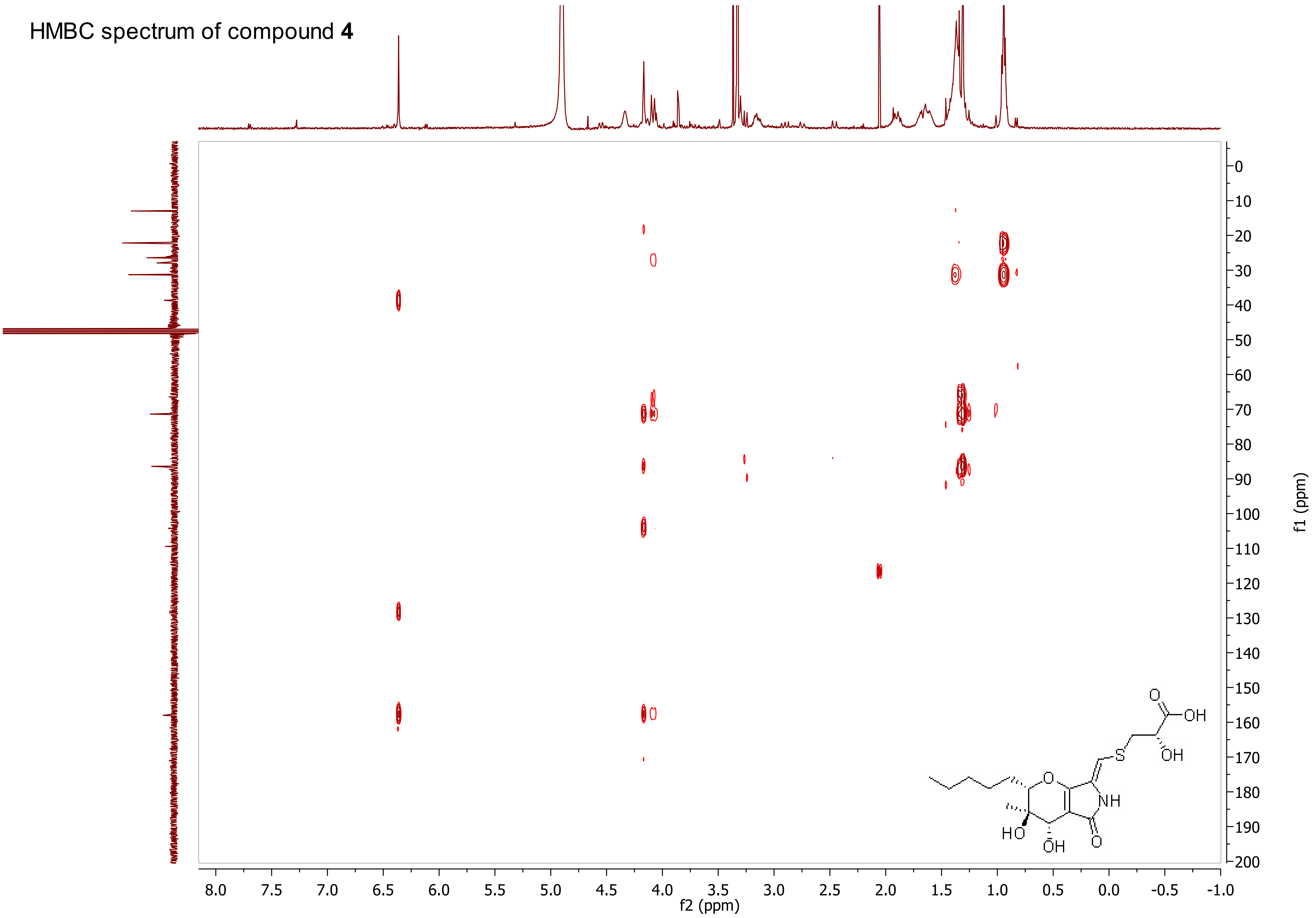

^{1}H NMR spectrum of compound **5** (400 MHz in methanol-d_4)

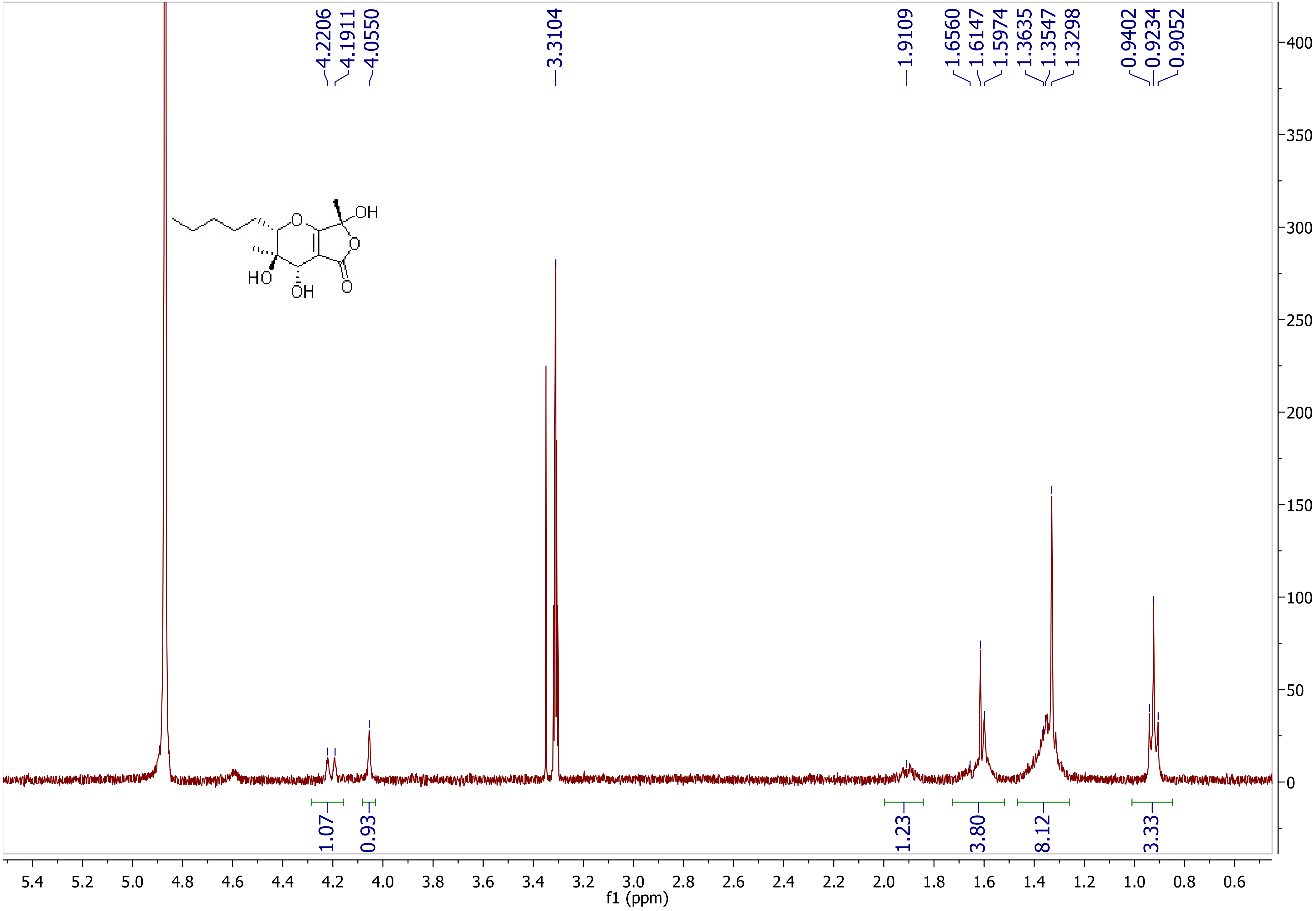

^{13}C NMR spectrum of compound **5**

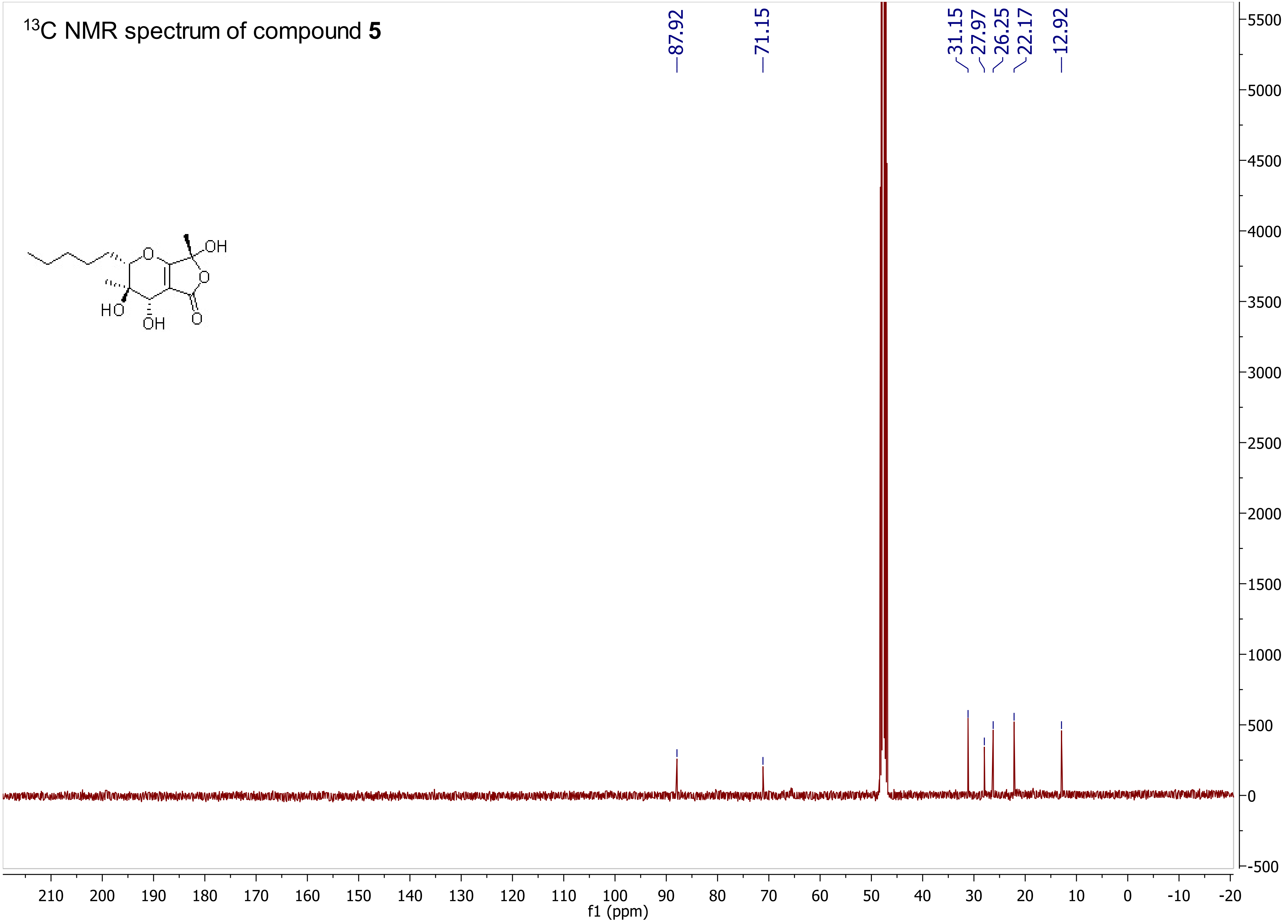

HSQC spectrum of compound **5**

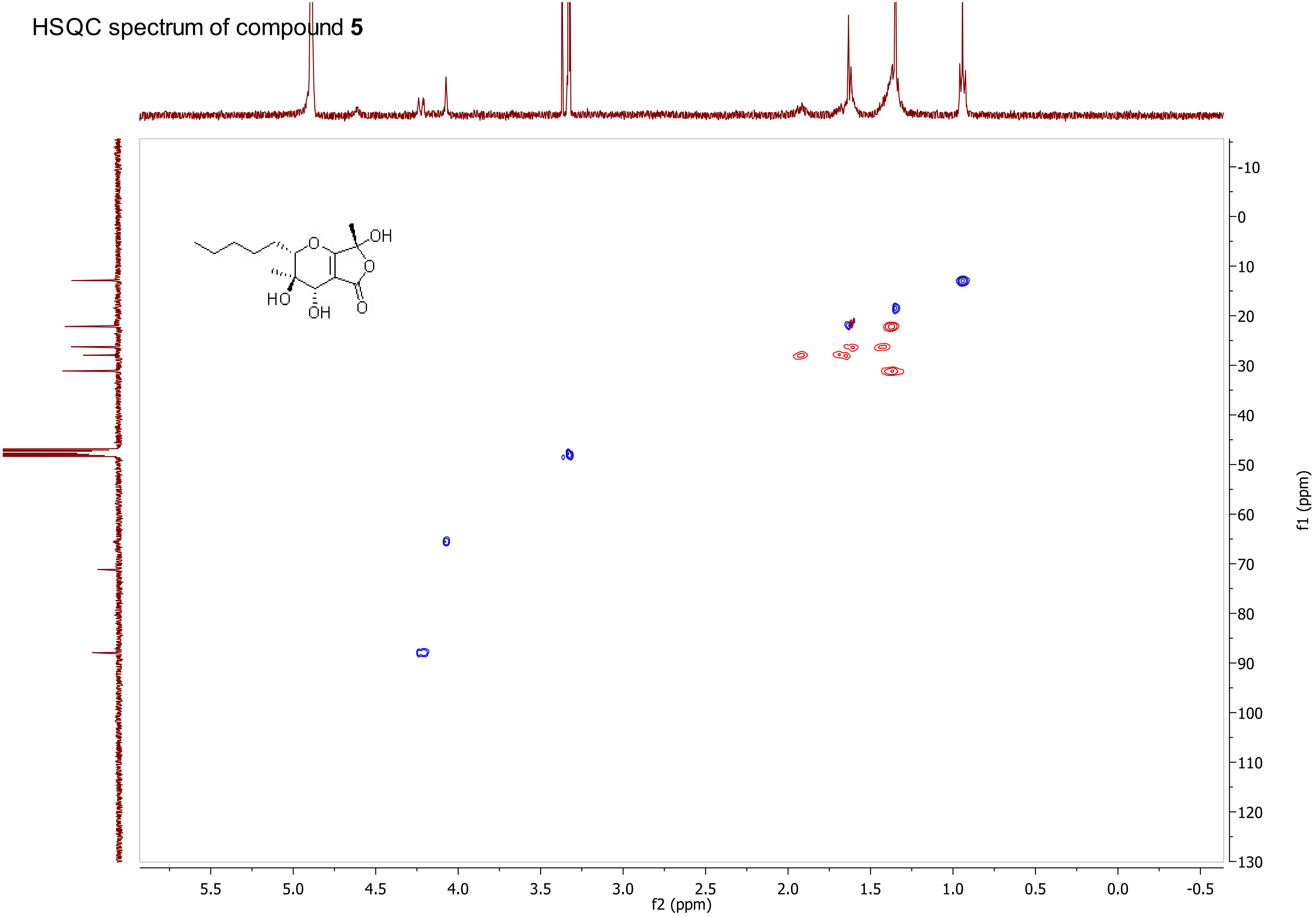

HMBC spectrum of compound **5**

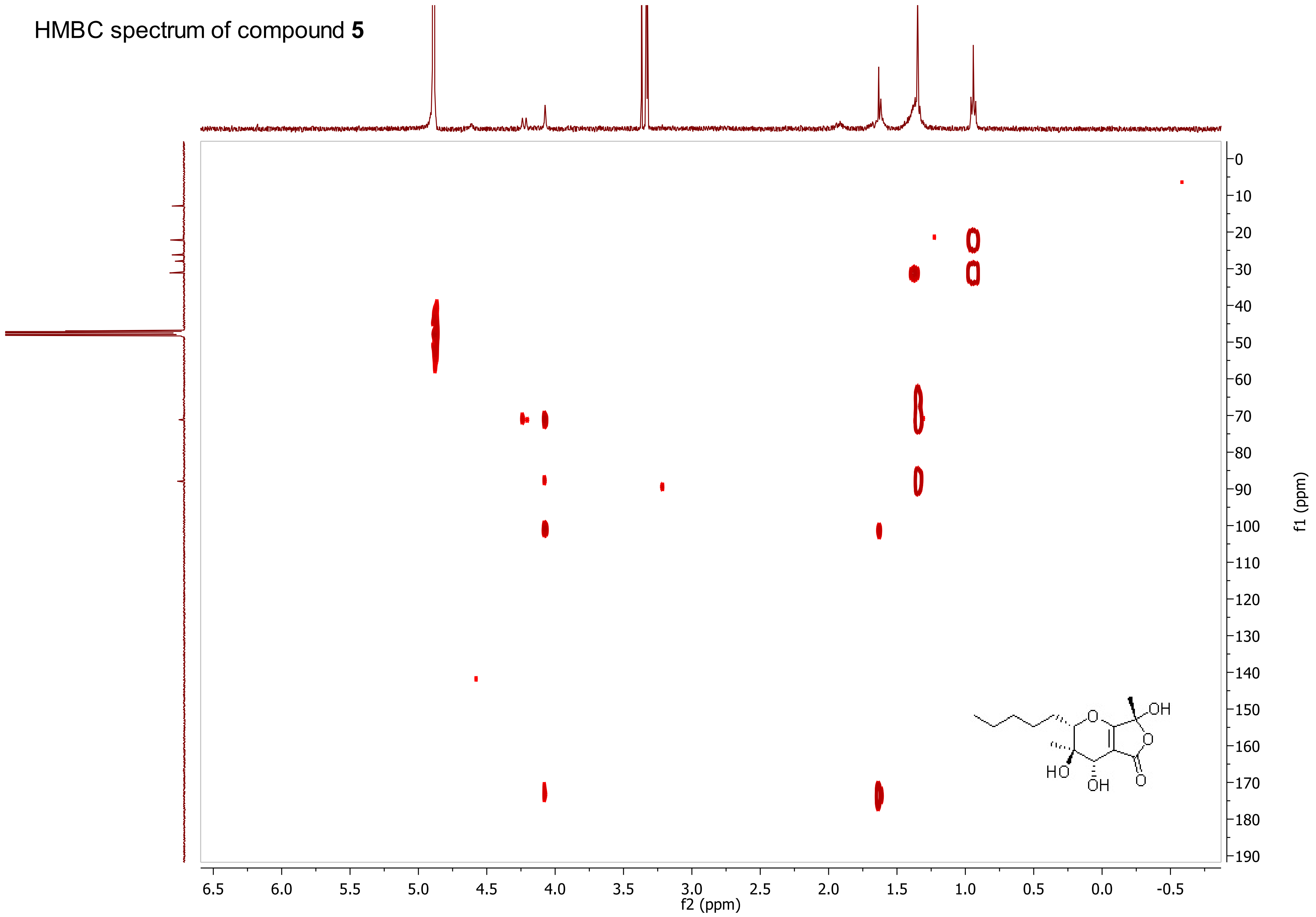

^{1}H NMR spectrum of compound **7** (*R*-MTPA of **1**) (400 MHz in methanol-d_4)

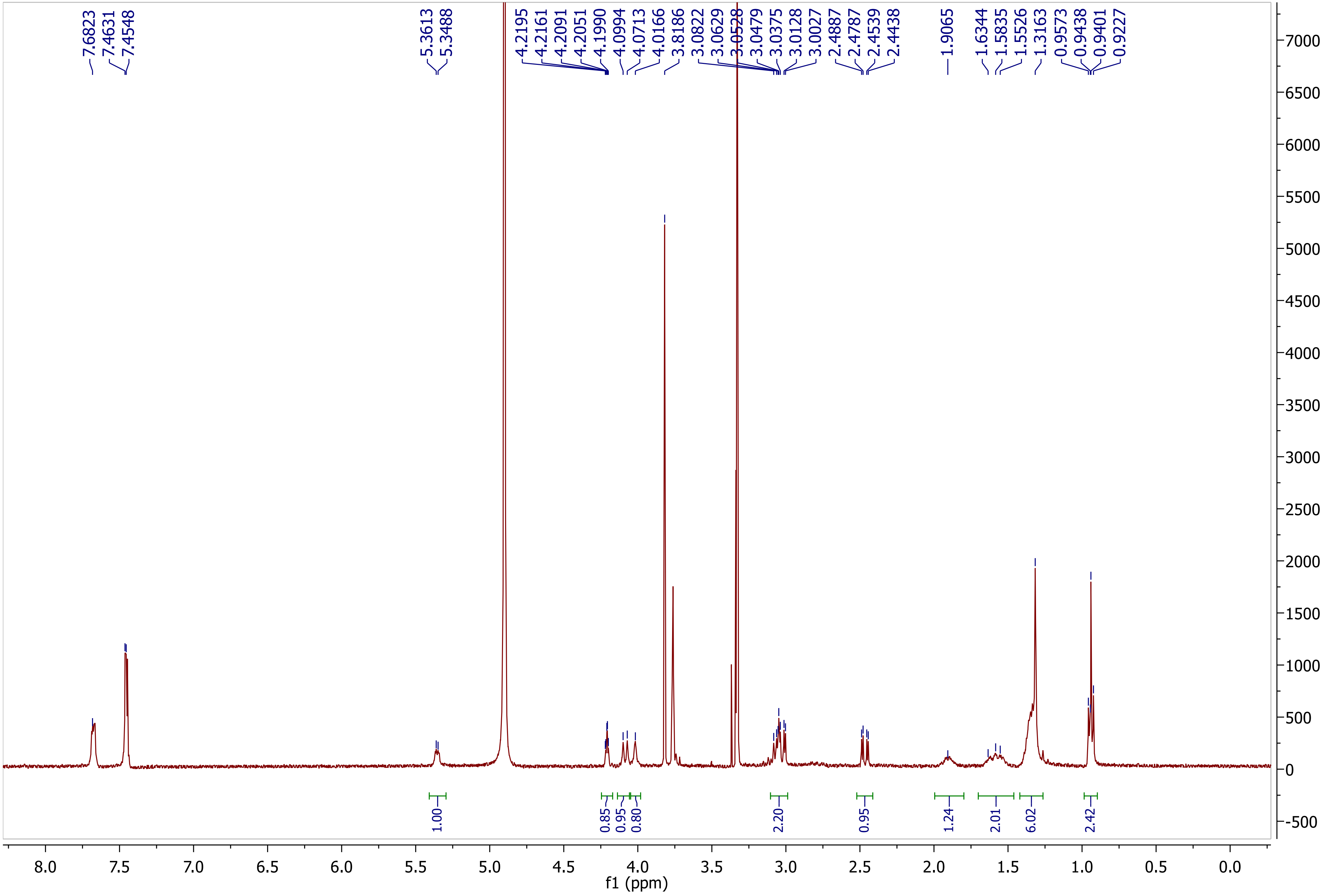

^{1}H NMR spectrum of compound **8** (*S*-MTPA of **1**) (400 MHz in methanol-d_4)

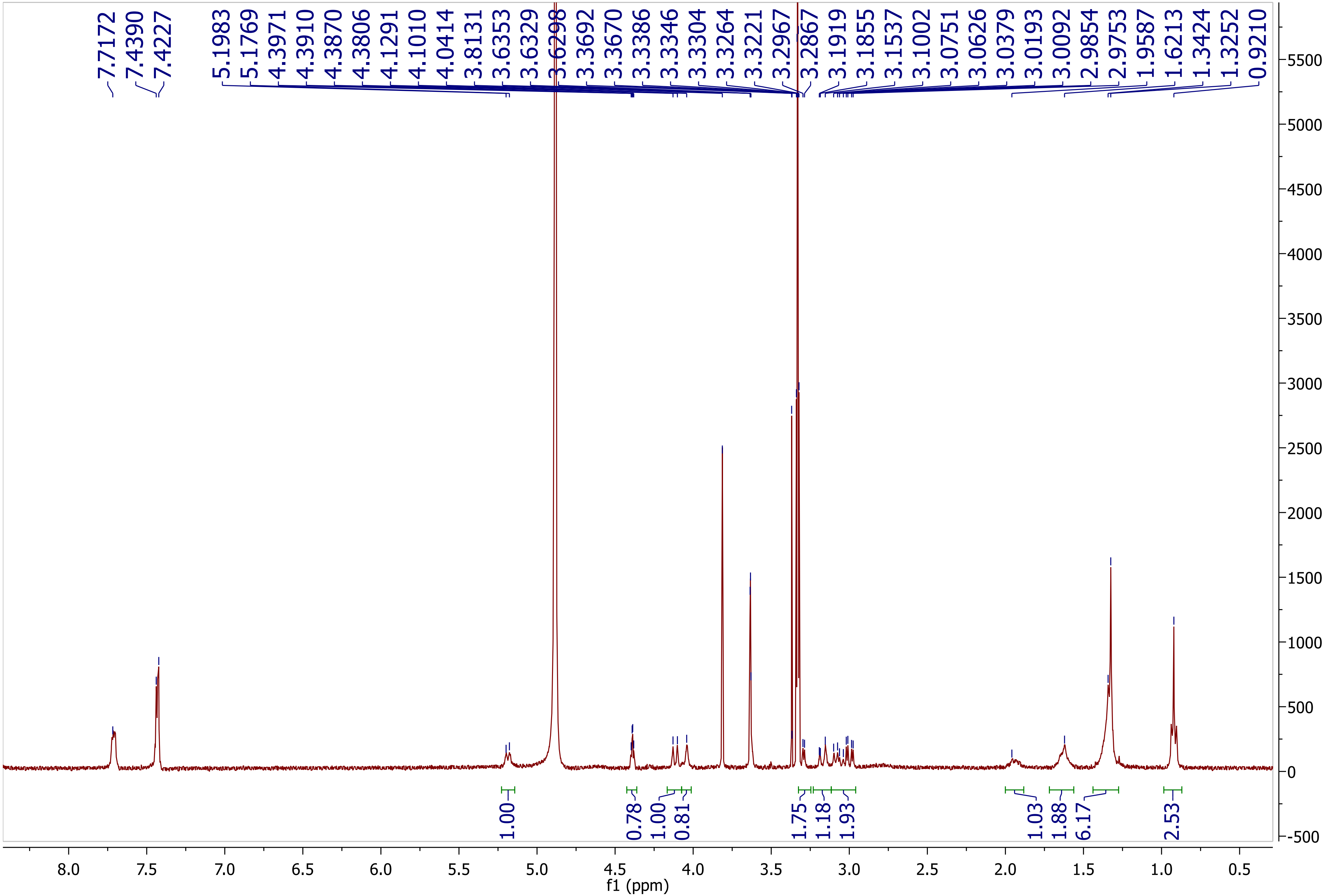

^{1}H NMR spectrum of compound **9** (*R*-MTPA of **1**) (400 MHz in methanol-d_4)

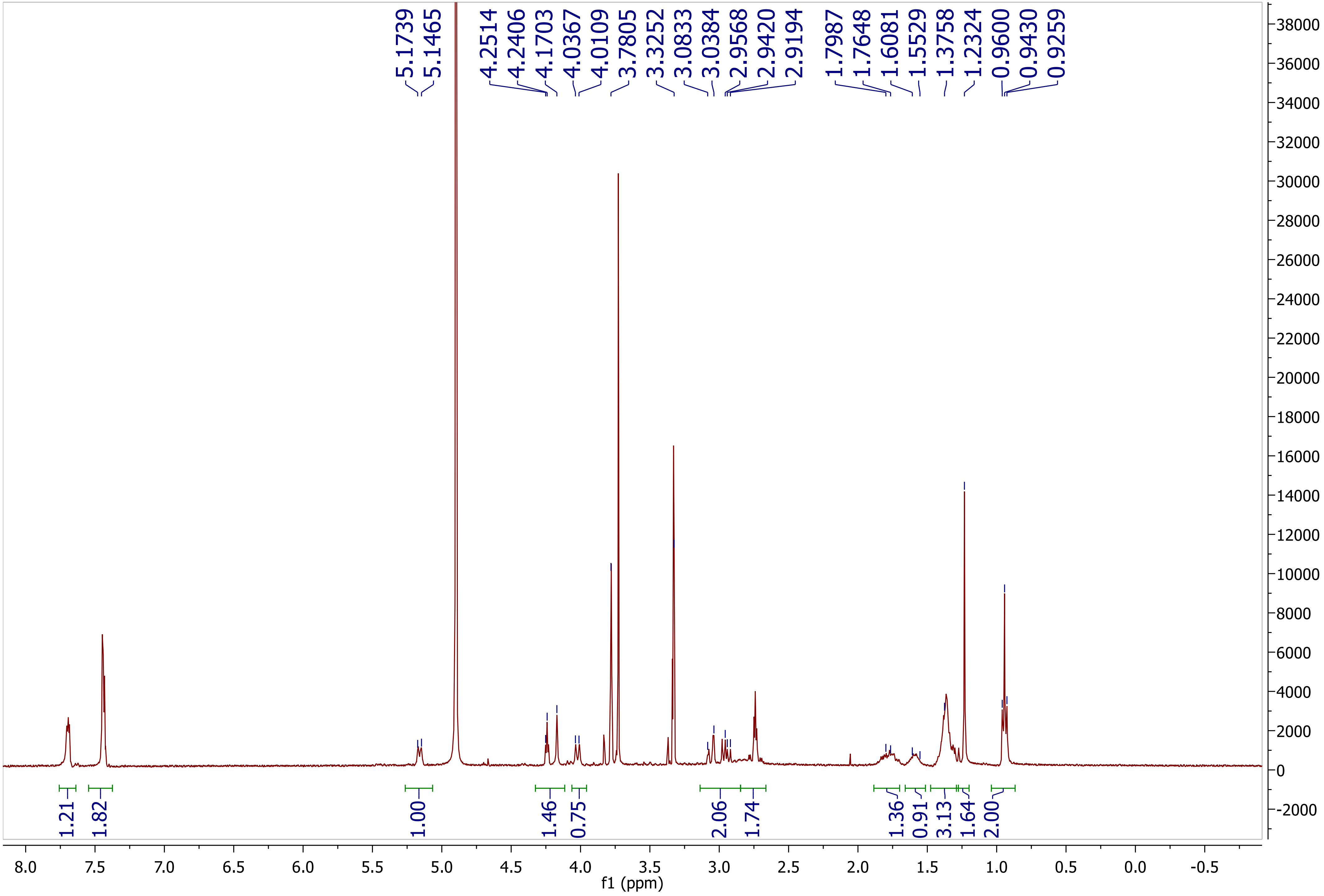

^{1}H-^{1}H COSY of compound **9** (*R*-MTPA of **2**)

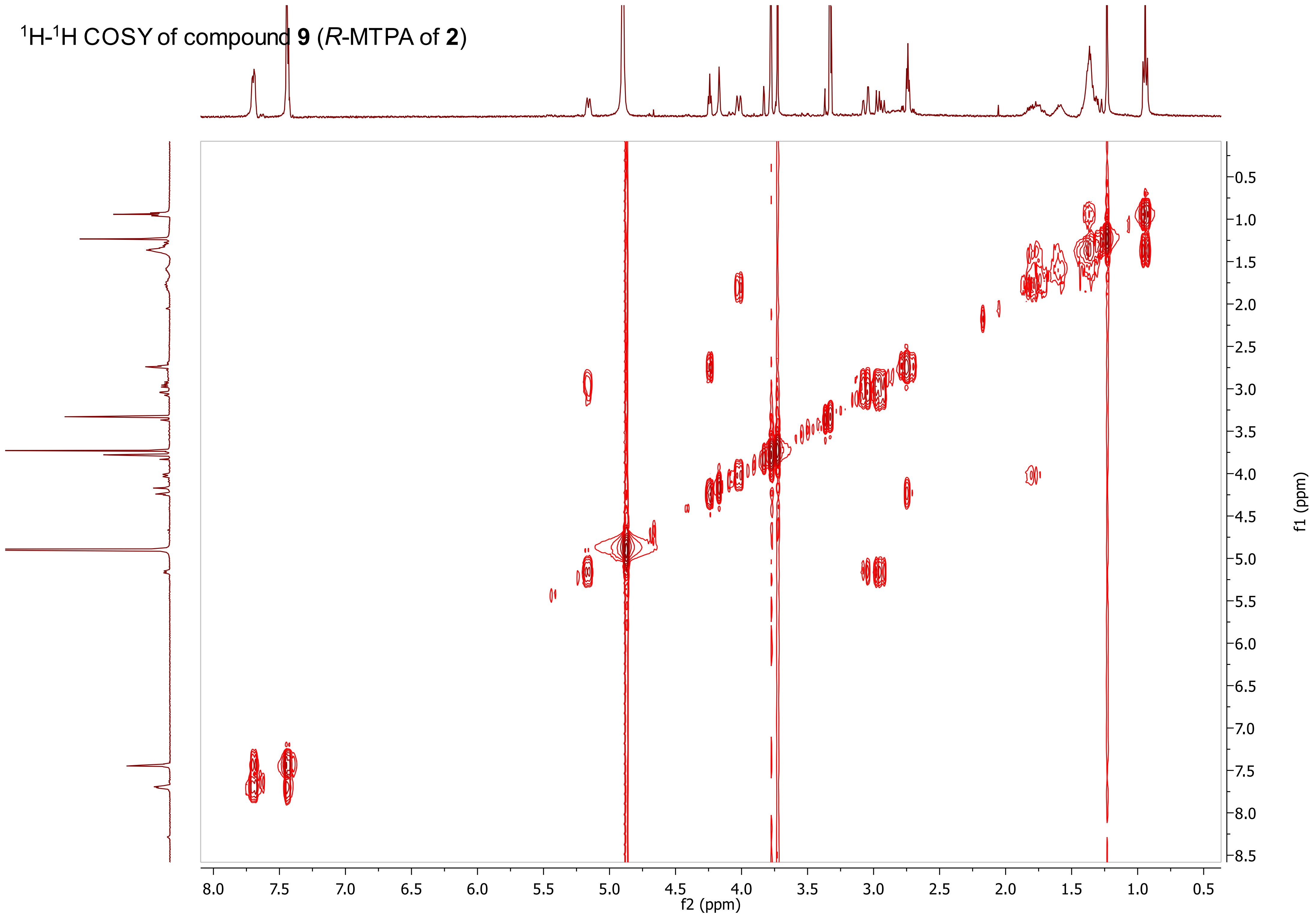

^{1}H NMR spectrum of compound **10** (*S*-MTPA of **2**) (400 MHz in methanol-d_4)

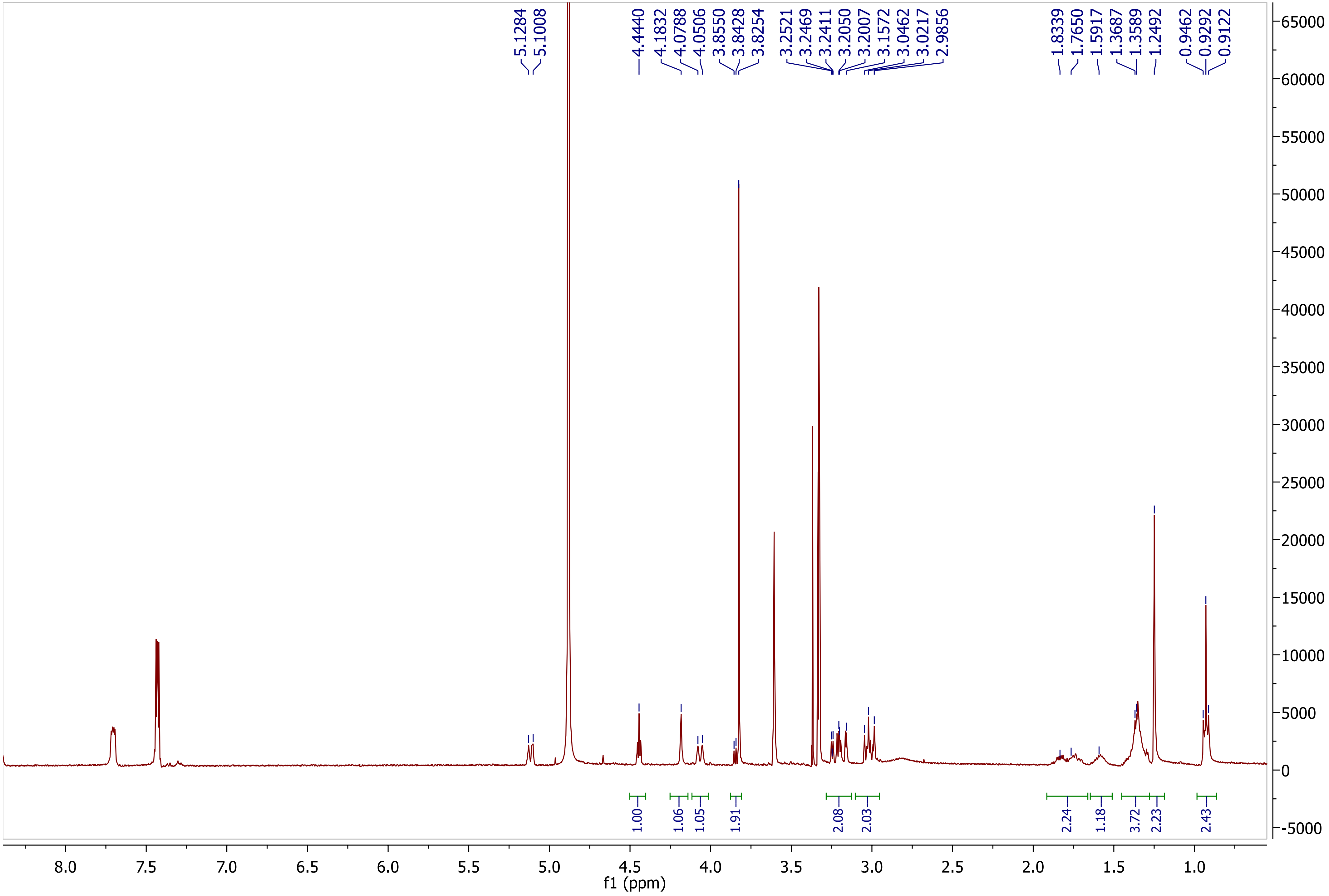

^1H-^1H COSY of compound **10** (*S*-MTPA of **2**)

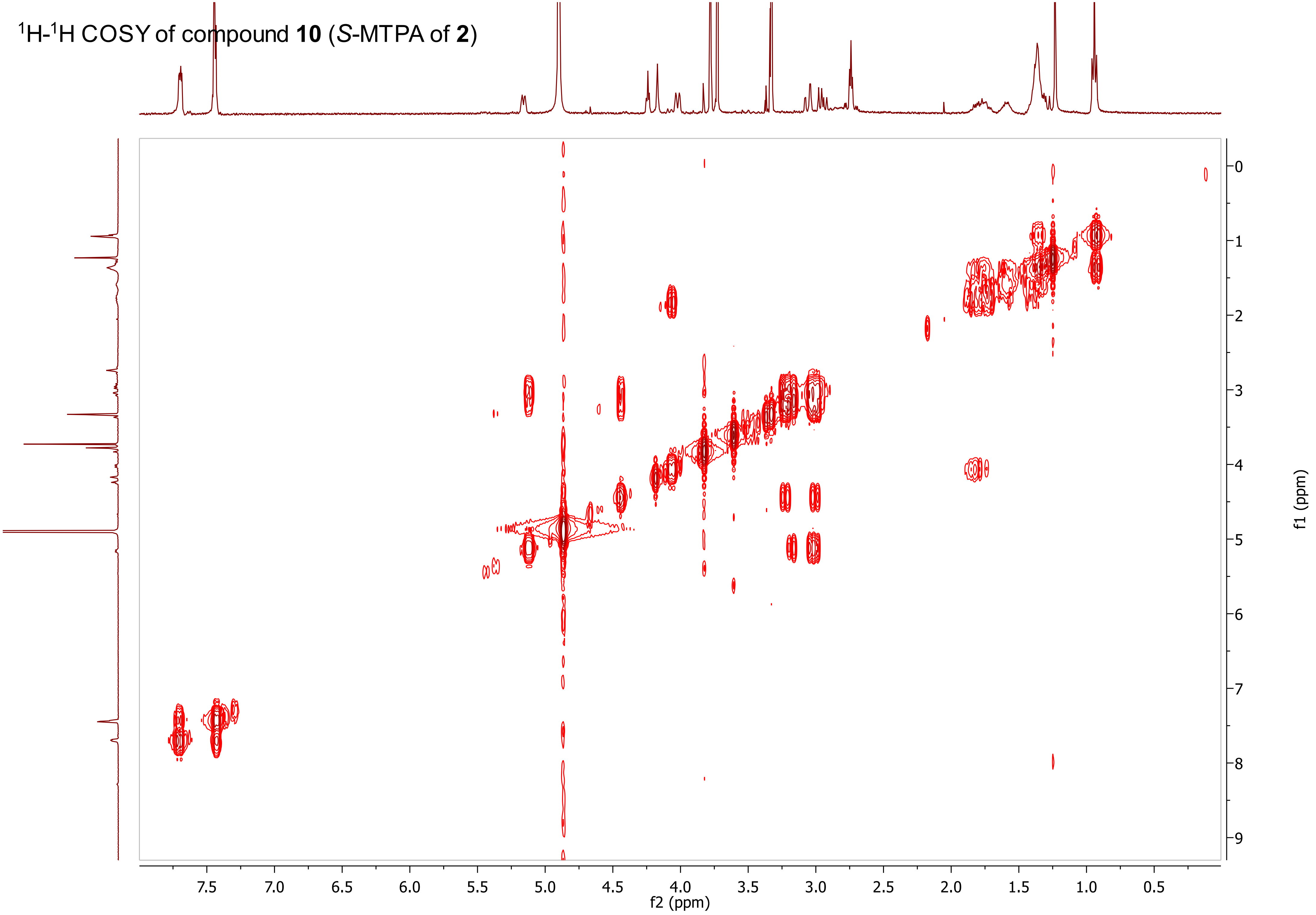

^{1}H NMR spectrum of compound **11** (400 MHz in methanol-d_4)

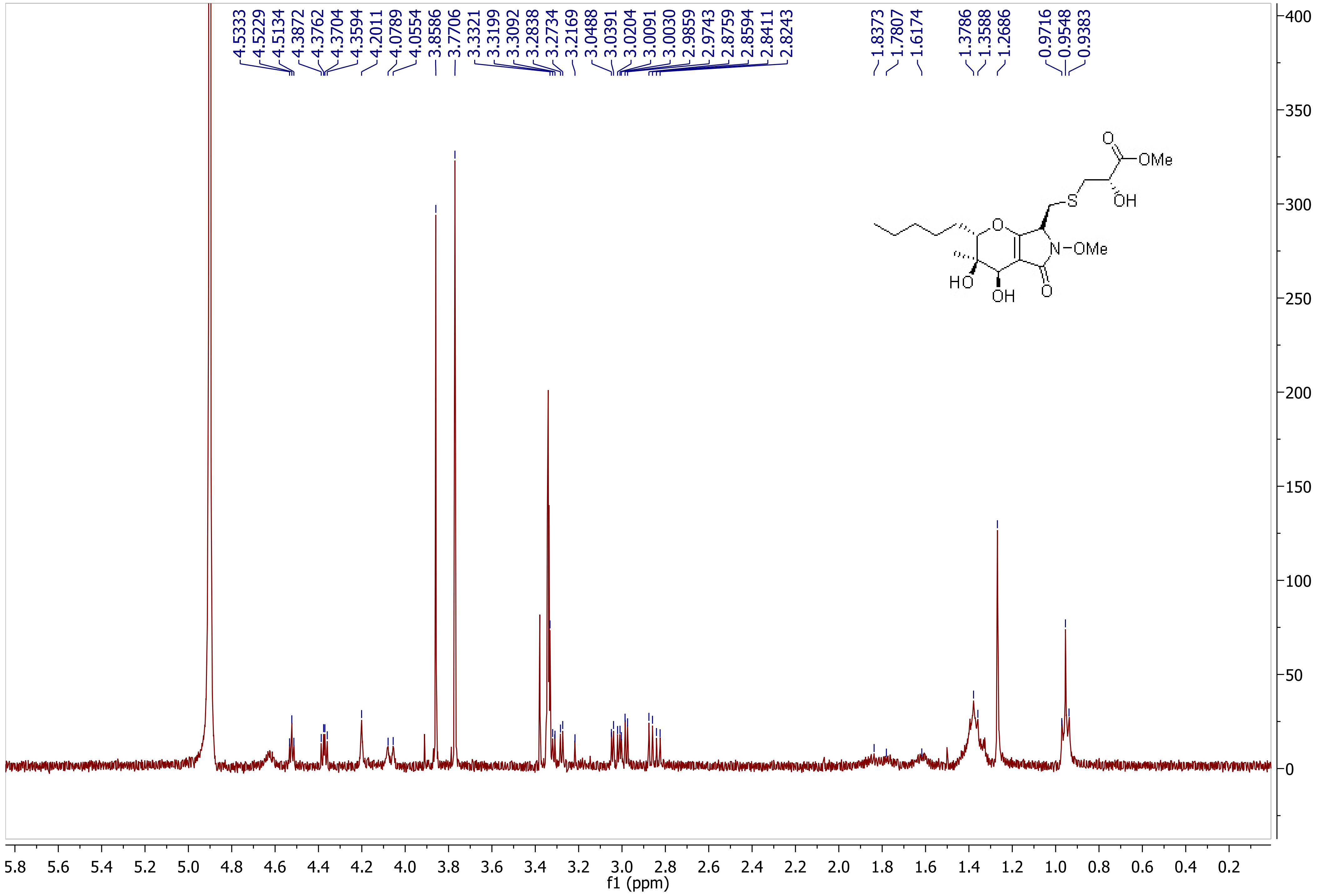

^{13}C NMR spectrum of compound **11**

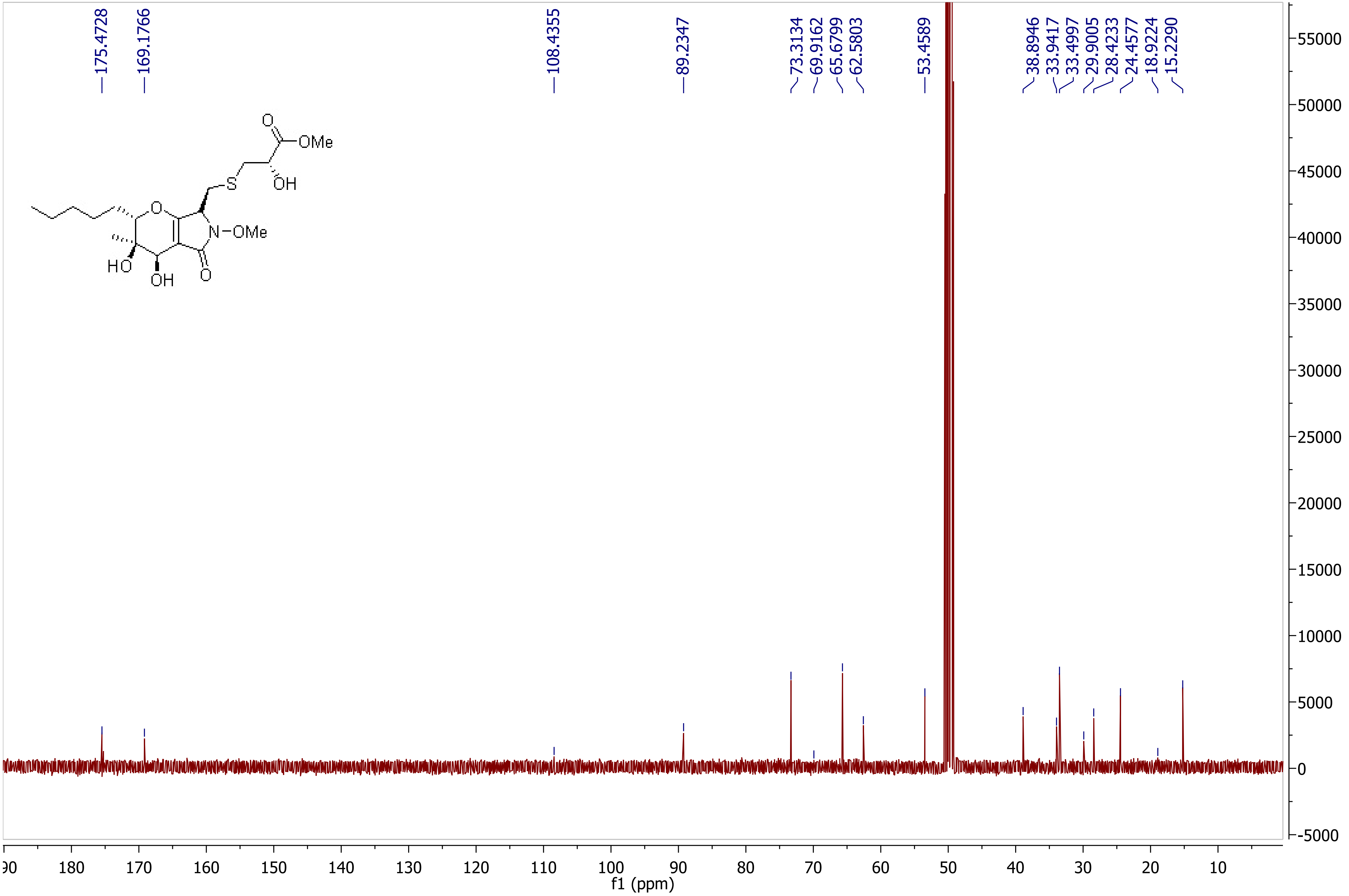

^1H-^1H COSY of compound **11**

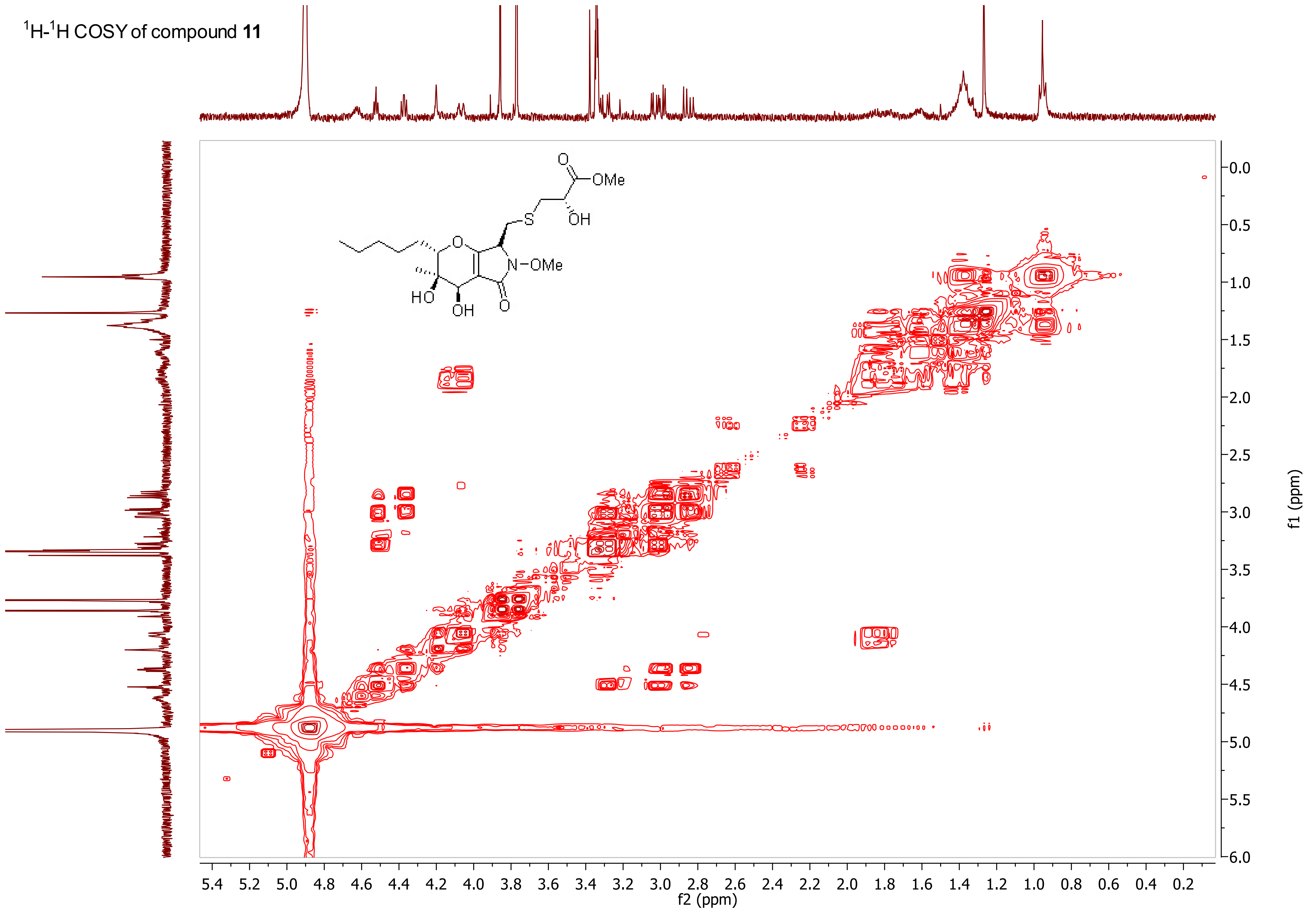

HSQC spectrum of compound **11**

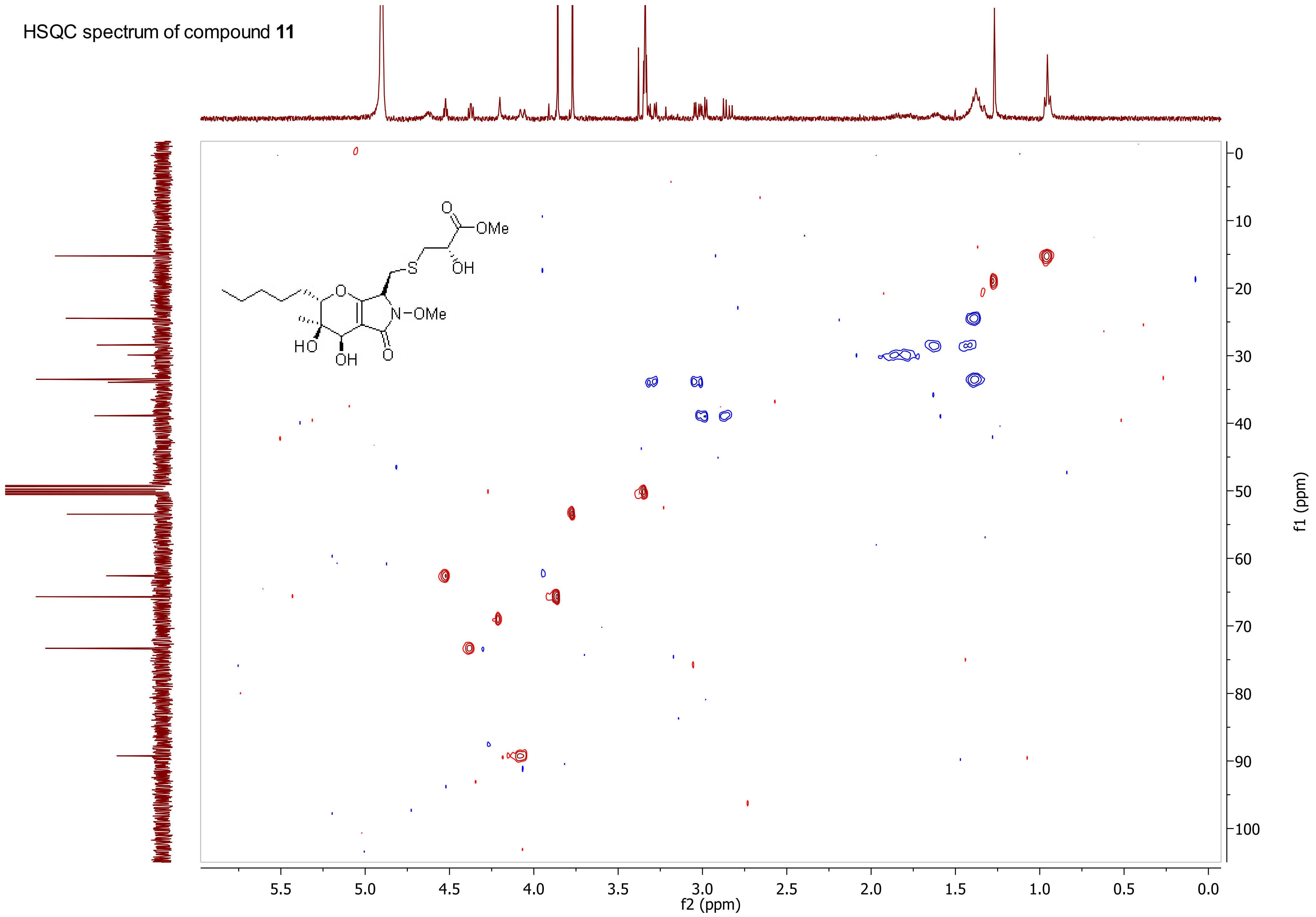

HMBC spectrum of compound **11**

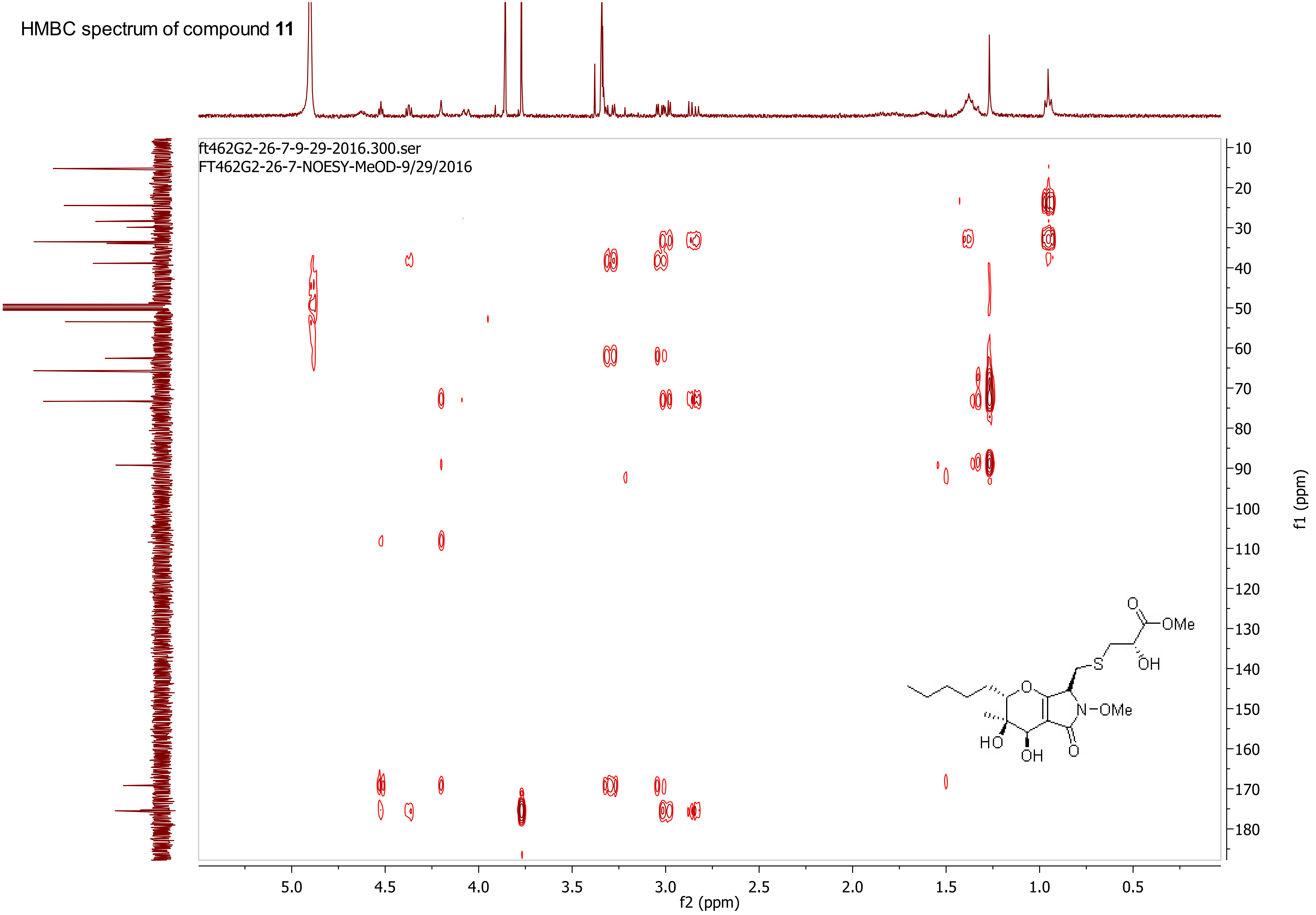

^1H NMR spectrum of **12** (*S*-MTPA of **11**) (400 MHz in methanol-d_4)

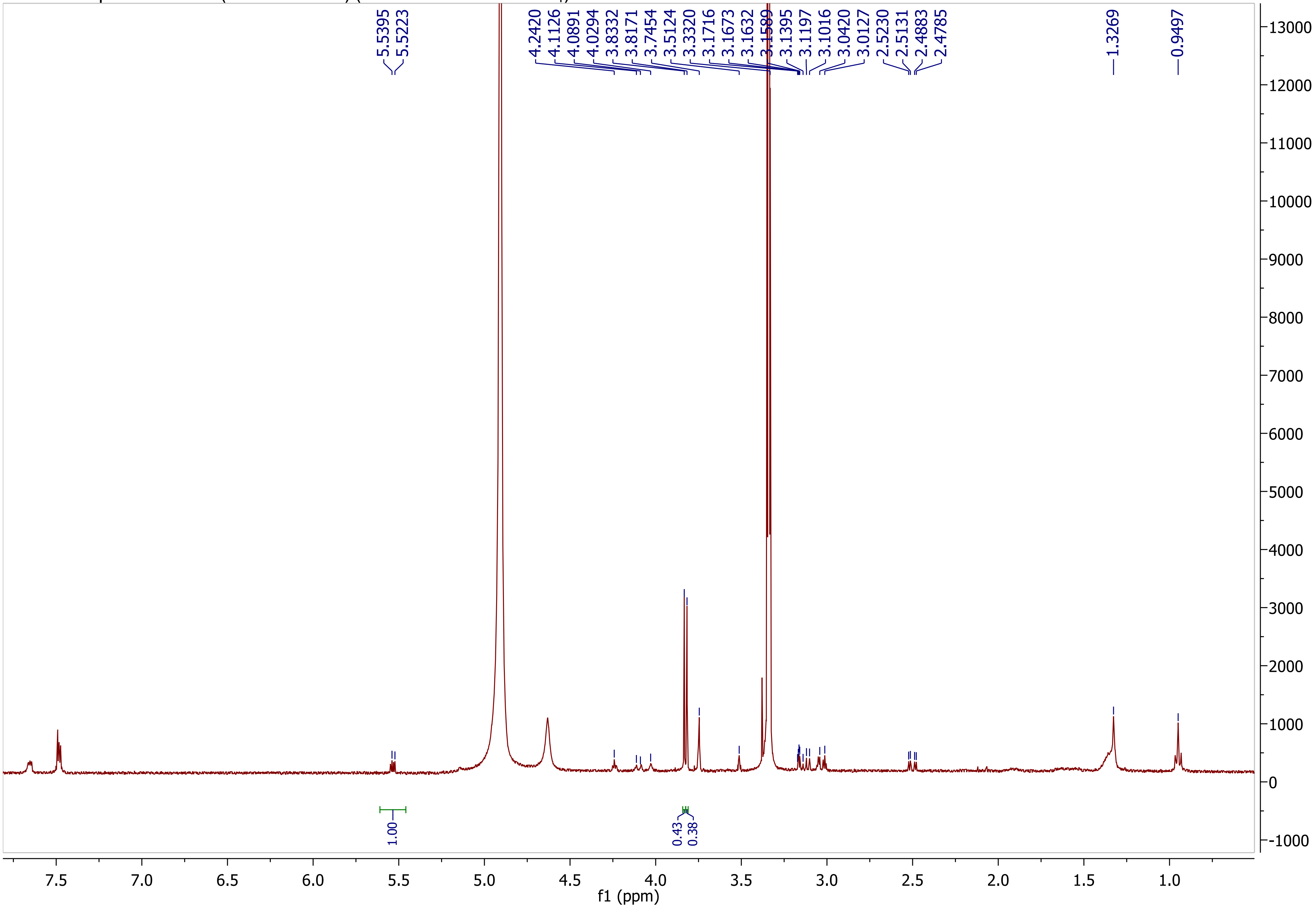

^{1}H NMR spectrum of **13** (R-MTPA of **11**) (400 MHz in methanol-d_4)

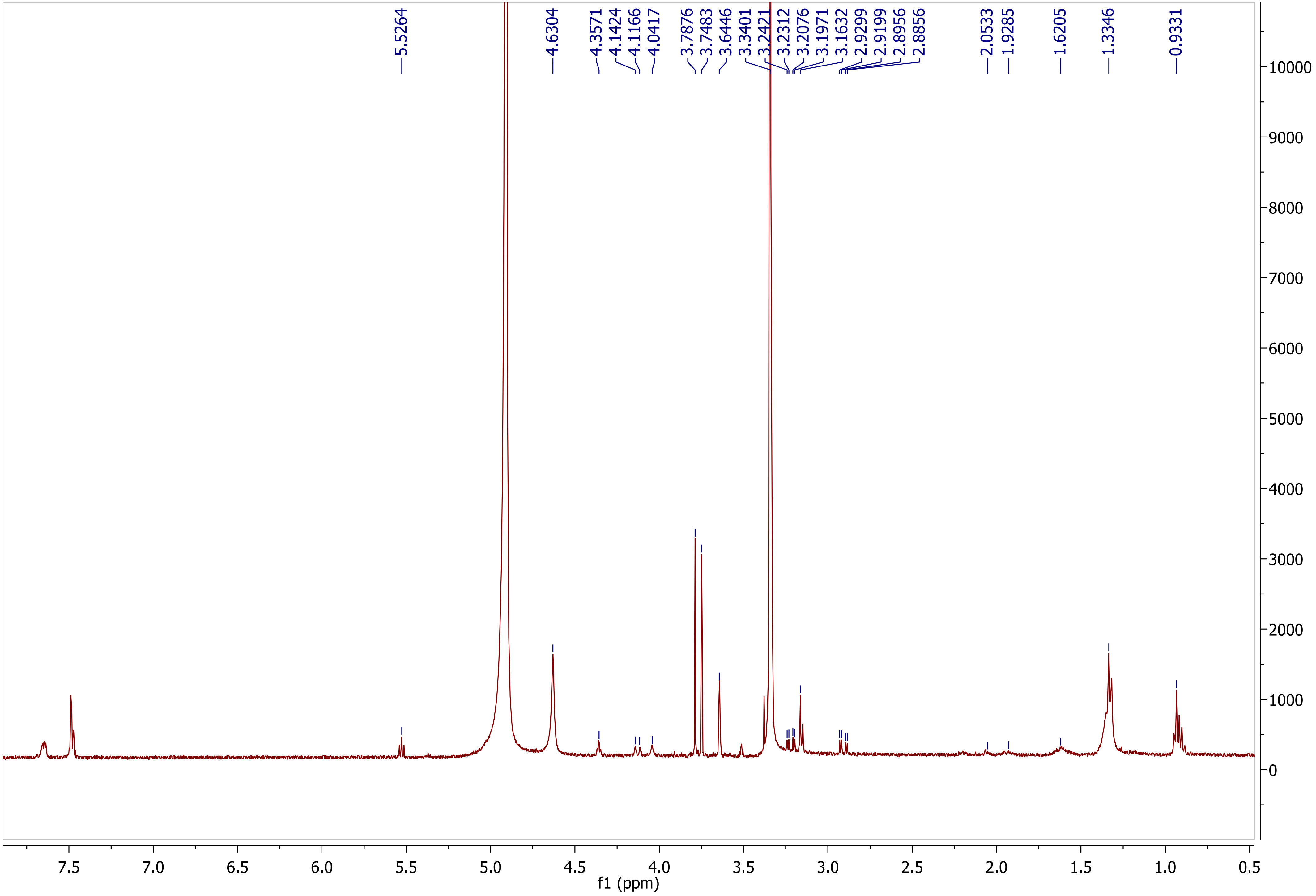

HRESIMS spectrum of compound **1**

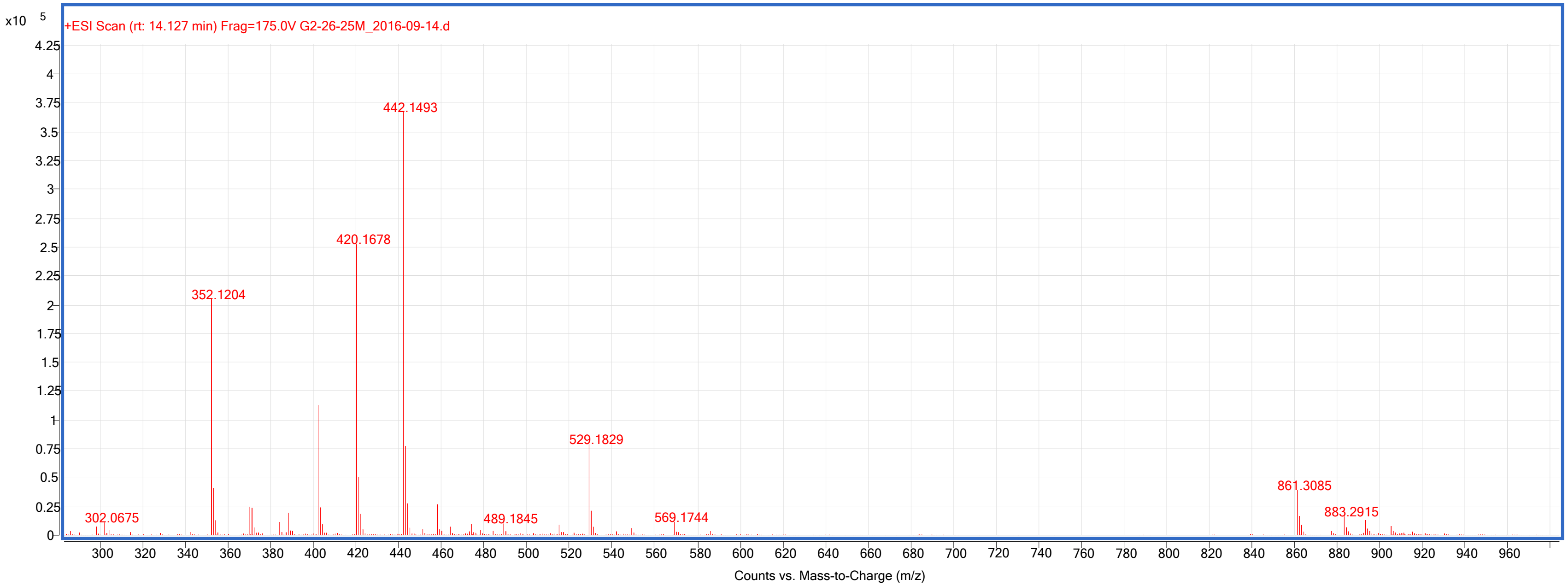

HRESIMS spectrum of compound **2**

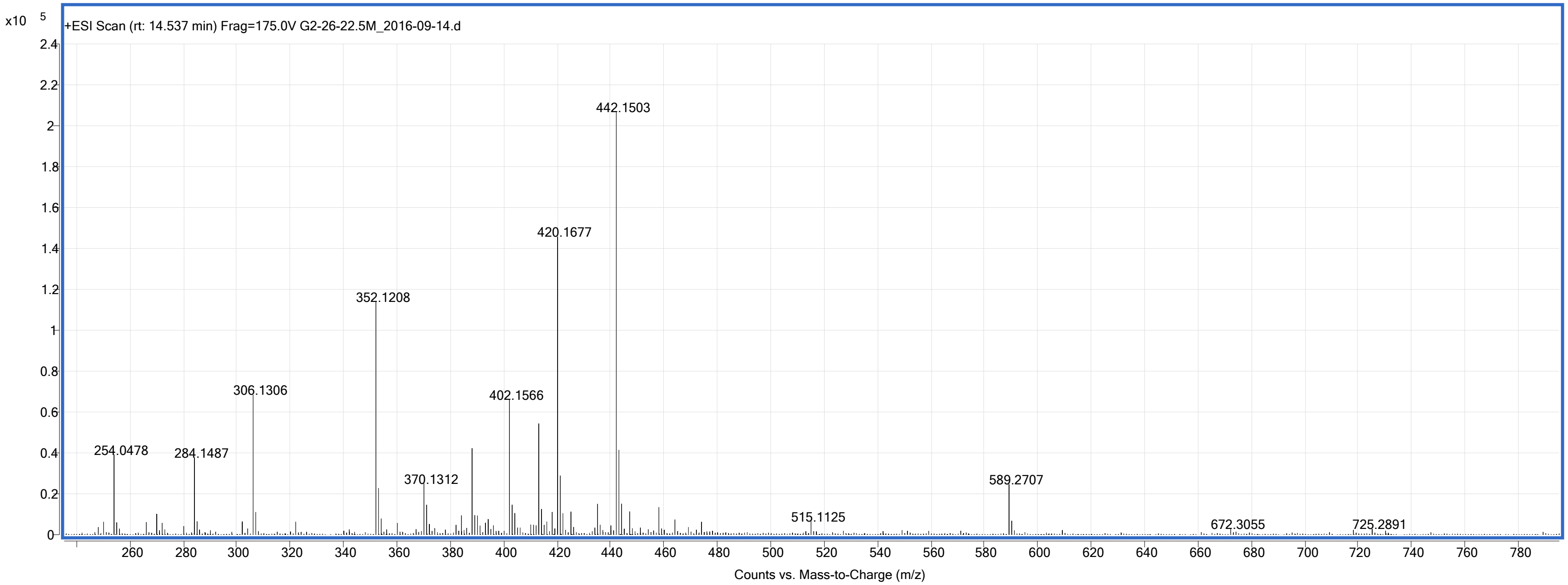

HRESIMS spectrum of compound **3**

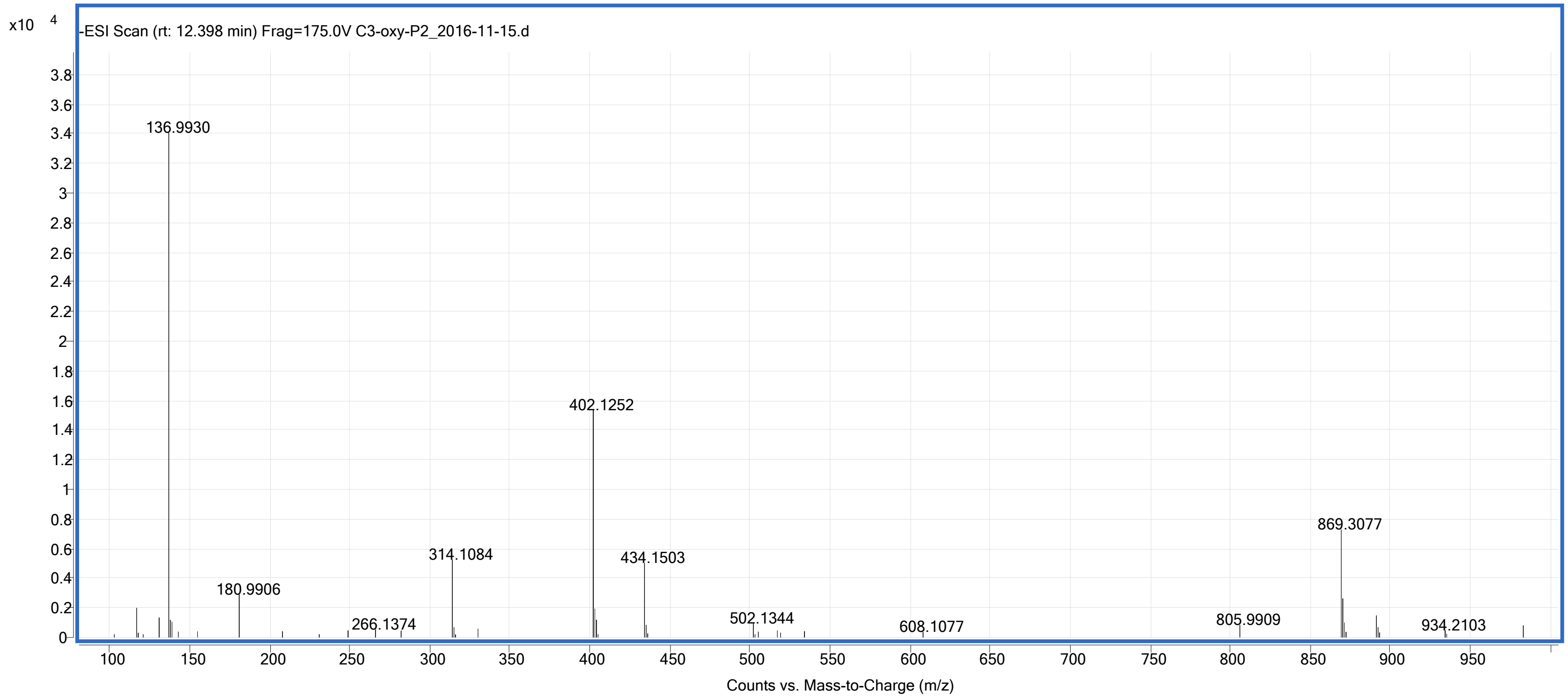

HRESIMS spectrum of compound **4**

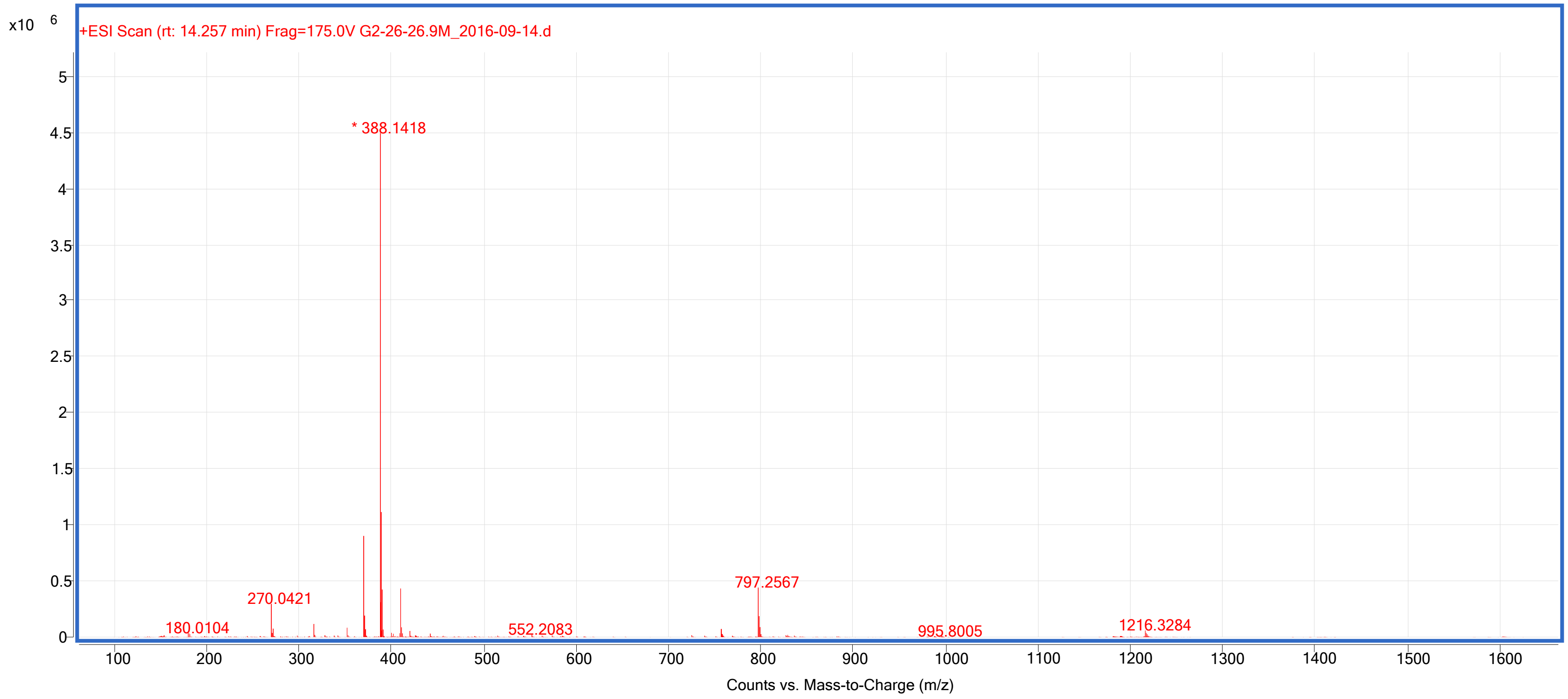

HRESIMS spectrum of compound **5**

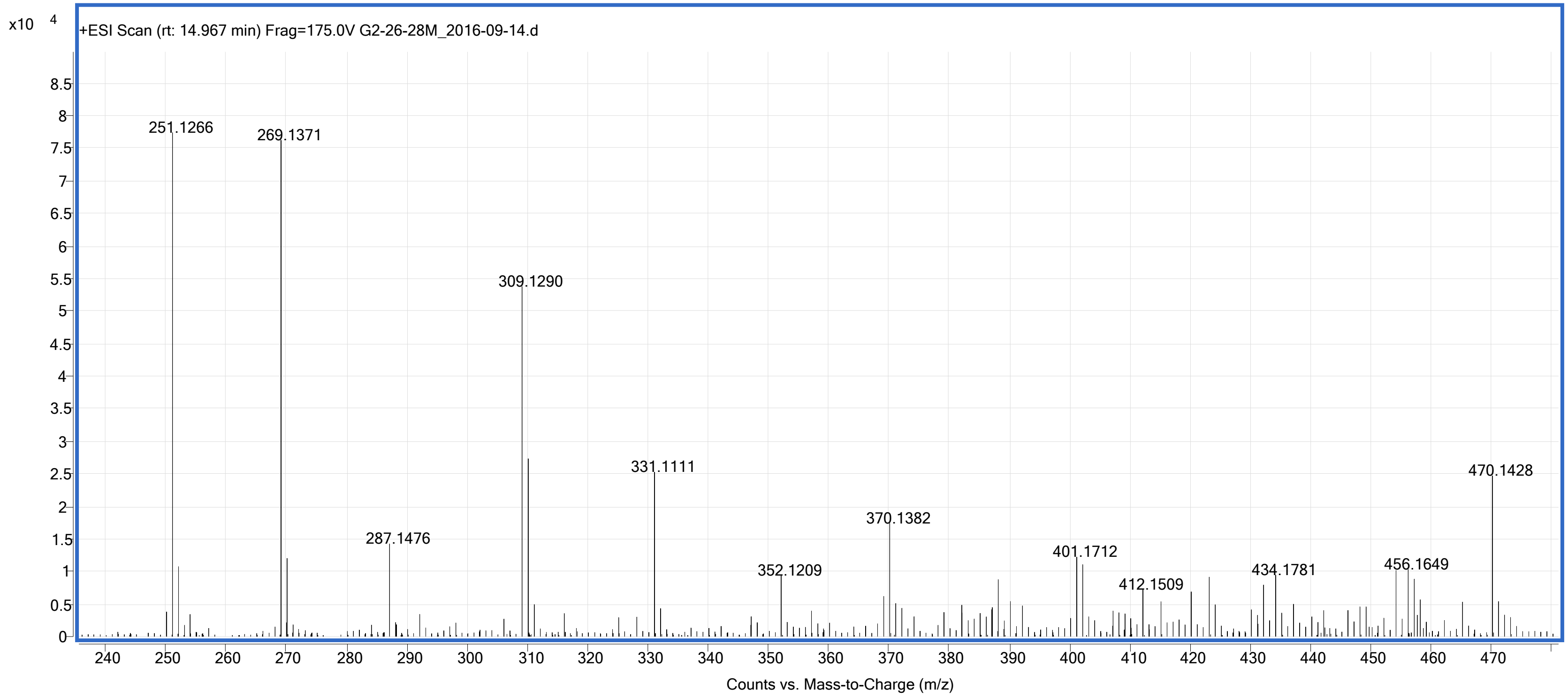

HRESIMS spectrum of compound **11**

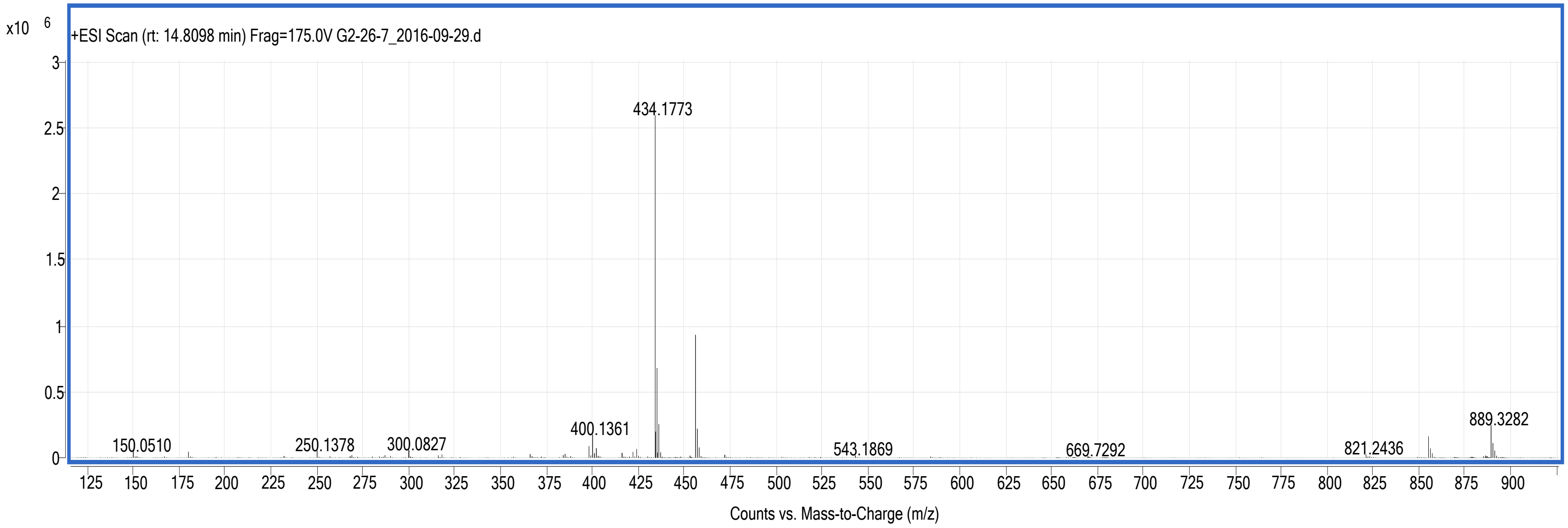

IR spectrum of compound **1**

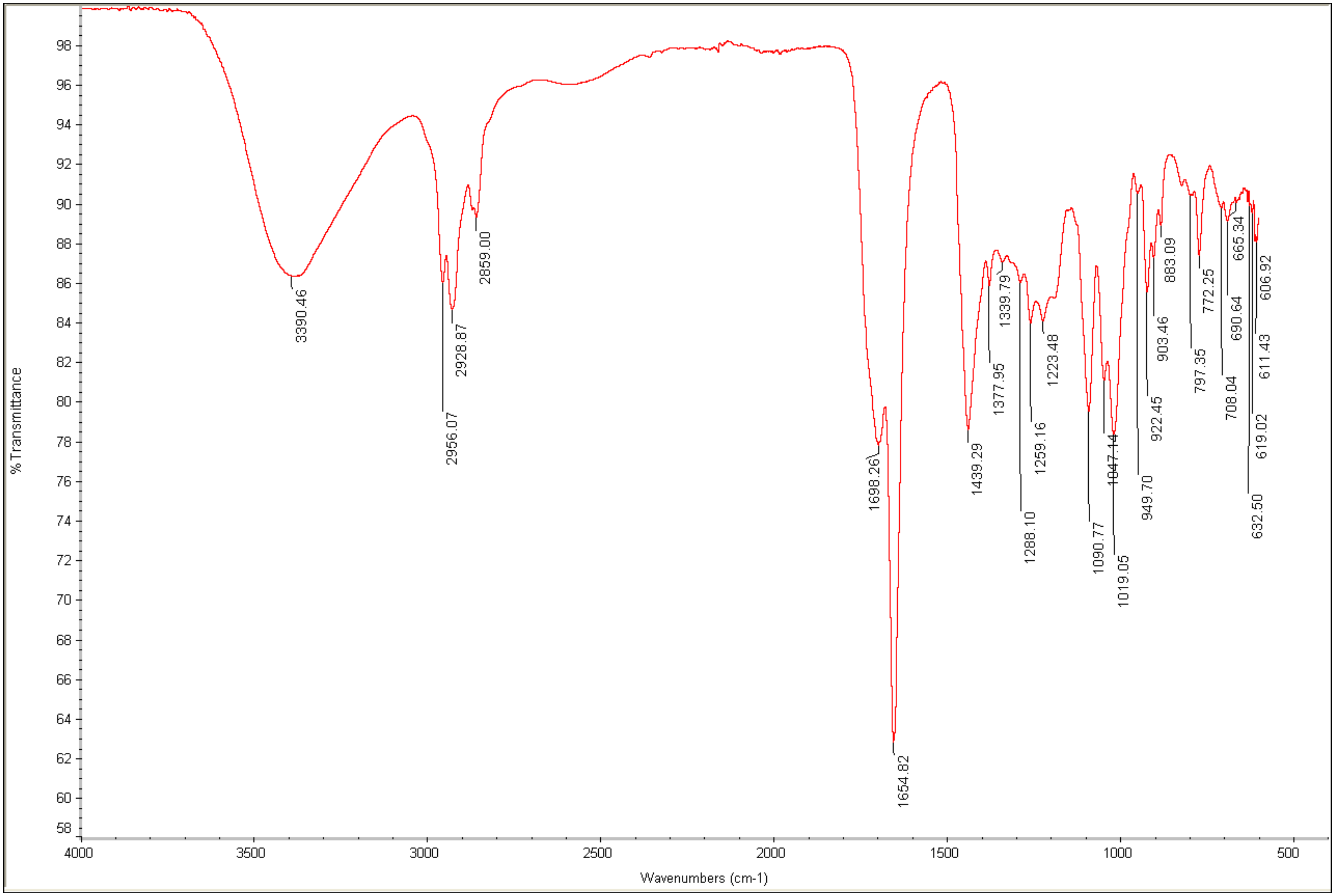

IR spectrum of compound **2**

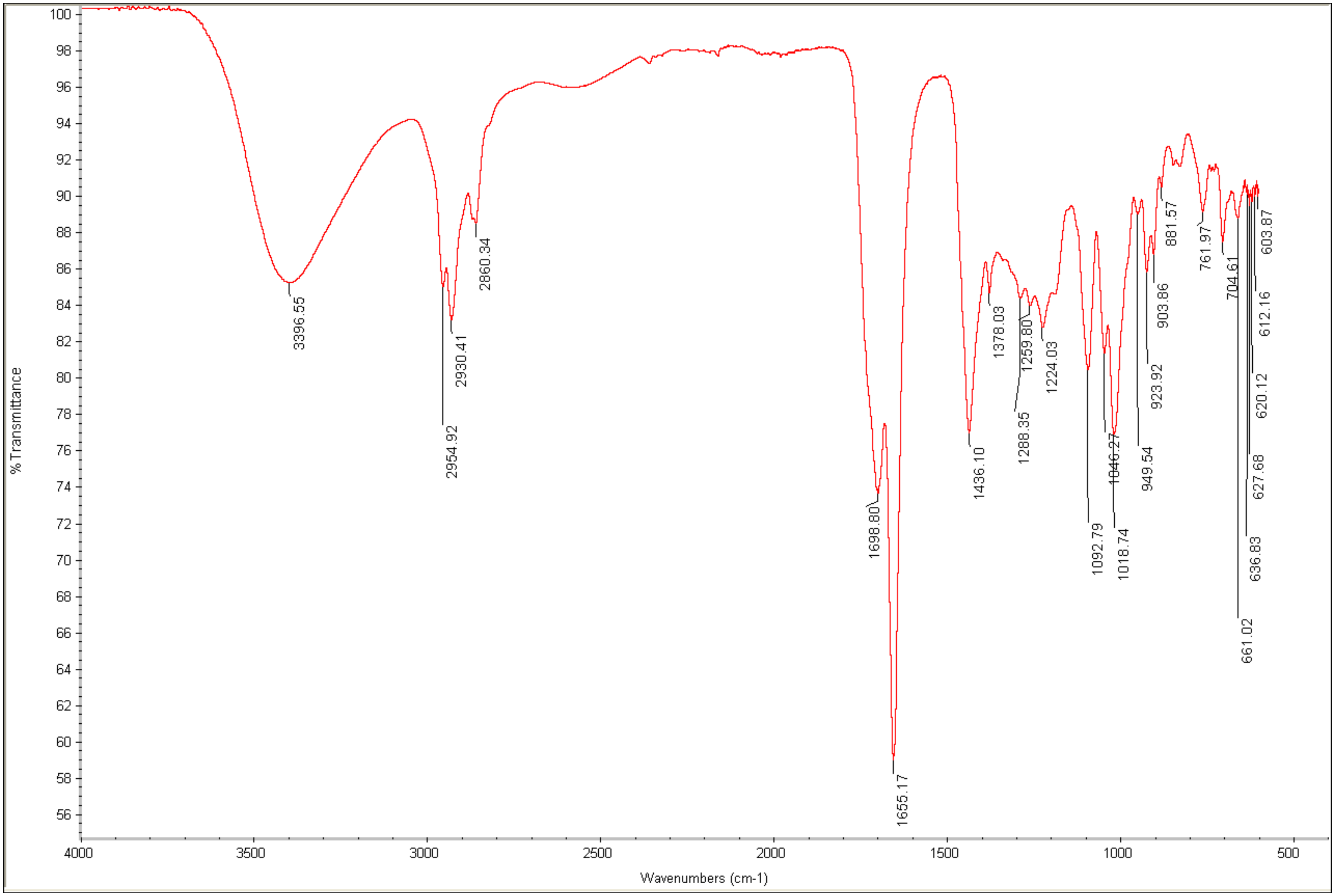

IR spectrum of compound **3**

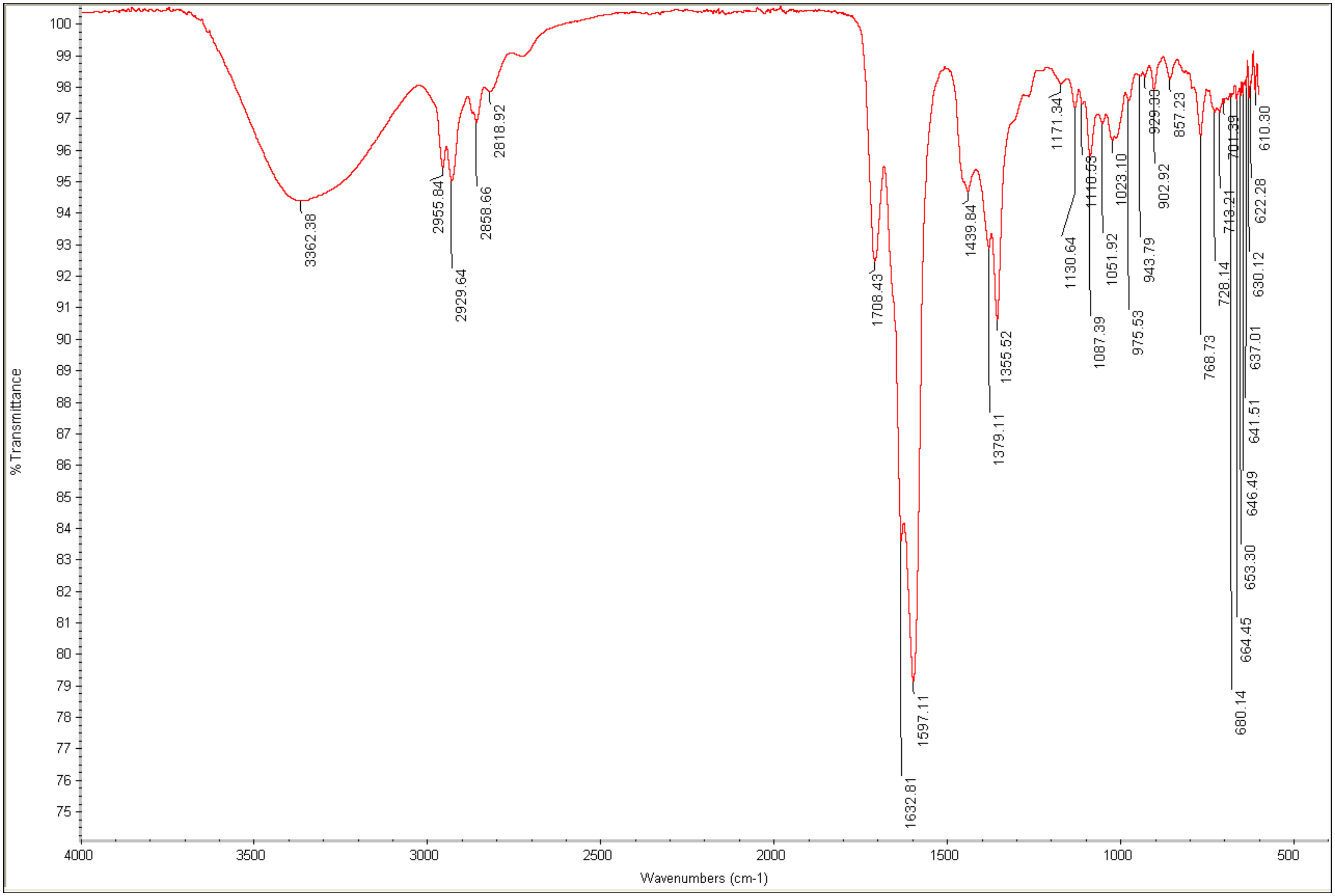

IR spectrum of compound **4**

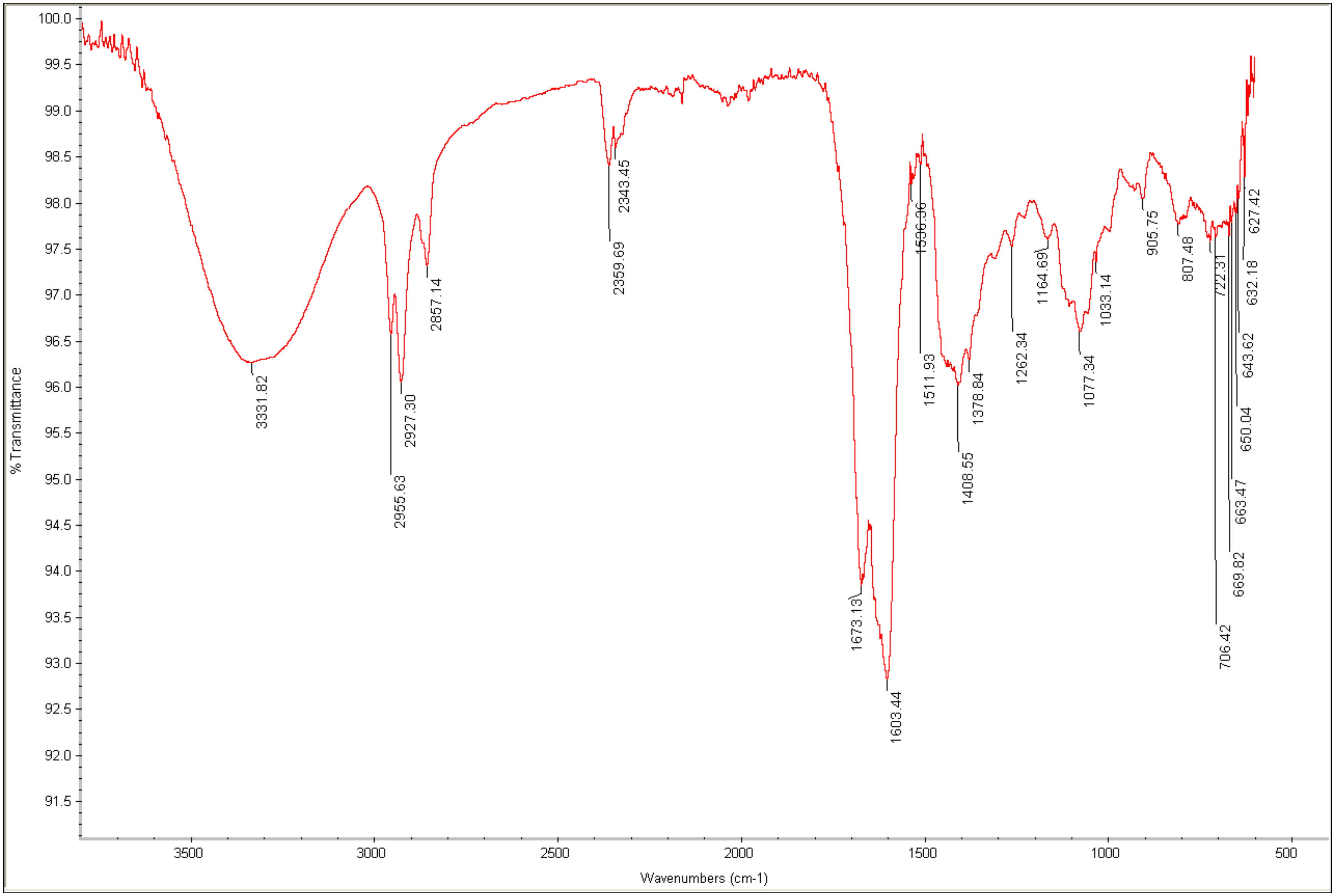

IR spectrum of compound **5**

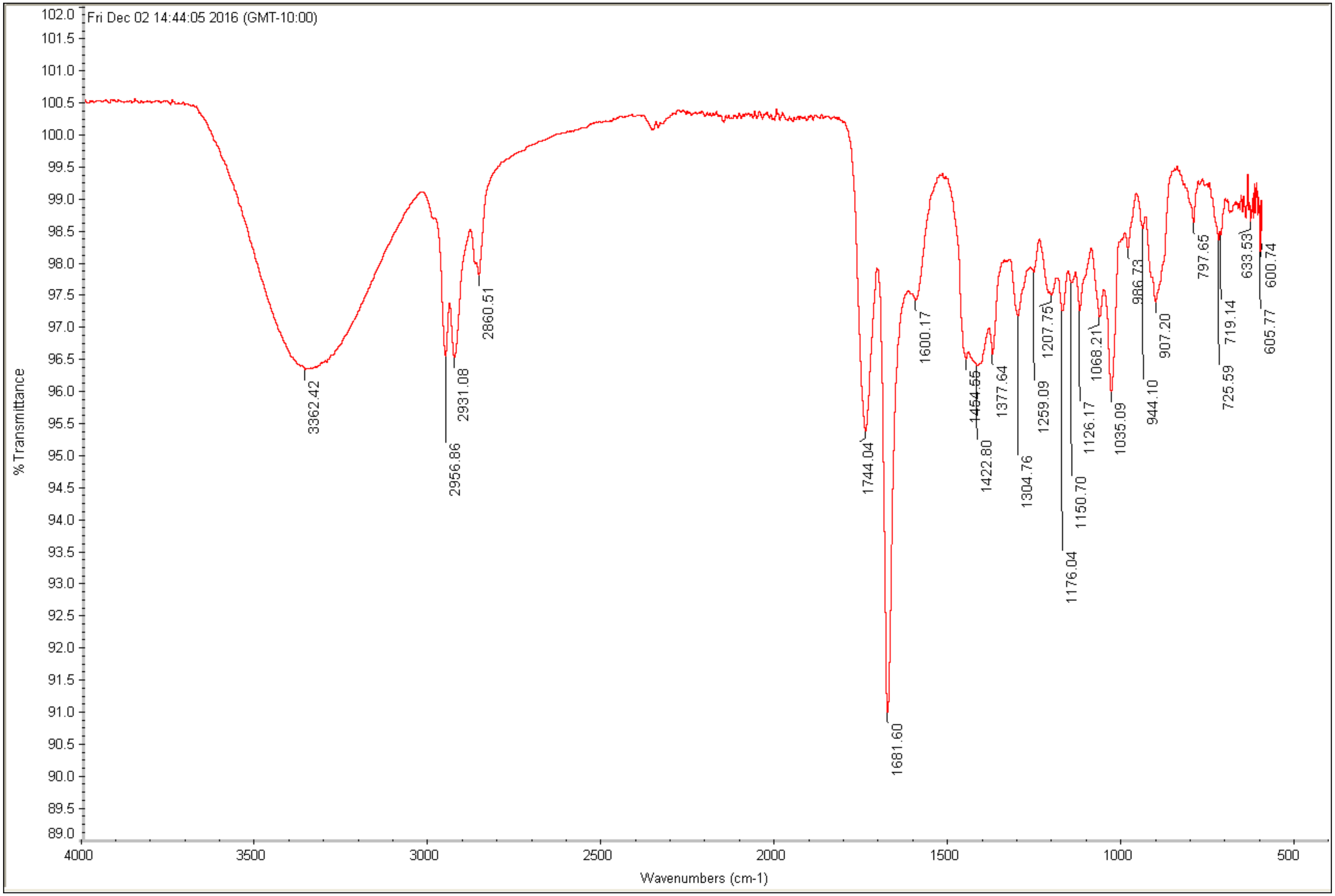

IR spectrum of compound **11**

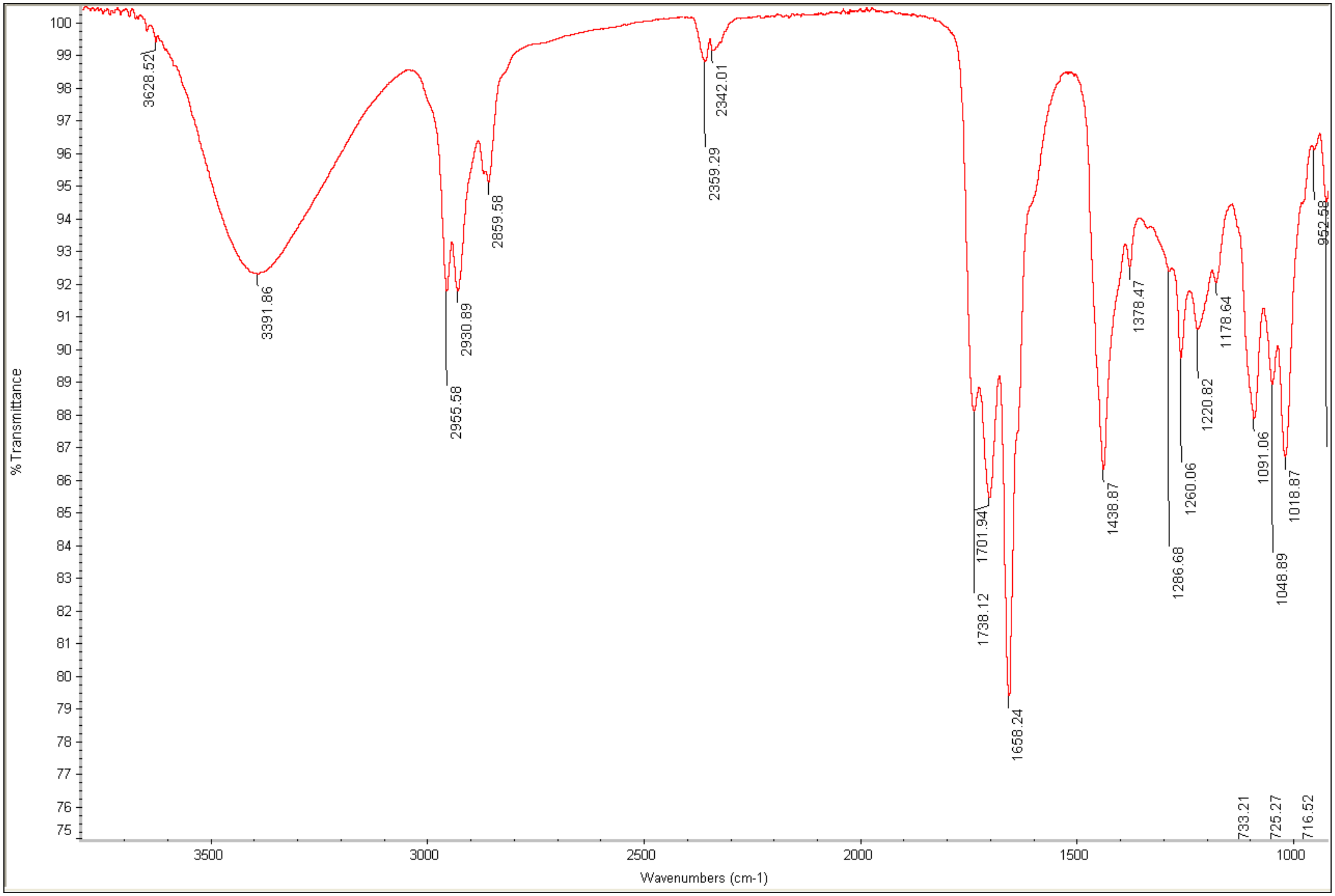

Table S1. NMR Boltzmann averaged isotropic magnetic shielding values (σ), unscaled (δ_u) and scaled (δ_s) chemical shifts calculated at the PCM/mPW1PW91/6-31+G**//PCM/B3LYP/6-31G* (solvent: MeOH) level of theory for all significantly populated conformers of **1-3*R*** and **1-3*S***.

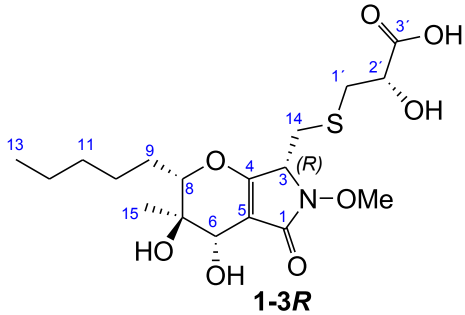

1-3*R*
DP4+ < 0.1%

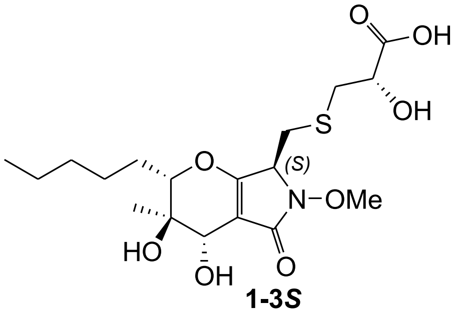

1-3*S*
DP4+ > 99.9%

Atom	δ_{exp}	σ		δ_u		Unscaled Error (abs[δ_{exp}-δ_u])		δ_s		Scaled Error (abs[δ_{exp}-δ_s])	
		**1-3*R***	**1-3*S***	**1-3*R***	**1-3*S***	**1-3*R***	**1-3*S***	**1-3*R***	**1-3*S***	**1-3*R***	**1-3*S***
C-1	175.7	23.8124	23.5001	173.0	173.3	2.7	2.4	175.1	175.8	0.6	0.1
C-2-OMe	65	131.1340	130.9314	65.6	65.8	0.6	0.8	64.2	64.1	0.8	0.9
C-3	62.3	131.9584	131.0416	64.8	65.7	2.5	3.4	63.4	64.0	1.1	1.7
C-4	168.6	29.7100	29.8394	167.1	166.9	1.5	1.7	169.0	169.2	0.4	0.6
C-5	106.6	90.6774	90.1192	106.1	106.7	0.5	0.1	106.0	106.5	0.6	0.1
C-6	66.6	123.1411	127.7651	73.6	69.0	7.0	2.4	72.5	67.4	5.9	0.8
C-7	72.8	122.7622	120.5333	74.0	76.2	1.2	3.4	72.9	74.9	0.1	2.1
C-8	88.7	108.7863	107.2456	88.0	89.5	0.7	0.8	87.3	88.7	1.4	0.0
C-9	29.5	166.0610	165.0125	30.7	31.8	1.2	2.3	28.2	28.7	1.3	0.8
C-10	28.1	165.9693	166.4093	30.8	30.4	2.7	2.3	28.2	27.2	0.1	0.9
C-11	32.7	161.3876	161.7262	35.4	35.1	2.7	2.4	33.0	32.1	0.3	0.6
C-12	23.7	169.4222	169.7023	27.4	27.1	3.7	3.4	24.7	23.8	1.0	0.1
C-13	14.4	180.1769	180.4343	16.6	16.3	2.2	1.9	13.6	12.6	0.8	1.8
C-14	33.6	158.0250	159.8961	38.8	36.9	5.2	3.3	36.5	34.0	2.9	0.4
C-15	20.0	181.0685	174.7048	15.7	22.1	4.3	2.1	12.7	18.6	7.3	1.4
C-1′	38.4	153.5293	152.4577	43.2	44.3	4.8	5.9	41.1	41.7	2.7	3.3
C-2′	72.5	123.5034	123.6006	73.3	73.2	0.8	0.7	72.1	71.7	0.4	0.8
C-3′	175.9	24.0218	25.2190	172.8	171.6	3.1	4.3	174.8	174.0	1.1	1.9
					MAE	***2.6***	***2.4***		***CMAE***	***1.6***	***1.0***
H-2-OMe	3.86	27.6149	27.6453	3.94	3.91	0.08	0.05	3.82	3.88	0.04	0.02
H-3	4.42	27.2243	27.3386	4.33	4.21	0.09	0.21	4.19	4.18	0.23	0.24
H-6	4.02	26.8670	27.3352	4.68	4.22	0.66	0.20	4.52	4.18	0.50	0.16
H-8	4.10	27.4749	27.2540	4.08	4.30	0.02	0.20	3.95	4.26	0.15	0.16
H-9a	1.93	29.6440	29.3996	1.91	2.15	0.02	0.22	1.91	2.16	0.02	0.23
H-9b	1.60	29.8971	30.0588	1.65	1.49	0.05	0.11	1.67	1.51	0.07	0.09
H-10a	1.60	29.8380	29.9079	1.71	1.64	0.11	0.04	1.73	1.66	0.13	0.06
H-10b	1.37	30.3328	30.3318	1.22	1.22	0.15	0.15	1.26	1.25	0.11	0.12
H-11	1.35	30.2660	30.3501	1.28	1.20	0.07	0.15	1.32	1.23	0.03	0.12
H-12	1.36	30.2109	30.2393	1.34	1.31	0.02	0.05	1.38	1.34	0.02	0.02
H-13	0.91	30.6189	30.6641	0.93	0.89	0.02	0.02	0.99	0.92	0.08	0.01
H-14	3.18	28.4127	28.3619	3.14	3.19	0.04	0.01	3.07	3.18	0.11	0.00
H-15	1.31	30.4426	30.1350	1.11	1.42	0.20	0.11	1.16	1.44	0.15	0.13
H-1′	2.94	28.5211	28.7722	3.03	2.78	0.09	0.16	2.97	2.78	0.03	0.16
H-2′	4.34	27.0490	27.1919	4.50	4.36	0.16	0.02	4.35	4.32	0.01	0.02
					MAE	***0.12***	***0.11***		***CMAE***	***0.11***	***0.10***

Table S2. NMR Boltzmann averaged isotropic magnetic shielding values (σ), unscaled (δ_u) and scaled (δ_s) chemical shifts calculated at the PCM/mPW1PW91/6-31+G**//PCM/B3LYP/6-31G* (solvent: MeOH) level of theory for all significantly populated conformers of **2-3*R*** and **2-3*S***.

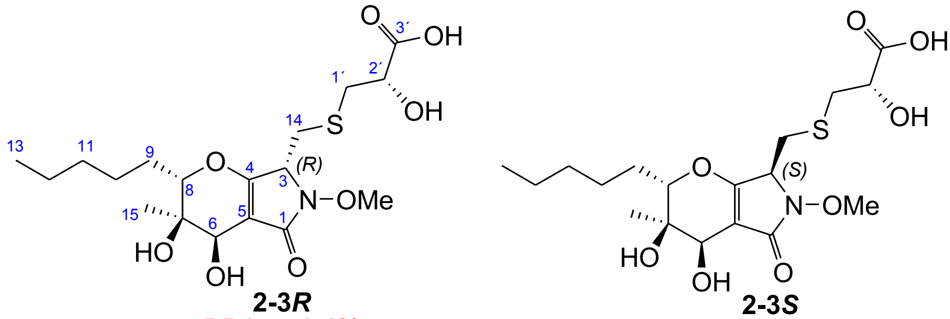

2-3*R*
DP4+ < 0.1%

2-3*S*
DP4+ > 99.9%

Atom	δ_{exp}	σ		δ_u		Unscaled Error (abs[δ_{exp}-δ_u])		δ_s		Scaled Error (abs[δ_{exp}-δ_s])	
		**2-3*R***	**2-3*S***	**2-3*R***	**2-3*S***	**2-3*R***	**2-3*S***	**2-3*R***	**2-3*S***	**2-3*R***	**2-3*S***
C-1	174.4	23.4424	25.2218	173.3	171.6	1.1	2.8	175.1	174.2	0.7	0.2
C-2-OMe	64.9	131.1884	131.4106	65.6	65.4	0.7	0.5	64.1	64.1	0.8	0.8
C-3	61.8	131.4890	132.5861	65.3	64.2	3.5	2.4	63.8	62.9	2.0	1.1
C-4	168.4	26.5264	29.2157	170.3	167.6	1.9	0.8	171.9	170.1	3.5	1.7
C-5	107.6	90.5787	91.1411	106.2	105.6	1.4	2.0	105.9	105.9	1.7	1.7
C-6	68.2	129.1848	129.9190	67.6	66.9	0.6	1.3	66.2	65.6	2.0	2.6
C-7	72.4	125.5052	123.0531	71.3	73.7	1.1	1.3	69.9	72.8	2.5	0.4
C-8	88.3	111.7136	109.3985	85.1	87.4	3.2	0.9	84.2	86.9	4.1	1.4
C-9	29.0	165.7895	164.8904	31.0	31.9	2.0	2.9	28.4	29.4	0.6	0.4
C-10	27.6	165.8398	166.2538	30.9	30.5	3.3	2.9	28.4	27.9	0.8	0.3
C-11	32.7	161.4149	161.6138	35.4	35.2	2.7	2.5	33.0	32.8	0.3	0.1
C-12	23.6	169.4214	169.6266	27.4	27.2	3.8	3.6	24.7	24.4	1.1	0.8
C-13	14.4	180.1837	180.4248	16.6	16.4	2.2	2.0	13.6	13.2	0.8	1.2
C-14	33.1	157.0026	160.6738	39.8	36.1	6.7	3.0	37.5	33.7	4.4	0.6
C-15	20.0	176.6961	174.8124	20.1	22.0	0.1	2.0	17.2	19.1	2.8	0.9
C-1′	38.3	152.9430	152.3963	43.8	44.4	5.5	6.1	41.7	42.3	3.4	4.0
C-2′	72.2	123.2263	124.5835	73.6	72.2	1.4	0.0	72.3	71.2	0.1	1.0
C-3′	175.9	23.6376	23.1214	173.1	173.7	2.8	2.2	174.9	176.4	1.0	0.5
					MAE	***2.4***	***2.2***		***CMAE***	***1.8***	***1.1***
H-2-OMe	3.82	27.6124	27.6871	3.94	3.86	0.12	0.04	3.88	3.80	0.06	0.02
H-3	4.48	27.2339	27.1905	4.32	4.36	0.16	0.12	4.24	4.27	0.24	0.21
H-6	4.17	27.3517	27.0902	4.20	4.46	0.03	0.29	4.13	4.37	0.04	0.20
H-8	4.04	27.4521	27.2938	4.10	4.26	0.06	0.22	4.03	4.17	0.01	0.13
H-9a	1.80	29.6499	29.9128	1.90	1.64	0.10	0.16	1.91	1.70	0.11	0.10
H-9b	1.73	29.9741	30.0609	1.58	1.49	0.15	0.24	1.60	1.56	0.13	0.17
H-10a	1.58	29.8575	30.0035	1.69	1.55	0.11	0.03	1.71	1.61	0.13	0.03
H-10b	1.37	30.2772	30.2671	1.27	1.28	0.10	0.09	1.30	1.36	0.07	0.01
H-11	1.33	30.2600	30.3340	1.29	1.22	0.04	0.11	1.32	1.30	0.01	0.03
H-12	1.34	30.1980	30.2482	1.35	1.30	0.01	0.04	1.38	1.38	0.04	0.04
H-13	0.91	30.6183	30.6525	0.93	0.90	0.02	0.01	0.98	1.00	0.07	0.09
H-14	3.15	28.4286	28.4065	3.12	3.14	0.03	0.01	3.09	3.12	0.06	0.03
H-15	1.23	30.5246	30.3499	1.03	1.20	0.20	0.03	1.07	1.28	0.16	0.05
H-1´	2.89	28.5625	28.5838	2.99	2.97	0.09	0.07	2.96	2.95	0.07	0.06
H-2′	4.29	26.9347	27.2095	4.62	4.34	0.33	0.05	4.53	4.25	0.24	0.04
					MAE	***0.10***	***0.10***		***CMAE***	***0.10***	***0.08***

Table S3. NMR Boltzmann averaged isotropic magnetic shielding values (σ), unscaled (δ_u) and scaled (δ_s) chemical shifts calculated at the PCM/mPW1PW91/6-31+G**//PCM/B3LYP/6-31G* (solvent: MeOH) level of theory for all significantly populated conformers of **3-S*R*** and **3-S*S***.

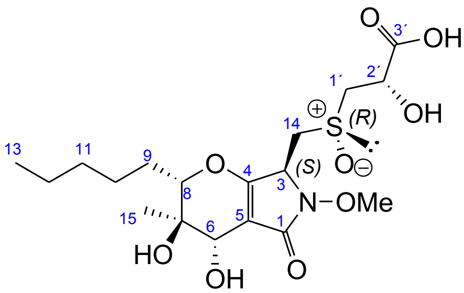

3-S*R*

DP4+ = 0.5%

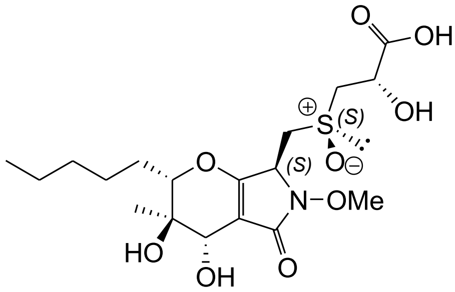

3-S*S*

DP4+ = 99.5%

Atom	δ_{exp}	σ		δ_u		Unscaled Error (abs[δ_{exp}-δ_u])		δ_s		Scaled Error (abs[δ_{exp}-δ_s])	
		**3-S*R***	**3-S*S***	**3-S*R***	**3-S*S***	**3-S*R***	**3-S*S***	**3-S*R***	**3-S*S***	**3-S*R***	**3-S*S***
C-2-OMe	64.8	131.2915	131.1198	65.5	65.7	0.7	0.9	64.2	63.9	0.6	0.9
C-3	58.6	136.1571	134.2567	60.6	62.5	2.0	3.9	59.2	60.7	0.6	2.1
C-4	169.3	30.9885	29.8370	165.8	166.9	3.5	2.4	167.2	167.7	2.1	1.6
C-5	105.6	89.8349	90.2849	106.9	106.5	1.3	0.9	106.8	105.8	1.2	0.2
C-6	66.3	124.1714	124.4510	72.6	72.3	6.3	6.0	71.5	70.7	5.2	4.4
C-7	72.4	121.8746	122.4500	74.9	74.3	2.5	1.9	73.9	72.8	1.5	0.4
C-8	89.1	107.3824	107.3040	89.4	89.5	0.3	0.4	88.8	88.3	0.3	0.8
C-9	29.2	166.0848	165.7240	30.7	31.1	1.5	1.9	28.5	28.4	0.7	0.8
C-10	27.7	166.2908	165.6435	30.5	31.1	2.8	3.4	28.3	28.5	0.6	0.8
C-11	32.3	161.3588	161.4001	35.4	35.4	3.1	3.1	33.3	32.9	1.0	0.6
C-12	23.4	169.4536	169.4640	27.3	27.3	3.9	3.9	25.0	24.6	1.6	1.2
C-13	14.2	180.2276	179.7549	16.6	17.0	2.4	2.8	13.9	14.0	0.3	0.2
C-14	52.1	140.4336	138.4176	56.3	58.4	4.2	6.3	54.8	56.4	2.7	4.3
C-15	19.8	179.6857	179.6991	17.1	17.1	2.7	2.7	14.5	14.1	5.3	5.7
C-1′	57.4	143.1502	142.8836	53.6	53.9	3.8	3.5	52.0	51.8	5.4	5.6
C-2′	66.9	128.4567	126.7194	68.3	70.1	1.4	3.2	67.1	68.4	0.2	1.5
					MAE	***2.7***	***2.9***		***CMAE***	***1.8***	***1.9***
H-2-OMe	3.90	27.5866	27.6096	3.96	3.94	0.06	0.04	3.93	3.92	0.03	0.02
H-3	4.61	27.1517	27.1953	4.40	4.36	0.21	0.25	4.36	4.32	0.25	0.29
H-6	4.00	26.9468	27.0765	4.60	4.47	0.60	0.47	4.56	4.44	0.56	0.44
H-8	4.15	27.4476	27.3809	4.10	4.17	0.05	0.02	4.07	4.14	0.08	0.01
H-9a	1.91	29.6700	29.7226	1.88	1.83	0.03	0.08	1.91	1.85	0.00	0.06
H-9b	1.64	29.7738	29.7779	1.78	1.77	0.14	0.13	1.81	1.80	0.17	0.16
H-10a	1.64	29.9627	29.8861	1.59	1.66	0.05	0.02	1.62	1.69	0.02	0.05
H-10b	1.40	30.2308	30.2888	1.32	1.26	0.08	0.14	1.36	1.30	0.04	0.10
H-11	1.34	30.2718	30.2872	1.28	1.26	0.06	0.08	1.32	1.30	0.02	0.04
H-12	1.35	30.2228	30.1691	1.33	1.38	0.02	0.03	1.37	1.41	0.02	0.06
H-13	0.92	30.6157	30.6351	0.93	0.92	0.01	0.00	0.99	0.96	0.07	0.04
H-14	3.54	28.3458	28.2194	3.20	3.33	0.34	0.21	3.20	3.32	0.34	0.22
H-15	1.32	30.3815	30.3785	1.17	1.17	0.15	0.15	1.22	1.21	0.10	0.11
H-1′	3.30	28.4383	28.2384	3.11	3.31	0.18	0.02	3.11	3.30	0.19	0.01
H-2′	4.40	26.9017	27.0323	4.65	4.52	0.25	0.12	4.60	4.48	0.20	0.08
					MAE	***0.15***	***0.12***		***CMAE***	***0.14***	***0.11***

Table S4. NMR Boltzmann averaged isotropic magnetic shielding values (σ), unscaled (δ_u) and scaled (δ_s) chemical shifts calculated at the PCM/mPW1PW91/6-31+G**//PCM/B3LYP/6-31G* (solvent: MeOH) level of theory for all significantly populated conformers of **4-3*E*** and **4-3*Z***.

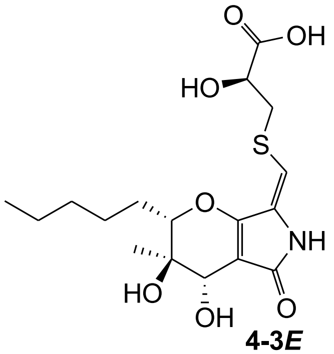

4-3*E*
DP4+ < 0.1%

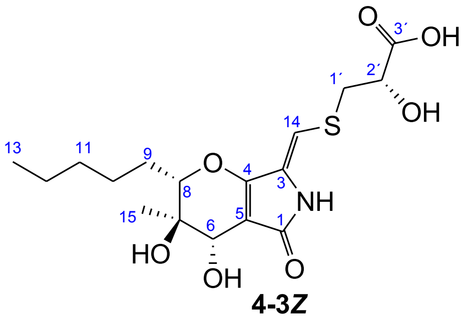

4-3*Z*
DP4+ > 99.9%

Atom	δ_{exp}	σ		δ_u		Unscaled Error (abs[δ_{exp}-δ_u])		δ_s		Scaled Error (abs[δ_{exp}-δ_s])	
		**4-3*E***	**4-3*Z***	**4-3*E***	**4-3*Z***	**4-3*E***	**4-3*Z***	**4-3*E***	**4-3*Z***	**4-3*E***	**4-3*Z***
C-1	175.0	32.1868	30.9502	164.6	165.8	10.4	9.2	167.7	169.4	7.3	5.6
C-3	130.2	72.8896	69.1313	123.9	127.6	6.3	2.6	125.0	129.3	5.2	0.9
C-4	159.5	39.9567	40.6332	156.8	156.1	2.7	3.4	159.5	159.2	0.0	0.3
C-5	105.7	91.1701	93.1350	105.6	103.6	0.1	2.1	105.8	104.1	0.1	1.6
C-6	67.5	123.0434	122.7562	73.7	74.0	6.2	6.5	72.4	73.1	4.9	5.6
C-7	73.0	122.2488	122.2200	74.5	74.6	1.5	1.6	73.2	73.6	0.2	0.6
C-8	87.6	108.7573	109.3215	88.0	87.5	0.4	0.1	87.4	87.1	0.2	0.5
C-9	29.1	165.8330	165.8193	30.9	31.0	1.8	1.9	27.5	27.9	1.6	1.2
C-10	27.7	165.7511	165.8652	31.0	30.9	3.3	3.2	27.6	27.8	0.1	0.1
C-11	32.5	161.2187	161.3375	35.6	35.4	3.1	2.9	32.4	32.6	0.1	0.1
C-12	23.4	169.3530	169.4581	27.4	27.3	4.0	3.9	23.8	24.1	0.4	0.7
C-13	13.9	180.2878	179.9037	16.5	16.9	2.6	3.0	12.4	13.1	1.5	0.8
C-14	110.5	73.1882	80.5010	123.6	116.3	13.1	5.8	124.7	117.4	14.2	6.9
C-15	18.8	180.8295	181.1981	15.9	15.6	2.9	3.2	11.8	11.8	7.0	7.0
C-1´	39.8	151.8481	151.4867	44.9	45.3	5.1	5.5	42.2	42.9	2.4	3.1
C-2´	72.2	123.4194	124.0875	73.4	72.7	1.2	0.5	72.0	71.7	0.2	0.5
C-3´	175.5	23.8207	23.7082	173.0	173.1	4.0	3.5	176.4	176.9	2.8	2.2
					MAE	***4.1***	***3.4***		***CMAE***	***2.9***	***2.2***
H-6	4.13	26.7868	26.7830	4.76	4.77	0.63	0.64	4.43	4.48	0.30	0.35
H-8	4.03	27.4563	27.5248	4.09	4.03	0.06	0.00	3.83	3.81	0.20	0.22
H-9a	1.86	29.6270	29.6552	1.92	1.90	0.06	0.04	1.87	1.87	0.01	0.01
H-9b	1.66	29.7952	29.8795	1.76	1.67	0.10	0.01	1.72	1.67	0.06	0.01
H-10	1.48	30.0119	30.0757	1.54	1.47	0.06	0.01	1.52	1.49	0.04	0.01
H-11	1.34	30.2474	30.2719	1.30	1.28	0.04	0.06	1.31	1.31	0.03	0.03
H-12	1.34	30.1970	30.2062	1.35	1.34	0.01	0.00	1.36	1.37	0.02	0.03
H-13	0.91	30.6071	30.6329	0.94	0.92	0.03	0.01	0.99	0.98	0.08	0.07
H-14	6.31	24.7342	24.8516	6.82	6.70	0.51	0.39	6.29	6.24	0.02	0.07
H-15	1.28	30.4140	30.4476	1.14	1.10	0.14	0.18	1.16	1.15	0.12	0.13
H-1´	3.19	28.3553	28.3076	3.20	3.24	0.01	0.05	3.02	3.10	0.17	0.09
H-2´	4.29	26.9019	26.9277	4.65	4.62	0.36	0.33	4.33	4.35	0.04	0.06
					MAE	***0.17***	***0.14***		***CMAE***	***0.09***	***0.09***

Table S5. NMR Boltzmann averaged isotropic magnetic shielding values (σ), unscaled (δ_u) and scaled (δ_s) chemical shifts calculated at the PCM/mPW1PW91/6-31+G**//PCM/B3LYP/6-31G* (solvent: MeOH) level of theory for all significantly populated conformers of **5-3*R*** and **5-3*S***.

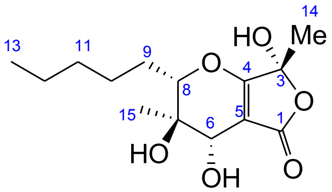

5-3*R*
DP4+ = 92.2%

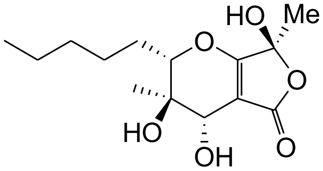

5-3*S*
DP4+ = 7.8%

Atom	δ_{exp}	σ		δ_u		Unscaled Error (abs[δ_{exp}-δ_u])		δ_s		Scaled Error (abs[δ_{exp}-δ_s])	
		**5-3*R***	**5-3*S***	**5-3*R***	**5-3*S***	**5-3*R***	**5-3*S***	**5-3*R***	**5-3*S***	**5-3*R***	**5-3*S***
C-1	174.7	30.1186	30.1382	166.7	166.6	8.0	8.1	169.6	169.6	5.1	5.1
C-3	102.5	92.8282	92.5063	104.0	104.3	1.5	1.8	104.3	104.7	1.8	2.2
C-4	174.7	24.3932	24.3599	172.4	172.4	2.3	2.3	175.5	175.6	0.8	0.9
C-5	102.0	93.1896	93.7808	103.6	103.0	1.6	1.0	104.0	103.3	2.0	1.3
C-6	66.5	125.0119	124.6208	71.8	72.2	5.3	5.7	70.9	71.2	4.4	4.7
C-7	72.4	122.5304	122.6374	74.2	74.1	1.8	1.7	73.4	73.3	1.0	0.9
C-8	88.6	106.8554	106.7124	89.9	90.1	1.3	1.5	89.7	89.9	1.1	1.3
C-9	28.9	165.9589	165.8647	30.8	30.9	1.9	2.0	28.2	28.3	0.7	0.6
C-10	27.2	166.2754	166.1219	30.5	30.7	3.3	3.5	27.9	28.0	0.7	0.8
C-11	32.1	161.5856	161.5418	35.2	35.2	3.1	3.1	32.8	32.8	0.7	0.7
C-12	23.1	169.7554	169.5599	27.0	27.2	3.9	4.1	24.3	24.4	1.2	1.3
C-13	13.9	180.3135	180.4119	16.5	16.4	2.6	2.5	13.3	13.1	0.6	0.8
C-14	22.9	173.3273	173.0114	23.5	23.8	0.6	0.9	20.6	20.8	2.3	2.1
C-15	19.4	179.3773	179.6340	17.4	17.1	2.0	2.3	14.3	13.9	5.1	5.5
					MAE	***2.8***	***2.9***		***CMAE***	***2.0***	***2.0***
H-6	4.05	27.0075	26.9616	4.54	4.59	0.49	0.54	4.27	4.30	0.22	0.25
H-8	4.17	27.3531	27.3594	4.20	4.19	0.03	0.02	3.96	3.94	0.21	0.23
H-9a	1.90	29.6428	29.6909	1.91	1.86	0.01	0.04	1.87	1.82	0.03	0.08
H-9b	1.66	29.7881	29.7174	1.76	1.83	0.10	0.17	1.74	1.80	0.08	0.14
H-10	1.49	30.0454	30.0537	1.51	1.50	0.02	0.01	1.50	1.50	0.01	0.01
H-11	1.34	30.2572	30.2499	1.29	1.30	0.05	0.04	1.31	1.32	0.03	0.02
H-12	1.35	30.2291	30.2298	1.32	1.32	0.03	0.03	1.33	1.34	0.02	0.01
H-13	0.92	30.6186	30.6102	0.93	0.94	0.01	0.02	0.98	0.99	0.06	0.07
H-14	1.64	29.8662	29.8849	1.68	1.67	0.04	0.03	1.67	1.65	0.03	0.01
H-15	1.32	30.3666	30.3883	1.18	1.16	0.14	0.16	1.21	1.19	0.11	0.13
					MAE	***0.09***	***0.11***		***CMAE***	***0.08***	***0.09***

Cartesian Coordinates of all significantly populated conformers found for all compounds under study at the PCM/B3LYP/6-31G* level of theory.

1-3*R*-c1
B3LYP/6-31G* geometry
O 0 0.961248 -0.880736 -0.855789
C 0 0.347093 -3.162214 -0.502072
C 0 2.121991 -2.223141 0.921462
C 0 1.540049 -3.531706 0.322362
C 0 2.262483 -1.191368 -0.237284
C 0 0.155939 -1.927914 -1.006804
C 0 -1.143032 -1.826488 -1.769604
N 0 -1.768790 -3.108391 -1.393606
C 0 -0.804842 -3.984948 -0.837190
O 0 -2.590682 -3.659018 -2.393525
C 0 -3.900889 -3.922616 -1.869947
C 0 -2.005200 -0.597910 -1.470805
S 0 -2.566138 -0.576624 0.286428
C 0 -2.820695 1.220788 0.543480
C 0 -4.043485 1.776454 -0.212822
C 0 -4.275600 3.228156 0.223339
O 0 -5.232995 1.050613 0.061799
O 0 -3.510738 4.127046 -0.052761
O 0 -5.372783 3.418910 0.967388
O 0 -0.974921 -5.185618 -0.644578
O 0 1.219872 -4.440962 1.371609
O 0 3.468258 -2.460182 1.345511
C 0 1.276175 -1.701226 2.088533
C 0 2.887368 0.141264 0.152862
C 0 3.243221 1.025501 -1.051113
C 0 3.885778 2.355421 -0.638216
C 0 4.260533 3.246511 -1.829589
C 0 4.902376 4.573097 -1.410465
H 0 2.327511 -3.969381 -0.311380
H 0 2.861932 -1.664248 -1.025641
H 0 -0.936784 -1.834041 -2.849405
H 0 -4.469288 -4.308473 -2.719542
H 0 -4.367301 -3.006651 -1.492183
H 0 -3.857937 -4.675722 -1.077546
H 0 -2.867504 -0.594010 -2.141503
H 0 -1.412559 0.301833 -1.661196
H 0 -1.924075 1.779580 0.263975
H 0 -2.971780 1.326557 1.622514
H 0 -3.836451 1.790306 -1.290487
H 0 -4.988285 0.114170 0.193133
H 0 -5.822314 2.545808 1.032764
H 0 0.672238 -5.140897 0.971153
H 0 3.432145 -3.196741 1.979132
H 0 1.759438 -0.831390 2.540979
H 0 0.268681 -1.415588 1.770241
H 0 1.180005 -2.480291 2.849405
H 0 2.201388 0.678287 0.819169
H 0 3.790282 -0.080565 0.732137
H 0 2.339263 1.223717 -1.641586
H 0 3.931460 0.477412 -1.710910
H 0 3.196556 2.903423 0.021249
H 0 4.786139 2.153820 -0.038956
H 0 3.360659 3.447435 -2.428037
H 0 4.949215 2.698434 -2.488138
H 0 4.222921 5.159655 -0.779603
H 0 5.160616 5.185618 -2.282019
H 0 5.822314 4.403320 -0.837159
Free Energy (PCM/B3LYP/6-31G*) = -1757.654959
Number of imaginary frequencies = 0

1-3*R*-c2
B3LYP/6-31G* geometry
O 0 0.096526 -0.822439 0.173369
C 0 -1.721191 0.531599 0.925309
C 0 0.028119 1.606303 -0.440348
C 0 -1.105218 1.854259 0.594504
C 0 0.889968 0.415209 0.075723
C 0 -1.120253 -0.648908 0.683598
C 0 -1.993012 -1.819863 1.063477
N 0 -3.081034 -1.102114 1.757260
C 0 -3.052026 0.272544 1.456854
O 0 -4.336855 -1.721394 1.737563
C 0 -4.796067 -1.986491 3.079555
C 0 -2.450651 -2.693515 -0.122197
S 0 -3.361252 -1.799423 -1.449093
C 0 -5.058199 -2.536060 -1.372538
C 0 -6.177200 -1.490812 -1.286211
C 0 -6.102686 -0.505493 -2.469229
O 0 -6.221280 -0.739073 -0.082671
O 0 -6.131165 -0.857303 -3.630429
O 0 -6.004312 0.775067 -2.099927
O 0 -3.977347 1.060182 1.644201
O 0 -2.055542 2.777346 0.068494
O 0 0.925082 2.721399 -0.428252
C 0 -0.521272 1.378356 -1.852582
C 0 2.113567 0.084282 -0.767678
C 0 3.052727 -0.936725 -0.109247
C 0 4.286560 -1.243252 -0.967163
C 0 5.237318 -2.261096 -0.323973
C 0 6.467638 -2.564106 -1.185361
H 0 -0.629065 2.291295 1.486759
H 0 1.200018 0.652841 1.101578
H 0 -1.466148 -2.478351 1.765562
H 0 -4.094170 -2.648082 3.593837
H 0 -5.761284 -2.480133 2.952558
H 0 -4.916373 -1.050634 3.630429
H 0 -3.076851 -3.507434 0.246988
H 0 -1.557002 -3.137289 -0.568699
H 0 -5.114194 -3.204383 -0.510577
H 0 -5.214675 -3.125010 -2.278405
H 0 -7.125631 -2.034975 -1.397595
H 0 -5.499876 -1.006553 0.527090
H 0 -5.997775 0.756428 -1.110885
H 0 -2.873474 2.659931 0.587317
H 0 0.393750 3.507434 -0.640120
H 0 0.303197 1.282251 -2.563555
H 0 -1.139169 2.230345 -2.147417
H 0 -1.142451 0.479546 -1.913107
H 0 1.786640 -0.289757 -1.745501
H 0 2.648924 1.023124 -0.946133
H 0 2.503725 -1.866741 0.086941
H 0 3.375397 -0.553724 0.869862
H 0 3.962686 -1.620386 -1.948562
H 0 4.834168 -0.309756 -1.164567
H 0 4.688939 -3.193235 -0.127084
H 0 5.560709 -1.884267 0.656795
H 0 6.176675 -2.973665 -2.160700
H 0 7.125631 -3.294031 -0.699826
H 0 7.055033 -1.656000 -1.369620
Free Energy (PCM/B3LYP/6-31G*) = -1757.654904
Number of imaginary frequencies = 0

1-3*R*-c3
B3LYP/6-31G* geometry
O 0 1.292311 -0.594236 -0.213857
C 0 0.236750 -2.699400 -0.609733
C 0 2.631949 -2.708596 -0.043818
C 0 1.496930 -3.493928 -0.752591
C 0 2.575543 -1.236728 -0.550739
C 0 0.224747 -1.374457 -0.364643
C 0 -1.176960 -0.837884 -0.193887
N 0 -1.942091 -2.103633 -0.183886
C 0 -1.134787 -3.175664 -0.642409
O 0 -3.215845 -2.028026 -0.795292
C 0 -4.213396 -2.632317 0.048928
C 0 -1.326804 0.000803 1.082391
S 0 -2.997105 0.674128 1.437296
C 0 -3.184754 2.017415 0.188488
C 0 -4.039837 1.652168 -1.044736
C 0 -5.472027 1.293379 -0.646643
O 0 -3.482118 0.640002 -1.844531
O 0 -5.976465 0.202863 -0.813236
O 0 -6.106406 2.332464 -0.073604
O 0 -1.555478 -4.288638 -0.947336
O 0 1.388900 -4.796809 -0.185248
O 0 3.896175 -3.194627 -0.505084
C 0 2.543083 -2.817854 1.482646
C 0 3.671260 -0.322449 -0.019412

C 0 3.724118 1.040726 -0.724002
C 0 4.837362 1.944910 -0.180421
C 0 4.905881 3.310432 -0.876163
C 0 6.018376 4.210644 -0.328874
H 0 1.782910 -3.580576 -1.812627
H 0 2.616773 -1.265586 -1.646972
H 0 -1.468166 -0.243525 -1.067947
H 0 -4.226008 -2.153669 1.031534
H 0 -4.023468 -3.704172 0.141756
H 0 -5.158103 -2.452552 -0.466875
H 0 -0.612338 0.828821 1.051099
H 0 -1.089243 -0.615271 1.954912
H 0 -2.190182 2.322038 -0.148557
H 0 -3.639241 2.865839 0.706889
H 0 -4.091862 2.555302 -1.665938
H 0 -3.661701 -0.227826 -1.425627
H 0 -7.003065 2.034061 0.175244
H 0 0.559429 -5.177266 -0.527473
H 0 3.900663 -4.152230 -0.336552
H 0 1.627234 -2.365151 1.874721
H 0 2.549813 -3.870510 1.776978
H 0 3.401435 -2.321432 1.942252
H 0 3.527655 -0.177843 1.058276
H 0 4.624173 -0.847235 -0.148872
H 0 2.756345 1.547158 -0.615371
H 0 3.874093 0.885118 -1.802250
H 0 4.689039 2.095874 0.899176
H 0 5.805991 1.434312 -0.286964
H 0 3.937210 3.819100 -0.769449
H 0 5.054104 3.159712 -1.954912
H 0 5.878039 4.407204 0.741353
H 0 6.040629 5.177266 -0.845266
H 0 7.003065 3.743093 -0.453162
Free Energy (PCM/B3LYP/6-31G*) = -1757.654843
Number of imaginary frequencies = 0

1-3*R*-c4
B3LYP/6-31G* geometry
O 0 1.854594 -1.152064 -0.240684
C 0 1.693046 -3.487800 -0.694858
C 0 3.918935 -2.576259 -0.174803
C 0 3.157728 -3.722979 -0.889531
C 0 3.272921 -1.233431 -0.627112
C 0 1.166734 -2.281767 -0.400108
C 0 -0.327216 -2.353866 -0.188276
N 0 -0.511440 -3.821811 -0.184168
C 0 0.628059 -4.477315 -0.691317
O 0 -1.752265 -4.254110 -0.685106
C 0 -2.398901 -5.112650 0.265799
C 0 -0.850601 -1.729604 1.113989
S 0 -0.865301 0.112652 1.142869
C 0 -2.629033 0.516982 0.840477
C 0 -3.044124 0.521302 -0.644510
C 0 -4.503342 0.957557 -0.759564
O 0 -2.897023 -0.742322 -1.256397
O 0 -5.394481 0.189445 -1.066633
O 0 -4.686837 2.247736 -0.454032
O 0 0.685088 -5.667000 -1.000552
O 0 3.579086 -4.982161 -0.370583
O 0 5.257502 -2.514570 -0.678490
C 0 3.930331 -2.752172 1.347748
C 0 3.943253 0.024342 -0.090207
C 0 3.438166 1.314792 -0.751075
C 0 4.140462 2.569100 -0.216139
C 0 3.651562 3.866278 -0.873122
C 0 4.356558 5.116693 -0.337347
H 0 3.422993 -3.663382 -1.957143
H 0 3.286008 -1.217975 -1.724511
H 0 -0.872098 -1.923427 -1.035631
H 0 -3.361604 -5.359814 -0.188193
H 0 -2.556671 -4.599361 1.220369
H 0 -1.816500 -6.025117 0.424074
H 0 -0.242668 -2.061308 1.960171
H 0 -1.874631 -2.071567 1.278264
H 0 -2.766895 1.518033 1.259846
H 0 -3.258471 -0.176541 1.407964
H 0 -2.419707 1.241432 -1.184730
H 0 -3.781282 -1.155510 -1.250029
H 0 -5.643088 2.442195 -0.512963
H 0 2.921980 -5.635540 -0.675316
H 0 5.643088 -3.396969 -0.544199
H 0 2.923294 -2.710358 1.773952
H 0 4.361985 -3.723725 1.601853
H 0 4.536883 -1.969227 1.809962
H 0 3.791245 0.077414 0.994761
H 0 5.020309 -0.083928 -0.258877
H 0 2.355634 1.407275 -0.595194
H 0 3.591154 1.247780 -1.838090
H 0 3.989690 2.634784 0.871612
H 0 5.225734 2.472054 -0.368613
H 0 2.567364 3.962656 -0.719629
H 0 3.801072 3.799565 -1.960171
H 0 4.197124 5.229252 0.742268
H 0 3.985382 6.025117 -0.825755
H 0 5.439055 5.064559 -0.508341
Free Energy (PCM/B3LYP/6-31G*) = -1757.65458
Number of imaginary frequencies = 0

1-3*R*-c5
B3LYP/6-31G* geometry
O 0 -0.429809 -1.560244 -0.861847
C 0 -2.446443 -2.784455 -0.489146
C 0 -0.504387 -3.322579 0.925870
C 0 -1.838054 -3.865205 0.347926
C 0 0.295910 -2.688511 -0.251059
C 0 -1.738716 -1.764438 -1.011374
C 0 -2.612146 -0.819326 -1.799322
N 0 -3.946839 -1.321203 -1.419474
C 0 -3.848540 -2.603476 -0.835466
O 0 -4.920673 -1.176660 -2.424724
C 0 -6.066464 -0.482582 -1.910184
C 0 -2.423009 0.680021 -1.553065
S 0 -2.650777 1.208000 0.199366
C 0 -0.928030 1.573100 0.727572
C 0 -0.367435 2.924799 0.257836
C 0 -1.258385 4.094397 0.664011
O 0 -0.178321 2.953927 -1.141605
O 0 -1.711659 4.882130 -0.144146
O 0 -1.467875 4.158940 1.983276
O 0 -4.792889 -3.362554 -0.629374
O 0 -2.692630 -4.278997 1.410850
O 0 0.307205 -4.422823 1.349645
C 0 -0.737340 -2.352295 2.089340
C 0 1.683925 -2.174937 0.106701
C 0 2.521045 -1.773260 -1.116406
C 0 3.918960 -1.269453 -0.736528
C 0 4.770706 -0.867723 -1.947768
C 0 6.164324 -0.360848 -1.562430
H 0 -1.583635 -4.737561 -0.274822
H 0 0.377053 -3.449791 -1.037464
H 0 -2.463554 -0.999955 -2.874676
H 0 -6.746654 -0.395815 -2.760924
H 0 -5.795940 0.514070 -1.545410
H 0 -6.542094 -1.057662 -1.110327
H 0 -3.156737 1.221620 -2.155758
H 0 -1.432234 1.000322 -1.878196
H 0 -0.270403 0.773952 0.378143
H 0 -0.944249 1.545554 1.820485
H 0 0.601672 3.064975 0.762779
H 0 -0.705847 3.708073 -1.468942
H 0 -2.044140 4.927626 2.162878
H 0 -3.572973 -4.416561 1.015035
H 0 -0.224251 -4.927626 1.988388
H 0 -1.301855 -1.467352 1.779670
H 0 -1.309069 -2.853720 2.874676
H 0 0.219772 -2.030614 2.507808
H 0 1.589752 -1.321344 0.789176
H 0 2.191265 -2.972330 0.660586
H 0 1.993817 -0.994642 -1.682535
H 0 2.615063 -2.637496 -1.789895
H 0 3.823103 -0.407573 -0.059631
H 0 4.444700 -2.049130 -0.165534
H 0 4.242970 -0.090397 -2.518424
H 0 4.867798 -1.729485 -2.623214

H 0 6.098463 0.520471 -0.912332
H 0 6.746654 -0.079810 -2.447534
H 0 6.729547 -1.129796 -1.021190
Free Energy (PCM/B3LYP/6-31G*) = -1757.654275
Number of imaginary frequencies = 0

1-3*R*-c6
B3LYP/6-31G* geometry
O 0 -0.362117 -1.517664 -0.869429
C 0 -2.281490 -2.887346 -0.486843
C 0 -0.313498 -3.254909 0.943604
C 0 -1.595424 -3.909183 0.364211
C 0 0.444451 -2.577227 -0.236994
C 0 -1.651301 -1.821448 -1.017218
C 0 -2.593933 -0.944748 -1.804005
N 0 -3.887597 -1.539959 -1.415744
C 0 -3.694368 -2.809180 -0.826649
O 0 -4.875275 -1.469500 -2.414862
C 0 -6.059552 -0.844187 -1.898844
C 0 -2.509650 0.562946 -1.553589
S 0 -2.758742 1.053311 0.207966
C 0 -1.086495 1.652625 0.682640
C 0 -0.742037 3.073858 0.209828
C 0 -1.765338 4.101726 0.681845
O 0 -0.631038 3.146315 -1.196045
O 0 -2.375627 4.818215 -0.088405
O 0 -1.903746 4.129017 2.011838
O 0 -4.581321 -3.632349 -0.611387
O 0 -2.421747 -4.377748 1.426642
O 0 0.577949 -4.281432 1.390969
C 0 -0.633939 -2.287512 2.088330
C 0 1.784594 -1.951201 0.124198
C 0 2.605441 -1.513678 -1.097563
C 0 3.952937 -0.891421 -0.711282
C 0 4.791966 -0.455938 -1.919609
C 0 6.133447 0.171035 -1.526160
H 0 -1.268228 -4.765186 -0.247179
H 0 0.591806 -3.341041 -1.011313
H 0 -2.439261 -1.114016 -2.880181
H 0 -6.747930 -0.805011 -2.746499
H 0 -5.849009 0.169519 -1.541578
H 0 -6.494835 -1.442253 -1.092847
H 0 -3.280791 1.055873 -2.151021
H 0 -1.544758 0.951872 -1.881519
H 0 -0.337123 0.955826 0.299736
H 0 -1.057424 1.613647 1.774959
H 0 0.223224 3.341482 0.667982
H 0 -1.297202 3.799548 -1.485069
H 0 -2.573647 4.804860 2.234699
H 0 -3.289532 -4.577693 1.029506
H 0 0.080255 -4.818215 2.030860
H 0 -1.269762 -1.460379 1.757994
H 0 -1.165522 -2.821851 2.880181
H 0 0.289813 -1.877873 2.504934
H 0 1.615855 -1.092465 0.785592
H 0 2.344386 -2.694537 0.702187
H 0 2.027016 -0.793344 -1.690366
H 0 2.777996 -2.383725 -1.747669
H 0 3.778165 -0.022190 -0.059922
H 0 4.528149 -1.611847 -0.110840
H 0 4.214772 0.260851 -2.520825
H 0 4.970002 -1.325053 -2.568630
H 0 5.985674 1.061455 -0.902381
H 0 6.707889 0.473845 -2.409221
H 0 6.747930 -0.535374 -0.954223
Free Energy (PCM/B3LYP/6-31G*) = -1757.654057
Number of imaginary frequencies = 0

1-3*R*-c7
B3LYP/6-31G* geometry
O 0 -0.419179 -0.305750 0.108769
C 0 0.820621 -2.346694 0.049126
C 0 -1.204374 -2.193173 -1.346169
C 0 -0.222035 -3.167717 -0.639167
C 0 -1.592678 -1.082728 -0.322802
C 0 0.666242 -1.043731 0.342707
C 0 1.889033 -0.466774 1.012123
N 0 2.688076 -1.696421 1.180996
C 0 2.165657 -2.760679 0.439293
O 0 4.074237 -1.563483 1.249863
C 0 4.541485 -1.853711 2.578649
C 0 2.592177 0.667535 0.243638
S 0 3.047436 0.265830 -1.498677
C 0 4.882876 0.136322 -1.442059
C 0 5.608552 1.479528 -1.599847
C 0 5.620658 2.321606 -0.308598
O 0 5.122067 2.263002 -2.686362
O 0 5.831966 1.861400 0.794883
O 0 5.421847 3.628536 -0.513084
O 0 2.730975 -3.827925 0.201926
O 0 0.342011 -4.073835 -1.584951
O 0 -2.435835 -2.875158 -1.608107
C 0 -0.620531 -1.630542 -2.646400
C 0 -2.623261 -0.077414 -0.818596
C 0 -3.154266 0.851540 0.282852
C 0 -4.203109 1.842076 -0.237995
C 0 -4.748447 2.779979 0.846869
C 0 -5.799397 3.762533 0.319910
H 0 -0.814148 -3.735504 0.096582
H 0 -1.968106 -1.584619 0.578494
H 0 1.619549 -0.057667 1.995236
H 0 4.320955 -2.891477 2.845736
H 0 4.094260 -1.172867 3.310056
H 0 5.621440 -1.697601 2.535786
H 0 3.492873 0.961799 0.782850
H 0 1.914145 1.524253 0.218889
H 0 5.141425 -0.505949 -2.287646
H 0 5.184102 -0.362102 -0.519859
H 0 6.661059 1.264182 -1.823537
H 0 4.151039 2.136239 -2.707917
H 0 5.264113 3.728494 -1.480962
H 0 1.160701 -4.417720 -1.178287
H 0 -2.214497 -3.647158 -2.155905
H 0 -1.364012 -1.009167 -3.152178
H 0 0.276698 -1.029606 -2.469276
H 0 -0.343875 -2.453867 -3.310056
H 0 -2.182483 0.519923 -1.625933
H 0 -3.449595 -0.648000 -1.256117
H 0 -2.318849 1.404802 0.731031
H 0 -3.593429 0.245105 1.088283
H 0 -3.764950 2.442678 -1.048875
H 0 -5.037582 1.284103 -0.688145
H 0 -3.914473 3.339808 1.293426
H 0 -5.182718 2.179572 1.658872
H 0 -5.384076 4.400055 -0.470475
H 0 -6.169623 4.417720 1.116891
H 0 -6.661059 3.230703 -0.102439
Free Energy (PCM/B3LYP/6-31G*) = -1757.65399
Number of imaginary frequencies = 0

1-3*R*-c8
B3LYP/6-31G* geometry
O 0 0.284554 -0.147278 -0.140438
C 0 -1.382380 1.556087 0.013776
C 0 0.487690 1.917640 -1.551787
C 0 -0.618102 2.616493 -0.712515
C 0 1.209757 0.892019 -0.626124
C 0 -0.911660 0.312224 0.220308
C 0 -1.904602 -0.553264 0.959801
N 0 -2.925414 0.465566 1.291837
C 0 -2.733803 1.645797 0.554815
O 0 -4.239256 0.024029 1.452404
C 0 -4.655861 0.139755 2.827196
C 0 -2.435972 -1.758499 0.169537
S 0 -3.127703 -1.289807 -1.478179
C 0 -4.422206 -2.583174 -1.695474
C 0 -5.827855 -2.113941 -1.316670
C 0 -6.353513 -1.034541 -2.284329
O 0 -5.975596 -1.684283 0.030568
O 0 -6.149621 -1.041395 -3.479754
O 0 -7.108686 -0.099863 -1.691362
O 0 -3.544633 2.561473 0.437702
O 0 -1.454418 3.402854 -1.557602
O 0 1.503099 2.872996 -1.876038

C 0 -0.072172 1.279056 -2.827232
C 0 2.387289 0.163932 -1.259792
C 0 3.219550 -0.646727 -0.256244
C 0 4.405577 -1.361496 -0.915926
C 0 5.257810 -2.168671 0.071571
C 0 6.440444 -2.879056 -0.594950
H 0 -0.101963 3.276054 0.003372
H 0 1.544525 1.434921 0.267249
H 0 -1.453029 -0.944431 1.880254
H 0 -4.648204 1.188419 3.135099
H 0 -4.010092 -0.455014 3.479754
H 0 -5.674301 -0.253015 2.845288
H 0 -3.217090 -2.248061 0.754139
H 0 -1.612216 -2.464065 0.033322
H 0 -4.145133 -3.464953 -1.113388
H 0 -4.417997 -2.856117 -2.752503
H 0 -6.503107 -2.972579 -1.428839
H 0 -5.246464 -1.068514 0.263683
H 0 -7.099089 -0.313291 -0.730350
H 0 -2.281915 3.553566 -1.062303
H 0 1.065595 3.586222 -2.370939
H 0 0.744610 0.875486 -3.431078
H 0 -0.776355 0.470789 -2.608010
H 0 -0.601383 2.034075 -3.414265
H 0 2.015195 -0.496514 -2.052564
H 0 3.016077 0.920500 -1.741451
H 0 2.578021 -1.385242 0.241705
H 0 3.590703 0.024521 0.531870
H 0 4.033011 -2.031957 -1.704684
H 0 5.041380 -0.619554 -1.421451
H 0 4.622104 -2.909848 0.576433
H 0 5.630359 -1.498614 0.859471
H 0 6.097557 -3.586222 -1.360517
H 0 7.033201 -3.440827 0.136182
H 0 7.108686 -2.159775 -1.084638
Free Energy (PCM/B3LYP/6-31G*) = -1757.653944
Number of imaginary frequencies = 0

1-3*R*-c9
B3LYP/6-31G* geometry
O 0 0.923165 -0.035178 -0.122917
C 0 -0.618210 1.783313 0.016689
C 0 1.348523 2.050391 -1.448095
C 0 0.252373 2.804438 -0.643933
C 0 1.944863 0.950611 -0.518877
C 0 -0.252788 0.500463 0.198033
C 0 -1.352534 -0.320237 0.829276
N 0 -2.288793 0.766856 1.186742
C 0 -1.985712 1.950363 0.495024
O 0 -3.639080 0.429477 1.308152
C 0 -4.101169 0.668342 2.653940
C 0 -1.939775 -1.407258 -0.093960
S 0 -2.546464 -0.748431 -1.704815
C 0 -4.182323 -1.557030 -1.891467
C 0 -5.244860 -1.094346 -0.882301
C 0 -6.604004 -1.725612 -1.220675
O 0 -5.413470 0.315459 -0.862962
O 0 -6.749241 -2.903154 -1.473418
O 0 -7.628901 -0.864433 -1.194803
O 0 -2.734660 2.917175 0.369880
O 0 -0.486723 3.664073 -1.508503
O 0 2.444507 2.938825 -1.690759
C 0 0.815182 1.488675 -2.769938
C 0 3.107209 0.157966 -1.102017
C 0 3.818393 -0.722837 -0.065165
C 0 4.985037 -1.521636 -0.659421
C 0 5.713198 -2.389744 0.374531
C 0 6.875701 -3.191091 -0.220221
H 0 0.776247 3.411655 0.111442
H 0 2.260987 1.442879 0.410041
H 0 -0.991221 -0.819874 1.736363
H 0 -4.021691 1.730562 2.897805
H 0 -3.528219 0.067262 3.365154
H 0 -5.147627 0.357205 2.651139
H 0 -2.761287 -1.907211 0.422069
H 0 -1.156459 -2.148163 -0.274549
H 0 -4.082282 -2.644038 -1.853156
H 0 -4.490832 -1.281679 -2.904974
H 0 -4.981304 -1.458186 0.118752
H 0 -4.819418 0.668711 -0.169616
H 0 -7.236469 0.013922 -0.978806
H 0 -1.321549 3.868314 -1.046344
H 0 2.085183 3.694312 -2.185984
H 0 1.631377 1.034113 -3.337477
H 0 0.036578 0.735998 -2.614242
H 0 0.383095 2.297195 -3.365154
H 0 2.739610 -0.461338 -1.929372
H 0 3.813137 0.878501 -1.528991
H 0 3.094773 -1.414363 0.385618
H 0 4.191041 -0.088018 0.751887
H 0 4.611513 -2.161839 -1.472405
H 0 5.703459 -0.828644 -1.122097
H 0 4.993219 -3.078460 0.839087
H 0 6.087836 -1.748265 1.184909
H 0 6.527096 -3.868314 -1.009880
H 0 7.374414 -3.798668 0.543773
H 0 7.628901 -2.527114 -0.662454
Free Energy (PCM/B3LYP/6-31G*) = -1757.65391
Number of imaginary frequencies = 0

1-3*R*-c10
B3LYP/6-31G* geometry
O 0 1.052881 -1.063187 -0.291914
C 0 0.682578 -3.419200 -0.279065
C 0 2.730507 -2.575108 0.796718
C 0 2.063098 -3.812728 0.138890
C 0 2.487452 -1.356402 -0.143271
C 0 0.283592 -2.142378 -0.433091
C 0 -1.178630 -2.049198 -0.804971
N 0 -1.463756 -3.489758 -1.035976
C 0 -0.451392 -4.303102 -0.516577
O 0 -2.781010 -3.921728 -0.869390
C 0 -3.347459 -4.285121 -2.139159
C 0 -2.085841 -1.456432 0.292327
S 0 -1.971015 0.370074 0.452169
C 0 -3.143381 0.939835 -0.844495
C 0 -4.625801 0.924204 -0.441357
C 0 -4.891279 1.724115 0.830695
O 0 -5.104677 -0.392994 -0.265035
O 0 -5.457031 1.246858 1.795535
O 0 -4.457669 2.986885 0.754762
O 0 -0.519049 -5.519056 -0.330705
O 0 2.056770 -4.913321 1.046042
O 0 4.151747 -2.748757 0.793127
C 0 2.227181 -2.339347 2.225113
C 0 3.155631 -0.058405 0.290074
C 0 3.080188 1.051977 -0.767455
C 0 3.788313 2.339023 -0.326265
C 0 3.715364 3.461360 -1.369489
C 0 4.431790 4.742072 -0.929156
H 0 2.674606 -4.072512 -0.740311
H 0 2.841684 -1.638404 -1.143574
H 0 -1.309294 -1.490233 -1.738600
H 0 -4.369720 -4.595560 -1.911700
H 0 -2.793529 -5.116416 -2.585883
H 0 -3.360989 -3.431598 -2.825141
H 0 -1.816729 -1.872660 1.267251
H 0 -3.123397 -1.713553 0.082785
H 0 -3.015243 0.333677 -1.745589
H 0 -2.842703 1.962136 -1.087875
H 0 -5.185665 1.417503 -1.252766
H 0 -5.496493 -0.419284 0.629456
H 0 -4.667866 3.433178 1.598517
H 0 1.364213 -5.523543 0.726020
H 0 4.330598 -3.578110 1.267754
H 0 1.156764 -2.114204 2.252021
H 0 2.394880 -3.237431 2.825141
H 0 2.769958 -1.507450 2.681212
H 0 2.697216 0.288054 1.224354
H 0 4.202508 -0.291399 0.513392
H 0 2.028935 1.272210 -0.993090
H 0 3.530174 0.691432 -1.704019
H 0 3.346780 2.692842 0.617313
H 0 4.842901 2.116802 -0.105169

H 0   2.661029   3.686373   -1.584463
H 0   4.150039   3.105296   -2.314404
H 0   3.997838   5.140691   -0.003616
H 0   4.360200   5.523543   -1.694477
H 0   5.496493   4.555235   -0.741567
Free Energy (PCM/B3LYP/6-31G*) = -1757.653849
Number of imaginary frequencies = 0

1-3*R*-c11
B3LYP/6-31G* geometry
O 0   0.388080   -0.693137   -0.793411
C 0   -0.840011   -2.664398   -0.233195
C 0   0.904329   -1.920633   1.336885
C 0   0.090860   -3.144434   0.835511
C 0   1.466517   -1.179185   0.086607
C 0   -0.632849   -1.533047   -0.934153
C 0   -1.743521   -1.274016   -1.923851
N 0   -2.726618   -2.282171   -1.484592
C 0   -2.110006   -3.241614   -0.645773
O 0   -3.524780   -2.796298   -2.521776
C 0   -4.915184   -2.631990   -2.204828
C 0   -2.282034   0.156120   -1.973120
S 0   -3.038326   0.659025   -0.367297
C 0   -3.023630   2.497463   -0.568677
C 0   -4.382397   3.102237   -0.203698
C 0   -4.660557   3.044079   1.312208
O 0   -5.470376   2.536889   -0.928145
O 0   -3.827689   3.296541   2.156502
O 0   -5.925340   2.730082   1.619673
O 0   -2.611419   -4.312299   -0.313298
O 0   -0.596382   -3.748433   1.928127
O 0   2.070610   -2.384652   2.024751
C 0   0.072255   -1.010396   2.246669
C 0   2.353868   0.020877   0.388935
C 0   3.080248   0.571535   -0.846525
C 0   3.978874   1.770560   -0.518081
C 0   4.716133   2.331401   -1.740828
C 0   5.611369   3.529247   -1.406731
H 0   0.817770   -3.861444   0.422153
H 0   2.026511   -1.913486   -0.506264
H 0   -1.399095   -1.530521   -2.936054
H 0   -5.182812   -3.215644   -1.318970
H 0   -5.448855   -3.014767   -3.078000
H 0   -5.161972   -1.576867   -2.046867
H 0   -3.022094   0.240825   -2.772538
H 0   -1.449680   0.829929   -2.192861
H 0   -2.798879   2.725523   -1.612837
H 0   -2.250606   2.932958   0.066811
H 0   -4.356053   4.167123   -0.465578
H 0   -5.275814   1.581392   -1.020522
H 0   -6.377754   2.555351   0.762406
H 0   -1.252078   -4.355167   1.537730
H 0   1.755584   -2.962821   2.740152
H 0   0.701504   -0.214109   2.652480
H 0   -0.332396   -1.593428   3.078000
H 0   -0.767763   -0.554886   1.713446
H 0   1.745213   0.811229   0.844742
H 0   3.084032   -0.296350   1.141441
H 0   2.343197   0.864405   -1.605310
H 0   3.688192   -0.227222   -1.295855
H 0   3.370465   2.567251   -0.064774
H 0   4.713972   1.476124   0.245515
H 0   3.980522   2.624390   -2.503307
H 0   5.323962   1.534997   -2.193268
H 0   5.025620   4.355167   -0.984246
H 0   6.124726   3.906338   -2.298781
H 0   6.377754   3.256671   -0.670552
Free Energy (PCM/B3LYP/6-31G*) = -1757.65383
Number of imaginary frequencies = 0

1-3*R*-c12
B3LYP/6-31G* geometry
O 0   1.880884   -1.093148   -0.472699
C 0   1.889069   -3.471534   -0.688021
C 0   3.907800   -2.397471   0.222734
C 0   3.364534   -3.651037   -0.513932
C 0   3.352933   -1.141331   -0.513068
C 0   1.284743   -2.268517   -0.658353
C 0   -0.215791   -2.383847   -0.796234
N 0   -0.376054   -3.848589   -0.693797
C 0   0.870748   -4.497870   -0.845055
O 0   -1.428596   -4.371181   -1.472260
C 0   -2.256548   -5.230664   -0.673513
C 0   -0.964658   -1.583893   0.272093
S 0   -2.782629   -1.595727   -0.032121
C 0   -3.338329   -0.372026   1.220125
C 0   -3.232196   1.099403   0.792360
C 0   -3.990028   1.386039   -0.500686
O 0   -1.886930   1.500294   0.634901
O 0   -3.464073   1.913359   -1.462111
O 0   -5.274969   1.019113   -0.447398
O 0   1.020335   -5.705295   -1.019342
O 0   3.686473   -4.824464   0.228181
O 0   5.324242   -2.319697   0.035504
C 0   3.566256   -2.415060   1.716877
C 0   3.846782   0.197108   0.019353
C 0   3.462179   1.387300   -0.871183
C 0   3.974884   2.725715   -0.325035
C 0   3.597386   3.925630   -1.203123
C 0   4.109694   5.260412   -0.652120
H 0   3.869363   -3.688757   -1.492171
H 0   3.619692   -1.238143   -1.573299
H 0   -0.526808   -2.059461   -1.798647
H 0   -3.052331   -5.555939   -1.348161
H 0   -2.683607   -4.684215   0.172967
H 0   -1.687696   -6.096873   -0.323870
H 0   -0.626660   -0.546677   0.247852
H 0   -0.758548   -2.006428   1.260367
H 0   -2.764284   -0.517909   2.139619
H 0   -4.382202   -0.613356   1.436383
H 0   -3.711022   1.700347   1.583192
H 0   -1.827551   1.895945   -0.256284
H 0   -5.691486   1.240528   -1.303425
H 0   3.155740   -5.545690   -0.157246
H 0   5.691486   -3.161612   0.354329
H 0   2.486683   -2.378727   1.891353
H 0   3.948416   -3.333279   2.170205
H 0   4.029168   -1.560457   2.216620
H 0   3.453786   0.349366   1.031913
H 0   4.936941   0.130451   0.104482
H 0   2.370259   1.429351   -0.974715
H 0   3.864235   1.227876   -1.882273
H 0   3.578175   2.880646   0.689315
H 0   5.069291   2.681802   -0.221378
H 0   2.503803   3.967594   -1.306475
H 0   3.994003   3.771207   -2.216620
H 0   3.702727   5.457906   0.347421
H 0   3.823481   6.096873   -1.300130
H 0   5.203763   5.260897   -0.570023
Free Energy (PCM/B3LYP/6-31G*) = -1757.653823
Number of imaginary frequencies = 0

1-3*R*-c13
B3LYP/6-31G* geometry
O 0   1.473559   -1.103575   -0.516597
C 0   1.513407   -3.483252   -0.676443
C 0   3.473251   -2.370621   0.312638
C 0   2.979833   -3.644041   -0.423508
C 0   2.945212   -1.138898   -0.480310
C 0   0.892292   -2.286788   -0.703365
C 0   -0.600025   -2.431506   -0.899702
N 0   -0.736947   -3.895281   -0.745900
C 0   0.518382   -4.532215   -0.830603
O 0   -1.773339   -4.460509   -1.509744
C 0   -2.650139   -5.225982   -0.669442
C 0   -1.487284   -1.678194   0.104388
S 0   -1.672185   0.118486   -0.250595
C 0   -3.119765   0.126411   -1.384934
C 0   -4.491154   0.033489   -0.697986
C 0   -4.690868   1.123504   0.351017
O 0   -4.686557   -1.228335   -0.092741
O 0   -4.996117   0.874047   1.501450
O 0   -4.514002   2.356664   -0.134901
O 0   0.692177   -5.743873   -0.948841

O 0 3.272414 -4.799727 0.357601
O 0 4.897053 -2.282797 0.198722
C 0 3.053996 -2.358076 1.786795
C 0 3.398888 0.217359 0.043122
C 0 3.066036 1.377798 -0.905498
C 0 3.535575 2.735768 -0.369020
C 0 3.221407 3.903959 -1.312397
C 0 3.691754 5.258389 -0.772052
H 0 3.534834 -3.696833 -1.373529
H 0 3.267619 -1.258547 -1.522615
H 0 -0.877344 -2.156978 -1.925952
H 0 -2.118565 -6.071021 -0.222184
H 0 -3.432232 -5.590859 -1.339546
H 0 -3.091370 -4.602371 0.115065
H 0 -1.077904 -1.764078 1.114547
H 0 -2.488443 -2.109334 0.103933
H 0 -3.027307 -0.694106 -2.101988
H 0 -3.055139 1.063856 -1.943349
H 0 -5.254071 0.193744 -1.477297
H 0 -4.923016 -1.046464 0.837537
H 0 -4.663680 2.994529 0.590393
H 0 2.750924 -5.528427 -0.027462
H 0 5.254071 -3.109316 0.565718
H 0 3.475969 -1.483653 2.288576
H 0 1.966323 -2.336698 1.903535
H 0 3.426518 -3.258295 2.282396
H 0 2.941529 0.395767 1.024033
H 0 4.481814 0.160948 0.198767
H 0 1.982966 1.408142 -1.081280
H 0 3.534290 1.190507 -1.882845
H 0 3.066218 2.923079 0.608175
H 0 4.619691 2.699794 -0.185034
H 0 2.138207 3.938809 -1.496203
H 0 3.690716 3.716276 -2.288576
H 0 3.212079 5.489980 0.187102
H 0 3.454479 6.071021 -1.468363
H 0 4.776754 5.264279 -0.609362
Free Energy (PCM/B3LYP/6-31G*) = -1757.65367
Number of imaginary frequencies = 0

1-3*R*-c14
B3LYP/6-31G* geometry
O 0 0.576949 -0.866104 -0.900266
C 0 -0.160075 -3.066587 -0.334004
C 0 1.447065 -1.977331 1.178257
C 0 0.899943 -3.343429 0.684818
C 0 1.774161 -1.115298 -0.077695
C 0 -0.241187 -1.908338 -1.017030
C 0 -1.428326 -1.886827 -1.950080
N 0 -2.139876 -3.095154 -1.491560
C 0 -1.289957 -3.905326 -0.704524
O 0 -2.867076 -3.755592 -2.496204
C 0 -4.242200 -3.888219 -2.105055
C 0 -2.272534 -0.611171 -1.937159
S 0 -3.071361 -0.327920 -0.302700
C 0 -3.480703 1.453634 -0.514763
C 0 -4.571288 1.856414 0.494309
C 0 -4.848286 3.353246 0.365826
O 0 -4.212816 1.563416 1.827319
O 0 -4.521688 4.154851 1.218843
O 0 -5.449832 3.674344 -0.786201
O 0 -1.526042 -5.065427 -0.375622
O 0 0.410780 -4.096483 1.791451
O 0 2.718294 -2.180289 1.804709
C 0 0.479385 -1.284531 2.144581
C 0 2.391526 0.246333 0.212191
C 0 2.939647 0.946161 -1.039706
C 0 3.556169 2.316216 -0.731563
C 0 4.134275 3.015442 -1.968762
C 0 4.744766 4.385572 -1.656467
H 0 1.747017 -3.878668 0.227079
H 0 2.453175 -1.702706 -0.708916
H 0 -1.084191 -2.048491 -2.981830
H 0 -4.732570 -4.361295 -2.959202
H 0 -4.691472 -2.909606 -1.907299
H 0 -4.333941 -4.527229 -1.221585
H 0 -3.037422 -0.674619 -2.716269
H 0 -1.617893 0.235236 -2.166219
H 0 -3.847345 1.616572 -1.532313
H 0 -2.581157 2.059204 -0.360831
H 0 -5.490697 1.307287 0.261621
H 0 -3.839113 2.385833 2.196252
H 0 -5.561575 4.645218 -0.811004
H 0 -0.114036 -4.826755 1.414780
H 0 2.573530 -2.823113 2.519634
H 0 -0.467075 -1.021668 1.661903
H 0 0.256565 -1.951156 2.981830
H 0 0.935459 -0.372421 2.538203
H 0 3.199195 0.090452 0.935670
H 0 1.642234 0.883025 0.698279
H 0 2.134484 1.064883 -1.776384
H 0 3.697863 0.302695 -1.509224
H 0 2.794208 2.963274 -0.272300
H 0 4.349623 2.197348 0.021287
H 0 3.342517 3.130248 -2.722696
H 0 4.898189 2.369588 -2.424456
H 0 3.995459 5.065427 -1.232232
H 0 5.150554 4.857661 -2.558675
H 0 5.561575 4.298022 -0.929229
Free Energy (PCM/B3LYP/6-31G*) = -1757.65346
Number of imaginary frequencies = 0

1-3*R*-c15
B3LYP/6-31G* geometry
O 0 0.477404 0.420551 -0.330371
C 0 2.308392 1.929744 -0.073945
C 0 0.534650 1.989654 1.633186
C 0 1.652191 2.814257 0.941352
C 0 -0.325746 1.323389 0.518716
C 0 1.697871 0.852472 -0.612843
C 0 2.582966 0.126697 -1.594493
N 0 3.889054 0.775698 -1.322595
C 0 3.651426 2.010013 -0.602310
O 0 4.604252 0.989370 -2.532515
C 0 5.977359 0.598809 -2.404999
C 0 2.581951 -1.402333 -1.498861
S 0 2.791832 -2.116392 0.185306
C 0 4.554801 -2.661444 0.244557
C 0 5.451078 -1.793723 1.130521
C 0 4.923580 -1.751923 2.578977
O 0 5.664777 -0.469727 0.659834
O 0 4.595200 -2.742101 3.198580
O 0 4.883945 -0.523213 3.106535
O 0 4.495453 2.883820 -0.462870
O 0 2.563968 3.294617 1.924111
O 0 -0.372929 2.884950 2.282380
C 0 1.105049 0.982609 2.638467
C 0 -1.514755 0.509642 1.011292
C 0 -2.466803 0.082464 -0.114750
C 0 -3.661478 -0.733881 0.394155
C 0 -4.623313 -1.161058 -0.721804
C 0 -5.813176 -1.980744 -0.211925
H 0 1.161911 3.667679 0.447595
H 0 -0.674267 2.121325 -0.148504
H 0 2.284817 0.392561 -2.618356
H 0 6.071870 -0.471080 -2.193456
H 0 6.481571 1.179941 -1.628387
H 0 6.415158 0.823217 -3.380399
H 0 3.342421 -1.815073 -2.166549
H 0 1.611184 -1.760793 -1.851756
H 0 4.953499 -2.709101 -0.771883
H 0 4.549136 -3.673464 0.654457
H 0 6.435209 -2.279106 1.177322
H 0 4.931491 -0.194457 0.063250
H 0 5.192342 0.080069 2.388832
H 0 3.320542 3.673464 1.441968
H 0 0.157514 3.415688 2.900752
H 0 0.290506 0.470114 3.156461
H 0 1.741364 0.233235 2.157776
H 0 1.711259 1.508909 3.380399
H 0 -1.148416 -0.374750 1.546466
H 0 -2.050457 1.127559 1.740187
H 0 -1.913578 -0.505560 -0.858467
H 0 -2.833159 0.978208 -0.637055

H 0 -3.294108 -1.628639 0.918113
H 0 -4.212649 -0.146081 1.143180
H 0 -4.070292 -1.745181 -1.471039
H 0 -4.991535 -0.266274 -1.243593
H 0 -5.477253 -2.900692 0.282447
H 0 -6.481571 -2.267845 -1.031896
H 0 -6.403907 -1.410681 0.515991
Free Energy (PCM/B3LYP/6-31G*) = -1757.653339
Number of imaginary frequencies = 0

1-3*R*-c16
B3LYP/6-31G* geometry
O 0 1.897864 -1.049017 -0.855487
C 0 1.588036 -3.404423 -0.608230
C 0 3.410435 -2.351539 0.665939
C 0 2.907697 -3.679876 0.040885
C 0 3.293003 -1.242833 -0.422026
C 0 1.199215 -2.173700 -0.992603
C 0 -0.199363 -2.174274 -1.565329
N 0 -0.613701 -3.554278 -1.240176
C 0 0.504873 -4.337347 -0.875009
O 0 -1.478046 -4.136723 -2.188866
C 0 -2.616013 -4.716666 -1.531827
C 0 -1.088518 -1.086911 -0.956749
S 0 -2.718508 -1.018916 -1.811560
C 0 -3.409817 0.539090 -1.137334
C 0 -4.026425 0.402144 0.269918
C 0 -4.654882 1.733436 0.679101
O 0 -3.080662 0.024268 1.247511
O 0 -4.187511 2.436175 1.554558
O 0 -5.736248 2.045256 -0.045523
O 0 0.503023 -5.561462 -0.763631
O 0 2.821128 -4.684478 1.048024
O 0 4.815727 -2.453437 0.916157
C 0 2.662648 -2.004358 1.958121
C 0 3.814016 0.127823 -0.011366
C 0 3.917365 1.118422 -1.179915
C 0 4.435750 2.494640 -0.744475
C 0 4.552911 3.495268 -1.901386
C 0 5.063510 4.870750 -1.459940
H 0 3.654505 -3.981198 -0.710639
H 0 3.835543 -1.595205 -1.308444
H 0 -0.157624 -2.062054 -2.657577
H 0 -2.305467 -5.533552 -0.874376
H 0 -3.236367 -5.106881 -2.342445
H 0 -3.169027 -3.958866 -0.968594
H 0 -0.593737 -0.119525 -1.087963
H 0 -1.236958 -1.265029 0.109151
H 0 -4.183455 0.843290 -1.848442
H 0 -2.629739 1.308136 -1.133756
H 0 -4.813887 -0.359327 0.236375
H 0 -2.829509 0.845746 1.711118
H 0 -6.059352 2.919465 0.249560
H 0 2.310524 -5.418354 0.659150
H 0 4.938764 -3.235787 1.480212
H 0 1.596180 -1.832491 1.783152
H 0 2.756316 -2.829701 2.668602
H 0 3.091579 -1.105552 2.408292
H 0 3.161909 0.540068 0.768246
H 0 4.800640 -0.021078 0.440944
H 0 2.933314 1.231917 -1.652830
H 0 4.585341 0.703067 -1.948403
H 0 3.767027 2.907981 0.025081
H 0 5.418552 2.378157 -0.263996
H 0 3.571135 3.606732 -2.383364
H 0 5.224353 3.084268 -2.668602
H 0 4.393669 5.321804 -0.717291
H 0 5.134567 5.561462 -2.308098
H 0 6.059352 4.796497 -1.005659
Free Energy (PCM/B3LYP/6-31G*) = -1757.653306
Number of imaginary frequencies = 0

1-3*R*-c17
B3LYP/6-31G* geometry
O 0 2.034499 -1.136730 -0.429357
C 0 1.905225 -3.513475 -0.617636
C 0 3.992636 -2.547454 0.257508
C 0 3.370375 -3.773973 -0.460936
C 0 3.501250 -1.268084 -0.483055
C 0 1.369270 -2.278196 -0.595102
C 0 -0.139425 -2.310758 -0.690961
N 0 -0.381274 -3.764637 -0.598939
C 0 0.829666 -4.481297 -0.753509
O 0 -1.445019 -4.229223 -1.399297
C 0 -2.346952 -5.023751 -0.615848
C 0 -0.797721 -1.483063 0.424677
S 0 -2.626799 -1.386890 0.385607
C 0 -2.884041 -0.325591 -1.100249
C 0 -4.141749 0.530764 -0.926876
C 0 -4.024424 1.529489 0.223070
O 0 -5.270408 -0.290203 -0.709175
O 0 -4.863844 1.615395 1.098944
O 0 -2.939597 2.306706 0.136286
O 0 0.910298 -5.696060 -0.921371
O 0 3.636357 -4.957334 0.287478
O 0 5.408976 -2.552714 0.053318
C 0 3.669416 -2.532806 1.755927
C 0 4.073940 0.044583 0.034610
C 0 3.758419 1.245264 -0.868855
C 0 4.346790 2.558142 -0.337272
C 0 4.045146 3.766098 -1.233534
C 0 4.630056 5.076294 -0.695977
H 0 3.859104 -3.848274 -1.445205
H 0 3.752314 -1.387041 -1.544893
H 0 -0.465929 -1.955745 -1.676488
H 0 -3.141225 -5.309155 -1.310356
H 0 -2.764544 -4.440209 0.209973
H 0 -1.844535 -5.919102 -0.238225
H 0 -0.387188 -0.468919 0.404066
H 0 -0.551764 -1.926097 1.394311
H 0 -3.002533 -0.950007 -1.989458
H 0 -2.012845 0.320456 -1.237092
H 0 -4.270124 1.121830 -1.848576
H 0 -5.730396 0.089201 0.064458
H 0 -2.947675 2.925641 0.892617
H 0 3.062856 -5.651556 -0.086177
H 0 5.730396 -3.412022 0.375283
H 0 2.596160 -2.431878 1.943871
H 0 4.003255 -3.468037 2.212765
H 0 4.187925 -1.702468 2.241832
H 0 3.692067 0.229568 1.046010
H 0 5.158532 -0.084191 0.119229
H 0 2.670719 1.348766 -0.974140
H 0 4.151094 1.051724 -1.877647
H 0 3.955109 2.749644 0.672741
H 0 5.436187 2.451167 -0.227041
H 0 2.956517 3.869615 -1.345925
H 0 4.439222 3.575503 -2.241832
H 0 4.228235 5.310484 0.297656
H 0 4.397559 5.919102 -1.357124
H 0 5.721694 5.014432 -0.605408
Free Energy (PCM/B3LYP/6-31G*) = -1757.65329
Number of imaginary frequencies = 0

1-3*R*-c18
B3LYP/6-31G* geometry
O 0 0.963785 0.218510 -0.052448
C 0 -0.391331 2.184204 0.007789
C 0 1.576836 2.192622 -1.475105
C 0 0.571185 3.086079 -0.696428
C 0 2.070538 1.076292 -0.505211
C 0 -0.150790 0.884274 0.252008
C 0 -1.308151 0.214874 0.951058
N 0 -2.167923 1.390289 1.188127
C 0 -1.740843 2.505836 0.464849
O 0 -3.536494 1.180551 1.339035
C 0 -3.938446 1.439344 2.695685
C 0 -1.966980 -0.943127 0.176378
S 0 -2.483445 -0.580569 -1.554023
C 0 -4.300495 -0.363068 -1.412960
C 0 -5.088803 -1.675155 -1.387538
C 0 -6.595517 -1.378122 -1.520078
O 0 -4.862508 -2.379939 -0.159796
O 0 -7.067532 -0.762997 -2.452619

O 0 -7.343097 -1.850278 -0.515657
O 0 -2.376044 3.545891 0.287425
O 0 -0.086919 3.985965 -1.585891
O 0 2.756244 2.952488 -1.763252
C 0 0.970683 1.639189 -2.768566
C 0 3.136951 0.147750 -1.071763
C 0 3.745071 -0.794087 -0.022886
C 0 4.825233 -1.713464 -0.607259
C 0 5.432058 -2.671204 0.425942
C 0 6.511124 -3.586628 -0.161987
H 0 1.161561 3.665479 0.031763
H 0 2.453563 1.572448 0.396190
H 0 -0.974506 -0.200655 1.911874
H 0 -5.009491 1.227014 2.716261
H 0 -3.757365 2.485990 2.957403
H 0 -3.412463 0.779807 3.393509
H 0 -2.836398 -1.310401 0.721636
H 0 -1.237091 -1.755757 0.123944
H 0 -4.599295 0.206286 -2.296725
H 0 -4.517656 0.235759 -0.525242
H 0 -4.804068 -2.291625 -2.246335
H 0 -4.570988 -3.282946 -0.360303
H 0 -6.714529 -2.293202 0.101149
H 0 -0.908850 4.260506 -1.135073
H 0 2.469733 3.722553 -2.282766
H 0 1.723519 1.073609 -3.323573
H 0 0.112869 0.988127 -2.574962
H 0 0.625628 2.467036 -3.393509
H 0 2.703944 -0.440303 -1.890064
H 0 3.919780 0.777474 -1.508356
H 0 2.950132 -1.404101 0.424974
H 0 4.176440 -0.197455 0.794023
H 0 4.396462 -2.298235 -1.434549
H 0 5.625539 -1.101295 -1.048892
H 0 4.631252 -3.283393 0.864545
H 0 5.858135 -2.087213 1.254047
H 0 6.106363 -4.205282 -0.972616
H 0 6.922078 -4.260506 0.598629
H 0 7.343097 -3.002886 -0.575144
Free Energy (PCM/B3LYP/6-31G*) = -1757.653145
Number of imaginary frequencies = 0

1-3*R*-c19
B3LYP/6-31G* geometry
O 0 -0.750725 -1.083447 -0.905551
C 0 -3.037791 -1.558049 -0.404991
C 0 -1.314260 -2.731651 0.906038
C 0 -2.781394 -2.790934 0.403979
C 0 -0.407347 -2.390542 -0.313844
C 0 -2.057732 -0.834636 -0.979069
C 0 -2.603751 0.352663 -1.733683
N 0 -4.003219 0.336820 -1.269807
C 0 -4.312822 -0.910275 -0.677475
O 0 -4.923212 0.802752 -2.228035
C 0 -5.754109 1.827752 -1.663302
C 0 -1.899885 1.699324 -1.534185
S 0 -1.802059 2.275383 0.213525
C 0 -0.052533 1.915842 0.644750
C 0 0.949963 2.889185 -0.001222
C 0 2.345065 2.598105 0.565372
O 0 0.651902 4.249858 0.272417
O 0 2.948471 1.572189 0.333753
O 0 2.831582 3.551561 1.369801
O 0 -5.445706 -1.305447 -0.416146
O 0 -3.665055 -2.910371 1.515340
O 0 -0.895150 -4.043500 1.294288
C 0 -1.152092 -1.752221 2.073631
C 0 1.088654 -2.355002 -0.029560
C 0 1.944986 -2.266896 -1.301071
C 0 3.443974 -2.145343 -0.999595
C 0 4.314211 -2.107994 -2.262414
C 0 5.810469 -1.975297 -1.960117
H 0 -2.866551 -3.689362 -0.227673
H 0 -0.617903 -3.132805 -1.094005
H 0 -2.588801 0.133855 -2.811924
H 0 -6.403863 2.143707 -2.482944
H 0 -5.155762 2.675546 -1.313120
H 0 -6.357379 1.429980 -0.842090
H 0 -2.448610 2.461129 -2.092952
H 0 -0.892724 1.650510 -1.956432
H 0 0.198413 0.889370 0.374804
H 0 -0.012977 2.011681 1.734443
H 0 0.997436 2.711824 -1.083571
H 0 -0.319285 4.348456 0.272475
H 0 2.164392 4.274668 1.385560
H 0 -4.562759 -2.752222 1.170103
H 0 -1.528854 -4.348456 1.965651
H 0 -0.118535 -1.755533 2.428708
H 0 -1.426500 -0.730765 1.792587
H 0 -1.803860 -2.055169 2.897242
H 0 1.312049 -1.505547 0.627572
H 0 1.337962 -3.264455 0.527825
H 0 1.625607 -1.403547 -1.898913
H 0 1.764055 -3.157902 -1.919623
H 0 3.618513 -1.233084 -0.411318
H 0 3.759662 -2.987762 -0.366136
H 0 3.993730 -1.269583 -2.897242
H 0 4.138503 -3.020263 -2.850011
H 0 6.020722 -1.055074 -1.401058
H 0 6.403863 -1.948928 -2.881419
H 0 6.168735 -2.818438 -1.356388
Free Energy (PCM/B3LYP/6-31G*) = -1757.653139
Number of imaginary frequencies = 0

1-3*R*-c20
B3LYP/6-31G* geometry
O 0 1.175152 -0.991402 -0.108126
C 0 0.494299 -3.202895 -0.695980
C 0 2.871532 -2.839051 -0.178429
C 0 1.868421 -3.740205 -0.945738
C 0 2.537943 -1.358875 -0.530880
C 0 0.256583 -1.931934 -0.317624
C 0 -1.211480 -1.681737 -0.059290
N 0 -1.739035 -3.056549 -0.167570
C 0 -0.770247 -3.915403 -0.740985
O 0 -3.018387 -3.135119 -0.761053
C 0 -3.885185 -3.957200 0.034556
C 0 -1.439883 -0.993411 1.292179
S 0 -3.185249 -0.708149 1.792045
C 0 -3.957348 0.137211 0.360075
C 0 -3.417227 1.545388 0.058001
C 0 -4.273412 2.221658 -1.012607
O 0 -2.077329 1.509043 -0.392587
O 0 -3.826586 2.542582 -2.097524
O 0 -5.540915 2.408099 -0.630473
O 0 -0.991634 -5.056476 -1.142058
O 0 2.009629 -5.093064 -0.520192
O 0 4.185701 -3.042493 -0.707065
C 0 2.854911 -3.113073 1.329827
C 0 3.474691 -0.319279 0.069738
C 0 3.254139 1.094782 -0.486348
C 0 4.218739 2.124420 0.115357
C 0 4.014676 3.542397 -0.433253
C 0 4.980514 4.566931 0.171014
H 0 2.126623 -3.666215 -2.014032
H 0 2.545027 -1.273166 -1.624927
H 0 -1.631820 -1.063773 -0.861111
H 0 -3.983785 -3.551310 1.045354
H 0 -4.849269 -3.924327 -0.479384
H 0 -3.516566 -4.986467 0.066563
H 0 -0.905112 -0.042538 1.281421
H 0 -1.035014 -1.614373 2.097524
H 0 -5.013887 0.200789 0.636933
H 0 -3.879628 -0.498274 -0.526993
H 0 -3.480087 2.153005 0.970560
H 0 -2.097675 1.816204 -1.320018
H 0 -6.023843 2.830351 -1.368021
H 0 1.229066 -5.568442 -0.859989
H 0 4.368341 -3.994385 -0.630074
H 0 3.627282 -2.519721 1.825632
H 0 1.887114 -2.872417 1.780186
H 0 3.056585 -4.171602 1.513139
H 0 3.354104 -0.312792 1.159882
H 0 4.499189 -0.646895 -0.138375

H 0 2.219643 1.407191 -0.294111
H 0 3.375384 1.076615 -1.579264
H 0 4.099845 2.138806 1.208958
H 0 5.254924 1.807372 -0.074891
H 0 2.979156 3.858125 -0.242360
H 0 4.132823 3.527746 -1.526065
H 0 4.861270 4.628918 1.259903
H 0 4.810069 5.568442 -0.240621
H 0 6.023843 4.295434 -0.032628
Free Energy (PCM/B3LYP/6-31G*) = -1757.65312
Number of imaginary frequencies = 0

1-3*R*-c21
B3LYP/6-31G* geometry
O 0 -0.502745 0.376571 -0.734576
C 0 0.702406 2.435057 -0.598326
C 0 -1.249111 2.152490 0.875602
C 0 -0.338985 3.190001 0.165288
C 0 -1.668664 1.087271 -0.181778
C 0 0.553808 1.150030 -0.973404
C 0 1.771136 0.633542 -1.703239
N 0 2.708646 1.745622 -1.461241
C 0 2.024507 2.891076 -1.006119
O 0 3.661162 1.940799 -2.473092
C 0 4.988740 1.863437 -1.928777
C 0 2.294158 -0.740241 -1.281216
S 0 2.795502 -0.777854 0.492366
C 0 3.312183 -2.531769 0.662161
C 0 4.727697 -2.843294 0.154976
C 0 5.783812 -1.935650 0.779284
O 0 4.804424 -2.736840 -1.251342
O 0 6.567632 -1.289679 0.109055
O 0 5.767424 -1.953087 2.115702
O 0 2.502525 4.021081 -0.930620
O 0 0.224933 4.077727 1.127649
O 0 -2.478434 2.785230 1.246707
C 0 -0.572484 1.537052 2.105120
C 0 -2.629947 0.018142 0.320408
C 0 -3.210079 -0.857021 -0.800037
C 0 -4.171303 -1.929780 -0.273480
C 0 -4.771703 -2.806119 -1.380449
C 0 -5.726458 -3.879329 -0.847454
H 0 -0.984271 3.761224 -0.520842
H 0 -2.120105 1.623911 -1.026008
H 0 1.553442 0.581946 -2.779989
H 0 5.650315 1.993097 -2.788704
H 0 5.170593 0.891836 -1.458024
H 0 5.157199 2.667229 -1.205707
H 0 3.150594 -1.012964 -1.898336
H 0 1.502862 -1.478995 -1.443087
H 0 2.597625 -3.174868 0.140340
H 0 3.250414 -2.757982 1.729792
H 0 4.965172 -3.873719 0.468877
H 0 5.589112 -2.185600 -1.436668
H 0 6.472755 -1.357955 2.437733
H 0 0.941106 4.556214 0.670752
H 0 -2.239578 3.544190 1.805511
H 0 0.313071 0.952875 1.837224
H 0 -0.259495 2.330508 2.788704
H 0 -1.275691 0.883835 2.628107
H 0 -2.113304 -0.612164 1.054298
H 0 -3.439958 0.530878 0.850646
H 0 -2.391602 -1.338185 -1.351033
H 0 -3.737138 -0.217279 -1.522734
H 0 -3.641788 -2.569562 0.447960
H 0 -4.984697 -1.445852 0.287497
H 0 -3.958190 -3.286285 -1.942642
H 0 -5.303287 -2.166527 -2.099309
H 0 -5.212668 -4.556214 -0.153620
H 0 -6.140072 -4.486440 -1.660991
H 0 -6.567632 -3.427516 -0.306998
Free Energy (PCM/B3LYP/6-31G*) = -1757.653085
Number of imaginary frequencies = 0

1-3*R*-c22
B3LYP/6-31G* geometry
O 0 1.813694 -0.775620 -0.595641
C 0 2.549928 -3.047960 -0.568585
C 0 3.997452 -1.364132 0.492317
C 0 3.968001 -2.759710 -0.185823
C 0 3.223084 -0.375837 -0.429712
C 0 1.624542 -2.084805 -0.739435
C 0 0.263296 -2.658564 -1.060132
N 0 0.519770 -4.093018 -0.827457
C 0 1.911600 -4.341629 -0.753413
O 0 -0.203358 -4.949166 -1.683700
C 0 -0.834974 -5.992311 -0.924539
C 0 -0.843832 -2.063116 -0.184688
S 0 -2.498726 -2.686259 -0.698090
C 0 -3.592716 -1.661685 0.357534
C 0 -3.685257 -0.192268 -0.099655
C 0 -4.773515 0.508286 0.722268
O 0 -4.036024 -0.067637 -1.469912
O 0 -4.657263 0.737489 1.906777
O 0 -5.881687 0.813399 0.034842
O 0 2.430023 -5.454531 -0.788859
O 0 4.503087 -3.736614 0.702613
O 0 5.340127 -0.870440 0.491121
C 0 3.445065 -1.408405 1.921595
C 0 3.211626 1.074677 0.033340
C 0 2.667387 2.046798 -1.023157
C 0 2.672944 3.504697 -0.546783
C 0 2.143734 4.489069 -1.597719
C 0 2.148793 5.943935 -1.117193
H 0 4.605438 -2.692814 -1.081526
H 0 3.671192 -0.441785 -1.429256
H 0 0.028732 -2.503284 -2.121906
H 0 -1.388580 -6.576951 -1.663274
H 0 -1.524460 -5.574330 -0.184767
H 0 -0.085044 -6.621492 -0.437343
H 0 -0.817582 -0.975326 -0.291257
H 0 -0.668535 -2.322502 0.863304
H 0 -3.273436 -1.709993 1.401517
H 0 -4.575471 -2.137173 0.276525
H 0 -2.740071 0.324946 0.109066
H 0 -3.621771 -0.808952 -1.952665
H 0 -5.718366 0.539887 -0.896372
H 0 4.288543 -4.605671 0.316952
H 0 5.881687 -1.544273 0.936198
H 0 3.543492 -0.427055 2.392512
H 0 2.391593 -1.703390 1.945677
H 0 4.009400 -2.135779 2.510908
H 0 2.619128 1.153729 0.952911
H 0 4.242198 1.341896 0.291202
H 0 1.645156 1.755863 -1.297591
H 0 3.271097 1.960435 -1.938240
H 0 2.068065 3.591262 0.367992
H 0 3.696735 3.791413 -0.264061
H 0 1.121657 4.200230 -1.881130
H 0 2.749533 4.402759 -2.510908
H 0 1.522650 6.067969 -0.224881
H 0 1.766873 6.621492 -1.889560
H 0 3.163269 6.271393 -0.857789
Free Energy (PCM/B3LYP/6-31G*) = -1757.653044
Number of imaginary frequencies = 0

1-3*R*-c23
B3LYP/6-31G* geometry
O 0 -0.285112 -0.438591 0.060624
C 0 1.184801 -2.282550 -0.316937
C 0 -0.580880 -1.860962 -1.984141
C 0 0.381591 -2.931668 -1.398721
C 0 -1.266345 -1.134516 -0.787603
C 0 0.826341 -1.137897 0.290618
C 0 1.844601 -0.696642 1.312563
N 0 2.742493 -1.867532 1.287662
C 0 2.490106 -2.695613 0.193681
O 0 4.058743 -1.692854 1.707824
C 0 4.284252 -2.389396 2.946078
C 0 2.527660 0.655407 1.022046
S 0 3.185002 0.927071 -0.669099
C 0 4.878381 0.198168 -0.582490
C 0 5.815304 0.961120 -1.521379
C 0 6.021707 2.415914 -1.105146

O 0 5.324289 0.926182 -2.846053
O 0 5.871996 3.343299 -1.877801
O 0 6.418777 2.548269 0.165366
O 0 3.221867 -3.594621 -0.224399
O 0 1.205148 -3.476376 -2.427833
O 0 -1.659137 -2.518286 -2.660193
C 0 0.138227 -0.898661 -2.934489
C 0 -2.321664 -0.103888 -1.167027
C 0 -3.123231 0.414567 0.035335
C 0 -4.198517 1.434380 -0.359854
C 0 -5.007320 1.956794 0.834270
C 0 -6.083672 2.972377 0.437270
H 0 -0.252228 -3.731420 -0.982706
H 0 -1.718106 -1.907329 -0.151629
H 0 1.375171 -0.609651 2.301830
H 0 5.324737 -2.177181 3.201438
H 0 4.142159 -3.466030 2.813461
H 0 3.621931 -2.016099 3.733942
H 0 3.319229 0.824137 1.755744
H 0 1.774147 1.436001 1.162366
H 0 4.851434 -0.852349 -0.880202
H 0 5.243356 0.254653 0.443844
H 0 6.803643 0.474910 -1.466623
H 0 5.379110 1.843746 -3.176003
H 0 6.549177 3.500075 0.344642
H 0 1.989270 -3.849367 -1.980519
H 0 -1.257582 -3.056472 -3.363014
H 0 0.911179 -0.318347 -2.421880
H 0 0.621167 -1.466797 -3.733942
H 0 -0.580958 -0.208413 -3.383351
H 0 -1.838028 0.735645 -1.681351
H 0 -2.995110 -0.578010 -1.889281
H 0 -2.438611 0.869428 0.762852
H 0 -3.598395 -0.435677 0.546294
H 0 -3.724675 2.283020 -0.875262
H 0 -4.882915 0.977442 -1.090124
H 0 -4.322335 2.415342 1.561637
H 0 -5.477443 1.108101 1.351192
H 0 -5.640135 3.849367 -0.050496
H 0 -6.642807 3.324740 1.311786
H 0 -6.803643 2.532562 -0.264120
Free Energy (PCM/B3LYP/6-31G*) = -1757.653042
Number of imaginary frequencies = 0

1-3*R*-c24
B3LYP/6-31G* geometry
O 0 1.407494 -0.717396 -0.687697
C 0 0.658676 -2.983397 -0.780715
C 0 2.744118 -2.517656 0.440797
C 0 1.941524 -3.603431 -0.323776
C 0 2.765440 -1.235050 -0.442911
C 0 0.493609 -1.654944 -0.926824
C 0 -0.915044 -1.302101 -1.346106
N 0 -1.570079 -2.617217 -1.198001
C 0 -0.603192 -3.642577 -1.077052
O 0 -2.592289 -2.861271 -2.137449
C 0 -3.778802 -3.319383 -1.470552
C 0 -1.526547 -0.189109 -0.490114
S 0 -3.167907 0.325573 -1.141307
C 0 -3.475810 1.756559 -0.023685
C 0 -4.979332 2.022438 0.089706
C 0 -5.736004 0.867617 0.744169
O 0 -5.534622 2.271607 -1.184984
O 0 -6.708655 0.351017 0.227993
O 0 -5.242707 0.524921 1.938867
O 0 -0.838617 -4.845731 -1.160188
O 0 1.731492 -4.731809 0.520894
O 0 4.116108 -2.916390 0.518623
C 0 2.180905 -2.267403 1.844663
C 0 3.580238 -0.075188 0.113484
C 0 3.782742 1.066203 -0.893380
C 0 4.635298 2.210017 -0.330286
C 0 4.855549 3.352301 -1.330417
C 0 5.715051 4.489034 -0.768032
H 0 2.555584 -3.904223 -1.187303
H 0 3.157083 -1.522394 -1.426970
H 0 -0.929885 -1.012345 -2.405685
H 0 -3.595483 -4.275931 -0.972919
H 0 -4.513550 -3.450084 -2.268832
H 0 -4.134601 -2.573976 -0.752883
H 0 -0.853715 0.673470 -0.514207
H 0 -1.625350 -0.531794 0.544386
H 0 -2.983066 2.648934 -0.419396
H 0 -3.063820 1.537733 0.964789
H 0 -5.112057 2.899869 0.743479
H 0 -6.327670 1.704699 -1.246536
H 0 -5.788860 -0.202773 2.295734
H 0 1.066272 -5.287412 0.074684
H 0 4.124278 -3.791729 0.941769
H 0 1.159582 -1.875418 1.814112
H 0 2.163092 -3.205320 2.405685
H 0 2.812741 -1.553236 2.378779
H 0 3.091435 0.307947 1.017568
H 0 4.551442 -0.478789 0.420059
H 0 2.806121 1.457568 -1.206285
H 0 4.262153 0.667450 -1.799254
H 0 4.157320 2.609441 0.576586
H 0 5.610963 1.813768 -0.011878
H 0 3.880317 3.750516 -1.644670
H 0 5.328031 2.950974 -2.238276
H 0 5.251226 4.934136 0.121107
H 0 5.854768 5.287412 -1.506021
H 0 6.708655 4.126486 -0.476543
Free Energy (PCM/B3LYP/6-31G*) = -1757.653021
Number of imaginary frequencies = 0

1-3*R*-c25
B3LYP/6-31G* geometry
O 0 -0.756794 -1.169630 -0.917268
C 0 -3.040775 -1.680445 -0.439260
C 0 -1.313257 -2.811316 0.900774
C 0 -2.773289 -2.900982 0.384136
C 0 -0.402152 -2.468245 -0.315457
C 0 -2.067199 -0.941979 -1.005631
C 0 -2.625563 0.234287 -1.768175
N 0 -4.031047 0.190648 -1.322603
C 0 -4.324243 -1.057239 -0.727484
O 0 -4.949939 0.640080 -2.289016
C 0 -5.791117 1.663888 -1.737527
C 0 -1.953192 1.594430 -1.551553
S 0 -1.887597 2.161853 0.200090
C 0 -0.113300 1.908581 0.598956
C 0 0.813489 3.033382 0.089900
C 0 2.259276 2.682003 0.436253
O 0 0.497268 4.288148 0.648295
O 0 2.889323 3.270145 1.293490
O 0 2.733264 1.649828 -0.275004
O 0 -5.452449 -1.472392 -0.473897
O 0 -3.668121 -3.023983 1.486416
O 0 -0.877232 -4.111811 1.309231
C 0 -1.177475 -1.814789 2.057136
C 0 1.090901 -2.419821 -0.019738
C 0 1.958567 -2.306254 -1.281126
C 0 3.459217 -2.265623 -0.966720
C 0 4.339786 -2.173605 -2.219323
C 0 5.838192 -2.131380 -1.902073
H 0 -2.836122 -3.807478 -0.238685
H 0 -0.599212 -3.218069 -1.092157
H 0 -2.593215 0.016433 -2.846274
H 0 -6.438581 1.968838 -2.563174
H 0 -5.200487 2.518530 -1.390942
H 0 -6.396235 1.268929 -0.916205
H 0 -2.507062 2.344245 -2.121853
H 0 -0.937941 1.566016 -1.956553
H 0 0.201336 0.937696 0.209874
H 0 -0.063024 1.873510 1.691934
H 0 0.732188 3.107540 -1.000909
H 0 1.123734 4.419651 1.384975
H 0 3.646322 1.469977 0.024135
H 0 -4.563141 -2.874936 1.129875
H 0 -1.515021 -4.419651 1.975392
H 0 -1.449341 -0.798494 1.755921
H 0 -1.842659 -2.109511 2.873006
H 0 -0.150312 -1.809403 2.430464

H 0 1.342670 -3.335980 0.525641
H 0 1.300908 -1.578848 0.652441
H 0 1.679512 -1.402973 -1.838623
H 0 1.747055 -3.158495 -1.943061
H 0 3.668984 -1.406852 -0.311709
H 0 3.736501 -3.162113 -0.392500
H 0 4.061355 -1.277426 -2.791923
H 0 4.127710 -3.031444 -2.873006
H 0 6.085610 -1.266393 -1.274031
H 0 6.438581 -2.061847 -2.816489
H 0 6.154395 -3.032953 -1.362823
Free Energy (PCM/B3LYP/6-31G*) = -1757.652893
Number of imaginary frequencies = 0

1-3*R*-c26
B3LYP/6-31G* geometry
O 0 0.925223 -0.675489 -0.128909
C 0 -0.065193 -2.796359 -0.599968
C 0 2.336600 -2.749345 -0.066762
C 0 1.217585 -3.541417 -0.793111
C 0 2.222765 -1.260203 -0.509100
C 0 -0.119465 -1.484129 -0.300417
C 0 -1.535636 -1.006612 -0.081133
N 0 -2.259457 -2.294450 -0.136202
C 0 -1.420918 -3.318271 -0.634734
O 0 -3.530833 -2.226936 -0.742232
C 0 -4.518134 -2.826789 0.106881
C 0 -1.686129 -0.259228 1.251335
S 0 -3.350000 0.445565 1.592918
C 0 -3.197735 2.190974 1.037898
C 0 -3.363780 2.454897 -0.461281
C 0 -4.641024 1.860892 -1.048892
O 0 -2.250844 1.964263 -1.180986
O 0 -4.644155 1.208697 -2.074822
O 0 -5.736777 2.177495 -0.349390
O 0 -1.801679 -4.436251 -0.977141
O 0 1.157510 -4.871014 -0.282469
O 0 3.609751 -3.171053 -0.566573
C 0 2.275851 -2.926388 1.454596
C 0 3.295851 -0.331906 0.044329
C 0 3.303465 1.053436 -0.617637
C 0 4.394055 1.973447 -0.055036
C 0 4.423252 3.356911 -0.717198
C 0 5.513650 4.273030 -0.152143
H 0 1.491761 -3.573806 -1.859544
H 0 2.249874 -1.242440 -1.606142
H 0 -1.863713 -0.361392 -0.902483
H 0 -4.306945 -3.889780 0.255915
H 0 -5.460636 -2.707827 -0.433605
H 0 -4.574920 -2.311845 1.071161
H 0 -0.956272 0.553552 1.294421
H 0 -1.474824 -0.947445 2.074822
H 0 -2.230492 2.586962 1.361030
H 0 -3.977117 2.729883 1.582793
H 0 -3.445149 3.548940 -0.584793
H 0 -2.602208 1.640626 -2.032369
H 0 -6.510014 1.786894 -0.802022
H 0 0.323725 -5.252543 -0.613992
H 0 3.648785 -4.134561 -0.442335
H 0 3.121575 -2.416809 1.923315
H 0 2.326898 -3.989375 1.704343
H 0 1.349484 -2.526659 1.878411
H 0 3.155777 -0.225824 1.126960
H 0 4.263046 -0.823374 -0.107979
H 0 2.322108 1.527807 -0.487516
H 0 3.449897 0.935062 -1.701100
H 0 4.246221 2.093421 1.028492
H 0 5.375668 1.491911 -0.177620
H 0 3.441821 3.836849 -0.595011
H 0 4.571042 3.236329 -1.799790
H 0 5.373419 4.438783 0.923295
H 0 5.507235 5.252543 -0.644128
H 0 6.510014 3.835661 -0.292451
Free Energy (PCM/B3LYP/6-31G*) = -1757.652891
Number of imaginary frequencies = 0

1-3*R*-c27
B3LYP/6-31G* geometry
O 0 -0.638643 -0.799052 -0.878377
C 0 -2.870083 -1.495021 -0.391017
C 0 -1.043684 -2.471079 0.948416
C 0 -2.500967 -2.672119 0.457347
C 0 -0.182357 -2.079337 -0.288227
C 0 -1.973161 -0.683488 -0.976686
C 0 -2.637688 0.406525 -1.785282
N 0 -4.033435 0.240760 -1.333537
C 0 -4.210790 -1.008193 -0.703556
O 0 -4.995944 0.597472 -2.291378
C 0 -5.908493 1.561995 -1.742994
C 0 -2.111719 1.833831 -1.626349
S 0 -2.200770 2.433596 0.119190
C 0 -0.592472 3.302655 0.300786
C 0 0.597359 2.372403 0.555262
C 0 1.847770 3.184172 0.934103
O 0 0.935509 1.583103 -0.584056
O 0 1.833174 4.080446 1.750648
O 0 2.967309 2.804809 0.305630
O 0 -5.292384 -1.524468 -0.440437
O 0 -3.366257 -2.832165 1.576944
O 0 -0.507640 -3.731028 1.361175
C 0 -0.957918 -1.452189 2.089805
C 0 1.311502 -1.941150 -0.029528
C 0 2.149314 -1.812799 -1.310513
C 0 3.648107 -1.660651 -1.022130
C 0 4.503372 -1.562579 -2.291767
C 0 5.998522 -1.399860 -1.999031
H 0 -2.509699 -3.592897 -0.146976
H 0 -0.354846 -2.833704 -1.065258
H 0 -2.580547 0.147356 -2.852996
H 0 -6.581651 1.809885 -2.566918
H 0 -5.378677 2.459701 -1.407756
H 0 -6.476475 1.129629 -0.914129
H 0 -2.703652 2.494247 -2.265207
H 0 -1.073862 1.879497 -1.960538
H 0 -0.714714 3.964092 1.161982
H 0 -0.409218 3.924673 -0.580336
H 0 0.372025 1.729491 1.415963
H 0 0.433642 0.744945 -0.568619
H 0 2.706419 2.081970 -0.309656
H 0 -4.275808 -2.796469 1.228575
H 0 -1.111648 -4.080446 2.038288
H 0 0.074264 -1.365289 2.438951
H 0 -1.313185 -0.462992 1.784856
H 0 -1.579802 -1.785698 2.924477
H 0 1.491897 -1.079156 0.624777
H 0 1.622272 -2.829734 0.529997
H 0 1.805558 -0.951514 -1.898246
H 0 1.984402 -2.700232 -1.937853
H 0 3.809568 -0.763834 -0.405694
H 0 3.991373 -2.512929 -0.417282
H 0 4.154626 -0.714690 -2.898337
H 0 4.345043 -2.461568 -2.903960
H 0 6.190269 -0.491667 -1.414159
H 0 6.581651 -1.330007 -2.924477
H 0 6.384320 -2.251154 -1.424514
Free Energy (PCM/B3LYP/6-31G*) = -1757.652866
Number of imaginary frequencies = 0

1-3*R*-c28
B3LYP/6-31G* geometry
O 0 0.976163 -0.587410 -0.211606
C 0 -0.074203 -2.699321 -0.588000
C 0 2.322552 -2.697103 -0.027757
C 0 1.188675 -3.490397 -0.729674
C 0 2.262757 -1.228400 -0.542996
C 0 -0.087651 -1.372526 -0.352180
C 0 -1.491413 -0.838398 -0.180555
N 0 -2.250058 -2.105779 -0.144275
C 0 -1.444613 -3.179460 -0.609788
O 0 -3.531483 -2.033016 -0.743472
C 0 -4.515333 -2.658667 0.105095
C 0 -1.648483 0.020161 1.080639
S 0 -3.335132 0.664957 1.414102
C 0 -3.528810 1.976029 0.130288

C 0 -4.517834 1.626622 -0.982971
C 0 -5.953669 1.451924 -0.451181
O 0 -4.155356 0.520204 -1.799478
O 0 -6.410985 2.068012 0.487303
O 0 -6.687812 0.577850 -1.156853
O 0 -1.868975 -4.291643 -0.907209
O 0 1.089288 -4.789597 -0.153421
O 0 3.586723 -3.182833 -0.488501
C 0 2.236310 -2.797776 1.499613
C 0 3.354780 -0.308755 -0.013757
C 0 3.408043 1.050923 -0.724924
C 0 4.520202 1.957311 -0.182700
C 0 4.591405 3.319694 -0.884232
C 0 5.703324 4.221151 -0.337796
H 0 1.471541 -3.581630 -1.789997
H 0 2.305180 -1.262460 -1.638900
H 0 -1.781919 -0.265161 -1.070535
H 0 -5.465892 -2.506194 -0.410810
H 0 -4.535583 -2.179740 1.087261
H 0 -4.300968 -3.725412 0.195358
H 0 -0.947321 0.858527 1.036917
H 0 -1.402127 -0.579279 1.961918
H 0 -2.549215 2.198586 -0.299986
H 0 -3.887317 2.872561 0.640178
H 0 -4.553187 2.487206 -1.664671
H 0 -3.980018 -0.270271 -1.240729
H 0 -6.091618 0.206416 -1.844829
H 0 0.270508 -5.185408 -0.502998
H 0 3.594313 -4.139231 -0.313170
H 0 1.321190 -2.343079 1.891150
H 0 2.244025 -3.848726 1.799760
H 0 3.095410 -2.298521 1.954593
H 0 3.208031 -0.159033 1.062768
H 0 4.308835 -0.832739 -0.137956
H 0 2.439971 1.557694 -0.620513
H 0 3.560433 0.890348 -1.802084
H 0 4.369181 2.113097 0.895821
H 0 5.488806 1.445732 -0.284504
H 0 3.622912 3.829614 -0.782135
H 0 4.742363 3.164197 -1.961918
H 0 5.560127 4.422632 0.731128
H 0 5.728161 5.185408 -0.858448
H 0 6.687812 3.751898 -0.457146
Free Energy (PCM/B3LYP/6-31G*) = -1757.65286
Number of imaginary frequencies = 0

1-3*R*-c29
B3LYP/6-31G* geometry
O 0 -0.757199 -1.168994 -0.917270
C 0 -3.041322 -1.679255 -0.439343
C 0 -1.314190 -2.810890 0.900464
C 0 -2.774181 -2.900145 0.383619
C 0 -0.402859 -2.467761 -0.315587
C 0 -2.067532 -0.940949 -1.005548
C 0 -2.625589 0.235700 -1.767753
N 0 -4.031043 0.192363 -1.322070
C 0 -4.324593 -1.055500 -0.727236
O 0 -4.949929 0.642600 -2.288082
C 0 -5.790584 1.666529 -1.735977
C 0 -1.952785 1.595573 -1.550838
S 0 -1.886750 2.162491 0.200961
C 0 -0.112428 1.908705 0.599392
C 0 0.814532 3.033402 0.090412
C 0 2.260314 2.681628 0.436374
O 0 0.498706 4.288111 0.649163
O 0 2.890727 3.269469 1.293539
O 0 2.733879 1.649488 -0.275236
O 0 -5.452905 -1.470285 -0.473401
O 0 -3.669249 -3.023478 1.485665
O 0 -0.878498 -4.111577 1.308690
C 0 -1.178336 -1.814644 2.057055
C 0 1.090150 -2.419579 -0.019621
C 0 1.958047 -2.305691 -1.280819
C 0 3.458640 -2.265203 -0.966112
C 0 4.339469 -2.172693 -2.218492
C 0 5.837818 -2.130737 -1.900919
H 0 -2.837011 -3.806395 -0.239568
H 0 -0.599896 -3.217436 -1.092433
H 0 -2.593408 0.018103 -2.845908
H 0 -6.438402 1.971751 -2.561244
H 0 -5.199565 2.520977 -1.389573
H 0 -6.395361 1.271564 -0.914415
H 0 -2.506499 2.345713 -2.120861
H 0 -0.937592 1.566961 -1.955966
H 0 0.201892 0.937836 0.210019
H 0 -0.061934 1.873385 1.692350
H 0 0.733056 3.107822 -1.000369
H 0 1.125503 4.419399 1.385601
H 0 3.646968 1.469342 0.023630
H 0 -4.564073 -2.872996 1.129209
H 0 -1.516382 -4.419399 1.974766
H 0 -1.449535 -0.798142 1.755930
H 0 -1.843976 -2.109176 2.872619
H 0 -0.151298 -1.809868 2.430735
H 0 1.341732 -3.335935 0.525517
H 0 1.300129 -1.578841 0.652861
H 0 1.679135 -1.402235 -1.838101
H 0 1.746623 -3.157728 -1.943046
H 0 3.668275 -1.406680 -0.310731
H 0 3.735804 -3.161908 -0.392171
H 0 4.061230 -1.276225 -2.790735
H 0 4.127453 -3.030209 -2.872619
H 0 6.085180 -1.266061 -1.272427
H 0 6.438402 -2.060831 -2.815177
H 0 6.153835 -3.032584 -1.362020
Free Energy (PCM/B3LYP/6-31G*) = -1757.652849
Number of imaginary frequencies = 0

1-3*R*-c30
B3LYP/6-31G* geometry
O 0 -0.844393 0.192390 -0.443441
C 0 0.574820 2.111112 -0.377010
C 0 -1.354882 2.066176 1.152011
C 0 -0.361556 2.986650 0.394432
C 0 -1.912675 1.029196 0.131985
C 0 0.280020 0.843555 -0.726659
C 0 1.414618 0.184189 -1.469729
N 0 2.477010 1.198752 -1.300647
C 0 1.916859 2.418987 -0.837565
O 0 3.297756 1.336139 -2.442160
C 0 4.680621 1.173729 -2.097393
C 0 1.788915 -1.229010 -1.004991
S 0 2.021358 -1.466465 0.805414
C 0 3.769002 -0.992716 1.111886
C 0 4.798538 -2.051260 0.689823
C 0 6.207901 -1.651800 1.126687
O 0 4.798715 -2.239419 -0.713321
O 0 7.129508 -1.552040 0.338690
O 0 6.313235 -1.446529 2.443474
O 0 2.508083 3.495508 -0.810716
O 0 0.317728 3.832295 1.319171
O 0 -2.502276 2.831839 1.536348
C 0 -0.713293 1.412190 2.380649
C 0 -2.959894 0.072389 0.686863
C 0 -3.671290 -0.748796 -0.397752
C 0 -4.711079 -1.717835 0.179210
C 0 -5.447362 -2.529340 -0.894225
C 0 -6.475292 -3.505359 -0.312340
H 0 -0.962249 3.606164 -0.290381
H 0 -2.334813 1.593319 -0.709274
H 0 1.167364 0.123521 -2.539763
H 0 4.993274 1.932788 -1.373652
H 0 5.219244 1.318100 -3.037546
H 0 4.876307 0.170503 -1.706587
H 0 2.686440 -1.568811 -1.522644
H 0 0.969457 -1.903278 -1.271904
H 0 3.824861 -0.847378 2.194139
H 0 3.975461 -0.037461 0.625418
H 0 4.559170 -2.998305 1.196109
H 0 5.730405 -2.158017 -0.996274
H 0 7.239770 -1.206668 2.642257
H 0 1.058726 4.232397 0.827997
H 0 -2.171037 3.569588 2.076005
H 0 0.102838 0.736259 2.107445

H 0 -0.306854 2.185804 3.037546
H 0 -1.465878 0.845305 2.934921
H 0 -2.481938 -0.600041 1.409622
H 0 -3.690626 0.672400 1.240154
H 0 -2.929046 -1.313051 -0.977018
H 0 -4.162660 -0.064432 -1.104569
H 0 -4.215728 -2.407474 0.878692
H 0 -5.444749 -1.154055 0.774362
H 0 -4.713018 -3.085234 -1.494418
H 0 -5.948941 -1.839622 -1.587921
H 0 -5.996680 -4.232397 0.355447
H 0 -6.986996 -4.065699 -1.103374
H 0 -7.239770 -2.975071 0.269022
Free Energy (PCM/B3LYP/6-31G*) = -1757.652816
Number of imaginary frequencies = 0

1-3*R*-c31
B3LYP/6-31G* geometry
O 0 -0.757349 -1.169115 -0.917961
C 0 -3.041493 -1.679096 -0.439777
C 0 -1.314420 -2.810745 0.900052
C 0 -2.774437 -2.899938 0.383248
C 0 -0.403022 -2.467815 -0.315973
C 0 -2.067675 -0.940953 -1.006117
C 0 -2.625667 0.235753 -1.768318
N 0 -4.031092 0.192439 -1.322686
C 0 -4.324754 -1.055341 -0.727816
O 0 -4.949960 0.642956 -2.288550
C 0 -5.790371 1.666963 -1.736228
C 0 -1.952872 1.595586 -1.551175
S 0 -1.886839 2.162069 0.200785
C 0 -0.112491 1.908424 0.599019
C 0 0.814438 3.033188 0.090144
C 0 2.260252 2.681334 0.435880
O 0 0.498676 4.287764 0.649275
O 0 2.890793 3.269046 1.293045
O 0 2.733726 1.649336 -0.275981
O 0 -5.453072 -1.470107 -0.474021
O 0 -3.669472 -3.023068 1.485344
O 0 -0.878799 -4.111431 1.308371
C 0 -1.178559 -1.814423 2.056582
C 0 1.089972 -2.419389 -0.019915
C 0 1.957912 -2.305841 -1.281116
C 0 3.458475 -2.264720 -0.966376
C 0 4.339272 -2.172938 -2.218846
C 0 5.837605 -2.130117 -1.901350
H 0 -2.837406 -3.806240 -0.239847
H 0 -0.599941 -3.217618 -1.092717
H 0 -2.593404 0.018155 -2.846479
H 0 -6.438128 1.972545 -2.561410
H 0 -5.199122 2.521174 -1.389634
H 0 -6.395236 1.271964 -0.914746
H 0 -2.506637 2.345836 -2.121003
H 0 -0.937683 1.567118 -1.956318
H 0 0.201833 0.937557 0.209650
H 0 -0.061934 1.873071 1.691981
H 0 0.732869 3.107876 -1.000615
H 0 1.125733 4.418938 1.385517
H 0 3.646868 1.469169 0.022705
H 0 -4.564315 -2.872825 1.128838
H 0 -1.516382 -4.418938 1.974881
H 0 -1.449898 -0.797971 1.755397
H 0 -1.844131 -2.108964 2.872204
H 0 -0.151524 -1.809507 2.430260
H 0 1.341661 -3.335504 0.525577
H 0 1.299796 -1.578358 0.652241
H 0 1.678771 -1.402724 -1.838825
H 0 1.746766 -3.158252 -1.942947
H 0 3.667854 -1.405634 -0.311655
H 0 3.735881 -3.160910 -0.391756
H 0 4.060670 -1.277076 -2.791857
H 0 4.127575 -3.031115 -2.872204
H 0 6.084675 -1.264716 -1.273746
H 0 6.438128 -2.060919 -2.815703
H 0 6.153968 -3.031305 -1.361554
Free Energy (PCM/B3LYP/6-31G*) = -1757.6528
Number of imaginary frequencies = 0

1-3*R*-c32
B3LYP/6-31G* geometry
O 0 -0.745672 -1.170394 -0.830779
C 0 -2.995375 -1.929951 -0.608175
C 0 -1.260865 -3.090114 0.698567
C 0 -2.672471 -3.222998 0.072030
C 0 -0.318261 -2.512526 -0.397912
C 0 -2.062808 -1.035520 -0.991866
C 0 -2.686762 0.194178 -1.614729
N 0 -4.111547 -0.062907 -1.315855
C 0 -4.313985 -1.399015 -0.917878
O 0 -5.003211 0.447276 -2.275258
C 0 -6.002722 1.261359 -1.642729
C 0 -2.263793 1.567908 -1.066767
S 0 -0.642512 2.186501 -1.684642
C 0 0.512691 1.795783 -0.303231
C 0 0.889488 3.017895 0.548331
C 0 -0.315558 3.672275 1.214316
O 0 1.570836 3.979125 -0.227510
O 0 -0.600867 4.842509 1.051761
O 0 -1.005653 2.829214 1.995211
O 0 -5.407182 -1.951518 -0.813001
O 0 -3.620992 -3.557883 1.081850
O 0 -0.740100 -4.396338 0.964538
C 0 -1.272779 -2.251185 1.981471
C 0 1.148168 -2.401506 -0.002982
C 0 2.079336 -2.102597 -1.186575
C 0 3.552052 -2.001070 -0.770550
C 0 4.499202 -1.724677 -1.945167
C 0 5.968632 -1.622662 -1.523423
H 0 -2.619005 -4.042331 -0.662162
H 0 -0.413773 -3.155421 -1.282227
H 0 -2.542120 0.176608 -2.703023
H 0 -6.618799 0.661963 -0.966268
H 0 -6.613985 1.642876 -2.463996
H 0 -5.547480 2.094195 -1.096551
H 0 -2.273269 1.576519 0.025522
H 0 -3.002995 2.295514 -1.411255
H 0 1.429601 1.415774 -0.760740
H 0 0.090872 0.997058 0.308095
H 0 1.548126 2.659289 1.355521
H 0 1.048204 4.800651 -0.160012
H 0 -1.751527 3.322571 2.390252
H 0 -4.500609 -3.446092 0.676261
H 0 -1.392577 -4.842509 1.530731
H 0 -1.593799 -1.221025 1.798514
H 0 -1.964529 -2.693583 2.703023
H 0 -0.273506 -2.232172 2.423912
H 0 1.256733 -1.625060 0.764225
H 0 1.431892 -3.352125 0.461522
H 0 1.770541 -1.167619 -1.671873
H 0 1.968117 -2.894573 -1.941403
H 0 3.664058 -1.204935 -0.019558
H 0 3.854550 -2.934571 -0.273044
H 0 4.195406 -0.793146 -2.443500
H 0 4.388136 -2.521643 -2.694166
H 0 6.118200 -0.809865 -0.801871
H 0 6.618799 -1.428232 -2.384219
H 0 6.309922 -2.552050 -1.050637
Free Energy (PCM/B3LYP/6-31G*) = -1757.652773
Number of imaginary frequencies = 0

1-3*R*-c33
B3LYP/6-31G* geometry
O 0 1.288527 -0.730715 -1.467377
C 0 2.223101 -2.515817 -0.165808
C 0 3.755183 -1.083358 -1.461136
C 0 3.658479 -2.109447 -0.297687
C 0 2.600215 -0.044417 -1.417652
C 0 1.200907 -1.842520 -0.740575
C 0 -0.135160 -2.498392 -0.470101
N 0 0.270699 -3.504799 0.528791
C 0 1.673640 -3.679438 0.530427
O 0 -0.492559 -4.686219 0.483857
C 0 -1.011695 -4.991202 1.785970
C 0 -1.233934 -1.573862 0.078086

S 0 -2.060889 -0.542833 -1.203667
C 0 -3.421186 -1.652616 -1.750528
C 0 -4.667148 -1.651287 -0.850666
C 0 -5.239021 -0.250127 -0.656169
O 0 -4.397943 -2.217169 0.415579
O 0 -5.424175 0.232432 0.444659
O 0 -5.522349 0.363967 -1.809771
O 0 2.259090 -4.618567 1.054334
O 0 4.177126 -1.526083 0.903890
O 0 3.660075 -1.805521 -2.698514
C 0 5.118577 -0.398218 -1.504289
C 0 2.587637 0.948187 -0.253555
C 0 1.504811 2.028076 -0.389943
C 0 1.522540 3.036838 0.765595
C 0 0.450703 4.126611 0.635379
C 0 0.464650 5.128785 1.794256
H 0 4.272066 -2.975276 -0.582985
H 0 2.621265 0.493660 -2.369034
H 0 -0.495504 -3.016483 -1.368702
H 0 -1.612523 -5.892908 1.644601
H 0 -0.199283 -5.192435 2.490576
H 0 -1.641719 -4.178146 2.162033
H 0 -0.819391 -0.888786 0.822614
H 0 -2.008007 -2.172103 0.559325
H 0 -3.045428 -2.676288 -1.833595
H 0 -3.695728 -1.317136 -2.753933
H 0 -5.439104 -2.245715 -1.365811
H 0 -4.662758 -1.544055 1.072207
H 0 -5.884742 1.248065 -1.604174
H 0 4.072354 -2.186946 1.608070
H 0 2.790251 -2.235931 -2.741541
H 0 5.884742 -1.151811 -1.712396
H 0 5.357811 0.084008 -0.556315
H 0 5.143652 0.346195 -2.306946
H 0 2.476573 0.404069 0.690768
H 0 3.570886 1.431550 -0.215123
H 0 1.644348 2.561700 -1.341562
H 0 0.515856 1.555510 -0.443768
H 0 1.382570 2.502625 1.717067
H 0 2.514462 3.509233 0.823849
H 0 -0.539438 3.653283 0.572221
H 0 0.593793 4.662349 -0.313815
H 0 0.289760 4.626665 2.753933
H 0 -0.311664 5.892908 1.671547
H 0 1.431385 5.643282 1.861412
Free Energy (PCM/B3LYP/6-31G*) = -1757.652769
Number of imaginary frequencies = 0

1-3*R*-c34
B3LYP/6-31G* geometry
O 0 0.726315 -0.852542 -0.849016
C 0 0.055004 -3.120989 -0.517610
C 0 1.867883 -2.246938 0.899661
C 0 1.244482 -3.532106 0.291813
C 0 2.024311 -1.206879 -0.249336
C 0 -0.109009 -1.875825 -1.004898
C 0 -1.411346 -1.730832 -1.754232
N 0 -2.065035 -3.002038 -1.388229
C 0 -1.121226 -3.909397 -0.851346
O 0 -2.917167 -3.517491 -2.380642
C 0 -4.229486 -3.736860 -1.841695
C 0 -2.237948 -0.483651 -1.432684
S 0 -2.794205 -0.464601 0.323980
C 0 -2.931214 1.340035 0.607158
C 0 -4.146963 2.005819 -0.077954
C 0 -4.122430 3.504694 0.215637
O 0 -5.375279 1.471003 0.357504
O 0 -4.916181 4.037959 0.966591
O 0 -3.124670 4.143265 -0.408531
O 0 -1.320178 -5.107965 -0.669777
O 0 0.908259 -4.444589 1.333501
O 0 3.211623 -2.525138 1.307046
C 0 1.048479 -1.715831 2.081277
C 0 2.691438 0.103408 0.147209
C 0 3.052269 0.991927 -1.052132
C 0 3.731611 2.302215 -0.635318
C 0 4.107678 3.197333 -1.823247
C 0 4.784006 4.505690 -1.401118
H 0 2.014348 -3.984247 -0.353437
H 0 2.601551 -1.688000 -1.049306
H 0 -1.213504 -1.732882 -2.835815
H 0 -4.204474 -4.500580 -1.058600
H 0 -4.824303 -4.090447 -2.687295
H 0 -4.653954 -2.808227 -1.446206
H 0 -3.100435 -0.445975 -2.103166
H 0 -1.619365 0.399803 -1.618868
H 0 -2.002414 1.827671 0.296057
H 0 -3.028330 1.452135 1.691958
H 0 -4.072421 1.868920 -1.162889
H 0 -5.708819 2.085733 1.038194
H 0 -3.152032 5.083739 -0.143225
H 0 0.334110 -5.121436 0.930193
H 0 3.161593 -3.267586 1.932769
H 0 0.044456 -1.403271 1.777504
H 0 0.942556 -2.499824 2.835815
H 0 1.557762 -0.862387 2.536493
H 0 2.030343 0.650860 0.830013
H 0 3.596777 -0.149339 0.709847
H 0 2.145276 1.217721 -1.627861
H 0 3.717986 0.434583 -1.727132
H 0 3.065395 2.858620 0.040601
H 0 4.636396 2.074034 -0.052462
H 0 3.203401 3.423574 -2.405890
H 0 4.774291 2.641391 -2.497688
H 0 4.126902 5.100099 -0.754095
H 0 5.041815 5.121436 -2.270532
H 0 5.708819 4.310923 -0.843831
Free Energy (PCM/B3LYP/6-31G*) = -1757.652768
Number of imaginary frequencies = 0

1-3*R*-c35
B3LYP/6-31G* geometry
O 0 1.800549 -0.699741 -0.593667
C 0 2.650774 -2.931062 -0.538808
C 0 3.999895 -1.167213 0.521151
C 0 4.048610 -2.567820 -0.145866
C 0 3.185554 -0.227424 -0.417104
C 0 1.678662 -2.018234 -0.726316
C 0 0.349946 -2.663273 -1.047410
N 0 0.678309 -4.079889 -0.792711
C 0 2.078995 -4.257599 -0.707343
O 0 0.001963 -4.987411 -1.633231
C 0 -0.586986 -6.039930 -0.853395
C 0 -0.793491 -2.111330 -0.190895
S 0 -2.415626 -2.812651 -0.704046
C 0 -3.536258 -1.775858 0.310757
C 0 -3.829647 -0.385213 -0.294251
C 0 -4.718246 0.401498 0.667161
O 0 -4.467935 -0.470463 -1.547766
O 0 -5.886489 0.641916 0.430958
O 0 -4.084214 0.758497 1.791012
O 0 2.653456 -5.343846 -0.721133
O 0 4.623912 -3.510379 0.754686
O 0 5.315493 -0.604798 0.528852
C 0 3.435921 -1.228320 1.945189
C 0 3.094392 1.224649 0.032513
C 0 2.509614 2.157115 -1.038043
C 0 2.431386 3.617288 -0.575240
C 0 1.857103 4.561339 -1.639406
C 0 1.777700 6.018624 -1.173030
H 0 4.690843 -2.474632 -1.035822
H 0 3.647461 -0.278420 -1.411238
H 0 0.116113 -2.536015 -2.113089
H 0 -1.299366 -5.635648 -0.127948
H 0 0.187418 -6.622954 -0.347173
H 0 -1.108894 -6.666333 -1.580947
H 0 -0.817255 -1.024927 -0.318286
H 0 -0.608550 -2.338289 0.863519
H 0 -3.120917 -1.680836 1.318421
H 0 -4.472823 -2.337681 0.381171
H 0 -2.890082 0.166354 -0.416904
H 0 -5.416970 -0.320905 -1.375514
H 0 -4.723316 1.222913 2.366743
H 0 4.442596 -4.392482 0.381271

H 0 5.886489 -1.245605 0.985725
H 0 2.399945 -1.580157 1.961334
H 0 4.032857 -1.918229 2.547277
H 0 3.475869 -0.238740 2.407331
H 0 2.489714 1.280823 0.945886
H 0 4.106934 1.548304 0.297173
H 0 1.507464 1.809039 -1.319837
H 0 3.126408 2.095422 -1.946385
H 0 1.815136 3.678673 0.334019
H 0 3.435458 3.962694 -0.287371
H 0 0.854895 4.213628 -1.927632
H 0 2.473894 4.500326 -2.547277
H 0 1.139863 6.115880 -0.285665
H 0 1.363342 6.666333 -1.954233
H 0 2.770517 6.405053 -0.910968
Free Energy (PCM/B3LYP/6-31G*) = -1757.652749
Number of imaginary frequencies = 0

1-3*R*-c36
B3LYP/6-31G* geometry
O 0 -0.757560 -1.168003 -0.917514
C 0 -3.041824 -1.677697 -0.439700
C 0 -1.315033 -2.810003 0.899987
C 0 -2.775053 -2.898786 0.383087
C 0 -0.403546 -2.466908 -0.315945
C 0 -2.067814 -0.939559 -1.005742
C 0 -2.625495 0.237426 -1.767697
N 0 -4.030944 0.194453 -1.321959
C 0 -4.324893 -1.053448 -0.727405
O 0 -4.949692 0.645212 -2.287874
C 0 -5.790123 1.669129 -1.735432
C 0 -1.952265 1.597041 -1.550533
S 0 -1.885711 2.163435 0.201409
C 0 -0.111395 1.908858 0.599369
C 0 0.815808 3.033571 0.090856
C 0 2.261592 2.680982 0.435981
O 0 0.500660 4.287995 0.650640
O 0 2.892504 3.267765 1.293503
O 0 2.734587 1.649410 -0.276829
O 0 -5.453334 -1.467915 -0.473626
O 0 -3.670224 -3.021971 1.485070
O 0 -0.879652 -4.110880 1.307971
C 0 -1.178940 -1.813983 2.056744
C 0 1.089450 -2.418989 -0.019831
C 0 1.957442 -2.305133 -1.280961
C 0 3.457982 -2.264248 -0.966076
C 0 4.338931 -2.171866 -2.218377
C 0 5.837219 -2.129390 -1.900610
H 0 -2.838127 -3.804943 -0.240203
H 0 -0.600660 -3.216457 -1.092895
H 0 -2.593427 0.020023 -2.845895
H 0 -6.394958 1.274028 -0.913978
H 0 -6.437900 1.974734 -2.560590
H 0 -5.198924 2.523351 -1.388777
H 0 -2.505879 2.347496 -2.120241
H 0 -0.937159 1.568265 -1.955870
H 0 0.202523 0.938113 0.209369
H 0 -0.060693 1.872901 1.692295
H 0 0.733897 3.108858 -0.999830
H 0 1.127522 4.418357 1.387185
H 0 3.647727 1.468673 0.021518
H 0 -4.564982 -2.871132 1.128595
H 0 -1.517094 -4.418357 1.974627
H 0 -1.449896 -0.797377 1.755754
H 0 -1.844662 -2.108452 2.872264
H 0 -0.151900 -1.809498 2.430424
H 0 1.299469 -1.578295 0.652694
H 0 1.340873 -3.335397 0.525286
H 0 1.678397 -1.401824 -1.838417
H 0 1.746262 -3.157328 -1.943058
H 0 3.667327 -1.405505 -0.310888
H 0 3.735273 -3.160743 -0.391871
H 0 4.060512 -1.275652 -2.790928
H 0 4.127244 -3.029645 -2.872264
H 0 6.084235 -1.264482 -1.272301
H 0 6.437900 -2.059537 -2.814807
H 0 6.153426 -3.031008 -1.361439
Free Energy (PCM/B3LYP/6-31G*) = -1757.65274
Number of imaginary frequencies = 0

1-3*R*-c37
B3LYP/6-31G* geometry
O 0 1.929907 -1.224010 -0.263255
C 0 1.851114 -3.602937 -0.423354
C 0 3.913292 -2.578906 0.454066
C 0 3.313853 -3.841226 -0.223072
C 0 3.396589 -1.335122 -0.328906
C 0 1.285349 -2.381562 -0.405948
C 0 -0.215054 -2.453232 -0.576693
N 0 -0.355838 -3.893976 -0.907970
C 0 0.808692 -4.607138 -0.598766
O 0 -1.577990 -4.503858 -0.618510
C 0 -2.262297 -4.845533 -1.835928
C 0 -1.016048 -2.076823 0.687103
S 0 -1.106165 -0.270335 0.997705
C 0 -2.502916 0.235127 -0.081603
C 0 -3.879153 -0.194670 0.461800
C 0 -4.969028 0.436785 -0.412346
O 0 -4.098913 0.235159 1.796492
O 0 -5.157190 0.110386 -1.564344
O 0 -5.673408 1.406437 0.185355
O 0 0.910960 -5.831484 -0.522983
O 0 3.565759 -4.995980 0.575060
O 0 5.329993 -2.563064 0.249363
C 0 3.590839 -2.519572 1.951159
C 0 3.953640 0.004475 0.134346
C 0 3.607028 1.166099 -0.807894
C 0 4.171107 2.508749 -0.326535
C 0 3.842756 3.677663 -1.264216
C 0 4.401637 5.017958 -0.775228
H 0 3.823851 -3.953271 -1.193269
H 0 3.638890 -1.490170 -1.388495
H 0 -0.543619 -1.841752 -1.424502
H 0 -3.202737 -5.296345 -1.511362
H 0 -1.679989 -5.567921 -2.415777
H 0 -2.463756 -3.954125 -2.438993
H 0 -0.553593 -2.518705 1.573905
H 0 -2.023955 -2.486932 0.603627
H 0 -2.368060 -0.142914 -1.097757
H 0 -2.440849 1.327413 -0.113826
H 0 -3.990666 -1.283598 0.378705
H 0 -3.251944 0.164406 2.276989
H 0 -5.340667 1.469405 1.109140
H 0 2.925731 -5.673082 0.282260
H 0 5.673408 -3.392109 0.623263
H 0 4.089997 -1.661065 2.407691
H 0 2.515701 -2.437594 2.137123
H 0 3.945747 -3.431051 2.438993
H 0 3.579288 0.219084 1.142715
H 0 5.041026 -0.102846 0.211481
H 0 2.516677 1.242890 -0.908378
H 0 3.997059 0.945512 -1.812195
H 0 3.778916 2.728535 0.677474
H 0 5.262809 2.427523 -0.216877
H 0 2.752043 3.754895 -1.377127
H 0 4.238611 3.459531 -2.266241
H 0 3.996165 5.279781 0.209977
H 0 4.151370 5.831484 -1.465928
H 0 5.494419 4.981535 -0.684403
Free Energy (PCM/B3LYP/6-31G*) = -1757.652698
Number of imaginary frequencies = 0

1-3*R*-c38
B3LYP/6-31G* geometry
O 0 -1.290649 -0.503718 0.010951
C 0 -3.609974 -0.861394 -0.427166
C 0 -2.259233 -2.800835 0.262529
C 0 -3.551210 -2.354415 -0.471778
C 0 -1.084251 -1.937897 -0.287230
C 0 -2.550008 -0.076263 -0.170900
C 0 -2.920291 1.388466 -0.151100
N 0 -4.304876 1.292694 -0.684540
C 0 -4.787365 -0.018457 -0.619783
O 0 -5.192070 2.317935 -0.350202

C 0 -5.513548 3.093449 -1.517262
C 0 -2.919050 2.051557 1.243756
S 0 -1.318110 2.134917 2.132443
C 0 -0.339912 3.347156 1.152370
C 0 0.746490 2.674396 0.304486
C 0 1.636943 3.729007 -0.370864
O 0 0.218272 1.849809 -0.728761
O 0 2.089769 4.686582 0.218817
O 0 1.897774 3.497961 -1.664084
O 0 -5.960161 -0.370328 -0.739430
O 0 -4.693039 -2.955245 0.134050
O 0 -1.930319 -4.135139 -0.135784
C 0 -2.399700 -2.718725 1.786226
C 0 0.293058 -2.309992 0.247061
C 0 1.459432 -1.735181 -0.570029
C 0 2.827495 -2.155056 -0.017642
C 0 4.006840 -1.606129 -0.830478
C 0 5.369922 -2.032560 -0.275859
H 0 -3.463892 -2.705478 -1.512410
H 0 -1.098184 -2.019045 -1.381280
H 0 -2.286442 1.958207 -0.837496
H 0 -6.190951 3.871518 -1.158559
H 0 -6.016227 2.473799 -2.265955
H 0 -4.615925 3.549161 -1.948130
H 0 -3.570487 1.483081 1.914734
H 0 -3.334216 3.057147 1.152750
H 0 0.135560 4.030344 1.859332
H 0 -1.018151 3.929018 0.521317
H 0 1.404435 2.091331 0.963049
H 0 -0.055341 0.986190 -0.362797
H 0 1.412735 2.678535 -1.908983
H 0 -5.464162 -2.437000 -0.166095
H 0 -2.701970 -4.686582 0.077805
H 0 -2.578810 -1.695323 2.129142
H 0 -3.245038 -3.332034 2.108651
H 0 -1.492266 -3.094321 2.265955
H 0 0.369316 -1.986423 1.292291
H 0 0.353372 -3.403513 0.244924
H 0 1.404340 -0.638952 -0.596742
H 0 1.368161 -2.071761 -1.612686
H 0 2.917878 -1.818276 1.025650
H 0 2.884890 -3.253235 0.009646
H 0 3.948876 -0.508675 -0.856417
H 0 3.914487 -1.941628 -1.873173
H 0 5.505529 -1.681563 0.754728
H 0 6.190951 -1.625780 -0.877374
H 0 5.470187 -3.125040 -0.269354
Free Energy (PCM/B3LYP/6-31G*) = -1757.652691
Number of imaginary frequencies = 0

1-3*R*-c39
B3LYP/6-31G* geometry
O 0 1.083747 -0.046731 -0.444680
C 0 2.837258 -1.647438 -0.680870
C 0 3.439056 0.593086 0.140471
C 0 3.968559 -0.673109 -0.581787
C 0 2.106851 1.008514 -0.551933
C 0 1.541107 -1.285793 -0.606911
C 0 0.618188 -2.480767 -0.679470
N 0 1.604529 -3.573997 -0.564971
C 0 2.914251 -3.095812 -0.786165
O 0 1.246064 -4.745877 -1.254148
C 0 1.301612 -5.876899 -0.371425
C 0 -0.454728 -2.596900 0.412304
S 0 -1.825694 -1.395140 0.151577
C 0 -3.111869 -2.182988 1.210583
C 0 -4.510325 -1.781565 0.733765
C 0 -4.832559 -2.302638 -0.665814
O 0 -4.653408 -0.376608 0.746412
O 0 -5.240046 -1.577558 -1.553088
O 0 -4.659514 -3.623099 -0.787855
O 0 3.903647 -3.801674 -0.968807
O 0 5.083017 -1.204513 0.130372
O 0 4.326402 1.686088 -0.116303
C 0 3.285302 0.374170 1.649755
C 0 1.469307 2.286380 -0.022772
C 0 0.322347 2.805873 -0.901282
C 0 -0.304880 4.095040 -0.356333
C 0 -1.438090 4.638908 -1.235790
C 0 -2.066335 5.922869 -0.684219
H 0 4.295258 -0.354669 -1.584336
H 0 2.314812 1.115325 -1.624179
H 0 0.142926 -2.530322 -1.668617
H 0 2.324367 -6.045669 -0.022057
H 0 0.971241 -6.722848 -0.978851
H 0 0.630032 -5.744700 0.483422
H 0 -0.004573 -2.460052 1.399690
H 0 -0.872436 -3.605940 0.355936
H 0 -2.984265 -1.862441 2.247918
H 0 -3.004702 -3.269715 1.163617
H 0 -5.237323 -2.248032 1.419045
H 0 -5.050616 -0.140189 -0.113995
H 0 -4.903844 -3.878818 -1.698906
H 0 5.240046 -2.095538 -0.233235
H 0 5.207812 1.400686 0.178650
H 0 2.550736 -0.403662 1.879732
H 0 4.243668 0.066374 2.075996
H 0 2.971760 1.303508 2.131965
H 0 1.106597 2.110037 0.997143
H 0 2.260301 3.041503 0.042919
H 0 -0.451947 2.032860 -0.989648
H 0 0.700029 2.985897 -1.918338
H 0 -0.689655 3.911893 0.657853
H 0 0.475099 4.864132 -0.252822
H 0 -2.214499 3.868086 -1.342919
H 0 -1.051954 4.825744 -2.247918
H 0 -2.492664 5.758050 0.313167
H 0 -2.870520 6.285927 -1.334652
H 0 -1.320519 6.722848 -0.597352
Free Energy (PCM/B3LYP/6-31G*) = -1757.652662
Number of imaginary frequencies = 0

1-3*R*-c40
B3LYP/6-31G* geometry
O 0 2.064271 -1.150059 -0.551449
C 0 1.843583 -3.520832 -0.746011
C 0 3.929620 -2.643148 0.219630
C 0 3.289256 -3.841539 -0.529922
C 0 3.526149 -1.341684 -0.535287
C 0 1.361725 -2.263486 -0.744495
C 0 -0.138892 -2.232915 -0.924598
N 0 -0.447548 -3.670722 -0.802490
C 0 0.732304 -4.442973 -0.921001
O 0 -1.532267 -4.093139 -1.597742
C 0 -2.446024 -4.877842 -0.814462
C 0 -0.835109 -1.345425 0.110837
S 0 -2.642355 -1.227031 -0.222048
C 0 -3.097424 0.138675 0.936224
C 0 -4.052368 1.127052 0.260457
C 0 -5.448960 0.516903 0.023143
O 0 -3.540524 1.676695 -0.949763
O 0 -6.036511 -0.149715 0.848124
O 0 -5.980250 0.822524 -1.167202
O 0 0.763450 -5.659958 -1.084383
O 0 3.476962 -5.035288 0.225033
O 0 5.351767 -2.707804 0.078022
C 0 3.541065 -2.618507 1.702423
C 0 4.125645 -0.056391 0.019409
C 0 3.923656 1.157563 -0.898639
C 0 4.532516 2.442377 -0.323160
C 0 4.358199 3.661261 -1.238207
C 0 4.959729 4.943731 -0.654075
H 0 3.814443 -3.932803 -1.493666
H 0 3.822818 -1.465965 -1.584522
H 0 -0.391027 -1.895932 -1.939230
H 0 -3.260198 -5.122999 -1.500734
H 0 -2.830825 -4.300451 0.031654
H 0 -1.963567 -5.794959 -0.465373
H 0 -0.405724 -0.341287 0.055905
H 0 -0.672878 -1.750397 1.113299
H 0 -2.182604 0.662000 1.224203
H 0 -3.571917 -0.269633 1.830142
H 0 -4.201584 1.969684 0.946576
H 0 -3.029484 0.964862 -1.386809

H 0 -5.297989 1.351718 -1.641162
H 0 2.897946 -5.708737 -0.176622
H 0 5.621999 -3.578587 0.415834
H 0 4.067472 -1.808123 2.212869
H 0 2.464887 -2.478707 1.842495
H 0 3.819982 -3.565714 2.171212
H 0 3.690470 0.145887 1.005692
H 0 5.195664 -0.235493 0.171039
H 0 2.851148 1.309436 -1.076479
H 0 4.374640 0.947614 -1.879366
H 0 4.075505 2.655007 0.654655
H 0 5.603983 2.283070 -0.131155
H 0 3.287716 3.815404 -1.435162
H 0 4.820658 3.450534 -2.212869
H 0 4.491475 5.199069 0.304656
H 0 4.820595 5.794959 -1.330492
H 0 6.036511 4.830698 -0.476847
Free Energy (PCM/B3LYP/6-31G*) = -1757.652641
Number of imaginary frequencies = 0

1-3*R*-c41
B3LYP/6-31G* geometry
O 0 1.706462 -1.047605 -0.862040
C 0 1.386307 -3.401211 -0.610291
C 0 3.210034 -2.353064 0.666030
C 0 2.703610 -3.680540 0.042094
C 0 3.099773 -1.245481 -0.423852
C 0 1.003781 -2.169562 -0.998244
C 0 -0.393590 -2.165405 -1.573876
N 0 -0.815943 -3.542068 -1.245029
C 0 0.299519 -4.329725 -0.876951
O 0 -1.678631 -4.122260 -2.197284
C 0 -2.818136 -4.703568 -1.544466
C 0 -1.278751 -1.071759 -0.970484
S 0 -2.911430 -1.008878 -1.820286
C 0 -3.593839 0.562191 -1.168572
C 0 -4.242728 0.454919 0.214533
C 0 -4.989244 1.765003 0.536700
O 0 -3.248895 0.220488 1.219024
O 0 -5.884038 2.201965 -0.155414
O 0 -4.559251 2.388946 1.639509
O 0 0.291324 -5.553418 -0.763780
O 0 2.611053 -4.682620 1.051209
O 0 4.614198 -2.460000 0.919827
C 0 2.460411 -2.000992 1.955898
C 0 3.623894 0.123953 -0.013163
C 0 3.733085 1.113538 -1.182018
C 0 4.254020 2.488475 -0.745516
C 0 4.376646 3.488710 -1.902179
C 0 4.889593 4.862889 -1.459371
H 0 3.450764 -3.986132 -0.707285
H 0 3.643735 -1.600824 -1.308164
H 0 -0.349938 -2.056295 -2.666263
H 0 -3.372311 -3.947215 -0.980310
H 0 -2.509535 -5.522482 -0.888670
H 0 -3.436886 -5.091075 -2.357563
H 0 -0.782820 -0.106320 -1.110100
H 0 -1.417376 -1.244152 0.098055
H 0 -4.356663 0.875704 -1.885788
H 0 -2.806638 1.323447 -1.158493
H 0 -4.985621 -0.348845 0.204989
H 0 -3.537942 -0.513703 1.783684
H 0 -3.830509 1.829625 1.996997
H 0 2.103227 -5.417747 0.661214
H 0 4.733016 -3.242177 1.485039
H 0 2.891879 -1.103400 2.406023
H 0 1.395126 -1.824826 1.777912
H 0 2.548854 -2.825786 2.667673
H 0 2.971168 0.538753 0.764562
H 0 4.608940 -0.027687 0.441668
H 0 2.750640 1.229570 -1.657626
H 0 4.402018 0.696036 -1.948496
H 0 3.584431 2.903632 0.022313
H 0 5.235293 2.369449 -0.262539
H 0 3.396390 3.602661 -2.386632
H 0 5.049005 3.076002 -2.667673
H 0 4.218926 5.315633 -0.718495
H 0 4.964628 5.553418 -2.307326
H 0 5.884038 4.786084 -1.002451
Free Energy (PCM/B3LYP/6-31G*) = -1757.65257
Number of imaginary frequencies = 0

1-3*R*-c42
B3LYP/6-31G* geometry
O 0 1.242223 -0.565173 -0.210935
C 0 0.158805 -2.659073 -0.591704
C 0 2.555096 -2.695623 -0.031320
C 0 1.408756 -3.469981 -0.733848
C 0 2.517224 -1.225704 -0.545610
C 0 0.164995 -1.332482 -0.354545
C 0 -1.229286 -0.776128 -0.183906
N 0 -2.010743 -2.031756 -0.159905
C 0 -1.218562 -3.117593 -0.617243
O 0 -3.283447 -1.942920 -0.774106
C 0 -4.291200 -2.529269 0.071577
C 0 -1.367188 0.075499 1.084792
S 0 -3.032730 0.761295 1.437454
C 0 -3.221116 2.087284 0.168753
C 0 -4.121297 1.710676 -1.022181
C 0 -5.554592 1.460663 -0.536345
O 0 -3.604836 0.688676 -1.840354
O 0 -6.136817 2.195712 0.235264
O 0 -6.103829 0.351650 -1.062895
O 0 -1.656656 -4.225343 -0.914504
O 0 1.287126 -4.768569 -0.159971
O 0 3.811766 -3.200654 -0.492341
C 0 2.467373 -2.795959 1.495935
C 0 3.625019 -0.323543 -0.018903
C 0 3.693537 1.037538 -0.726181
C 0 4.819882 1.927701 -0.186413
C 0 4.903512 3.292323 -0.882265
C 0 6.029338 4.177967 -0.338434
H 0 1.690648 -3.564670 -1.794243
H 0 2.556971 -1.260124 -1.641677
H 0 -1.512860 -0.186699 -1.064251
H 0 -4.104785 -3.599442 0.185004
H 0 -5.230094 -2.360414 -0.459395
H 0 -4.313194 -2.032069 1.044692
H 0 -0.647025 0.897979 1.043156
H 0 -1.130426 -0.534746 1.961523
H 0 -2.230892 2.368203 -0.198947
H 0 -3.653641 2.949248 0.681671
H 0 -4.182118 2.603495 -1.659615
H 0 -3.677684 -0.172968 -1.379473
H 0 -7.007415 0.275897 -0.697877
H 0 0.455842 -5.144196 -0.502784
H 0 3.803238 -4.157633 -0.320287
H 0 1.560052 -2.326110 1.887718
H 0 2.457403 -3.847124 1.795371
H 0 3.334798 -2.311684 1.951369
H 0 3.485728 -0.174838 1.058774
H 0 4.570940 -0.860635 -0.149328
H 0 2.732720 1.556628 -0.615795
H 0 3.838755 0.878294 -1.804546
H 0 4.676945 2.080586 0.893642
H 0 5.781575 1.404819 -0.296019
H 0 3.941819 3.813327 -0.772140
H 0 5.046187 3.139931 -1.961523
H 0 5.894810 4.376392 0.732198
H 0 6.062709 5.144196 -0.854948
H 0 7.007415 3.697511 -0.465621
Free Energy (PCM/B3LYP/6-31G*) = -1757.652562
Number of imaginary frequencies = 0

1-3*S*-c1
B3LYP/6-31G* geometry
O 0 -1.049943 -0.626611 -0.619236
C 0 -1.436958 1.540175 0.331852
C 0 -0.709530 -0.287949 1.823904
C 0 -0.881986 1.252515 1.688314
C 0 -0.173175 -0.941140 0.525824
C 0 -1.484827 0.631062 -0.661738
C 0 -2.138693 1.180615 -1.904497
N 0 -2.624757 2.476224 -1.397685

C 0 -2.007654 2.790934 -0.164126
O 0 -2.625014 3.499842 -2.362633
C 0 -3.923785 4.104195 -2.442560
C 0 -3.236001 0.311599 -2.526045
S 0 -4.656800 0.023547 -1.383092
C 0 -4.850966 -1.798048 -1.503354
C 0 -3.815947 -2.593946 -0.697776
C 0 -3.974606 -4.099391 -0.981372
O 0 -3.969945 -2.389850 0.700834
O 0 -4.005262 -4.570894 -2.099458
O 0 -4.065992 -4.850673 0.121936
O 0 -1.994252 3.902891 0.349553
O 0 0.373461 1.899062 1.920684
O 0 -2.029952 -0.855637 1.969895
C 0 0.138982 -0.665671 3.038140
C 0 1.271449 -0.606253 0.148268
C 0 1.756320 -1.338982 -1.110871
C 0 3.217946 -1.019982 -1.449324
C 0 3.720647 -1.736483 -2.709231
C 0 5.178713 -1.409215 -3.047537
H 0 -1.599685 1.569703 2.461037
H 0 -0.292919 -2.022372 0.632181
H 0 -1.373696 1.364883 -2.672770
H 0 -4.688579 3.365461 -2.705396
H 0 -3.838014 4.850673 -3.235901
H 0 -4.183975 4.592399 -1.498618
H 0 -2.808497 -0.652385 -2.815816
H 0 -3.601469 0.798882 -3.433438
H 0 -4.832969 -2.107944 -2.550814
H 0 -5.849843 -2.003221 -1.105834
H 0 -2.804982 -2.320179 -1.021341
H 0 -3.226511 -1.847821 1.044474
H 0 -4.023991 -4.207305 0.871569
H 0 0.235696 2.843056 1.733209
H 0 -2.406708 -0.558708 2.814715
H 0 -0.356771 -0.315401 3.951110
H 0 1.126710 -0.204030 3.002052
H 0 0.247327 -1.752970 3.101776
H 0 1.376608 0.477324 0.027635
H 0 1.910368 -0.886130 0.993850
H 0 1.642078 -2.423509 -0.968152
H 0 1.118230 -1.070161 -1.961871
H 0 3.330847 0.066349 -1.581094
H 0 3.858760 -1.292210 -0.597554
H 0 3.077986 -1.466350 -3.559166
H 0 3.610465 -2.822117 -2.576311
H 0 5.313234 -0.334135 -3.220078
H 0 5.506910 -1.935782 -3.951110
H 0 5.849843 -1.699640 -2.229665
Free Energy (PCM/B3LYP/6-31G*) = -1757.657679
Number of imaginary frequencies = 0

1-3*S*-c2
B3LYP/6-31G* geometry
O 0 1.178640 -0.378593 -0.899897
C 0 2.067533 -2.394292 0.065506
C 0 1.090149 -0.722298 1.558077
C 0 1.458421 -2.225759 1.425137
C 0 0.341790 -0.193885 0.304420
C 0 1.905830 -1.489775 -0.926780
C 0 2.797126 -1.773573 -2.108133
N 0 3.589145 -2.896883 -1.581245
C 0 3.019391 -3.410324 -0.394032
O 0 3.968561 -3.841967 -2.549121
C 0 5.391544 -4.030812 -2.528342
C 0 3.645164 -0.569198 -2.536053
S 0 4.719308 0.051690 -1.162794
C 0 4.513905 1.870170 -1.346997
C 0 3.260814 2.439719 -0.667662
C 0 3.051589 3.905605 -1.092156
O 0 3.364302 2.407961 0.750590
O 0 3.058184 4.273184 -2.248579
O 0 2.844293 4.743128 -0.070171
O 0 3.319383 -4.478733 0.119930
O 0 0.292485 -3.019436 1.646630
O 0 2.338725 0.007447 1.662684
C 0 0.319012 -0.423071 2.838922
C 0 -1.052875 -0.755226 0.021215
C 0 -1.744805 -0.079958 -1.171693
C 0 -3.159933 -0.620740 -1.413443
C 0 -3.866928 0.036976 -2.605446
C 0 -5.279577 -0.507090 -2.842254
H 0 2.209469 -2.448082 2.196937
H 0 0.289078 0.894615 0.396012
H 0 2.212163 -2.116161 -2.972503
H 0 5.712939 -4.450202 -1.570436
H 0 5.918313 -3.090425 -2.722136
H 0 5.591353 -4.743128 -3.332335
H 0 2.979563 0.236484 -2.856886
H 0 4.273667 -0.850696 -3.384217
H 0 4.521431 2.129897 -2.407817
H 0 5.401083 2.310769 -0.882484
H 0 2.374411 1.890366 -1.007802
H 0 2.827740 1.666676 1.110877
H 0 2.906082 4.183480 0.741439
H 0 0.545413 -3.944479 1.491773
H 0 3.000223 -0.401776 1.070656
H 0 0.945148 -0.678816 3.698859
H 0 -0.600540 -1.007084 2.893526
H 0 0.071474 0.641996 2.897243
H 0 -0.987181 -1.837047 -0.131621
H 0 -1.661664 -0.607240 0.921030
H 0 -1.793293 1.005072 -0.998379
H 0 -1.140871 -0.222546 -2.076610
H 0 -3.110868 -1.707593 -1.576440
H 0 -3.765836 -0.475193 -0.506864
H 0 -3.261023 -0.109843 -3.510803
H 0 -3.915096 1.123115 -2.443154
H 0 -5.260040 -1.585801 -3.041528
H 0 -5.758037 -0.018411 -3.698859
H 0 -5.918313 -0.344111 -1.965278
Free Energy (PCM/B3LYP/6-31G*) = -1757.656761
Number of imaginary frequencies = 0

1-3*S*-c3
B3LYP/6-31G* geometry
O 0 -0.494947 -0.690280 -0.171493
C 0 -0.778462 1.515191 0.726961
C 0 -0.112200 -0.301218 2.258190
C 0 -0.210542 1.241057 2.080928
C 0 0.378505 -1.014609 0.973657
C 0 -0.880466 0.582165 -0.240308
C 0 -1.540534 1.122584 -1.484060
N 0 -1.972798 2.445777 -1.000733
C 0 -1.318867 2.771203 0.210845
O 0 -1.961364 3.444137 -1.991761
C 0 -3.242334 4.086611 -2.061069
C 0 -2.678773 0.276754 -2.062290
S 0 -4.079949 0.051136 -0.881363
C 0 -4.329635 -1.765832 -0.964107
C 0 -3.308569 -2.576310 -0.155494
C 0 -3.513967 -4.081510 -0.407296
O 0 -3.438799 -2.341623 1.240651
O 0 -3.574983 -4.573169 -1.515391
O 0 -3.609153 -4.809214 0.711487
O 0 -1.260002 3.894601 0.695492
O 0 1.079909 1.829484 2.271676
O 0 -1.458128 -0.797193 2.430817
C 0 0.728189 -0.687456 3.475254
C 0 1.832708 -0.760337 0.571150
C 0 2.268935 -1.565477 -0.661400
C 0 3.738680 -1.315797 -1.027590
C 0 4.244928 -2.138810 -2.223948
C 0 3.581594 -1.789325 -3.561868
H 0 -0.896171 1.615514 2.856972
H 0 0.208968 -2.085613 1.111694
H 0 -0.786010 1.262049 -2.271950
H 0 -4.034866 3.365513 -2.288296
H 0 -3.150719 4.809214 -2.875583
H 0 -3.467072 4.607037 -1.125273
H 0 -2.287758 -0.704584 -2.345181
H 0 -3.051519 0.759519 -2.969195
H 0 -4.333223 -2.094891 -2.005831
H 0 -5.329084 -1.934381 -0.551196

H 0 -2.294358 -2.338230 -0.496461
H 0 -2.682731 -1.803307 1.562062
H 0 -3.537850 -4.153738 1.448165
H 0 0.985987 2.773999 2.061690
H 0 -1.813709 -0.454967 3.267736
H 0 0.257173 -0.290027 4.381936
H 0 1.736391 -0.274536 3.420191
H 0 0.785412 -1.776625 3.567232
H 0 1.983696 0.311661 0.404754
H 0 2.468177 -1.033024 1.421621
H 0 2.119519 -2.637862 -0.466570
H 0 1.621676 -1.311032 -1.508046
H 0 3.882408 -0.245703 -1.239257
H 0 4.365413 -1.540917 -0.152961
H 0 4.102061 -3.208236 -2.012483
H 0 5.329084 -1.989088 -2.315276
H 0 2.506585 -2.001673 -3.554050
H 0 4.024172 -2.366516 -4.381936
H 0 3.708198 -0.724897 -3.797178
Free Energy (PCM/B3LYP/6-31G*) = -1757.656596
Number of imaginary frequencies = 0

1-3*S*-c4
B3LYP/6-31G* geometry
O 0 0.503900 -0.644862 -0.150492
C 0 -0.424008 -2.831488 -0.380129
C 0 1.812691 -2.631281 0.633373
C 0 0.837714 -3.588558 -0.107722
C 0 1.834464 -1.280993 -0.144359
C 0 -0.505802 -1.486868 -0.345172
C 0 -1.915132 -0.994021 -0.582406
N 0 -2.584421 -2.269207 -0.928108
C 0 -1.758913 -3.365768 -0.575536
O 0 -3.919448 -2.381550 -0.467338
C 0 -4.776554 -2.837328 -1.532313
C 0 -1.983953 0.079212 -1.676433
S 0 -3.651311 0.707094 -2.116636
C 0 -4.139613 1.712086 -0.653373
C 0 -5.105948 0.995184 0.298943
C 0 -5.666679 1.986409 1.333803
O 0 -4.501465 -0.067105 1.025941
O 0 -6.097381 3.080970 1.038658
O 0 -5.663968 1.534408 2.593953
O 0 -2.140560 -4.529787 -0.473536
O 0 0.606962 -4.757009 0.675464
O 0 3.147259 -3.132140 0.509248
C 0 1.441597 -2.461221 2.110684
C 0 2.810354 -0.239559 0.386081
C 0 2.968885 0.978505 -0.535235
C 0 3.948854 2.017450 0.023809
C 0 4.125749 3.239797 -0.886166
C 0 5.099568 4.277679 -0.318837
H 0 1.329598 -3.874077 -1.051176
H 0 2.062936 -1.506413 -1.193958
H 0 -2.338081 -0.605088 0.350943
H 0 -5.774802 -2.873945 -1.090460
H 0 -4.471142 -3.835779 -1.852604
H 0 -4.754476 -2.133303 -2.367691
H 0 -1.591341 -0.326938 -2.613563
H 0 -1.351177 0.922764 -1.384926
H 0 -4.633072 2.605922 -1.041821
H 0 -3.238964 2.028238 -0.117986
H 0 -5.968527 0.629993 -0.274652
H 0 -4.501881 -0.891186 0.493512
H 0 -5.256891 0.638360 2.564741
H 0 -0.236500 -5.137139 0.363537
H 0 3.145182 -4.025586 0.892372
H 0 1.418590 -3.439493 2.597512
H 0 0.457039 -2.000973 2.237736
H 0 2.185162 -1.837812 2.613563
H 0 2.480204 0.087648 1.379525
H 0 3.777466 -0.736901 0.517440
H 0 3.316138 0.643167 -1.523281
H 0 1.989768 1.448870 -0.693599
H 0 3.600304 2.349877 1.012913
H 0 4.927823 1.543362 0.188861
H 0 3.146294 3.709872 -1.054008
H 0 4.478062 2.908029 -1.873199
H 0 5.203850 5.137139 -0.991107
H 0 6.097381 3.845818 -0.172029
H 0 4.755395 4.653623 0.652699
Free Energy (PCM/B3LYP/6-31G*) = -1757.655574
Number of imaginary frequencies = 0

1-3*S*-c5
B3LYP/6-31G* geometry
O 0 1.255879 -0.794084 -0.023321
C 0 -0.144004 -2.543717 -0.858192
C 0 2.282068 -2.738990 -1.227890
C 0 0.884112 -3.177074 -1.742054
C 0 2.252919 -1.193564 -1.030874
C 0 0.104807 -1.461252 -0.098118
C 0 -1.090077 -1.065826 0.735775
N 0 -1.946686 -2.241329 0.495001
C 0 -1.497971 -3.018498 -0.580009
O 0 -3.317606 -2.136046 0.711396
C 0 -3.706609 -2.880639 1.885458
C 0 -1.704785 0.290912 0.346925
S 0 -2.104231 0.414451 -1.452185
C 0 -3.638823 1.424034 -1.426167
C 0 -4.906063 0.608226 -1.153756
C 0 -6.160501 1.475924 -1.347147
O 0 -4.956683 0.089437 0.170071
O 0 -6.321751 2.208209 -2.300285
O 0 -7.081367 1.339826 -0.384628
O 0 -2.106942 -3.950024 -1.100543
O 0 0.795474 -4.599415 -1.748609
O 0 3.248103 -2.946664 -2.262697
C 0 2.689728 -3.494401 0.042497
C 0 3.574630 -0.568092 -0.605285
C 0 3.570574 0.966131 -0.663156
C 0 4.905641 1.579383 -0.222687
C 0 4.921101 3.112057 -0.285076
C 0 6.253502 3.720441 0.164611
H 0 0.792088 -2.801451 -2.773364
H 0 1.920028 -0.748129 -1.977223
H 0 -0.819855 -1.008931 1.797794
H 0 -3.482069 -3.940610 1.745052
H 0 -4.784342 -2.729660 1.971724
H 0 -3.198143 -2.494487 2.773364
H 0 -0.985354 1.069439 0.614742
H 0 -2.618602 0.453018 0.919174
H 0 -3.710795 1.875650 -2.418471
H 0 -3.535058 2.230147 -0.693927
H 0 -4.974229 -0.204382 -1.890176
H 0 -4.449234 -0.747125 0.216115
H 0 -6.703554 0.709366 0.270759
H 0 -0.150148 -4.814813 -1.851488
H 0 3.193372 -3.886247 -2.506605
H 0 2.661010 -4.570788 -0.145224
H 0 2.018570 -3.282297 0.880509
H 0 3.706621 -3.217706 0.332058
H 0 3.817140 -0.900670 0.411437
H 0 4.351623 -0.965445 -1.267423
H 0 2.763740 1.354884 -0.028540
H 0 3.341841 1.288777 -1.689264
H 0 5.132477 1.256561 0.804330
H 0 5.714893 1.182064 -0.853253
H 0 4.108237 3.507432 0.340531
H 0 4.699919 3.434553 -1.312428
H 0 6.484127 3.446087 1.201594
H 0 6.233357 4.814813 0.105870
H 0 7.081367 3.367612 -0.462964
Free Energy (PCM/B3LYP/6-31G*) = -1757.655562
Number of imaginary frequencies = 0

1-3*S*-c6
B3LYP/6-31G* geometry
O 0 -1.926596 -0.204306 -0.495809
C 0 -3.116607 -2.097737 0.338079
C 0 -4.240530 0.092708 0.410395
C 0 -4.206796 -1.335526 1.022065
C 0 -2.776350 0.624712 0.376711
C 0 -2.126082 -1.512551 -0.362810

C 0 -1.221370 -2.529975 -1.016317
N 0 -1.726705 -3.770849 -0.391158
C 0 -2.986229 -3.540122 0.204094
O 0 -1.644898 -4.900705 -1.224978
C 0 -0.925621 -5.952069 -0.563975
C 0 0.281224 -2.304984 -0.846053
S 0 0.773444 -2.315465 0.930429
C 0 2.318785 -1.331877 0.879833
C 0 3.549681 -2.120017 0.386766
C 0 4.785180 -1.224244 0.453014
O 0 3.398945 -2.595756 -0.933440
O 0 5.344605 -0.805231 -0.542061
O 0 5.151989 -0.932219 1.706875
O 0 -3.785022 -4.413564 0.538135
O 0 -5.478216 -1.964686 0.875314
O 0 -4.919187 0.974309 1.310599
C 0 -4.913620 0.113723 -0.966467
C 0 -2.617136 2.063187 -0.095153
C 0 -1.199941 2.621189 0.100623
C 0 -1.069586 4.074714 -0.370713
C 0 0.338193 4.654203 -0.180220
C 0 0.460788 6.105919 -0.654340
H 0 -3.983156 -1.216439 2.094278
H 0 -2.362359 0.517383 1.387654
H 0 -1.433501 -2.574338 -2.094278
H 0 0.084358 -5.628790 -0.291259
H 0 -0.874782 -6.760782 -1.296911
H 0 -1.463720 -6.290646 0.326682
H 0 0.538926 -1.331191 -1.272576
H 0 0.835258 -3.070453 -1.390829
H 0 2.485377 -0.999061 1.908522
H 0 2.162572 -0.444978 0.256142
H 0 3.706005 -2.978497 1.049908
H 0 3.887035 -1.971229 -1.503059
H 0 5.922132 -0.331318 1.666817
H 0 -5.319395 -2.924976 0.952761
H 0 -5.805188 0.599755 1.451454
H 0 -5.922132 -0.299920 -0.887236
H 0 -4.364507 -0.482677 -1.701373
H 0 -4.984214 1.140826 -1.333415
H 0 -2.897282 2.128043 -1.153585
H 0 -3.337137 2.669843 0.464663
H 0 -0.928206 2.556404 1.164270
H 0 -0.480316 1.994829 -0.442304
H 0 -1.346437 4.138174 -1.433600
H 0 -1.795320 4.699170 0.171235
H 0 1.062593 4.029342 -0.721743
H 0 0.614644 4.591286 0.881840
H 0 0.221640 6.195195 -1.721419
H 0 1.476578 6.490419 -0.506741
H 0 -0.227203 6.760782 -0.105276
Free Energy (PCM/B3LYP/6-31G*) = -1757.655438
Number of imaginary frequencies = 0

2-3*R*-c1
B3LYP/6-31G* geometry
O 0 1.861966 -1.193168 0.032163
C 0 1.660852 -3.550197 -0.331360
C 0 3.916309 -2.639192 0.135670
C 0 3.120263 -3.822846 -0.482723
C 0 3.280591 -1.300817 -0.353751
C 0 1.161774 -2.314563 -0.101867
C 0 -0.339832 -2.326902 0.059632
N 0 -0.579747 -3.783014 0.095561
C 0 0.547199 -4.496195 -0.372793
O 0 -1.820343 -4.169560 -0.446077
C 0 -2.520699 -5.013289 0.477913
C 0 -0.884561 -1.644283 1.323652
S 0 -0.843282 0.197832 1.296393
C 0 -2.584581 0.646333 0.934026
C 0 -2.954935 0.632232 -0.562922
C 0 -4.396411 1.109107 -0.730198
O 0 -2.827465 -0.646321 -1.147089
O 0 -5.302366 0.360747 -1.042438
O 0 -4.548150 2.411914 -0.462228
O 0 0.538080 -5.677219 -0.698006
O 0 3.527937 -3.898858 -1.864721
O 0 5.254629 -2.686658 -0.348790
C 0 3.971154 -2.749196 1.659095
C 0 3.962864 -0.034736 0.144655
C 0 3.475921 1.242208 -0.554763
C 0 4.181442 2.504847 -0.044217
C 0 3.707619 3.789313 -0.736277
C 0 4.411417 5.048147 -0.218880
H 0 3.402482 -4.752257 0.026957
H 0 3.287573 -1.325478 -1.451124
H 0 -0.834632 -1.897871 -0.819192
H 0 -2.684955 -4.504088 1.433807
H 0 -1.974765 -5.947241 0.641251
H 0 -3.479730 -5.222690 -0.002449
H 0 -0.315429 -1.967308 2.199699
H 0 -1.923312 -1.951026 1.464142
H 0 -2.706114 1.659437 1.328761
H 0 -3.250857 -0.016743 1.495752
H 0 -2.292589 1.321869 -1.097509
H 0 -3.720583 -1.039737 -1.146205
H 0 -5.495912 2.633727 -0.552940
H 0 3.294710 -4.779172 -2.199699
H 0 5.185998 -3.076044 -1.242678
H 0 4.370553 -3.726968 1.945430
H 0 2.982966 -2.628905 2.112427
H 0 4.632630 -1.980678 2.068653
H 0 3.809695 0.054942 1.227442
H 0 5.038803 -0.163322 -0.018886
H 0 3.638986 1.145752 -1.638154
H 0 2.392458 1.346693 -0.412231
H 0 4.020435 2.597792 1.040151
H 0 5.267563 2.397200 -0.183177
H 0 2.621998 3.894129 -0.599009
H 0 3.870679 3.697910 -1.819581
H 0 4.237664 5.185435 0.855687
H 0 4.051433 5.947241 -0.732317
H 0 5.495912 4.987302 -0.373528
Free Energy (PCM/B3LYP/6-31G*) =-1757.658874
Number of imaginary frequencies = 0

2-3*R*-c2
B3LYP/6-31G* geometry
O 0 1.297287 -0.924560 -0.057845
C 0 0.255260 -3.064626 -0.335352
C 0 2.677351 -3.016433 0.172773
C 0 1.517712 -3.854893 -0.435981
C 0 2.589468 -1.560503 -0.383873
C 0 0.243097 -1.723706 -0.169694
C 0 -1.157697 -1.170982 -0.053072
N 0 -1.930692 -2.426649 0.011334
C 0 -1.127821 -3.527909 -0.393702
O 0 -3.193167 -2.365995 -0.625360
C 0 -4.210494 -2.921990 0.226373
C 0 -1.325571 -0.255721 1.167064
S 0 -2.994257 0.461456 1.439070
C 0 -3.138362 1.718335 0.098489
C 0 -3.974453 1.281994 -1.124543
C 0 -5.415532 0.955403 -0.730476
O 0 -3.408777 0.222310 -1.854124
O 0 -5.929576 -0.136340 -0.852983
O 0 -6.047610 2.027162 -0.217606
O 0 -1.570823 -4.629238 -0.689828
O 0 1.894013 -4.121500 -1.802487
O 0 3.915138 -3.569989 -0.261366
C 0 2.655469 -3.071624 1.700079
C 0 3.674313 -0.610687 0.102511
C 0 3.708236 0.724737 -0.654269
C 0 4.810895 1.663760 -0.149606
C 0 4.859685 3.003478 -0.895436
C 0 5.960565 3.938265 -0.383806
H 0 1.434597 -4.803755 0.107317
H 0 2.613759 -1.633303 -1.478550
H 0 -1.422170 -0.624442 -0.966492
H 0 -4.235575 -2.399450 1.186253
H 0 -4.032879 -3.990295 0.370873
H 0 -5.144771 -2.757166 -0.312971
H 0 -0.598182 0.559065 1.104199
H 0 -1.119610 -0.821504 2.080707

H 0 -2.133141 1.986834 -0.237554
H 0 -3.590698 2.605611 0.549357
H 0 -4.011534 2.146719 -1.799384
H 0 -3.595913 -0.619444 -1.387530
H 0 -6.951332 1.749928 0.030107
H 0 1.382931 -4.885281 -2.113707
H 0 3.729426 -3.938998 -1.147310
H 0 2.655397 -4.113872 2.033567
H 0 1.774675 -2.572187 2.113707
H 0 3.547324 -2.586564 2.106308
H 0 3.536720 -0.425174 1.175161
H 0 4.632042 -1.130118 -0.014053
H 0 3.856762 0.529451 -1.726287
H 0 2.734163 1.222608 -0.562101
H 0 4.663735 1.852733 0.924200
H 0 5.786040 1.162498 -0.240211
H 0 3.884301 3.502473 -0.805550
H 0 5.008412 2.815336 -1.968242
H 0 5.818665 4.172463 0.678607
H 0 5.969443 4.885281 -0.935732
H 0 6.951332 3.479614 -0.492079
Free Energy (PCM/B3LYP/6-31G*) = -1757.658849
Number of imaginary frequencies = 0

2-3*R*-c3
B3LYP/6-31G* geometry
O 0 -0.912080 -1.416251 -0.721948
C 0 -3.271978 -1.687308 -0.415627
C 0 -1.762500 -3.265993 0.752229
C 0 -3.204483 -3.037886 0.216720
C 0 -0.747216 -2.835520 -0.352169
C 0 -2.173685 -1.012186 -0.820239
C 0 -2.515439 0.345150 -1.388462
N 0 -3.949345 0.410535 -1.054649
C 0 -4.448123 -0.867029 -0.704870
O 0 -4.713525 1.138711 -1.985050
C 0 -5.494759 2.135876 -1.311537
C 0 -1.756564 1.543963 -0.801851
S 0 0.000431 1.583615 -1.365403
C 0 0.324754 3.389063 -1.437331
C 0 0.556959 4.066354 -0.078578
C 0 1.680416 3.405053 0.713865
O 0 -0.619073 4.069516 0.701720
O 0 1.521298 2.981966 1.842987
O 0 2.838489 3.362704 0.046178
O 0 -5.635788 -1.155978 -0.641056
O 0 -3.450734 -4.101056 -0.726966
O 0 -1.580828 -4.658050 0.989349
C 0 -1.541415 -2.537631 2.077492
C 0 0.720697 -3.025274 0.001228
C 0 1.668362 -2.850566 -1.193801
C 0 3.141365 -3.056017 -0.818639
C 0 4.100074 -2.890742 -2.004719
C 0 5.570612 -3.091786 -1.624289
H 0 -3.915757 -3.121247 1.047313
H 0 -0.994971 -3.406228 -1.256395
H 0 -2.398516 0.332570 -2.480713
H 0 -6.007082 2.678456 -2.109822
H 0 -4.856839 2.820944 -0.743027
H 0 -6.229045 1.670098 -0.647522
H 0 -1.811810 1.533729 0.288451
H 0 -2.237181 2.457396 -1.159636
H 0 -0.506950 3.887174 -1.944131
H 0 1.216286 3.501791 -2.060486
H 0 0.872954 5.102594 -0.282095
H 0 -0.377078 3.657227 1.553372
H 0 3.503933 2.931160 0.617272
H 0 -4.410373 -4.189803 -0.839331
H 0 -2.142125 -5.102594 0.323925
H 0 -2.316224 -2.826740 2.794165
H 0 -1.566095 -1.451109 1.954640
H 0 -0.570848 -2.813199 2.499330
H 0 0.993715 -2.321299 0.797198
H 0 0.823367 -4.034838 0.414666
H 0 1.389943 -3.564010 -1.983141
H 0 1.537629 -1.848154 -1.621909
H 0 3.417870 -2.343132 -0.027608
H 0 3.270312 -4.058622 -0.384296
H 0 3.967758 -1.889695 -2.439305
H 0 3.824987 -3.604564 -2.794165
H 0 5.883564 -2.370009 -0.859504
H 0 6.229045 -2.965230 -2.491478
H 0 5.741137 -4.097252 -1.219763
Free Energy (PCM/B3LYP/6-31G*) = -1757.658434
Number of imaginary frequencies = 0

2-3*R*-c4
B3LYP/6-31G* geometry
O 0 0.722647 -0.511680 -0.824456
C 0 -0.266992 -2.669196 -0.487484
C 0 1.668056 -2.050088 0.926775
C 0 0.832676 -3.229667 0.351759
C 0 1.963860 -1.038343 -0.224887
C 0 -0.237211 -1.413881 -0.985709
C 0 -1.480222 -1.085716 -1.775544
N 0 -2.311725 -2.257274 -1.452682
C 0 -1.522029 -3.297353 -0.902608
O 0 -3.190554 -2.633834 -2.483929
C 0 -4.538809 -2.673140 -1.994838
C 0 -2.141040 0.260371 -1.473690
S 0 -2.721663 0.354046 0.273749
C 0 -2.876619 2.169256 0.503353
C 0 -4.135461 2.798729 -0.111572
C 0 -5.416493 2.136296 0.386550
O 0 -4.104088 2.739846 -1.521848
O 0 -6.246346 1.668836 -0.370023
O 0 -5.528749 2.147303 1.719043
O 0 -1.886373 -4.460392 -0.792961
O 0 1.754361 -4.035462 -0.411969
O 0 2.916685 -2.558187 1.385946
C 0 0.962320 -1.404422 2.118593
C 0 2.803007 0.168862 0.168800
C 0 3.274390 1.006493 -1.028050
C 0 4.134978 2.206010 -0.611082
C 0 4.606810 3.057713 -1.796281
C 0 5.467375 4.253271 -1.374336
H 0 0.433474 -3.826808 1.180384
H 0 2.462871 -1.599517 -1.025362
H 0 -1.240994 -1.103624 -2.849095
H 0 -5.146562 -2.930707 -2.865745
H 0 -4.844710 -1.698911 -1.599066
H 0 -4.649638 -3.441639 -1.223703
H 0 -2.980413 0.417353 -2.151964
H 0 -1.412470 1.058234 -1.644238
H 0 -1.993722 2.665259 0.089745
H 0 -2.878776 2.331044 1.584698
H 0 -4.167342 3.850033 0.218962
H 0 -4.945441 2.323359 -1.791399
H 0 -6.371106 1.712722 1.957399
H 0 1.368458 -4.919608 -0.515872
H 0 3.113850 -3.312598 0.796566
H 0 0.724562 -2.168011 2.865745
H 0 0.036110 -0.903795 1.821713
H 0 1.618323 -0.665706 2.587691
H 0 2.227793 0.799437 0.858180
H 0 3.668287 -0.209444 0.724656
H 0 3.848176 0.367098 -1.714646
H 0 2.402050 1.361676 -1.591928
H 0 3.564339 2.838716 0.084924
H 0 5.010929 1.848237 -0.049723
H 0 3.730406 3.415990 -2.354916
H 0 5.174812 2.425028 -2.493120
H 0 4.914988 4.919608 -0.700166
H 0 5.784407 4.844061 -2.241519
H 0 6.371106 3.923796 -0.846725
Free Energy (PCM/B3LYP/6-31G*) =-1757.658353
Number of imaginary frequencies = 0

2-3*R*-c5
B3LYP/6-31G* geometry
O 0 0.548057 -0.745127 -0.319995
C 0 -1.278266 0.772861 0.012483
C 0 0.612030 1.516450 -1.417132
C 0 -0.615748 1.975735 -0.570494

C 0 1.394271 0.425631 -0.619331
C 0 -0.680540 -0.437325 0.077785
C 0 -1.599517 -1.489995 0.644835
N 0 -2.660728 -0.614808 1.167225
C 0 -2.639941 0.629557 0.536322
O 0 -3.888201 -1.207069 1.447896
C 0 -4.156177 -1.157720 2.861932
C 0 -2.112054 -2.523980 -0.389664
S 0 -2.694374 -1.831806 -1.996622
C 0 -4.515004 -2.066850 -2.025499
C 0 -5.351211 -1.008189 -1.285708
C 0 -6.844376 -1.284624 -1.523862
O 0 -5.063869 0.316984 -1.722499
O 0 -7.362639 -2.368095 -1.344829
O 0 -7.535354 -0.216770 -1.941805
O 0 -3.597067 1.406645 0.467958
O 0 -0.105049 2.854984 0.456030
O 0 1.481059 2.629596 -1.587843
C 0 0.178900 1.045949 -2.805012
C 0 2.647950 -0.106897 -1.297980
C 0 3.511785 -0.986655 -0.383295
C 0 4.771593 -1.515384 -1.080344
C 0 5.639747 -2.400202 -0.176639
C 0 6.895591 -2.930074 -0.876429
H 0 -1.305904 2.533795 -1.212825
H 0 1.649877 0.861943 0.355503
H 0 -1.117245 -2.041803 1.460150
H 0 -5.113546 -1.668509 2.982186
H 0 -4.236170 -0.121056 3.201005
H 0 -3.376354 -1.681472 3.422974
H 0 -2.913038 -3.111935 0.063600
H 0 -1.285001 -3.200405 -0.620038
H 0 -4.774062 -3.062240 -1.656955
H 0 -4.750579 -2.037389 -3.094582
H 0 -5.179533 -1.099657 -0.209894
H 0 -4.628256 0.796361 -0.984294
H 0 -6.867203 0.507999 -2.011777
H 0 -0.754854 3.558757 0.606979
H 0 1.356606 3.163505 -0.777239
H 0 -0.397962 1.835024 -3.297374
H 0 -0.436811 0.143514 -2.756898
H 0 1.056034 0.834225 -3.422974
H 0 2.360851 -0.672883 -2.192959
H 0 3.223690 0.761531 -1.637147
H 0 3.801884 -0.408275 0.505976
H 0 2.913461 -1.832614 -0.020674
H 0 4.480228 -2.086863 -1.974146
H 0 5.371496 -0.667283 -1.442545
H 0 5.038045 -3.245914 0.185588
H 0 5.931528 -1.828938 0.716107
H 0 6.634835 -3.533672 -1.754722
H 0 7.491947 -3.558757 -0.205107
H 0 7.535354 -2.107107 -1.218647
Free Energy (PCM/B3LYP/6-31G*) =-1757.658338
Number of imaginary frequencies = 0

2-3*R*-c6
B3LYP/6-31G* geometry
O 0 0.942501 -0.266570 -0.000242
C 0 -0.581256 1.583859 -0.098703
C 0 1.438614 1.655372 -1.538086
C 0 0.311185 2.499125 -0.868906
C 0 1.994920 0.644140 -0.487340
C 0 -0.220433 0.328412 0.243931
C 0 -1.318858 -0.407434 0.971533
N 0 -2.236740 0.717609 1.216025
C 0 -1.947343 1.818342 0.384567
O 0 -3.586008 0.407071 1.405111
C 0 -4.014325 0.800733 2.724403
C 0 -1.919194 -1.587573 0.178628
S 0 -2.527436 -1.112377 -1.496395
C 0 -4.182903 -1.899269 -1.575456
C 0 -5.219466 -1.316679 -0.602483
C 0 -6.592860 -1.961139 -0.846983
O 0 -5.370314 0.089755 -0.728982
O 0 -6.758033 -3.156396 -0.972604
O 0 -7.605965 -1.086961 -0.890962
O 0 -2.707022 2.755590 0.182951
O 0 0.968349 3.458579 -0.012016
O 0 2.499389 2.531020 -1.901166
C 0 0.939224 0.975830 -2.812856
C 0 3.142676 -0.231689 -0.967474
C 0 3.814477 -1.033792 0.156209
C 0 4.981807 -1.894179 -0.342923
C 0 5.659501 -2.704951 0.769158
C 0 6.825061 -3.562579 0.265854
H 0 -0.247330 3.032878 -1.645879
H 0 2.308674 1.229408 0.387333
H 0 -0.946896 -0.809150 1.922303
H 0 -5.064466 0.507056 2.779843
H 0 -3.915509 1.882118 2.847007
H 0 -3.432496 0.273691 3.485570
H 0 -2.743187 -2.017108 0.750977
H 0 -1.143664 -2.351625 0.079991
H 0 -4.104340 -2.978894 -1.429530
H 0 -4.502913 -1.720099 -2.606846
H 0 -4.939943 -1.575484 0.426280
H 0 -4.773841 0.504815 -0.072411
H 0 -7.196298 -0.197166 -0.776557
H 0 0.454437 4.280840 -0.024013
H 0 2.476959 3.237560 -1.224776
H 0 0.507313 1.723263 -3.485570
H 0 0.180485 0.216949 -2.601837
H 0 1.771604 0.493851 -3.333392
H 0 2.776167 -0.916425 -1.742607
H 0 3.873986 0.431045 -1.443536
H 0 4.177084 -0.340368 0.929071
H 0 3.069038 -1.676646 0.642308
H 0 4.620304 -2.581275 -1.122499
H 0 5.728796 -1.248422 -0.828130
H 0 4.912031 -3.350181 1.252418
H 0 6.019866 -2.018999 1.548994
H 0 6.489300 -4.280840 -0.492543
H 0 7.285255 -4.130675 1.082571
H 0 7.605965 -2.941109 -0.189774
Free Energy (PCM/B3LYP/6-31G*) =-1757.658178
Number of imaginary frequencies = 0

2-3*R*-c7
B3LYP/6-31G* geometry
O 0 -0.300073 -1.016927 -0.904441
C 0 -2.095154 -2.529870 -0.420324
C 0 -0.038415 -2.698775 0.949589
C 0 -1.314182 -3.444508 0.464241
C 0 0.618651 -1.984858 -0.273842
C 0 -1.554927 -1.442826 -1.012615
C 0 -2.561550 -0.693035 -1.848914
N 0 -3.800216 -1.379087 -1.445186
C 0 -3.504250 -2.608671 -0.807386
O 0 -4.767706 -1.440702 -2.466351
C 0 -6.025505 -0.947200 -1.985744
C 0 -2.609755 0.827812 -1.680810
S 0 -2.926381 1.395182 0.045862
C 0 -1.300675 2.107502 0.526264
C 0 -1.014523 3.516169 -0.018427
C 0 -2.101026 4.515202 0.366383
O 0 -0.866095 3.515579 -1.422876
O 0 -2.720079 5.158525 -0.459469
O 0 -2.282311 4.607229 1.688260
O 0 -4.315416 -3.505670 -0.619344
O 0 -0.850522 -4.618538 -0.234130
O 0 0.893167 -3.657385 1.439456
C 0 -0.369630 -1.743104 2.094951
C 0 1.908760 -1.234826 0.022732
C 0 2.652864 -0.769047 -1.237028
C 0 3.950844 -0.017918 -0.916066
C 0 4.707413 0.452796 -2.164925
C 0 6.000362 1.206807 -1.837719
H 0 -1.904958 -3.753120 1.334995
H 0 0.799387 -2.755980 -1.033796
H 0 -2.372621 -0.903804 -2.912763
H 0 -6.694211 -0.992582 -2.848867
H 0 -5.937551 0.086843 -1.635189
H 0 -6.411168 -1.582545 -1.182821

H 0 -3.414045 1.218144 -2.309700
H 0 -1.678228 1.281344 -2.021869
H 0 -0.504973 1.431569 0.204122
H 0 -1.301175 2.133124 1.619388
H 0 -0.077782 3.858734 0.449146
H 0 -1.550833 4.121925 -1.765823
H 0 -2.989258 5.261411 1.854149
H 0 -1.576683 -5.261411 -0.258917
H 0 0.730402 -4.461678 0.908291
H 0 -0.837542 -2.299882 2.912763
H 0 -1.054182 -0.951965 1.775992
H 0 0.542362 -1.278771 2.480368
H 0 1.687762 -0.371263 0.662818
H 0 2.545257 -1.910709 0.604839
H 0 2.882169 -1.642091 -1.865289
H 0 1.995291 -0.122815 -1.832967
H 0 3.720058 0.852221 -0.283481
H 0 4.607631 -0.665388 -0.316194
H 0 4.049081 1.097496 -2.764465
H 0 4.940335 -0.416524 -2.796189
H 0 5.795574 2.099374 -1.233377
H 0 6.515375 1.531961 -2.749068
H 0 6.694211 0.574673 -1.269709
Free Energy (PCM/B3LYP/6-31G*) = -1757.658084
Number of imaginary frequencies = 0

2-3*R*-c8
B3LYP/6-31G* geometry
O 0 1.387749 -0.968812 -0.881130
C 0 1.057663 -3.305410 -0.456252
C 0 2.710706 -2.097868 0.935057
C 0 2.260447 -3.490370 0.408479
C 0 2.720073 -1.092238 -0.258704
C 0 0.730834 -2.115182 -1.005570
C 0 -0.545939 -2.190103 -1.806534
N 0 -1.010348 -3.537247 -1.436098
C 0 0.041353 -4.283457 -0.844852
O 0 -1.722577 -4.190784 -2.458883
C 0 -2.994232 -4.638254 -1.968347
C 0 -1.566452 -1.078395 -1.547789
S 0 -2.182171 -1.113139 0.188623
C 0 -2.681440 0.636578 0.399413
C 0 -4.001413 1.015441 -0.310874
C 0 -4.287363 2.496317 -0.069029
O 0 -5.097686 0.255234 0.142154
O 0 -5.183288 2.879756 0.658113
O 0 -3.433349 3.305386 -0.708325
O 0 0.025152 -5.496787 -0.689055
O 0 3.387004 -4.024570 -0.317695
O 0 4.045339 -2.204708 1.419622
C 0 1.829090 -1.635675 2.094547
C 0 3.175158 0.320146 0.078803
C 0 3.434973 1.190145 -1.159173
C 0 3.897578 2.608945 -0.805394
C 0 4.176678 3.481494 -2.035944
C 0 4.634697 4.899506 -1.679587
H 0 2.037850 -4.143369 1.260889
H 0 3.368429 -1.521282 -1.033415
H 0 -0.303014 -2.178881 -2.879100
H 0 -3.587680 -3.800550 -1.587253
H 0 -2.865220 -5.392150 -1.185847
H 0 -3.489038 -5.083576 -2.834958
H 0 -2.398959 -1.189158 -2.247551
H 0 -1.084877 -0.113486 -1.734159
H 0 -1.867928 1.289079 0.067969
H 0 -2.807527 0.769682 1.478764
H 0 -3.890781 0.860492 -1.390459
H 0 -5.561026 0.815555 0.793287
H 0 -3.656915 4.228335 -0.476240
H 0 3.284741 -4.987914 -0.369947
H 0 4.460116 -2.893790 0.863998
H 0 1.820051 -2.398703 2.879100
H 0 0.799512 -1.449803 1.774257
H 0 2.228111 -0.713104 2.525851
H 0 2.423662 0.798783 0.719134
H 0 4.092392 0.228883 0.671310
H 0 4.195930 0.704530 -1.787375
H 0 2.521777 1.244851 -1.766184
H 0 3.133555 3.096112 -0.181456
H 0 4.805767 2.552221 -0.187044
H 0 3.269381 3.534361 -2.654533
H 0 4.941636 2.994965 -2.657638
H 0 3.875921 5.422701 -1.084377
H 0 4.822777 5.496787 -2.579287
H 0 5.561026 4.880589 -1.092083
Free Energy (PCM/B3LYP/6-31G*) =-1757.658005
Number of imaginary frequencies = 0

2-3*R*-c9
B3LYP/6-31G* geometry
O 0 0.167686 -0.410313 -0.818964
C 0 -0.880170 -2.542960 -0.502639
C 0 1.224141 -2.094414 0.723850
C 0 0.275846 -3.200004 0.176097
C 0 1.441272 -1.021774 -0.389079
C 0 -0.842466 -1.263425 -0.932025
C 0 -2.152480 -0.830038 -1.548709
N 0 -2.997088 -1.989358 -1.216005
C 0 -2.196155 -3.093488 -0.824914
O 0 -3.979279 -2.270268 -2.188968
C 0 -5.254055 -2.471763 -1.560554
C 0 -2.657100 0.502287 -0.986296
S 0 -4.159141 1.066457 -1.896562
C 0 -4.253005 2.834916 -1.409809
C 0 -4.768135 3.077130 0.018673
C 0 -3.666505 2.871738 1.076440
O 0 -5.916314 2.302692 0.347729
O 0 -2.559393 3.359117 0.988606
O 0 -4.044905 2.124050 2.121030
O 0 -2.596092 -4.247764 -0.759234
O 0 1.068857 -3.992604 -0.731098
O 0 2.489349 -2.681565 1.007880
C 0 0.685404 -1.499549 2.024700
C 0 2.367184 0.127140 -0.015601
C 0 2.770031 1.002679 -1.210350
C 0 3.691939 2.162653 -0.813077
C 0 4.111654 3.039399 -1.999612
C 0 5.023837 4.202639 -1.596093
H 0 -0.061045 -3.830716 1.007378
H 0 1.827366 -1.547334 -1.271704
H 0 -2.045221 -0.760685 -2.640057
H 0 -5.947389 -2.648695 -2.386449
H 0 -5.559612 -1.582260 -1.000356
H 0 -5.225966 -3.345568 -0.903739
H 0 -1.878694 1.259631 -1.112455
H 0 -2.873957 0.387135 0.078290
H 0 -4.954265 3.273455 -2.124506
H 0 -3.281117 3.316963 -1.538245
H 0 -5.062103 4.131308 0.091212
H 0 -5.855510 1.471466 -0.167870
H 0 -4.980121 1.868072 1.941727
H 0 0.648861 -4.862673 -0.820075
H 0 2.582848 -3.404597 0.356493
H 0 0.495035 -2.298940 2.747408
H 0 -0.243321 -0.943774 1.865840
H 0 1.422084 -0.818514 2.459964
H 0 1.882071 0.745132 0.750125
H 0 3.259451 -0.313373 0.443230
H 0 3.272588 0.378099 -1.963131
H 0 1.867909 1.402771 -1.691381
H 0 3.186259 2.787361 -0.061915
H 0 4.591268 1.762262 -0.321830
H 0 3.212091 3.433506 -2.493535
H 0 4.621917 2.415797 -2.747408
H 0 4.526639 4.862673 -0.874465
H 0 5.306044 4.809636 -2.464125
H 0 5.947389 3.837939 -1.129489
Free Energy (PCM/B3LYP/6-31G*) =-1757.657819
Number of imaginary frequencies = 0

2-3*R*-c10
B3LYP/6-31G* geometry
O 0 1.001053 -0.923439 -0.083583
C 0 -0.018187 -3.075330 -0.355551

C 0 2.400332 -2.999367 0.168964
C 0 1.253113 -3.853961 -0.442538
C 0 2.301813 -1.549035 -0.399593
C 0 -0.042986 -1.733835 -0.195659
C 0 -1.450417 -1.194562 -0.083656
N 0 -2.210775 -2.455770 -0.014611
C 0 -1.396507 -3.551541 -0.419742
O 0 -3.471577 -2.400276 -0.659636
C 0 -4.484361 -2.996910 0.173734
C 0 -1.637046 -0.281107 1.134614
S 0 -3.327495 0.388950 1.390839
C 0 -3.468372 1.644657 0.046471
C 0 -4.434687 1.259412 -1.074843
C 0 -5.887698 1.142546 -0.574961
O 0 -4.074786 0.105386 -1.823579
O 0 -6.357990 1.822668 0.311586
O 0 -6.619268 0.240968 -1.247549
O 0 -1.831653 -4.654865 -0.715989
O 0 1.639906 -4.125354 -1.804693
O 0 3.645839 -3.543872 -0.252899
C 0 2.368456 -3.043340 1.696491
C 0 3.374696 -0.583674 0.082338
C 0 3.401328 0.741096 -0.693347
C 0 4.491831 1.698471 -0.196951
C 0 4.535654 3.023804 -0.968464
C 0 5.623731 3.978320 -0.466052
H 0 1.175515 -4.799497 0.107009
H 0 2.330369 -1.629966 -1.493559
H 0 -1.711570 -0.658290 -1.005568
H 0 -4.538332 -2.486162 1.138644
H 0 -4.275316 -4.060262 0.306669
H 0 -5.416575 -2.861292 -0.379263
H 0 -0.928883 0.550370 1.075862
H 0 -1.421113 -0.844210 2.047270
H 0 -2.474681 1.831876 -0.367893
H 0 -3.824861 2.569020 0.505480
H 0 -4.433568 2.084928 -1.799538
H 0 -3.912071 -0.655189 -1.220672
H 0 -6.010472 -0.182814 -1.893095
H 0 1.160810 -4.913706 -2.105027
H 0 3.469614 -3.919754 -1.138017
H 0 2.378321 -4.083018 2.037701
H 0 1.479249 -2.551241 2.100826
H 0 3.251814 -2.544809 2.105027
H 0 3.228039 -0.385552 1.151519
H 0 4.337698 -1.096040 -0.021131
H 0 3.558557 0.531036 -1.761329
H 0 2.422067 1.231158 -0.614965
H 0 4.333830 1.906598 0.871729
H 0 5.471839 1.203889 -0.269889
H 0 3.555183 3.515760 -0.897114
H 0 4.695641 2.815750 -2.035969
H 0 5.469747 4.233246 0.589867
H 0 5.629968 4.913706 -1.037504
H 0 6.619268 3.525990 -0.555536
Free Energy (PCM/B3LYP/6-31G*) =-1757.657762
Number of imaginary frequencies = 0

2-3*R*-c11
B3LYP/6-31G* geometry
O 0 1.275954 -0.929235 -0.904540
C 0 0.848018 -3.239632 -0.425648
C 0 2.527705 -2.063268 0.960079
C 0 2.033722 -3.449666 0.457148
C 0 2.592207 -1.084029 -0.253981
C 0 0.577357 -2.052017 -1.009933
C 0 -0.692324 -2.095318 -1.824322
N 0 -1.220243 -3.409071 -1.422137
C 0 -0.206053 -4.183227 -0.797360
O 0 -1.942154 -4.061640 -2.439611
C 0 -3.235060 -4.452300 -1.956651
C 0 -1.667557 -0.935859 -1.603434
S 0 -2.295799 -0.910242 0.131718
C 0 -2.607066 0.881628 0.363300
C 0 -3.819897 1.397540 -0.435697
C 0 -4.099501 2.848089 -0.024935
O 0 -4.998945 0.646296 -0.185317
O 0 -3.351190 3.762912 -0.293945
O 0 -5.218795 3.019684 0.690465
O 0 -0.278532 -5.389045 -0.607395
O 0 3.148221 -4.037956 -0.244658
O 0 3.850390 -2.208336 1.466637
C 0 1.647442 -1.544387 2.096451
C 0 3.087676 0.319316 0.063962
C 0 3.395249 1.156992 -1.185302
C 0 3.902887 2.564618 -0.848836
C 0 4.226112 3.406698 -2.089792
C 0 4.729751 4.813349 -1.750223
H 0 1.777939 -4.076001 1.320090
H 0 3.239278 -1.550224 -1.007915
H 0 -0.440107 -2.121483 -2.894290
H 0 -3.730636 -4.907934 -2.817374
H 0 -3.807160 -3.584423 -1.611641
H 0 -3.142999 -5.185004 -1.149372
H 0 -2.501016 -1.027530 -2.303647
H 0 -1.147740 0.005890 -1.802606
H 0 -1.717172 1.461801 0.106982
H 0 -2.794863 0.992823 1.436074
H 0 -3.581339 1.402522 -1.506882
H 0 -4.735982 -0.283032 -0.039754
H 0 -5.647767 2.136300 0.755467
H 0 3.012946 -4.998005 -0.279478
H 0 4.248458 -2.921923 0.930099
H 0 1.594325 -2.291733 2.894290
H 0 0.631471 -1.322827 1.756121
H 0 2.076482 -0.631062 2.518336
H 0 2.342160 0.834180 0.682820
H 0 3.991847 0.209761 0.673180
H 0 4.147177 0.634086 -1.794099
H 0 2.492922 1.233069 -1.806005
H 0 3.149217 3.088050 -0.241973
H 0 4.801452 2.486630 -0.218917
H 0 3.328091 3.481164 -2.719486
H 0 4.980445 2.883767 -2.694735
H 0 3.982979 5.372378 -1.172722
H 0 4.949542 5.389045 -2.656770
H 0 5.647767 4.771201 -1.150986
Free Energy (PCM/B3LYP/6-31G*) =-1757.657666
Number of imaginary frequencies = 0

2-3*R*-c12
B3LYP/6-31G* geometry
O 0 1.512985 -1.104441 -0.474312
C 0 1.541224 -3.497518 -0.537134
C 0 3.523997 -2.341791 0.392158
C 0 2.997118 -3.656328 -0.249342
C 0 2.987048 -1.133857 -0.436912
C 0 0.937004 -2.291320 -0.627701
C 0 -0.549747 -2.417705 -0.869719
N 0 -0.716959 -3.872013 -0.689445
C 0 0.531570 -4.535754 -0.737083
O 0 -1.737135 -4.425205 -1.486084
C 0 -2.642021 -5.186543 -0.674277
C 0 -1.457558 -1.623651 0.083944
S 0 -1.622579 0.160818 -0.336780
C 0 -3.022799 0.130247 -1.528679
C 0 -4.421546 0.078686 -0.894149
C 0 -4.654063 1.221359 0.089933
O 0 -4.651266 -1.149675 -0.234803
O 0 -5.000864 1.032156 1.240086
O 0 -4.454439 2.427523 -0.451904
O 0 0.671630 -5.743458 -0.880643
O 0 3.777996 -3.857007 -1.445975
O 0 4.945142 -2.329175 0.304886
C 0 3.149376 -2.261301 1.871675
C 0 3.435485 0.238325 0.045144
C 0 3.127965 1.367471 -0.948538
C 0 3.586011 2.742331 -0.446214
C 0 3.304101 3.878358 -1.437826
C 0 3.760003 5.249749 -0.928694
H 0 3.173738 -4.491152 0.439472
H 0 3.305823 -1.291414 -1.475337
H 0 -0.783785 -2.160118 -1.911471
H 0 -2.130380 -6.038812 -0.217343

H 0 -3.409836 -5.541463 -1.366119
H 0 -3.098504 -4.563159 0.101941
H 0 -1.075871 -1.676206 1.107187
H 0 -2.460878 -2.049700 0.071702
H 0 -2.908749 -0.722982 -2.203144
H 0 -2.927437 1.040605 -2.126062
H 0 -5.151491 0.203817 -1.710520
H 0 -4.912941 -0.919772 0.677899
H 0 -4.627090 3.102303 0.233820
H 0 3.719226 -4.793916 -1.691141
H 0 5.151491 -2.823564 -0.512744
H 0 3.498621 -3.158525 2.391910
H 0 2.068486 -2.173274 2.013796
H 0 3.628167 -1.393977 2.334844
H 0 2.961175 0.453468 1.010915
H 0 4.515348 0.180406 0.221910
H 0 3.619413 1.147076 -1.907437
H 0 2.049357 1.392851 -1.151893
H 0 3.088858 2.964370 0.509737
H 0 4.664137 2.710121 -0.229109
H 0 2.227281 3.908231 -1.656746
H 0 3.803443 3.657384 -2.391910
H 0 3.250435 5.514004 0.006279
H 0 3.546883 6.038812 -1.659115
H 0 4.839262 5.259923 -0.731643
Free Energy (PCM/B3LYP/6-31G*) =-1757.657642
Number of imaginary frequencies = 0

2-3*R*-c13
B3LYP/6-31G* geometry
O 0 1.990636 -1.126372 -0.396419
C 0 1.920398 -3.517306 -0.549085
C 0 4.005495 -2.471394 0.280618
C 0 3.385662 -3.740344 -0.371461
C 0 3.462616 -1.210103 -0.462139
C 0 1.364605 -2.286510 -0.554322
C 0 -0.137692 -2.338009 -0.708848
N 0 -0.364064 -3.789353 -0.588015
C 0 0.853832 -4.499273 -0.739174
O 0 -1.435729 -4.262237 -1.374645
C 0 -2.289630 -5.107201 -0.589564
C 0 -0.865114 -1.484359 0.331893
S 0 -2.684174 -1.459519 0.030782
C 0 -3.183192 -0.068366 1.122582
C 0 -3.037703 1.331858 0.505262
C 0 -3.818277 1.471809 -0.798518
O 0 -1.685904 1.665410 0.268293
O 0 -3.294819 1.822788 -1.838613
O 0 -5.117560 1.184628 -0.664563
O 0 0.930949 -5.702050 -0.952698
O 0 4.072208 -3.918912 -1.627605
O 0 5.416543 -2.507351 0.094372
C 0 3.736808 -2.436904 1.784820
C 0 3.996444 0.122552 0.042691
C 0 3.645558 1.309113 -0.866084
C 0 4.192540 2.641820 -0.339592
C 0 3.851531 3.837896 -1.237582
C 0 4.395384 5.166925 -0.703048
H 0 3.578672 -4.607509 0.271343
H 0 3.703731 -1.334581 -1.525649
H 0 -0.418273 -2.013806 -1.720643
H 0 -3.101548 -5.388799 -1.264770
H 0 -2.691235 -4.564549 0.271989
H 0 -1.751024 -6.000956 -0.262082
H 0 -0.504253 -0.456680 0.266017
H 0 -0.666751 -1.874803 1.334769
H 0 -2.599723 -0.113015 2.046489
H 0 -4.230720 -0.244664 1.380391
H 0 -3.474625 2.046960 1.220874
H 0 -1.625601 1.877375 -0.683441
H 0 -5.546533 1.298391 -1.535393
H 0 3.968420 -4.844357 -1.899795
H 0 5.546533 -2.974994 -0.754186
H 0 4.078362 -3.369653 2.244064
H 0 2.673637 -2.307047 2.006432
H 0 4.286696 -1.611981 2.246249
H 0 3.612842 0.304870 1.054269
H 0 5.084644 0.022209 0.123939
H 0 4.044165 1.123179 -1.874020
H 0 2.555054 1.378536 -0.972117
H 0 3.796486 2.823335 0.670605
H 0 5.284988 2.569773 -0.230578
H 0 2.760020 3.906784 -1.348273
H 0 4.249810 3.658214 -2.246249
H 0 3.987739 5.389740 0.290856
H 0 4.135252 6.000956 -1.365043
H 0 5.488588 5.139930 -0.613913
Free Energy (PCM/B3LYP/6-31G*) =-1757.657470
Number of imaginary frequencies = 0

2-3*R*-c14
B3LYP/6-31G* geometry
O 0 -1.445548 -0.278784 1.429674
C 0 -1.961056 -1.910872 -0.243118
C 0 -3.701622 -0.282244 0.339532
C 0 -3.219670 -1.284730 -0.746707
C 0 -2.522836 0.569178 0.878427
C 0 -1.235295 -1.413826 0.779586
C 0 -0.101658 -2.337653 1.160073
N 0 -0.188290 -3.332903 0.070434
C 0 -1.430419 -3.217984 -0.589102
O 0 0.177179 -4.634099 0.459131
C 0 1.224220 -5.126341 -0.390114
C 0 1.278219 -1.696206 1.314054
S 0 1.875792 -0.999331 -0.284735
C 0 3.081740 0.259553 0.291079
C 0 4.437353 -0.296021 0.751057
C 0 5.104979 -1.156617 -0.317445
O 0 4.305647 -1.050980 1.936203
O 0 5.467708 -2.297709 -0.103596
O 0 5.262564 -0.518333 -1.482013
O 0 -1.939403 -4.080381 -1.305283
O 0 -4.252710 -2.265692 -0.912000
O 0 -4.161681 -1.040474 1.460474
C 0 -4.843805 0.599598 -0.181617
C 0 -1.897162 1.562634 -0.103161
C 0 -0.850473 2.483844 0.540772
C 0 -0.281670 3.510775 -0.446013
C 0 0.768522 4.437824 0.180115
C 0 1.327824 5.467972 -0.806444
H 0 -3.062144 -0.758342 -1.699013
H 0 -2.890927 1.095508 1.763351
H 0 -0.347502 -2.838752 2.107314
H 0 0.876188 -5.217421 -1.423731
H 0 1.467240 -6.114317 0.008201
H 0 2.105164 -4.477215 -0.347799
H 0 1.987627 -2.436583 1.686443
H 0 1.213993 -0.888865 2.049003
H 0 2.641926 0.839896 1.107683
H 0 3.231266 0.931976 -0.558018
H 0 5.099631 0.567674 0.927561
H 0 4.729061 -1.912136 1.754384
H 0 5.700090 -1.128877 -2.107314
H 0 -3.809645 -3.080152 -1.223507
H 0 -4.588492 -1.825152 1.065182
H 0 -5.700090 -0.039597 -0.417707
H 0 -4.571527 1.147442 -1.088599
H 0 -5.153426 1.316891 0.585568
H 0 -1.445557 1.015878 -0.941547
H 0 -2.700421 2.177608 -0.524174
H 0 -1.307945 3.009837 1.391299
H 0 -0.032928 1.879404 0.952319
H 0 0.164389 2.984479 -1.302788
H 0 -1.103601 4.117405 -0.854209
H 0 1.592094 3.831048 0.582636
H 0 0.324223 4.958480 1.040318
H 0 1.809661 4.977151 -1.661165
H 0 2.073386 6.114317 -0.329127
H 0 0.531093 6.111271 -1.200108
Free Energy (PCM/B3LYP/6-31G*) =-1757.657400
Number of imaginary frequencies = 0

2-3*R*-c15
B3LYP/6-31G* geometry

O 0 -0.611374 -0.719865 -0.816148
C 0 -2.840023 -1.365055 -0.219922
C 0 -0.964097 -2.335130 1.080915
C 0 -2.458586 -2.493876 0.680623
C 0 -0.141332 -1.985598 -0.198035
C 0 -1.944414 -0.601798 -0.876232
C 0 -2.609227 0.449883 -1.733403
N 0 -3.999376 0.322872 -1.261582
C 0 -4.185123 -0.896941 -0.570903
O 0 -4.959169 0.626031 -2.242993
C 0 -5.885986 1.602146 -1.744559
C 0 -2.065604 1.877376 -1.656463
S 0 -2.099917 2.560327 0.061118
C 0 -0.481130 3.424267 0.151774
C 0 0.712057 2.499272 0.412121
C 0 1.979557 3.321848 0.699731
O 0 1.003809 1.648222 -0.694600
O 0 2.000906 4.258104 1.469973
O 0 3.071701 2.906022 0.046507
O 0 -5.271305 -1.396875 -0.316702
O 0 -2.565740 -3.769852 0.019246
O 0 -0.487323 -3.583925 1.566985
C 0 -0.801538 -1.305929 2.198582
C 0 1.360025 -1.848730 0.005631
C 0 2.163110 -1.795608 -1.302480
C 0 3.670624 -1.645878 -1.062995
C 0 4.492888 -1.620914 -2.357733
C 0 5.997051 -1.462017 -2.113213
H 0 -3.075844 -2.501479 1.586423
H 0 -0.352522 -2.762149 -0.943011
H 0 -2.562897 0.132716 -2.786227
H 0 -5.369241 2.519857 -1.444408
H 0 -6.456293 1.200151 -0.902010
H 0 -6.555660 1.806931 -2.583219
H 0 -2.669561 2.512926 -2.309160
H 0 -1.036979 1.897857 -2.019774
H 0 -0.571778 4.127229 0.983424
H 0 -0.322911 4.001722 -0.763890
H 0 0.516583 1.905184 1.314587
H 0 0.491235 0.819457 -0.621581
H 0 2.784767 2.154313 -0.520728
H 0 -3.486380 -4.069271 0.084132
H 0 -1.002219 -4.258104 1.080617
H 0 -1.415695 -1.596937 3.056187
H 0 -1.104704 -0.304727 1.877998
H 0 0.240348 -1.264360 2.528530
H 0 1.569062 -0.956019 0.608710
H 0 1.677069 -2.711719 0.601110
H 0 1.971852 -2.711934 -1.878793
H 0 1.811329 -0.961456 -1.924050
H 0 3.857320 -0.721300 -0.496637
H 0 4.021655 -2.470613 -0.425285
H 0 4.137048 -0.800157 -2.996645
H 0 4.308995 -2.547287 -2.920099
H 0 6.215437 -0.526218 -1.583928
H 0 6.555660 -1.450027 -3.056187
H 0 6.388205 -2.286164 -1.503789
Free Energy (PCM/B3LYP/6-31G*) =-1757.657345
Number of imaginary frequencies = 0

2-3*R*-c16
B3LYP/6-31G* geometry
O 0 2.076304 -1.002989 -0.780908
C 0 1.859745 -3.363553 -0.427978
C 0 3.657351 -2.170098 0.787906
C 0 3.168609 -3.551294 0.265171
C 0 3.482591 -1.114014 -0.348218
C 0 1.437771 -2.163201 -0.880108
C 0 0.062510 -2.237254 -1.501784
N 0 -0.305569 -3.622787 -1.160282
C 0 0.833743 -4.356116 -0.745015
O 0 -1.105221 -4.255091 -2.134799
C 0 -2.237499 -4.881443 -1.513725
C 0 -0.888259 -1.171173 -0.951299
S 0 -2.489639 -1.177404 -1.861985
C 0 -3.242324 0.381162 -1.259023
C 0 -3.899478 0.271368 0.132262
C 0 -4.565580 1.600656 0.484261
O 0 -2.977188 -0.060016 1.148521
O 0 -4.141041 2.335053 1.355555
O 0 -5.627341 1.872792 -0.284144
O 0 0.878278 -5.576422 -0.659331
O 0 4.199296 -4.029296 -0.623159
O 0 5.047077 -2.261177 1.083367
C 0 2.933461 -1.781612 2.076781
C 0 3.943040 0.295416 -0.005763
C 0 3.995726 1.235144 -1.218775
C 0 4.457843 2.651486 -0.854607
C 0 4.513858 3.602218 -2.057247
C 0 4.970971 5.016903 -1.686843
H 0 3.072111 -4.242449 1.111037
H 0 4.031524 -1.488516 -1.221785
H 0 0.142710 -2.145380 -2.594080
H 0 -2.850786 -4.144434 -0.985904
H 0 -1.913607 -5.670879 -0.829635
H 0 -2.804844 -5.315873 -2.340667
H 0 -0.425366 -0.188761 -1.086724
H 0 -1.068504 -1.332045 0.112377
H 0 -4.000691 0.643791 -2.002389
H 0 -2.481861 1.169622 -1.255664
H 0 -4.671224 -0.506044 0.096273
H 0 -2.761165 0.778128 1.599835
H 0 -5.975654 2.748409 -0.023977
H 0 4.092557 -4.988764 -0.720719
H 0 5.397865 -2.916687 0.448762
H 0 3.039090 -2.581096 2.816577
H 0 1.867912 -1.601457 1.907699
H 0 3.372789 -0.872415 2.496746
H 0 3.278778 0.715839 0.759581
H 0 4.938386 0.208671 0.444256
H 0 4.673954 0.812681 -1.974429
H 0 3.003516 1.284091 -1.686156
H 0 3.782110 3.070074 -0.093969
H 0 5.451678 2.601459 -0.385253
H 0 3.521006 3.649402 -2.526865
H 0 5.190859 3.185675 -2.816577
H 0 4.293320 5.472654 -0.954166
H 0 5.000078 5.670879 -2.566074
H 0 5.975654 5.005672 -1.246126
Free Energy (PCM/B3LYP/6-31G*) =-1757.657327
Number of imaginary frequencies = 0

2-3*R*-c17
B3LYP/6-31G* geometry
O 0 1.334487 -0.605585 -1.279289
C 0 2.282210 -2.318075 0.091708
C 0 3.820715 -0.909028 -1.203517
C 0 3.703596 -1.868164 0.013096
C 0 2.636725 0.090924 -1.241862
C 0 1.264229 -1.716165 -0.559311
C 0 -0.040602 -2.457212 -0.359181
N 0 0.383139 -3.483860 0.619502
C 0 1.788405 -3.547993 0.683421
O 0 -0.278899 -4.716306 0.463888
C 0 -0.859515 -5.128374 1.709429
C 0 -1.209494 -1.619331 0.183386
S 0 -2.049065 -0.579253 -1.082467
C 0 -3.330531 -1.734541 -1.719018
C 0 -4.594944 -1.852519 -0.853110
C 0 -5.252709 -0.499390 -0.599078
O 0 -4.324020 -2.471394 0.387985
O 0 -5.487730 -0.087830 0.520897
O 0 -5.549557 0.157458 -1.725337
O 0 2.440816 -4.482068 1.151929
O 0 4.582350 -2.975715 -0.228461
O 0 3.690900 -1.693053 -2.391732
C 0 5.173153 -0.184876 -1.215863
C 0 2.587880 1.135007 -0.122545
C 0 1.477564 2.178464 -0.311464
C 0 1.456722 3.235033 0.800770
C 0 0.353266 4.284201 0.615514
C 0 0.323766 5.332355 1.732777
H 0 4.016944 -1.348930 0.930228
H 0 2.671616 0.586297 -2.216045

H 0 -0.337042 -2.964144 -1.286198
H 0 -0.084341 -5.300809 2.462225
H 0 -1.371506 -6.066663 1.482795
H 0 -1.579772 -4.389044 2.075286
H 0 -0.860133 -0.946253 0.971078
H 0 -1.965222 -2.278883 0.610241
H 0 -2.894528 -2.729482 -1.845988
H 0 -3.601299 -1.361704 -2.710224
H 0 -5.318436 -2.460761 -1.419867
H 0 -4.641353 -1.850831 1.072599
H 0 -5.968706 1.006409 -1.482613
H 0 4.208162 -3.733839 0.263744
H 0 4.135922 -2.535761 -2.177034
H 0 5.968706 -0.929699 -1.315636
H 0 5.354091 0.382135 -0.297733
H 0 5.233397 0.497256 -2.070296
H 0 2.465677 0.631180 0.845631
H 0 3.556027 1.647124 -0.089409
H 0 1.612973 2.674288 -1.283634
H 0 0.503380 1.675899 -0.353284
H 0 1.324204 2.737901 1.773178
H 0 2.433273 3.739623 0.846079
H 0 -0.620848 3.777668 0.562536
H 0 0.489950 4.784940 -0.353441
H 0 0.153254 4.864122 2.710224
H 0 -0.473857 6.066663 1.571252
H 0 1.273652 5.878590 1.788855
Free Energy (PCM/B3LYP/6-31G*) =-1757.657325
Number of imaginary frequencies = 0

2-3*R*-c18
B3LYP/6-31G* geometry
O 0 1.716055 -1.011851 -1.386254
C 0 1.160724 -2.684850 0.231116
C 0 3.154570 -3.046804 -1.155827
C 0 2.438347 -3.455954 0.162569
C 0 3.106887 -1.511743 -1.364249
C 0 0.889483 -1.613960 -0.543864
C 0 -0.525647 -1.117997 -0.346878
N 0 -0.957994 -1.980693 0.776566
C 0 -0.053667 -3.057872 0.929916
O 0 -2.317985 -2.368558 0.722833
C 0 -2.941772 -2.170131 2.003939
C 0 -0.588819 0.386388 -0.056179
S 0 -2.254122 1.105930 0.225291
C 0 -3.005950 1.102624 -1.458089
C 0 -3.991214 -0.054241 -1.735254
C 0 -5.177673 -0.022399 -0.771315
O 0 -3.391419 -1.325463 -1.735877
O 0 -5.435695 -0.900006 0.025349
O 0 -5.898475 1.106045 -0.902239
O 0 -0.291799 -4.099612 1.540906
O 0 2.186690 -4.865711 0.091374
O 0 2.412854 -3.598375 -2.245607
C 0 4.593640 -3.577346 -1.197434
C 0 3.907124 -0.667524 -0.369242
C 0 3.912649 0.832357 -0.698540
C 0 4.742953 1.650839 0.298336
C 0 4.755274 3.153420 -0.010527
C 0 5.578852 3.967248 0.992973
H 0 3.088287 -3.245372 1.023746
H 0 3.447601 -1.316687 -2.384572
H 0 -1.130938 -1.353739 -1.230373
H 0 -2.493245 -2.836571 2.744895
H 0 -3.990685 -2.424699 1.844105
H 0 -2.852545 -1.126074 2.315066
H 0 -0.106434 0.927058 -0.875950
H 0 -0.036659 0.606306 0.862448
H 0 -2.203341 1.069957 -2.200008
H 0 -3.526989 2.057361 -1.568611
H 0 -4.388699 0.110374 -2.744895
H 0 -3.283846 -1.623602 -0.808340
H 0 -6.624886 1.073204 -0.249395
H 0 1.405790 -5.034878 0.655103
H 0 2.101601 -4.463722 -1.916173
H 0 4.564820 -4.671123 -1.178190
H 0 5.190289 -3.239852 -0.344707
H 0 5.089488 -3.263958 -2.122006
H 0 3.515449 -0.822915 0.644892
H 0 4.939427 -1.035217 -0.365414
H 0 4.310739 0.977459 -1.713295
H 0 2.882848 1.209960 -0.712233
H 0 4.349650 1.493710 1.313718
H 0 5.776546 1.274309 0.309705
H 0 3.721762 3.527506 -0.025373
H 0 5.151678 3.311379 -1.023569
H 0 5.183546 3.857550 2.010517
H 0 5.569694 5.034878 0.744959
H 0 6.624886 3.636716 1.006831
Free Energy (PCM/B3LYP/6-31G*) =-1757.657308
Number of imaginary frequencies = 0

2-3*R*-c19
B3LYP/6-31G* geometry
O 0 1.267522 -0.932281 -0.912224
C 0 0.832168 -3.191500 -0.238381
C 0 2.290725 -1.810987 1.211269
C 0 1.888202 -3.260624 0.814621
C 0 2.492739 -0.973647 -0.091156
C 0 0.615392 -2.085953 -0.983545
C 0 -0.522769 -2.270185 -1.957247
N 0 -1.063812 -3.556085 -1.487649
C 0 -0.129160 -4.215336 -0.648828
O 0 -1.627022 -4.341584 -2.510028
C 0 -2.968666 -4.713706 -2.164217
C 0 -1.544747 -1.132790 -2.013606
S 0 -2.428165 -0.926820 -0.411428
C 0 -3.044267 0.787873 -0.667658
C 0 -4.158102 1.095007 0.349895
C 0 -4.616132 2.541700 0.172386
O 0 -3.745341 0.899872 1.685052
O 0 -4.370050 3.410578 0.985590
O 0 -5.277432 2.741779 -0.974338
O 0 -0.189319 -5.398015 -0.341524
O 0 3.095261 -3.895663 0.343589
O 0 3.539995 -1.856794 1.892937
C 0 1.263799 -1.195886 2.161523
C 0 2.922666 0.470275 0.123277
C 0 3.343832 1.184443 -1.168628
C 0 3.788773 2.632692 -0.928986
C 0 4.204936 3.361811 -2.212708
C 0 4.646219 4.808690 -1.968735
H 0 1.526003 -3.790013 1.703955
H 0 3.237389 -1.502115 -0.700321
H 0 -0.114471 -2.403369 -2.969797
H 0 -3.336344 -5.274854 -3.026824
H 0 -3.592862 -3.829550 -1.997573
H 0 -2.977421 -5.350893 -1.274652
H 0 -2.265386 -1.329168 -2.812342
H 0 -1.014739 -0.204761 -2.248668
H 0 -3.443067 0.876694 -1.682345
H 0 -2.218380 1.497368 -0.547916
H 0 -5.008527 0.431007 0.157794
H 0 -3.458719 1.773385 2.011894
H 0 -5.506165 3.690475 -1.032085
H 0 2.976606 -4.856374 0.408845
H 0 4.011510 -2.618723 1.502007
H 0 1.123116 -1.851028 3.026824
H 0 0.295785 -1.046229 1.674385
H 0 1.619564 -0.227963 2.525862
H 0 2.105190 1.023449 0.602408
H 0 3.758578 0.453925 0.831572
H 0 4.163785 0.624293 -1.641403
H 0 2.509210 1.172224 -1.881655
H 0 2.971751 3.188876 -0.445616
H 0 4.627529 2.643213 -0.217139
H 0 3.365855 3.349611 -2.922838
H 0 5.021386 2.806392 -2.695865
H 0 3.838205 5.398015 -1.517540
H 0 4.936182 5.301420 -2.903979
H 0 5.506165 4.851280 -1.288759
Free Energy (PCM/B3LYP/6-31G*) =-1757.657282
Number of imaginary frequencies = 0

2-3*R*-c20
B3LYP/6-31G* geometry

O	0	2.041114	-1.130560	-0.365064
C	0	1.925290	-3.522177	-0.457626
C	0	4.019483	-2.495013	0.375024
C	0	3.382311	-3.769998	-0.247933
C	0	3.511646	-1.246594	-0.412204
C	0	1.393011	-2.281973	-0.503149
C	0	-0.110209	-2.308502	-0.661855
N	0	-0.365930	-3.755741	-0.553616
C	0	0.845789	-4.487120	-0.654778
O	0	-1.398398	-4.220192	-1.394964
C	0	-2.334599	-5.004893	-0.644432
C	0	-0.810553	-1.452563	0.406292
S	0	-2.634903	-1.337771	0.279420
C	0	-2.811939	-0.287727	-1.226104
C	0	-4.051436	0.603450	-1.107495
C	0	-3.951974	1.606480	0.040186
O	0	-5.208742	-0.186600	-0.929150
O	0	-4.824068	1.724702	0.879540
O	0	-2.841511	2.350504	-0.006439
O	0	0.908156	-5.694171	-0.847592
O	0	4.085299	-4.003043	-1.485723
O	0	5.431842	-2.565089	0.209778
C	0	3.731282	-2.408888	1.873555
C	0	4.067336	0.090750	0.055684
C	0	3.768935	1.247729	-0.908405
C	0	4.328591	2.589636	-0.420132
C	0	4.055509	3.749986	-1.385792
C	0	4.607587	5.090348	-0.889383
H	0	3.545479	-4.620541	0.424744
H	0	3.761294	-1.409750	-1.468530
H	0	-0.387614	-1.968301	-1.667807
H	0	-1.851211	-5.897196	-0.235754
H	0	-3.098785	-5.296829	-1.369489
H	0	-2.787344	-4.412675	0.156644
H	0	-0.388691	-0.443100	0.387999
H	0	-0.616611	-1.881112	1.394054
H	0	-2.915612	-0.919239	-2.112138
H	0	-1.919430	0.333279	-1.339182
H	0	-4.128040	1.191541	-2.036935
H	0	-5.694555	0.216239	-0.183699
H	0	-2.862674	2.975691	0.744464
H	0	3.954929	-4.931195	-1.736545
H	0	5.564074	-3.063935	-0.620424
H	0	4.049416	-3.332688	2.366416
H	0	2.667765	-2.253059	2.075773
H	0	4.289848	-1.579705	2.316650
H	0	3.663447	0.320690	1.049588
H	0	5.150443	-0.031866	0.168073
H	0	4.193323	1.012726	-1.895350
H	0	2.683792	1.334942	-1.050831
H	0	3.896869	2.827336	0.563393
H	0	5.413513	2.496646	-0.262824
H	0	2.971807	3.837712	-1.548149
H	0	4.492809	3.514412	-2.366416
H	0	4.160794	5.370281	0.072701
H	0	4.398902	5.897196	-1.601416
H	0	5.694555	5.042678	-0.748188

Free Energy (PCM/B3LYP/6-31G*) =-1757.657230
Number of imaginary frequencies = 0

2-3*R*-c21
B3LYP/6-31G* geometry

O	0	-0.871272	-1.482272	-0.939029
C	0	-3.189188	-1.681852	-0.363695
C	0	-1.546239	-2.947301	0.989524
C	0	-3.034234	-2.856541	0.544190
C	0	-0.635521	-2.772534	-0.266791
C	0	-2.143579	-1.114031	-1.003853
C	0	-2.561573	0.073261	-1.836618
N	0	-3.955234	0.229481	-1.386591
C	0	-4.398843	-0.936983	-0.713431
O	0	-4.818468	0.727308	-2.379966
C	0	-5.507038	1.889973	-1.898835
C	0	-1.702359	1.330790	-1.699518
S	0	-1.746227	2.008706	0.015777
C	0	-0.276464	3.107424	-0.009446
C	0	1.070995	2.389649	0.211915
C	0	2.188842	3.425837	0.323100
O	0	1.391913	1.487960	-0.827978
O	0	3.051421	3.550312	-0.525224
O	0	2.094571	4.181890	1.423048
O	0	-5.571164	-1.193598	-0.473905
O	0	-3.329514	-4.106804	-0.113513
O	0	-1.309020	-4.247702	1.518754
C	0	-1.238700	-1.932232	2.089905
C	0	0.861422	-2.855794	-0.003316
C	0	1.710100	-2.880105	-1.282278
C	0	3.211505	-3.010692	-0.996506
C	0	4.072611	-3.028331	-2.265880
C	0	5.571779	-3.156998	-1.976561
H	0	-3.670509	-2.757325	1.431718
H	0	-0.933877	-3.541871	-0.990699
H	0	-2.561775	-0.213644	-2.898625
H	0	-6.158330	1.634939	-1.057246
H	0	-6.109448	2.230678	-2.744578
H	0	-4.800731	2.672512	-1.602088
H	0	-2.063866	2.090681	-2.398361
H	0	-0.666634	1.087093	-1.941256
H	0	-0.442981	3.824731	0.799180
H	0	-0.257524	3.661499	-0.953744
H	0	1.021009	1.832776	1.154896
H	0	2.059226	1.940894	-1.378571
H	0	2.821941	4.834756	1.407394
H	0	-4.290943	-4.235776	-0.101078
H	0	-1.912072	-4.834756	1.021482
H	0	-1.955491	-2.044357	2.909352
H	0	-1.286639	-0.904058	1.719104
H	0	-0.236758	-2.106796	2.491987
H	0	1.166240	-2.008273	0.623050
H	0	1.034120	-3.767070	0.580274
H	0	1.385854	-3.716519	-1.918601
H	0	1.527338	-1.962838	-1.856539
H	0	3.532560	-2.178125	-0.353002
H	0	3.394444	-3.930031	-0.420570
H	0	3.887755	-2.109345	-2.840023
H	0	3.751557	-3.859857	-2.909352
H	0	5.929922	-2.320479	-1.363642
H	0	6.158330	-3.167078	-2.902524
H	0	5.791589	-4.083866	-1.432235

Free Energy (PCM/B3LYP/6-31G*) =-1757.657198
Number of imaginary frequencies = 0

2-3*R*-c22
B3LYP/6-31G* geometry

O	0	1.866272	-0.799462	-0.564301
C	0	2.513117	-3.102815	-0.419973
C	0	4.024534	-1.412559	0.576477
C	0	3.922206	-2.856420	0.006704
C	0	3.287342	-0.435906	-0.393451
C	0	1.640211	-2.103298	-0.668597
C	0	0.273523	-2.622269	-1.052110
N	0	0.451232	-4.060113	-0.788283
C	0	1.829091	-4.373767	-0.652048
O	0	-0.264702	-4.888071	-1.679250
C	0	-0.979586	-5.897507	-0.950927
C	0	-0.852676	-1.961421	-0.250749
S	0	-2.501623	-2.527544	-0.843851
C	0	-3.608813	-1.478752	0.173085
C	0	-3.635248	-0.002090	-0.269665
C	0	-4.742627	0.720698	0.506196
O	0	-3.913134	0.148804	-1.654024
O	0	-4.679308	0.932831	1.697897
O	0	-5.806012	1.065488	-0.231533
O	0	2.286954	-5.507267	-0.698638
O	0	4.848167	-2.916232	-1.097355
O	0	5.396223	-1.033515	0.609108
C	0	3.490957	-1.344863	2.007212
C	0	3.313528	1.029688	0.014972
C	0	2.812680	1.983247	-1.079009
C	0	2.843870	3.453405	-0.642750
C	0	2.350784	4.421386	-1.725897

C 0 2.376479 5.887164 -1.280280
H 0 4.232513 -3.571788 0.777675
H 0 3.743826 -0.561223 -1.383600
H 0 0.107316 -2.470629 -2.127647
H 0 -1.691727 -5.445981 -0.253150
H 0 -0.284851 -6.553313 -0.418890
H 0 -1.515422 -6.465555 -1.715480
H 0 -0.773630 -0.878158 -0.376272
H 0 -0.748788 -2.206490 0.810216
H 0 -3.337503 -1.547766 1.229422
H 0 -4.601366 -1.921753 0.043518
H 0 -2.686815 0.483976 -0.007891
H 0 -3.499728 -0.600678 -2.124847
H 0 -5.603196 0.798346 -1.156864
H 0 5.057286 -3.848476 -1.266732
H 0 5.806012 -1.505184 -0.142691
H 0 3.995090 -2.092686 2.626930
H 0 2.412856 -1.524490 2.050451
H 0 3.691842 -0.359492 2.436638
H 0 2.713094 1.160943 0.923791
H 0 4.349890 1.271391 0.275998
H 0 3.429851 1.856142 -1.980284
H 0 1.788439 1.708961 -1.363510
H 0 2.228140 3.577068 0.260554
H 0 3.869518 3.725409 -0.352217
H 0 1.327765 4.145376 -2.018614
H 0 2.968911 4.301336 -2.626930
H 0 1.738182 6.044546 -0.401920
H 0 2.020754 6.553313 -2.074768
H 0 3.392718 6.201163 -1.011285
Free Energy (PCM/B3LYP/6-31G*) =-1757.657014
Number of imaginary frequencies = 0

2-3*R*-c23
B3LYP/6-31G* geometry
O 0 1.470598 -0.486424 -0.138960
C 0 1.676220 -2.865117 -0.317470
C 0 3.538832 -1.617677 0.733994
C 0 3.129888 -2.933669 0.014494
C 0 2.941401 -0.413158 -0.058171
C 0 0.987987 -1.702293 -0.369253
C 0 -0.477587 -1.922933 -0.666044
N 0 -0.542471 -3.392945 -0.567952
C 0 0.752744 -3.961306 -0.606153
O 0 -1.494229 -3.972950 -1.427798
C 0 -2.363641 -4.842872 -0.689858
C 0 -1.467032 -1.249280 0.299078
S 0 -1.732801 0.543936 -0.022353
C 0 -3.100707 0.498722 -1.250716
C 0 -4.507644 0.333315 -0.653836
C 0 -4.826256 1.411568 0.377681
O 0 -4.678867 -0.936553 -0.058857
O 0 -5.168043 1.147794 1.514524
O 0 -4.702241 2.651715 -0.107109
O 0 0.986859 -5.145971 -0.808122
O 0 3.962172 -3.019673 -1.160876
O 0 4.957617 -1.500977 0.698382
C 0 3.112114 -1.634960 2.201466
C 0 3.278879 0.963302 0.495805
C 0 2.887266 2.117308 -0.438278
C 0 3.264899 3.488986 0.138374
C 0 2.827182 4.686490 -0.721012
C 0 3.521222 4.777111 -2.085627
H 0 3.344386 -3.785928 0.670659
H 0 3.298155 -0.501274 -1.092483
H 0 -0.700807 -1.626168 -1.699566
H 0 -3.079272 -5.216080 -1.426570
H 0 -2.891569 -4.298853 0.100707
H 0 -1.802865 -5.677942 -0.259697
H 0 -1.115071 -1.340759 1.330297
H 0 -2.439246 -1.737588 0.226329
H 0 -2.921780 -0.309503 -1.965415
H 0 -3.044913 1.443757 -1.797132
H 0 -5.226051 0.453893 -1.480616
H 0 -4.942179 -0.766212 0.866352
H 0 -4.922546 3.281805 0.606827
H 0 3.960288 -3.941032 -1.465162
H 0 5.226051 -1.939890 -0.132660
H 0 3.505968 -2.530604 2.691689
H 0 2.023776 -1.627376 2.309453
H 0 3.514846 -0.760889 2.720669
H 0 2.788327 1.092663 1.468729
H 0 4.359616 0.979231 0.676351
H 0 3.375425 1.969581 -1.411116
H 0 1.805595 2.086411 -0.625009
H 0 2.813525 3.588118 1.135937
H 0 4.353842 3.531117 0.290906
H 0 3.025437 5.609458 -0.159593
H 0 1.738078 4.646578 -0.866226
H 0 4.611801 4.819216 -1.970117
H 0 3.208688 5.677942 -2.626418
H 0 3.287937 3.915065 -2.720669
Free Energy (PCM/B3LYP/6-31G*) =-1757.656991
Number of imaginary frequencies = 0

2-3*R*-c24
B3LYP/6-31G* geometry
O 0 0.372795 -0.380886 -0.708074
C 0 -0.853848 -2.359359 -0.137654
C 0 0.972138 -1.654783 1.380182
C 0 0.091818 -2.847680 0.908809
C 0 1.484583 -0.880243 0.124720
C 0 -0.644950 -1.221923 -0.836123
C 0 -1.757664 -0.942055 -1.814593
N 0 -2.731534 -1.976790 -1.425212
C 0 -2.100999 -2.956942 -0.605959
O 0 -3.423987 -2.509379 -2.538613
C 0 -4.828890 -2.583073 -2.271030
C 0 -2.290998 0.496333 -1.802210
S 0 -2.770374 1.167485 -0.161297
C 0 -4.531713 0.643559 -0.019512
C 0 -5.294827 1.617490 0.883203
C 0 -5.361675 3.031494 0.309422
O 0 -4.705835 1.668649 2.166221
O 0 -5.026381 4.010679 0.948211
O 0 -5.859098 3.072321 -0.931346
O 0 -2.578148 -4.057041 -0.363490
O 0 1.006361 -3.842057 0.401199
O 0 2.111540 -2.172321 2.059684
C 0 0.212322 -0.761592 2.360012
C 0 2.376361 0.317088 0.420133
C 0 3.068099 0.892641 -0.823662
C 0 3.961587 2.096863 -0.500102
C 0 4.659715 2.686443 -1.732197
C 0 5.544482 3.893377 -1.402877
H 0 -0.447597 -3.259866 1.769697
H 0 2.019721 -1.604229 -0.503093
H 0 -1.409063 -1.158105 -2.835517
H 0 -5.250597 -1.592092 -2.073266
H 0 -5.031165 -3.251585 -1.429428
H 0 -5.265590 -2.994741 -3.184476
H 0 -3.130904 0.585275 -2.496691
H 0 -1.496573 1.156100 -2.163810
H 0 -4.591052 -0.360677 0.404621
H 0 -4.984605 0.633295 -1.013983
H 0 -6.333873 1.255596 0.953310
H 0 -4.608438 2.617057 2.378224
H 0 -5.889182 4.006680 -1.216334
H 0 0.560406 -4.703323 0.424404
H 0 2.294803 -3.029187 1.626233
H 0 -0.621092 -0.242959 1.877025
H 0 0.886195 -0.011192 2.783141
H 0 -0.179722 -1.365341 3.184476
H 0 1.779364 1.099088 0.905624
H 0 3.126953 -0.012827 1.147109
H 0 3.672656 0.106576 -1.299214
H 0 2.309828 1.188758 -1.560194
H 0 3.355858 2.878904 -0.018502
H 0 4.719807 1.799618 0.239513
H 0 3.900833 2.978979 -2.471753
H 0 5.268018 1.906226 -2.211427
H 0 4.956747 4.703323 -0.953066
H 0 6.028708 4.291964 -2.301868
H 0 6.333873 3.622584 -0.690715

Free Energy (PCM/B3LYP/6-31G*) =-1757.656890
Number of imaginary frequencies = 0

2-3*R*-c25
B3LYP/6-31G* geometry
O 0 1.265153 -1.637313 -1.193946
C 0 -0.469904 -2.236077 0.345366
C 0 1.785692 -3.099071 0.775696
C 0 0.412428 -2.772121 1.425088
C 0 2.259527 -1.946819 -0.146286
C 0 -0.003170 -1.751932 -0.823733
C 0 -1.121642 -1.359771 -1.757421
N 0 -2.258216 -1.973282 -1.049334
C 0 -1.922680 -2.285357 0.282756
O 0 -3.511845 -1.390456 -1.268551
C 0 -4.426666 -2.346847 -1.844315
C 0 -1.258523 0.158465 -1.997190
S 0 -1.472906 1.173117 -0.475224
C 0 -3.176523 1.876269 -0.651606
C 0 -4.077979 1.611853 0.560786
C 0 -3.416025 2.115988 1.858613
O 0 -4.467345 0.260480 0.754120
O 0 -3.041103 3.260013 2.012927
O 0 -3.293483 1.177715 2.802331
O 0 -2.726721 -2.609719 1.154853
O 0 -0.101111 -3.996895 1.962176
O 0 1.603834 -4.220979 -0.092429
C 0 2.838832 -3.440492 1.837991
C 0 2.646903 -0.636507 0.544237
C 0 3.222001 0.413070 -0.418340
C 0 3.626432 1.710561 0.292648
C 0 4.204413 2.766425 -0.658704
C 0 4.596980 4.066363 0.050893
H 0 0.546519 -2.043838 2.237497
H 0 3.104877 -2.331848 -0.723037
H 0 -0.986553 -1.832371 -2.738370
H 0 -4.583529 -3.180459 -1.155954
H 0 -4.041346 -2.705582 -2.802331
H 0 -5.355673 -1.792848 -1.991009
H 0 -2.101105 0.344647 -2.665519
H 0 -0.350453 0.498145 -2.501952
H 0 -3.640583 1.467921 -1.552295
H 0 -3.081871 2.957137 -0.773315
H 0 -4.982787 2.219757 0.419974
H 0 -4.060662 -0.327355 0.080508
H 0 -3.671234 0.357251 2.398714
H 0 -1.062561 -3.873096 2.070239
H 0 0.970837 -4.798331 0.375095
H 0 2.519990 -4.340661 2.371988
H 0 2.968751 -2.640118 2.572627
H 0 3.804722 -3.646621 1.365200
H 0 1.774126 -0.222442 1.066485
H 0 3.394030 -0.863424 1.312831
H 0 2.484288 0.639230 -1.197929
H 0 4.097221 -0.011019 -0.931697
H 0 2.750666 2.129786 0.809704
H 0 4.365010 1.484373 1.075912
H 0 3.467726 2.985704 -1.444465
H 0 5.082638 2.348823 -1.171211
H 0 3.730365 4.525914 0.542259
H 0 5.007939 4.798331 -0.653965
H 0 5.355673 3.882791 0.821840
Free Energy (PCM/B3LYP/6-31G*) =-1757.656880
Number of imaginary frequencies = 0

2-3*R*-c26
B3LYP/6-31G* geometry
O 0 0.909081 -0.941527 -0.438004
C 0 3.208658 -1.404570 0.033226
C 0 1.736241 -3.310381 -0.549476
C 0 3.065081 -2.886896 0.138785
C 0 0.608284 -2.340536 -0.081327
C 0 2.171238 -0.580606 -0.238151
C 0 2.606324 0.863819 -0.341034
N 0 4.069782 0.700201 -0.264049
C 0 4.415102 -0.593924 0.188595
O 0 4.725855 1.773384 0.367283
C 0 5.800531 2.249748 -0.455180
C 0 2.207242 1.606808 -1.629151
S 0 0.456414 2.150872 -1.721051
C 0 0.505866 3.657213 -0.654964
C 0 -0.779951 3.772427 0.166962
C 0 -0.970591 2.604601 1.131795
O 0 -1.900342 3.850286 -0.689940
O 0 -1.997082 1.953827 1.174643
O 0 0.083071 2.406337 1.933771
O 0 5.526119 -0.917773 0.588900
O 0 2.966503 -3.335498 1.506343
O 0 1.393095 -4.621172 -0.111993
C 0 1.888358 -3.353863 -2.069435
C 0 -0.782260 -2.633440 -0.626386
C 0 -1.895073 -1.874556 0.111299
C 0 -3.283817 -2.113809 -0.493875
C 0 -4.405152 -1.374580 0.247728
C 0 -5.787737 -1.596929 -0.374152
H 0 3.904231 -3.398858 -0.348012
H 0 0.596774 -2.370602 1.015898
H 0 2.260019 1.420198 0.539866
H 0 5.435242 2.573343 -1.435750
H 0 6.566355 1.478215 -0.577254
H 0 6.214749 3.102186 0.088759
H 0 2.372508 0.966003 -2.499734
H 0 2.851017 2.483249 -1.739652
H 0 0.602461 4.545435 -1.284663
H 0 1.372129 3.608129 0.008640
H 0 -0.704704 4.684742 0.781423
H 0 -2.541980 3.195243 -0.353975
H 0 -0.122390 1.657334 2.527278
H 0 3.861823 -3.363949 1.879253
H 0 1.745785 -4.684742 0.797366
H 0 2.720811 -4.010752 -2.339774
H 0 2.076333 -2.361325 -2.488767
H 0 0.979039 -3.753486 -2.527278
H 0 -0.807055 -2.386301 -1.695232
H 0 -0.945869 -3.713444 -0.540768
H 0 -1.899359 -2.182439 1.167280
H 0 -1.675950 -0.799122 0.104970
H 0 -3.278796 -1.799305 -1.548135
H 0 -3.500559 -3.192546 -0.498389
H 0 -4.178409 -0.299287 0.264956
H 0 -4.419360 -1.699763 1.297790
H 0 -5.813921 -1.249130 -1.414391
H 0 -6.566355 -1.057498 0.177579
H 0 -6.055350 -2.660980 -0.374327
Free Energy (PCM/B3LYP/6-31G*) = -1757.656832
Number of imaginary frequencies = 0

2-3*R*-c27
B3LYP/6-31G* geometry
O 0 0.453823 -0.018969 -0.450314
C 0 2.258658 1.469581 0.061526
C 0 0.430256 1.264413 1.718984
C 0 1.579109 2.172218 1.192235
C 0 -0.390550 0.738218 0.499433
C 0 1.663259 0.478806 -0.643179
C 0 2.556344 -0.076117 -1.723361
N 0 3.847991 0.555837 -1.370907
C 0 3.591044 1.673477 -0.479727
O 0 4.538528 0.955388 -2.548667
C 0 5.931968 0.629391 -2.475906
C 0 2.585196 -1.603266 -1.845184
S 0 2.801020 -2.541584 -0.275654
C 0 4.573537 -3.056819 -0.281363
C 0 5.460009 -2.289796 0.702012
C 0 4.923826 -2.414676 2.142498
O 0 5.673595 -0.920399 0.386274
O 0 4.591037 -3.468887 2.642540
O 0 4.881976 -1.254198 2.806928
O 0 4.410997 2.547500 -0.259300
O 0 0.956654 3.403991 0.776555
O 0 -0.446601 2.056300 2.512051
C 0 0.976880 0.145566 2.604587
C 0 -1.568249 -0.161089 0.843947
C 0 -2.493276 -0.449477 -0.347031

C 0 -3.682271 -1.342044 0.030786
C 0 -4.614232 -1.646177 -1.149206
C 0 -5.800317 -2.536932 -0.764949
H 0 2.281079 2.376395 2.008909
H 0 -0.736475 1.616937 -0.058886
H 0 2.239679 0.326385 -2.696101
H 0 6.413804 1.146216 -1.641663
H 0 6.344604 0.987427 -3.422059
H 0 6.087481 -0.450662 -2.388463
H 0 3.357190 -1.903478 -2.558296
H 0 1.623363 -1.927089 -2.251752
H 0 4.970681 -2.975855 -1.296260
H 0 4.584153 -4.110744 0.003941
H 0 6.446072 -2.773618 0.699894
H 0 4.937757 -0.572649 -0.169190
H 0 5.198966 -0.574906 2.164708
H 0 1.619924 4.110744 0.816581
H 0 -0.414530 2.946887 2.109952
H 0 1.562118 0.578038 3.422059
H 0 1.615511 -0.543980 2.045076
H 0 0.153734 -0.425166 3.043263
H 0 -1.192784 -1.104927 1.258128
H 0 -2.129272 0.340145 1.640748
H 0 -2.865460 0.501231 -0.755544
H 0 -1.918289 -0.928428 -1.150139
H 0 -3.308139 -2.288062 0.449493
H 0 -4.258563 -0.859435 0.834010
H 0 -4.036886 -2.129570 -1.950081
H 0 -4.986838 -0.701066 -1.569146
H 0 -5.458782 -3.502563 -0.371750
H 0 -6.446072 -2.737544 -1.627656
H 0 -6.415726 -2.063081 0.009836
Free Energy (PCM/B3LYP/6-31G*) =-1757.656733
Number of imaginary frequencies = 0

2-3*R*-c28
B3LYP/6-31G* geometry
O 0 -0.788834 0.075022 -0.609152
C 0 0.185747 2.259194 -0.434970
C 0 -1.686048 1.700092 1.085817
C 0 -0.886743 2.854101 0.415458
C 0 -2.013926 0.623627 0.003448
C 0 0.152550 0.977091 -0.858723
C 0 1.370984 0.616216 -1.673196
N 0 2.194134 1.816090 -1.455552
C 0 1.415081 2.877137 -0.938432
O 0 3.045857 2.133147 -2.526603
C 0 4.406881 2.202024 -2.074686
C 0 2.061145 -0.700822 -1.314626
S 0 2.684630 -0.692607 0.420065
C 0 3.287156 -2.419045 0.579076
C 0 4.667393 -2.685358 -0.041820
C 0 5.727827 -1.713800 0.468448
O 0 4.621074 -2.615748 -1.451696
O 0 6.404999 -1.033958 -0.279829
O 0 5.839753 -1.712384 1.800478
O 0 1.765542 4.049601 -0.912768
O 0 -1.846339 3.609040 -0.353673
O 0 -2.921087 2.222112 1.565316
C 0 -0.928002 1.125683 2.282248
C 0 -2.827425 -0.567187 0.489755
C 0 -3.337966 -1.464966 -0.646516
C 0 -4.165866 -2.650375 -0.130784
C 0 -4.648184 -3.616322 -1.225785
C 0 -5.655004 -3.008009 -2.209877
H 0 -0.463092 3.498508 1.195071
H 0 -2.545406 1.136548 -0.808680
H 0 1.092041 0.561902 -2.735868
H 0 4.532070 3.008357 -1.345482
H 0 4.989208 2.421178 -2.973072
H 0 4.731810 1.250621 -1.640195
H 0 2.893269 -0.879341 -1.995959
H 0 1.341280 -1.517235 -1.430094
H 0 2.559122 -3.103289 0.133755
H 0 3.326492 -2.621233 1.652721
H 0 4.976728 -3.695437 0.274124
H 0 5.339628 -2.008468 -1.714336
H 0 6.535961 -1.072424 2.047560
H 0 -1.475225 4.490216 -0.518630
H 0 -3.148766 2.941342 0.943806
H 0 -0.659074 1.930882 2.973072
H 0 -0.014881 0.606947 1.975958
H 0 -1.561566 0.416296 2.822215
H 0 -2.221435 -1.159628 1.186599
H 0 -3.673588 -0.166780 1.059384
H 0 -3.937877 -0.857623 -1.337676
H 0 -2.484622 -1.842530 -1.225334
H 0 -3.561496 -3.213132 0.594930
H 0 -5.035681 -2.270411 0.425779
H 0 -5.106292 -4.490216 -0.743329
H 0 -3.777884 -3.995844 -1.780270
H 0 -6.535961 -2.621478 -1.681807
H 0 -6.001078 -3.757155 -2.931387
H 0 -5.219756 -2.179310 -2.779536
Free Energy (PCM/B3LYP/6-31G*) =-1757.656681
Number of imaginary frequencies = 0

2-3*R*-c29
B3LYP/6-31G* geometry
O 0 1.242168 -0.873372 -0.082882
C 0 0.173918 -3.001588 -0.350953
C 0 2.593542 -2.980873 0.171062
C 0 1.426589 -3.809289 -0.437862
C 0 2.527470 -1.528766 -0.398394
C 0 0.178693 -1.659409 -0.194443
C 0 -1.215274 -1.087544 -0.084945
N 0 -2.005540 -2.332285 -0.017982
C 0 -1.214426 -3.446948 -0.413901
O 0 -3.260778 -2.257709 -0.669993
C 0 -4.295226 -2.801373 0.170261
C 0 -1.375558 -0.168554 1.133445
S 0 -3.043347 0.546999 1.411150
C 0 -3.193712 1.804010 0.069980
C 0 -4.070263 1.370843 -1.119145
C 0 -5.512105 1.140094 -0.649622
O 0 -3.537009 0.313942 -1.880291
O 0 -6.105985 1.902054 0.086258
O 0 -6.055750 0.015662 -1.148641
O 0 -1.671801 -4.543062 -0.705917
O 0 1.806997 -4.091639 -1.799821
O 0 3.826369 -3.553905 -0.251256
C 0 2.562207 -3.023095 1.698676
C 0 3.622975 -0.589098 0.083352
C 0 3.680127 0.736371 -0.689524
C 0 4.795229 1.664706 -0.192709
C 0 4.868647 2.992678 -0.957476
C 0 5.982360 3.917000 -0.454746
H 0 1.327588 -4.752360 0.112764
H 0 2.554572 -1.611417 -1.492285
H 0 -1.467572 -0.540485 -1.001797
H 0 -4.118271 -3.866344 0.335998
H 0 -5.217892 -2.652423 -0.394151
H 0 -4.342789 -2.261726 1.119715
H 0 -0.647701 0.645307 1.065411
H 0 -1.164917 -0.733252 2.046647
H 0 -2.193548 2.058583 -0.289777
H 0 -3.630614 2.694703 0.527101
H 0 -4.120285 2.233487 -1.797923
H 0 -3.619903 -0.526764 -1.383606
H 0 -6.965889 -0.044821 -0.797345
H 0 1.292430 -4.855005 -2.106177
H 0 3.641102 -3.927539 -1.135370
H 0 2.548908 -4.062542 2.040517
H 0 1.684587 -2.510859 2.103352
H 0 3.456956 -2.544458 2.106177
H 0 3.482217 -0.388998 1.152957
H 0 4.573551 -1.123817 -0.021853
H 0 3.829595 0.525246 -1.758411
H 0 2.713365 1.249990 -0.607357
H 0 4.646265 1.871262 0.877589
H 0 5.762638 1.146825 -0.271774
H 0 3.901036 3.508421 -0.879030
H 0 5.018774 2.786490 -2.026770
H 0 5.839253 4.170043 0.603165

H 0 6.009578 4.855005 -1.021264
H 0 6.965889 3.440432 -0.550907
Free Energy (PCM/B3LYP/6-31G*) =-1757.656673
Number of imaginary frequencies = 0

2-3*R*-c30
B3LYP/6-31G* geometry
O 0 1.084807 -1.265988 0.171019
C 0 0.382886 -3.496012 -0.353506
C 0 2.781239 -3.121093 0.138479
C 0 1.750444 -4.061841 -0.548694
C 0 2.446667 -1.646882 -0.249391
C 0 0.164671 -2.202696 -0.029732
C 0 -1.302906 -1.901001 0.165271
N 0 -1.872091 -3.255785 0.058872
C 0 -0.913259 -4.158758 -0.471082
O 0 -3.126264 -3.289837 -0.592246
C 0 -4.054078 -4.083369 0.160519
C 0 -1.561152 -1.177264 1.493190
S 0 -3.315229 -0.838756 1.925742
C 0 -4.017592 -0.011654 0.448335
C 0 -3.438706 1.378933 0.135768
C 0 -4.243060 2.046682 -0.979184
O 0 -2.084311 1.307901 -0.264372
O 0 -3.754122 2.329242 -2.056580
O 0 -5.517893 2.270775 -0.643996
O 0 -1.180587 -5.272007 -0.905076
O 0 2.129795 -4.120966 -1.939460
O 0 4.077997 -3.419182 -0.368648
C 0 2.811727 -3.345080 1.650142
C 0 3.387032 -0.592130 0.315466
C 0 3.179381 0.801923 -0.293518
C 0 4.147410 1.848718 0.271810
C 0 3.955695 3.244557 -0.335227
C 0 4.924294 4.287237 0.232445
H 0 1.829048 -5.064341 -0.111246
H 0 2.441723 -1.597380 -1.345824
H 0 -1.665609 -1.282747 -0.664431
H 0 -3.724062 -5.125368 0.202227
H 0 -4.994241 -4.014279 -0.392857
H 0 -4.181333 -3.678969 1.168947
H 0 -1.002114 -0.240466 1.482522
H 0 -1.201497 -1.791090 2.325044
H 0 -5.082146 0.078547 0.684315
H 0 -3.919408 -0.669526 -0.420239
H 0 -3.522062 2.007716 1.032087
H 0 -2.066052 1.584482 -1.201378
H 0 -5.967109 2.683633 -1.407669
H 0 1.739139 -4.921025 -2.325044
H 0 3.926476 -3.717951 -1.287008
H 0 2.988655 -4.403392 1.865214
H 0 1.874225 -3.043698 2.126162
H 0 3.625300 -2.768417 2.098956
H 0 3.264201 -0.543239 1.404685
H 0 4.408947 -0.936656 0.121390
H 0 3.304500 0.741406 -1.384511
H 0 2.145811 1.127546 -0.117892
H 0 4.022806 1.908238 1.363262
H 0 5.182493 1.517610 0.100351
H 0 2.921054 3.574019 -0.163781
H 0 4.080125 3.184291 -1.425786
H 0 4.798804 4.394696 1.317087
H 0 4.762818 5.272007 -0.221101
H 0 5.967109 4.001226 0.046816
Free Energy (PCM/B3LYP/6-31G*) =-1757.656597
Number of imaginary frequencies = 0

2-3*R*-c31
B3LYP/6-31G* geometry
O 0 1.022983 -0.918720 -0.904245
C 0 0.552249 -3.223144 -0.436963
C 0 2.270058 -2.092317 0.939317
C 0 1.742605 -3.464234 0.431134
C 0 2.341274 -1.104392 -0.267584
C 0 0.299297 -2.025885 -1.009380
C 0 -0.979745 -2.036431 -1.809255
N 0 -1.530294 -3.341373 -1.407526
C 0 -0.527446 -4.141175 -0.800169
O 0 -2.286057 -3.971967 -2.413347
C 0 -3.591095 -4.301752 -1.917466
C 0 -1.925844 -0.856408 -1.571857
S 0 -2.543103 -0.815968 0.165148
C 0 -2.704295 0.990679 0.422910
C 0 -3.907060 1.637449 -0.301559
C 0 -3.900425 3.140601 -0.029652
O 0 -5.141663 1.099548 0.112003
O 0 -4.717011 3.679517 0.692241
O 0 -2.890862 3.776815 -0.637091
O 0 -0.623294 -5.346597 -0.614930
O 0 2.838230 -4.069654 -0.286349
O 0 3.595340 -2.267754 1.430038
C 0 1.412477 -1.565888 2.089505
C 0 2.866453 0.286505 0.057611
C 0 3.177388 1.129842 -1.187093
C 0 3.714516 2.524497 -0.842662
C 0 4.040634 3.372736 -2.078750
C 0 4.574337 4.766153 -1.730669
H 0 1.483464 -4.092151 1.291992
H 0 2.972938 -1.576401 -1.030976
H 0 -0.738757 -2.065191 -2.881999
H 0 -4.111250 -4.747447 -2.768895
H 0 -4.124020 -3.406319 -1.580269
H 0 -3.524584 -5.027072 -1.100923
H 0 -2.765026 -0.925359 -2.268685
H 0 -1.384592 0.072137 -1.777436
H 0 -1.771577 1.482436 0.130908
H 0 -2.831316 1.115009 1.503322
H 0 -3.804743 1.485288 -1.382258
H 0 -5.497231 1.722950 0.773399
H 0 -2.931674 4.720988 -0.387165
H 0 2.677980 -5.025254 -0.334866
H 0 3.972307 -2.986040 0.884623
H 0 1.354452 -2.318569 2.881999
H 0 0.396984 -1.323248 1.762263
H 0 1.863134 -0.664169 2.513858
H 0 2.136735 0.809601 0.688336
H 0 3.774345 0.154839 0.656794
H 0 3.913468 0.598822 -1.808109
H 0 2.270675 1.228384 -1.798157
H 0 2.976996 3.055833 -0.222895
H 0 4.617835 2.423767 -0.222886
H 0 3.137725 3.470237 -2.698209
H 0 4.778641 2.841815 -2.696712
H 0 3.844215 5.333504 -1.140108
H 0 4.796102 5.346597 -2.633696
H 0 5.497231 4.700452 -1.141154
Free Energy (PCM/B3LYP/6-31G*) =-1757.656527
Number of imaginary frequencies = 0

2-3*R*-c32
B3LYP/6-31G* geometry
O 0 1.181895 -0.075277 -0.318240
C 0 2.923326 -1.712287 -0.462592
C 0 3.527311 0.554322 0.332502
C 0 4.062331 -0.760968 -0.301195
C 0 2.219898 0.969996 -0.411128
C 0 1.631158 -1.316644 -0.459601
C 0 0.679148 -2.481314 -0.602524
N 0 1.624885 -3.605112 -0.484199
C 0 2.960200 -3.162833 -0.643053
O 0 1.256817 -4.734383 -1.238798
C 0 1.260842 -5.905821 -0.410420
C 0 -0.438296 -2.595418 0.444894
S 0 -1.755233 -1.333526 0.188511
C 0 -3.124539 -2.138628 1.122710
C 0 -4.478341 -1.628334 0.620423
C 0 -4.758557 -2.020016 -0.829438
O 0 -4.556173 -0.223323 0.741020
O 0 -5.085098 -1.206288 -1.672329
O 0 -4.646228 -3.334237 -1.049952
O 0 3.914369 -3.899623 -0.854087
O 0 4.649464 -0.385655 -1.564185
O 0 4.488486 1.584028 0.125320
C 0 3.329999 0.401566 1.840307

C 0 1.576560 2.263311 0.068642
C 0 0.480982 2.786403 -0.870584
C 0 -0.165313 4.084271 -0.370004
C 0 -1.250240 4.624428 -1.310216
C 0 -1.900793 5.915414 -0.802393
H 0 4.840174 -1.182874 0.346559
H 0 2.468212 1.044399 -1.477685
H 0 0.240541 -2.483132 -1.609906
H 0 0.570839 -5.796470 0.433126
H 0 2.269544 -6.118065 -0.043964
H 0 0.925228 -6.714020 -1.064812
H 0 -0.018619 -2.514411 1.451712
H 0 -0.890418 -3.585070 0.334539
H 0 -3.031380 -1.910407 2.187807
H 0 -3.068194 -3.222135 0.989608
H 0 -5.258157 -2.109595 1.233357
H 0 -4.890286 0.100778 -0.117652
H 0 -4.858987 -3.504917 -1.988451
H 0 5.258157 -1.090902 -1.835368
H 0 4.921644 1.358864 -0.721476
H 0 4.261512 0.061739 2.303249
H 0 2.539357 -0.315840 2.078105
H 0 3.063726 1.365094 2.283852
H 0 1.163001 2.108088 1.072899
H 0 2.376826 3.006205 0.160028
H 0 0.912138 2.954694 -1.868245
H 0 -0.293715 2.018291 -0.993418
H 0 -0.601251 3.912809 0.625402
H 0 0.612520 4.850640 -0.234754
H 0 -2.023185 3.855254 -1.449518
H 0 -0.812938 4.800713 -2.303249
H 0 -2.376553 5.760894 0.174090
H 0 -2.670532 6.274961 -1.495093
H 0 -1.157557 6.714020 -0.685668
Free Energy (PCM/B3LYP/6-31G*) =-1757.656525
Number of imaginary frequencies = 0

2-3*R*-c33
B3LYP/6-31G* geometry
O 0 -1.804661 0.790343 1.349525
C 0 -3.063083 -0.482590 -0.239274
C 0 -3.936269 1.717610 0.411954
C 0 -3.988499 0.605430 -0.673812
C 0 -2.477784 2.008735 0.851002
C 0 -2.124397 -0.327969 0.716777
C 0 -1.433382 -1.633792 1.035941
N 0 -1.997090 -2.505263 -0.015380
C 0 -3.135409 -1.890643 -0.586437
O 0 -2.173126 -3.839588 0.398050
C 0 -1.544521 -4.733098 -0.532208
C 0 0.096680 -1.595228 1.059809
S 0 0.783215 -1.210197 -0.608744
C 0 2.378059 -0.428350 -0.155917
C 0 3.408834 -1.421793 0.415158
C 0 4.750841 -0.697184 0.574700
O 0 3.621915 -2.539387 -0.434878
O 0 4.925603 0.190428 1.381592
O 0 5.705377 -1.096449 -0.275540
O 0 -4.002226 -2.470436 -1.239095
O 0 -5.342114 0.136530 -0.739507
O 0 -4.586005 1.215415 1.581893
C 0 -4.649498 2.991103 -0.059999
C 0 -1.566204 2.663585 -0.189519
C 0 -0.199270 3.078546 0.374164
C 0 0.698778 3.743275 -0.676547
C 0 2.064990 4.171040 -0.124918
C 0 2.961164 4.828285 -1.179651
H 0 -3.699570 1.020656 -1.649742
H 0 -2.536868 2.633397 1.746382
H 0 -1.776247 -1.999012 2.014210
H 0 -1.691730 -5.728744 -0.107073
H 0 -0.474584 -4.519072 -0.624355
H 0 -2.024175 -4.673603 -1.513921
H 0 0.476423 -2.558305 1.408274
H 0 0.418028 -0.819589 1.761447
H 0 2.219461 0.388458 0.552626
H 0 2.752238 -0.004168 -1.093262
H 0 3.093829 -1.748080 1.414651
H 0 2.776168 -2.743073 -0.878842
H 0 5.316454 -1.825769 -0.809974
H 0 -5.298539 -0.786402 -1.059516
H 0 -5.324292 0.675679 1.238808
H 0 -5.705377 2.758333 -0.228466
H 0 -4.237089 3.382506 -0.994892
H 0 -4.588916 3.769996 0.707221
H 0 -1.426758 1.980135 -1.037584
H 0 -2.074876 3.551532 -0.580876
H 0 -0.352320 3.770297 1.215152
H 0 0.309724 2.198148 0.785972
H 0 0.848603 3.050374 -1.517723
H 0 0.184726 4.622290 -1.092904
H 0 2.577515 3.293393 0.294291
H 0 1.915087 4.865870 0.713499
H 0 3.157042 4.143595 -2.014210
H 0 3.928107 5.122143 -0.755337
H 0 2.490642 5.728744 -1.593805
Free Energy (PCM/B3LYP/6-31G*) =-1757.656513
Number of imaginary frequencies = 0

2-3*S*-c1
B3LYP/6-31G* geometry
O 0 1.198683 -0.348900 -0.872552
C 0 2.163978 -2.367055 -0.021612
C 0 1.111775 -0.830533 1.581714
C 0 1.573895 -2.295145 1.349062
C 0 0.365568 -0.274816 0.344086
C 0 1.961035 -1.434237 -0.972150
C 0 2.766816 -1.715170 -2.217116
N 0 3.633215 -2.802535 -1.727497
C 0 3.156044 -3.316731 -0.504735
O 0 4.006579 -3.743871 -2.699885
C 0 5.437781 -3.826879 -2.788160
C 0 3.545101 -0.526998 -2.791066
S 0 4.817729 0.127639 -1.624005
C 0 4.464066 1.928536 -1.676148
C 0 3.269902 2.353512 -0.811990
C 0 2.958962 3.844418 -1.037164
O 0 3.528304 2.164107 0.572050
O 0 2.810353 4.339591 -2.135464
O 0 2.853053 4.551690 0.093649
O 0 3.572446 -4.332546 0.048588
O 0 2.554603 -2.587370 2.351232
O 0 2.298845 -0.027861 1.719863
C 0 0.277787 -0.695404 2.859451
C 0 -0.991683 -0.914183 0.038302
C 0 -1.731252 -0.249583 -1.132089
C 0 -3.102616 -0.883719 -1.396558
C 0 -3.860260 -0.228272 -2.558417
C 0 -5.226042 -0.869642 -2.823974
H 0 0.717608 -2.974101 1.463595
H 0 0.246396 0.801800 0.493411
H 0 2.109541 -2.108235 -3.006022
H 0 5.627573 -4.551690 -3.583294
H 0 5.863640 -4.185123 -1.846446
H 0 5.874310 -2.858079 -3.053197
H 0 2.843441 0.272752 -3.043107
H 0 4.036905 -0.839966 -3.715484
H 0 4.319579 2.250664 -2.709922
H 0 5.370961 2.408021 -1.294729
H 0 2.376549 1.798802 -1.122112
H 0 3.036010 1.378761 0.902560
H 0 3.031338 3.902525 0.817811
H 0 3.068721 -3.349556 2.023366
H 0 2.955667 -0.606829 2.155325
H 0 0.897024 -0.978640 3.715484
H 0 -0.602679 -1.344016 2.849407
H 0 -0.047371 0.340755 2.997043
H 0 -0.857569 -1.984327 -0.167176
H 0 -1.611587 -0.843042 0.938866
H 0 -1.858452 0.821071 -0.916338
H 0 -1.117769 -0.313313 -2.039099
H 0 -2.973223 -1.955555 -1.607769
H 0 -3.714946 -0.823771 -0.484653
H 0 -3.245800 -0.284553 -3.468144

H 0	-3.992998	0.841872	-2.345304
H 0	-5.122084	-1.932459	-3.075546
H 0	-5.742707	-0.378878	-3.656820
H 0	-5.874310	-0.800248	-1.941595

Free Energy (PCM/B3LYP/6-31G*) = -1757.662634
Number of imaginary frequencies = 0

2-3*S*-c2
B3LYP/6-31G* geometry

O 0	0.325045	0.096705	0.260099
C 0	2.336799	1.201139	-0.430145
C 0	0.187990	2.081173	-1.285382
C 0	1.730712	2.036126	-1.506092
C 0	-0.323589	0.646021	-0.948381
C 0	1.627125	0.337129	0.329942
C 0	2.495727	-0.382535	1.332443
N 0	3.775532	0.286001	1.067807
C 0	3.742138	1.116840	-0.033623
O 0	4.956541	-0.315316	1.468905
C 0	5.521151	0.370527	2.605627
C 0	2.516184	-1.920661	1.201439
S 0	2.625635	-2.632629	-0.491221
C 0	4.422798	-3.010023	-0.715346
C 0	4.888020	-2.618398	-2.136917
C 0	4.900250	-1.101309	-2.266768
O 0	4.060060	-3.183599	-3.131589
O 0	4.057336	-0.510366	-2.930432
O 0	5.852933	-0.534057	-1.544589
O 0	4.727534	1.662357	-0.554800
O 0	1.961952	1.514046	-2.823518
O 0	-0.428412	2.473243	-2.508473
C 0	-0.189285	3.112869	-0.224779
C 0	-1.823738	0.524322	-0.726755
C 0	-2.324751	-0.925327	-0.657890
C 0	-3.837113	-1.016082	-0.419762
C 0	-4.358469	-2.456659	-0.340598
C 0	-5.868063	-2.537624	-0.091359
H 0	2.122571	3.061513	-1.470686
H 0	-0.004207	-0.005754	-1.771984
H 0	2.166703	-0.156731	2.356156
H 0	5.769062	1.402429	2.342448
H 0	6.429018	-0.187308	2.842840
H 0	4.833451	0.348574	3.456719
H 0	1.570132	-2.289247	1.606448
H 0	3.318107	-2.321818	1.824839
H 0	4.567432	-4.085517	-0.587975
H 0	5.007643	-2.480398	0.039821
H 0	5.911072	-2.993145	-2.266646
H 0	3.503519	-2.450092	-3.456719
H 0	5.623361	0.420766	-1.359966
H 0	2.685181	0.858395	-2.797979
H 0	0.101709	2.043137	-3.207501
H 0	0.240759	4.085517	-0.483209
H 0	0.173555	2.823891	0.766199
H 0	-1.275725	3.226202	-0.173168
H 0	-2.094075	1.051992	0.196598
H 0	-2.314102	1.051101	-1.552925
H 0	-2.069201	-1.442610	-1.594114
H 0	-1.796821	-1.457498	0.144182
H 0	-4.089264	-0.489025	0.512554
H 0	-4.365304	-0.482359	-1.223856
H 0	-3.826128	-2.989839	0.459935
H 0	-4.112367	-2.983026	-1.273747
H 0	-6.139542	-2.049377	0.852942
H 0	-6.209647	-3.577873	-0.039256
H 0	-6.429018	-2.041865	-0.893376

Free Energy (PCM/B3LYP/6-31G*) = -1757.662085
Number of imaginary frequencies = 0

2-3*S*-c3
B3LYP/6-31G* geometry

O 0	1.118580	0.502449	-0.829275
C 0	1.854070	-1.537985	0.181228
C 0	1.006327	0.232730	1.663095
C 0	1.316579	-1.286578	1.549939
C 0	0.319029	0.771175	0.382374
C 0	1.745972	-0.671221	-0.841797
C 0	2.416965	-1.189256	-2.091021
N 0	2.873089	-2.515875	-1.611356
C 0	2.724570	-2.634275	-0.226875
O 0	4.047614	-3.023746	-2.171220
C 0	3.758748	-4.177114	-2.979192
C 0	3.527082	-0.311005	-2.684831
S 0	4.910540	0.082633	-1.531035
C 0	4.985869	1.909969	-1.701580
C 0	3.876899	2.665653	-0.958595
C 0	3.923466	4.160819	-1.328637
O 0	4.021161	2.554796	0.449342
O 0	3.933463	4.568782	-2.471842
O 0	3.940746	4.977571	-0.269560
O 0	3.236244	-3.495262	0.490212
O 0	2.293538	-1.596536	2.552603
O 0	2.272709	0.910220	1.762248
C 0	0.188619	0.548151	2.919944
C 0	-1.108147	0.274293	0.133452
C 0	-1.770215	0.911147	-1.097226
C 0	-3.213218	0.432858	-1.302448
C 0	-3.889401	1.056315	-2.530301
C 0	-5.329035	0.573251	-2.734183
H 0	0.402175	-1.865379	1.740957
H 0	0.330327	1.862764	0.443858
H 0	1.664152	-1.323922	-2.879781
H 0	4.726613	-4.488441	-3.378244
H 0	3.079912	-3.923853	-3.800260
H 0	3.328928	-4.977571	-2.369531
H 0	3.071458	0.625549	-3.016424
H 0	3.935536	-0.814254	-3.563013
H 0	4.983626	2.180227	-2.760149
H 0	5.955251	2.194787	-1.280690
H 0	2.893982	2.299375	-1.280002
H 0	3.314491	1.974969	0.815204
H 0	3.937682	4.375409	0.515125
H 0	2.787157	-2.374914	2.222581
H 0	2.852599	0.281217	2.239686
H 0	0.773119	0.264851	3.800260
H 0	-0.754898	-0.004000	2.947553
H 0	-0.025757	1.619989	2.979024
H 0	-1.105544	-0.818905	0.030134
H 0	-1.708789	0.504322	1.020343
H 0	-1.759330	2.005086	-0.986435
H 0	-1.179021	0.683795	-1.992568
H 0	-3.222932	-0.662762	-1.401456
H 0	-3.806097	0.663826	-0.405114
H 0	-3.295011	0.826055	-3.425864
H 0	-3.880633	2.151012	-2.431134
H 0	-5.365859	-0.514654	-2.871309
H 0	-5.784732	1.036166	-3.617069
H 0	-5.955251	0.819010	-1.867542

Free Energy (PCM/B3LYP/6-31G*) = -1757.661660
Number of imaginary frequencies = 0

2-3*S*-c4
B3LYP/6-31G* geometry

O 0	0.247798	-0.310493	0.393299
C 0	-1.734247	-1.296145	-0.524740
C 0	0.454365	-2.125249	-1.337054
C 0	-1.075996	-2.043021	-1.634173
C 0	0.952931	-0.736232	-0.832758
C 0	-1.059901	-0.528883	0.361044
C 0	-1.977356	0.105309	1.378130
N 0	-3.252712	-0.483142	0.952164
C 0	-3.162501	-1.205640	-0.218344
O 0	-4.444697	0.101550	1.342677
C 0	-5.102987	-0.695219	2.349796
C 0	-1.944913	1.647196	1.430747
S 0	-1.907584	2.559205	-0.166389
C 0	-3.675127	3.008187	-0.478165
C 0	-4.046801	2.780975	-1.961735
C 0	-4.081385	1.287279	-2.254296
O 0	-3.139912	3.425396	-2.831503
O 0	-3.202444	0.745877	-2.913331
O 0	-5.099321	0.675527	-1.670940
O 0	-4.122412	-1.663007	-0.858650
O 0	-1.241318	-1.415856	-2.915570

O	0	1.131979	-2.400454	-2.559058
C	0	0.768733	-3.260431	-0.365046
C	0	2.440716	-0.644432	-0.528776
C	0	2.933757	0.789033	-0.284160
C	0	4.435973	0.844606	0.027180
C	0	4.970182	2.249538	0.351800
C	0	4.921835	3.236262	-0.821090
H	0	-1.475080	-3.063496	-1.706416
H	0	0.673011	-0.002813	-1.600507
H	0	-1.728376	-0.249195	2.388124
H	0	-6.009466	-0.138358	2.594369
H	0	-4.473648	-0.798777	3.239036
H	0	-5.360255	-1.678971	1.947897
H	0	-1.020914	1.930202	1.941629
H	0	-2.778072	2.002353	2.040969
H	0	-3.804540	4.068688	-0.249066
H	0	-4.325022	2.422090	0.175491
H	0	-5.050418	3.195529	-2.119343
H	0	-2.577794	2.715830	-3.197481
H	0	-4.911243	-0.301659	-1.580090
H	0	-1.912278	-0.708928	-2.855241
H	0	0.632242	-1.907158	-3.239036
H	0	0.352732	-4.199209	-0.743531
H	0	0.350736	-3.069542	0.628021
H	0	1.850268	-3.386599	-0.263534
H	0	2.667336	-1.268043	0.345218
H	0	2.971021	-1.082299	-1.381795
H	0	2.707920	1.399778	-1.168590
H	0	2.373970	1.231434	0.550635
H	0	4.642631	0.183488	0.880880
H	0	4.998678	0.429952	-0.822667
H	0	6.009466	2.156540	0.694688
H	0	4.405556	2.662091	1.200270
H	0	5.479623	2.849914	-1.683647
H	0	5.365371	4.199209	-0.542381
H	0	3.894921	3.431018	-1.150102

Free Energy (PCM/B3LYP/6-31G*) = -1757.660988

Number of imaginary frequencies = 0

2-3*S*-c5

B3LYP/6-31G* geometry

O	0	1.181067	-0.274276	-0.017552
C	0	2.233989	-2.397972	0.273156
C	0	0.939240	-1.498833	2.161619
C	0	1.544569	-2.786485	1.537775
C	0	0.263950	-0.608826	1.088147
C	0	2.041424	-1.225059	-0.361272
C	0	2.964047	-1.073447	-1.552398
N	0	3.557399	-2.437700	-1.578244
C	0	3.314474	-3.120016	-0.383838
O	0	4.818601	-2.586609	-2.155114
C	0	4.718011	-3.329431	-3.382044
C	0	4.043727	0.014148	-1.377711
S	0	3.402240	1.686694	-1.829638
C	0	4.364914	2.755664	-0.671581
C	0	3.514819	3.382842	0.439018
C	0	2.709474	2.355245	1.235257
O	0	2.627745	4.337061	-0.106556
O	0	1.518557	2.517884	1.451416
O	0	3.429893	1.325022	1.656838
O	0	3.898679	-4.129897	0.013138
O	0	2.477388	-3.319412	2.488402
O	0	2.041587	-0.733601	2.698894
C	0	-0.015074	-1.825315	3.314419
C	0	-1.028742	-1.165914	0.484803
C	0	-1.707545	-0.199151	-0.496796
C	0	-3.018824	-0.758913	-1.062104
C	0	-3.719161	0.200316	-2.033303
C	0	-5.025213	-0.364944	-2.600703
H	0	0.745779	-3.520037	1.360750
H	0	0.082042	0.366984	1.544502
H	0	2.396504	-0.901168	-2.474500
H	0	4.335717	-4.337061	-3.192798
H	0	5.740663	-3.381709	-3.761997
H	0	4.077006	-2.813786	-4.104631
H	0	4.886643	-0.234158	-2.023903
H	0	4.383693	0.035746	-0.340626
H	0	4.798379	3.566201	-1.262671
H	0	5.185916	2.171516	-0.250984
H	0	4.208856	3.870207	1.145441
H	0	1.755831	4.130200	0.283492
H	0	2.836571	0.634035	2.074636
H	0	3.124861	-3.840864	1.971787
H	0	2.692089	-1.407622	2.988026
H	0	0.546897	-2.331769	4.104631
H	0	-0.827801	-2.485945	3.000443
H	0	-0.443677	-0.907868	3.730168
H	0	-0.822127	-2.121229	-0.016010
H	0	-1.722274	-1.384656	1.304203
H	0	-1.907048	0.752499	0.016571
H	0	-1.021307	0.031048	-1.320744
H	0	-2.817019	-1.710782	-1.575434
H	0	-3.701869	-0.997706	-0.233389
H	0	-3.035080	0.442405	-2.858976
H	0	-3.923864	1.149633	-1.518354
H	0	-4.846497	-1.295107	-3.154344
H	0	-5.503214	0.344458	-3.286054
H	0	-5.740663	-0.588887	-1.799628

Free Energy (PCM/B3LYP/6-31G*) = -1757.660970

Number of imaginary frequencies = 0

2-3*S*-c6

B3LYP/6-31G* geometry

O	0	0.265163	-0.385859	-0.988773
C	0	2.297544	-1.252001	-0.066003
C	0	0.150399	-2.252814	0.681971
C	0	1.671238	-2.090073	0.991031
C	0	-0.424311	-0.878020	0.217134
C	0	1.582886	-0.519667	-0.950663
C	0	2.469798	0.179675	-1.952003
N	0	3.796972	-0.077047	-1.359690
C	0	3.715924	-1.124208	-0.403060
O	0	4.802181	-0.250496	-2.335720
C	0	5.937290	0.571832	-2.036117
C	0	2.162826	1.660566	-2.223992
S	0	1.693608	2.728741	-0.805332
C	0	3.282380	3.009584	0.095393
C	0	2.993438	3.205303	1.591599
C	0	2.377342	1.946771	2.203350
O	0	2.119426	4.296838	1.801353
O	0	1.260851	1.955029	2.697736
O	0	3.170644	0.886911	2.121385
O	0	4.677302	-1.749045	0.019148
O	0	1.814994	-1.457078	2.301404
O	0	-0.527368	-2.629898	1.873722
C	0	-0.072954	-3.368011	-0.338913
C	0	-1.913983	-0.867266	-0.095490
C	0	-2.487509	0.541711	-0.300328
C	0	-3.991116	0.533163	-0.603925
C	0	-4.577173	1.935908	-0.808280
C	0	-6.078993	1.924144	-1.111923
H	0	2.143546	-3.075539	1.043455
H	0	-0.198315	-0.144423	1.003581
H	0	2.421432	-0.345924	-2.917488
H	0	5.667389	1.633314	-2.024152
H	0	6.644623	0.378651	-2.846390
H	0	6.383039	0.286371	-1.078777
H	0	1.289170	1.713135	-2.880571
H	0	3.001470	2.100361	-2.771240
H	0	3.770101	3.907287	-0.294328
H	0	3.936691	2.149782	-0.050938
H	0	3.953885	3.393919	2.094336
H	0	1.337844	3.917465	2.249918
H	0	2.661344	0.059704	2.349895
H	0	2.203246	-2.099811	2.917488
H	0	-0.137150	-2.085349	2.582959
H	0	0.378055	-4.296838	0.023259
H	0	0.366606	-3.121560	-1.309498
H	0	-1.142791	-3.543967	-0.479389
H	0	-2.098472	-1.476617	-0.988971
H	0	-2.421903	-1.360716	0.740577
H	0	-2.300472	1.142993	0.601035
H	0	-1.953002	1.038632	-1.120275
H	0	-4.177615	-0.071784	-1.503713

H 0 -4.525710 0.032154 0.216822
H 0 -4.041615 2.435193 -1.628234
H 0 -4.389933 2.540090 0.090738
H 0 -6.293959 1.355878 -2.025500
H 0 -6.466422 2.939870 -1.252352
H 0 -6.644623 1.461311 -0.293650
Free Energy (PCM/B3LYP/6-31G*) = -1757.660723
Number of imaginary frequencies = 0

3-S*R*-c1
B3LYP/6-31G* geometry
O 0 1.503449 -0.786739 0.096849
C 0 2.220347 -3.017280 -0.364334
C 0 3.943537 -1.344137 0.190406
C 0 3.677667 -2.708780 -0.505612
C 0 2.854070 -0.342229 -0.297079
C 0 1.294415 -2.090265 -0.053816
C 0 -0.079991 -2.700482 0.098025
N 0 0.181602 -4.077951 -0.361120
C 0 1.575326 -4.319891 -0.397069
O 0 -0.587187 -5.049723 0.315118
C 0 -1.157117 -5.973895 -0.626237
C 0 -1.147203 -1.966482 -0.716189
C 0 -3.834024 -1.353551 -1.169764
C 0 -3.885253 0.020674 -0.458211
C 0 -4.979535 0.095036 0.628427
O 0 -4.143525 1.012063 -1.423714
O 0 -5.863722 0.930976 0.528032
O 0 -4.911053 -0.742847 1.644563
O 0 2.098240 -5.430394 -0.453382
O 0 4.501144 -3.720754 0.067768
O 0 5.170919 -0.798335 -0.301685
C 0 3.991155 -1.475653 1.716782
C 0 3.007957 1.085172 0.209276
C 0 2.052878 2.081404 -0.463560
C 0 2.243287 3.516432 0.043570
C 0 1.296388 4.526272 -0.617338
C 0 1.494289 5.958508 -0.110288
S 0 -2.819132 -2.649316 -0.338418
O 0 -3.010102 -2.447404 1.184356
H 0 3.943795 -2.580827 -1.566874
H 0 2.862392 -0.351088 -1.394497
H 0 -0.374795 -2.719766 1.155649
H 0 -0.371386 -6.529048 -1.145506
H 0 -1.797164 -5.455481 -1.347279
H 0 -1.755826 -6.655213 -0.017459
H 0 -1.133855 -0.902719 -0.464497
H 0 -0.983115 -2.097871 -1.789642
H 0 -4.830852 -1.786594 -1.290617
H 0 -3.402087 -1.219508 -2.165583
H 0 -2.923777 0.224644 0.034533
H 0 -4.977326 1.434281 -1.126881
H 0 -4.133856 -1.387343 1.547030
H 0 4.078384 -4.575078 -0.142848
H 0 5.863722 -1.452334 -0.108157
H 0 4.741050 -2.219637 1.996810
H 0 3.030994 -1.794995 2.133443
H 0 4.264236 -0.517361 2.165583
H 0 2.855162 1.099549 1.295265
H 0 4.046379 1.382725 0.027375
H 0 1.014749 1.768625 -0.292126
H 0 2.209934 2.055517 -1.551690
H 0 2.094276 3.540082 1.133302
H 0 3.284136 3.828157 -0.128982
H 0 0.256701 4.215568 -0.441696
H 0 1.442937 4.500256 -1.706500
H 0 1.320729 6.023494 0.971095
H 0 0.804940 6.655213 -0.601070
H 0 2.516066 6.308490 -0.303041
Free Energy (PCM/B3LYP/6-31G*) = -1832.836375
Number of imaginary frequencies = 0

3-S*R*-c2
B3LYP/6-31G* geometry
O 0 2.005412 -0.539628 0.546624
C 0 2.626364 -2.644921 -0.396050
C 0 4.147240 -0.736368 -0.736504
C 0 3.715439 -2.126589 -1.281768
C 0 2.854545 0.094504 -0.479798
C 0 1.913838 -1.861480 0.435422
C 0 0.944642 -2.664375 1.271256
N 0 1.069229 -3.986307 0.628464
C 0 2.229811 -4.028854 -0.178264
O 0 0.891015 -5.064681 1.511764
C 0 -0.104673 -5.968574 0.998755
C 0 -0.484498 -2.121342 1.323174
C 0 -2.545407 -0.851573 0.013474
C 0 -3.731164 -0.991425 -0.952750
C 0 -4.665168 0.215544 -0.770348
O 0 -4.489114 -2.163037 -0.705515
O 0 -4.318480 1.356451 -0.987207
O 0 -5.890487 -0.099269 -0.338125
O 0 2.761770 -5.051891 -0.601143
O 0 4.837391 -3.004478 -1.327927
O 0 4.828724 -0.018265 -1.769152
C 0 5.034625 -0.856533 0.507431
C 0 3.080344 1.531308 -0.029372
C 0 1.798493 2.376245 -0.013218
C 0 2.047458 3.817290 0.449870
C 0 0.778400 4.678792 0.467732
C 0 1.032461 6.113742 0.941243
S 0 -1.323057 -2.193835 -0.322597
O 0 -2.164234 -3.485180 -0.357829
H 0 3.337978 -1.964083 -2.303743
H 0 2.263948 0.082362 -1.404932
H 0 1.314126 -2.734093 2.303743
H 0 -0.248412 -6.703362 1.794516
H 0 0.258811 -6.467002 0.094608
H 0 -1.037162 -5.438446 0.787385
H 0 -1.108950 -2.708605 2.003009
H 0 -0.470754 -1.078909 1.654208
H 0 -2.033228 0.103941 -0.127767
H 0 -2.893739 -0.944244 1.046943
H 0 -3.370711 -0.958938 -1.991524
H 0 -3.844135 -2.896093 -0.550170
H 0 -5.905566 -1.078463 -0.229362
H 0 4.478399 -3.911762 -1.353895
H 0 5.582282 -0.569020 -2.041210
H 0 5.905566 -1.475593 0.277986
H 0 4.506687 -1.320426 1.346177
H 0 5.379911 0.133015 0.817314
H 0 3.534360 1.527694 0.968952
H 0 3.811908 1.975297 -0.713077
H 0 1.358557 2.386767 -1.020980
H 0 1.056572 1.905673 0.644987
H 0 2.489733 3.803498 1.457081
H 0 2.795929 4.286515 -0.205775
H 0 0.029477 4.205434 1.118471
H 0 0.339414 4.697476 -0.539773
H 0 1.441821 6.127414 1.959108
H 0 0.108480 6.703362 0.945266
H 0 1.751630 6.624172 0.288603
Free Energy (PCM/B3LYP/6-31G*) = -1832.835382
Number of imaginary frequencies = 0

3-S*R*-c3
B3LYP/6-31G* geometry
O 0 1.885236 -1.335576 0.453907
C 0 1.698004 -3.580354 -0.341354
C 0 3.895282 -2.477831 -0.510282
C 0 3.002563 -3.618692 -1.073316
C 0 3.032306 -1.181456 -0.462080
C 0 1.274904 -2.513480 0.362655
C 0 -0.031946 -2.790848 1.068875
N 0 -0.390736 -4.088102 0.467345
C 0 0.740124 -4.661672 -0.161217
O 0 -1.110456 -4.930746 1.332502
C 0 -2.301548 -5.408604 0.681807
C 0 -1.110503 -1.715028 0.921794
C 0 -2.104530 0.242739 -0.825659
C 0 -3.252737 0.656752 0.124459
C 0 -4.661964 0.467277 -0.481357
O 0 -3.082917 2.018431 0.441126
O 0 -5.419645 1.424751 -0.523437

O 0	-5.015657	-0.728856	-0.905520
O 0	0.846845	-5.840659	-0.486397
O 0	3.664583	-4.872696	-0.930262
O 0	4.927452	-2.179938	-1.455003
C 0	4.503219	-2.839999	0.849380
C 0	3.769454	0.078645	-0.031280
C 0	2.956021	1.364303	-0.237657
C 0	3.729893	2.624676	0.169195
C 0	2.938093	3.921013	-0.044064
C 0	3.721126	5.176899	0.352782
S 0	-1.650152	-1.539478	-0.841143
O 0	-2.981186	-2.318769	-0.971469
H 0	2.851197	-3.405738	-2.143421
H 0	2.598599	-1.035787	-1.459754
H 0	0.149023	-2.932130	2.143421
H 0	-2.841953	-5.955407	1.458087
H 0	-2.045251	-6.084973	-0.139432
H 0	-2.901426	-4.573861	0.309108
H 0	-2.000738	-1.985193	1.495657
H 0	-0.723423	-0.756227	1.277133
H 0	-2.325744	0.497639	-1.866358
H 0	-1.196679	0.779446	-0.535606
H 0	-3.214140	0.053178	1.043821
H 0	-3.942823	2.432479	0.214729
H 0	-4.252180	-1.400556	-0.861278
H 0	2.975001	-5.559669	-1.000340
H 0	5.419645	-3.005901	-1.598846
H 0	5.075446	-3.766698	0.759263
H 0	3.737371	-2.994241	1.615621
H 0	5.174971	-2.044682	1.182072
H 0	4.057553	-0.015023	1.022729
H 0	4.695699	0.125641	-0.614106
H 0	2.022841	1.306127	0.337874
H 0	2.664958	1.442025	-1.295283
H 0	4.021605	2.547689	1.227079
H 0	4.667957	2.674384	-0.403374
H 0	2.003761	3.873822	0.533157
H 0	2.641138	3.993433	-1.099987
H 0	4.005216	5.148124	1.412107
H 0	3.128991	6.084973	0.190790
H 0	4.642862	5.270003	-0.234846

Free Energy (PCM/B3LYP/6-31G*) = -1832.834971
Number of imaginary frequencies = 0

3-S*R*-c4
B3LYP/6-31G* geometry

O 0	1.862691	-0.981295	0.053452
C 0	2.320607	-3.252893	-0.523665
C 0	4.236393	-1.766494	-0.083736
C 0	3.783168	-3.076475	-0.787478
C 0	3.209790	-0.653073	-0.450638
C 0	1.518229	-2.254460	-0.107383
C 0	0.113056	-2.740439	0.160546
N 0	0.198294	-4.118273	-0.359906
C 0	1.553817	-4.488290	-0.527955
O 0	-0.599326	-5.035941	0.357706
C 0	-1.337119	-5.867075	-0.552930
C 0	-0.957865	-1.882467	-0.514557
C 0	-3.573878	-1.019447	-0.730642
C 0	-4.935678	-0.892735	-0.029405
C 0	-5.768461	0.176567	-0.754866
O 0	-4.809115	-0.493717	1.324303
O 0	-6.104922	0.074889	-1.914704
O 0	-6.079702	1.235379	-0.000603
O 0	1.960601	-5.640455	-0.658716
O 0	4.553019	-4.178394	-0.313892
O 0	5.464736	-1.320717	-0.665944
C 0	4.397871	-1.952151	1.429024
C 0	3.542603	0.739465	0.066866
C 0	2.635735	1.837565	-0.506941
C 0	3.005918	3.236453	0.001777
C 0	2.113867	4.346189	-0.569269
C 0	2.487700	5.742040	-0.060012
S 0	-2.638368	-2.419354	0.021707
O 0	-2.700937	-2.175035	1.541866
H 0	3.968060	-2.939094	-1.864733
H 0	3.121600	-0.630888	-1.544410
H 0	-0.076206	-2.774165	1.242112
H 0	-1.942824	-6.511456	0.088338
H 0	-0.657588	-6.473723	-1.157399
H 0	-1.984997	-5.264091	-1.197077
H 0	-0.836317	-0.836802	-0.216352
H 0	-0.912396	-1.970429	-1.604230
H 0	-3.700764	-1.234810	-1.794805
H 0	-2.998195	-0.098705	-0.596554
H 0	-5.490179	-1.838649	-0.118261
H 0	-4.060313	-1.008684	1.712162
H 0	-5.678000	1.076172	0.884904
H 0	4.035379	-4.982755	-0.508844
H 0	6.104922	-2.043624	-0.553074
H 0	5.109919	-2.757614	1.625382
H 0	3.453545	-2.216013	1.914704
H 0	4.777284	-1.032010	1.880436
H 0	3.482916	0.741596	1.162023
H 0	4.586092	0.941692	-0.197944
H 0	2.697926	1.820995	-1.604799
H 0	1.590166	1.621369	-0.252064
H 0	2.945982	3.252292	1.100123
H 0	4.055550	3.449136	-0.249852
H 0	1.065412	4.132676	-0.317057
H 0	2.173350	4.328582	-1.666831
H 0	2.404509	5.800231	1.032397
H 0	1.833274	6.511456	-0.485729
H 0	3.520698	5.997357	-0.327296

Free Energy (PCM/B3LYP/6-31G*) =-1832.834744
Number of imaginary frequencies = 0

3-S*R*-c5
B3LYP/6-31G* geometry

O 0	1.190808	-1.139298	0.165012
C 0	1.450322	-3.466119	-0.320057
C 0	3.472195	-2.176181	0.236387
C 0	2.936680	-3.438533	-0.491550
C 0	2.603410	-0.965097	-0.217987
C 0	0.726274	-2.374702	-0.011652
C 0	-0.736891	-2.701718	0.168941
N 0	-0.674806	-4.173607	0.124330
C 0	0.566436	-4.627246	-0.351740
O 0	-1.817535	-4.844250	-0.320560
C 0	-2.424474	-5.570784	0.766036
C 0	-1.592013	-2.057499	-0.928794
C 0	-4.015925	-0.688484	-1.243441
C 0	-3.677959	0.540842	-0.365827
C 0	-4.724731	0.791100	0.740466
O 0	-3.615962	1.679119	-1.192140
O 0	-5.320575	1.856353	0.769076
O 0	-4.928244	-0.158046	1.633435
O 0	0.835670	-5.785725	-0.658907
O 0	3.567915	-4.603101	0.032914
O 0	4.783351	-1.875584	-0.250688
C 0	3.495665	-2.355225	1.758650
C 0	3.038454	0.389678	0.324131
C 0	2.307627	1.571393	-0.329139
C 0	2.777601	2.928614	0.209087
C 0	2.061993	4.120330	-0.439608
C 0	2.536956	5.473907	0.098755
S 0	-3.407867	-2.312622	-0.615206
O 0	-3.581468	-2.260227	0.924833
H 0	3.200723	-3.328364	-1.555268
H 0	2.616276	-0.945007	-1.315200
H 0	-1.103890	-2.364912	1.145169
H 0	-1.722368	-6.304118	1.171929
H 0	-3.280736	-6.078259	0.316470
H 0	-2.763405	-4.884289	1.546966
H 0	-1.377408	-0.986366	-0.952658
H 0	-1.390463	-2.489177	-1.912860
H 0	-5.094208	-0.806167	-1.383920
H 0	-3.561146	-0.557107	-2.229875
H 0	-2.709280	0.391525	0.133989
H 0	-4.287174	2.290492	-0.821764
H 0	-4.368076	-0.980702	1.437565
H 0	3.052694	-5.364351	-0.292493
H 0	5.320575	-2.673053	-0.106749
H 0	4.087241	-3.237522	2.016209

H 0 2.491907 -2.491960 2.172485
H 0 3.950706 -1.480377 2.229875
H 0 2.883944 0.411217 1.409754
H 0 4.116719 0.476618 0.151400
H 0 1.226611 1.469166 -0.168290
H 0 2.464571 1.536959 -1.417106
H 0 2.623153 2.962098 1.297811
H 0 3.862014 3.026635 0.051406
H 0 0.978811 4.022115 -0.280012
H 0 2.214643 4.084735 -1.527675
H 0 2.364671 5.552428 1.179446
H 0 2.008866 6.304118 -0.384317
H 0 3.610909 5.614274 -0.076198
Free Energy (PCM/B3LYP/6-31G*) =-1832.834363
Number of imaginary frequencies = 0

3-S*R*-c6
B3LYP/6-31G* geometry
O 0 -1.435624 -0.731309 1.184046
C 0 -2.203971 -2.770291 0.174947
C 0 -3.632105 -1.691516 1.872170
C 0 -3.599238 -2.734237 0.717488
C 0 -2.840765 -0.399902 1.526717
C 0 -1.287728 -1.810483 0.420687
C 0 0.035873 -2.108544 -0.243370
N 0 -0.207471 -3.475036 -0.731049
C 0 -1.596436 -3.762479 -0.711142
O 0 0.456628 -3.762715 -1.942148
C 0 1.116492 -5.035664 -1.854310
C 0 1.225035 -1.982654 0.711111
C 0 3.956185 -1.547941 1.100932
C 0 3.889660 -0.039722 1.443717
C 0 4.777476 0.829375 0.527244
O 0 4.315501 0.130991 2.774588
O 0 5.661051 1.509717 1.024610
O 0 4.535633 0.822025 -0.768944
O 0 -2.124994 -4.682616 -1.319940
O 0 -4.561151 -2.389805 -0.285872
O 0 -3.021085 -2.293230 3.023628
C 0 -5.061277 -1.367610 2.299662
C 0 -3.407137 0.499353 0.426493
C 0 -2.608002 1.795366 0.227950
C 0 -3.222611 2.710409 -0.838827
C 0 -2.433699 4.008710 -1.051686
C 0 -3.049265 4.920195 -2.118420
S 0 2.810550 -2.124674 -0.222946
O 0 2.763946 -0.998786 -1.284391
H 0 -3.859361 -3.704493 1.162593
H 0 -2.729974 0.165381 2.455530
H 0 0.184709 -1.433450 -1.097392
H 0 1.851427 -5.041830 -1.042837
H 0 1.621116 -5.154067 -2.815964
H 0 0.388177 -5.838679 -1.713054
H 0 1.193062 -1.009268 1.207098
H 0 1.220674 -2.782147 1.457438
H 0 3.700150 -2.124034 1.994829
H 0 4.957884 -1.849110 0.781040
H 0 2.858404 0.325526 1.333577
H 0 5.076724 0.746159 2.712880
H 0 3.792587 0.173910 -1.009353
H 0 -4.454765 -3.033818 -1.006160
H 0 -2.080828 -2.446015 2.833345
H 0 -5.524999 -2.276657 2.695496
H 0 -5.661051 -1.010965 1.461836
H 0 -5.057727 -0.611692 3.091693
H 0 -3.470356 -0.062416 -0.511991
H 0 -4.437259 0.752437 0.703925
H 0 -1.575312 1.552196 -0.051850
H 0 -2.550672 2.337098 1.183489
H 0 -3.287483 2.164780 -1.791896
H 0 -4.257136 2.956837 -0.557188
H 0 -1.400202 3.761455 -1.332806
H 0 -2.368757 4.552741 -0.098613
H 0 -3.094153 4.415849 -3.091693
H 0 -2.464645 5.838679 -2.245202
H 0 -4.072359 5.210259 -1.848492
Free Energy (PCM/B3LYP/6-31G*) =-1832.834272
Number of imaginary frequencies = 0

3-S*R*-c7
B3LYP/6-31G* geometry
O 0 -1.436954 -0.717340 0.823404
C 0 -2.182012 -2.777407 -0.157265
C 0 -3.601955 -1.723644 1.557116
C 0 -3.567153 -2.763225 0.405965
C 0 -2.832511 -0.418875 1.200840
C 0 -1.277301 -1.806415 0.084823
C 0 0.051187 -2.097193 -0.572782
N 0 -0.172353 -3.473536 -1.042113
C 0 -1.561160 -3.775545 -1.021489
O 0 0.489493 -3.760294 -2.256580
C 0 1.171432 -5.020166 -2.161121
C 0 1.233835 -1.944691 0.385949
C 0 3.960967 -1.492976 0.790346
C 0 3.885643 0.017017 1.123529
C 0 4.774075 0.884614 0.206178
O 0 4.304195 0.198275 2.455450
O 0 5.651956 1.572166 0.703936
O 0 4.539066 0.868025 -1.091073
O 0 -2.072927 -4.715202 -1.616201
O 0 -4.558541 -2.429574 -0.571127
O 0 -2.883376 -2.385630 2.609360
C 0 -5.033046 -1.421213 2.007226
C 0 -3.442842 0.480422 0.121556
C 0 -2.666573 1.787831 -0.090844
C 0 -3.313941 2.695756 -1.144357
C 0 -2.546275 4.004338 -1.371115
C 0 -3.193027 4.907604 -2.426368
S 0 2.825392 -2.082967 -0.536365
O 0 2.780089 -0.962863 -1.604606
H 0 -3.801727 -3.738803 0.853893
H 0 -2.711792 0.154410 2.125882
H 0 0.196110 -1.432947 -1.435724
H 0 1.914918 -5.004819 -1.357435
H 0 1.668425 -5.141868 -3.126482
H 0 0.458825 -5.834074 -2.002757
H 0 1.189198 -0.964893 0.868148
H 0 1.232606 -2.733555 1.143465
H 0 3.702466 -2.064562 1.686385
H 0 4.965913 -1.791229 0.477977
H 0 2.853215 0.376670 1.006372
H 0 5.063391 0.815899 2.393204
H 0 3.800456 0.214120 -1.331349
H 0 -4.446936 -3.057531 -1.304198
H 0 -2.926437 -1.821071 3.398907
H 0 -5.483569 -2.341851 2.391096
H 0 -5.651956 -1.049642 1.188955
H 0 -5.033100 -0.676577 2.812435
H 0 -3.520696 -0.078659 -0.817448
H 0 -4.469024 0.718066 0.425542
H 0 -1.636436 1.559077 -0.391060
H 0 -2.597946 2.329425 0.864161
H 0 -3.389894 2.150067 -2.096583
H 0 -4.346058 2.928306 -0.842741
H 0 -1.514920 3.770663 -1.671108
H 0 -2.471087 4.549233 -0.419293
H 0 -3.248232 4.402845 -3.398907
H 0 -2.623446 5.834074 -2.563181
H 0 -4.215096 5.183520 -2.138300
Free Energy (PCM/B3LYP/6-31G*) =-1832.834108
Number of imaginary frequencies = 0

3-S*R*-c8
B3LYP/6-31G* geometry
O 0 -1.437410 -0.714126 1.017257
C 0 -2.177245 -2.779710 0.045745
C 0 -3.599493 -1.717756 1.757101
C 0 -3.563009 -2.763760 0.605738
C 0 -2.832746 -0.418097 1.403107
C 0 -1.275485 -1.804427 0.282319
C 0 0.053666 -2.094680 -0.374352
N 0 -0.166278 -3.473559 -0.838188
C 0 -1.554216 -3.779548 -0.814535
O 0 0.494214 -3.762407 -2.053010

C 0 1.180923 -5.019407 -1.954477
C 0 1.235238 -1.935488 0.584707
C 0 3.962492 -1.487089 0.992623
C 0 3.887695 0.022739 1.326376
C 0 4.775035 0.891192 0.408731
O 0 4.308001 0.202808 2.657915
O 0 5.652145 1.579813 0.906403
O 0 4.539849 0.874643 -0.888472
O 0 -2.064034 -4.723488 -1.404312
O 0 -4.553757 -2.441219 -0.377034
O 0 -2.845003 -2.255935 2.853629
C 0 -5.031258 -1.413154 2.204455
C 0 -3.449407 0.478839 0.327218
C 0 -2.674094 1.786546 0.113339
C 0 -3.325262 2.695361 -0.937012
C 0 -2.558474 4.004255 -1.164871
C 0 -3.208622 4.908532 -2.217166
S 0 2.827953 -2.077479 -0.334645
O 0 2.786031 -0.959787 -1.405461
H 0 -3.799160 -3.744240 1.047795
H 0 -2.709739 0.141096 2.334104
H 0 0.197151 -1.433651 -1.239938
H 0 1.926719 -4.998198 -1.153067
H 0 1.675581 -5.143152 -2.920779
H 0 0.471734 -5.835267 -1.790893
H 0 1.188870 -0.953102 1.061415
H 0 1.233770 -2.719986 1.346740
H 0 3.703335 -2.058825 1.888387
H 0 4.967572 -1.785521 0.680818
H 0 2.855136 0.382559 1.210752
H 0 5.065402 0.822613 2.595460
H 0 3.802488 0.219463 -1.129269
H 0 -4.434544 -3.068910 -1.109406
H 0 -3.325080 -3.030719 3.190096
H 0 -5.491233 -2.329809 2.592656
H 0 -5.652145 -1.054381 1.381984
H 0 -5.025899 -0.668022 3.006611
H 0 -3.530881 -0.079030 -0.612257
H 0 -4.474092 0.716582 0.635724
H 0 -1.644982 1.558415 -0.190713
H 0 -2.602293 2.326908 1.068725
H 0 -3.404320 2.150701 -1.889588
H 0 -4.356390 2.927474 -0.631691
H 0 -1.528037 3.771008 -1.468339
H 0 -2.480350 4.548170 -0.212735
H 0 -3.266688 4.404838 -3.190096
H 0 -2.639645 5.835267 -2.354700
H 0 -4.229862 5.183938 -1.925686
Free Energy (PCM/B3LYP/6-31G*) =-1832.834093
Number of imaginary frequencies = 0

3-S*S*-c1
B3LYP/6-31G* geometry
O 0 -1.408477 -1.347469 0.648285
C 0 -3.503740 -0.889860 -0.398795
C 0 -2.500629 -3.119493 -0.732123
C 0 -3.516876 -2.087047 -1.295740
C 0 -1.183886 -2.353132 -0.404362
C 0 -2.511416 -0.630048 0.474035
C 0 -2.780106 0.625606 1.270732
N 0 -3.973591 1.141254 0.574391
C 0 -4.530452 0.126426 -0.247403
O 0 -4.885165 1.790146 1.430405
C 0 -5.224443 3.083552 0.908849
C 0 -1.612354 1.615110 1.344748
C 0 0.315102 3.259365 0.236277
C 0 1.314230 3.435002 -0.931726
C 0 2.336346 2.277918 -1.008789
O 0 2.016798 4.641144 -0.748263
O 0 3.529012 2.533951 -0.952587
O 0 1.884627 1.044921 -1.136491
O 0 -5.662383 0.150958 -0.722402
O 0 -4.811386 -2.676525 -1.394593
O 0 -2.133769 -4.033101 -1.770295
C 0 -3.063901 -3.879018 0.474396
C 0 -0.017171 -3.216618 0.053848
C 0 1.300271 -2.439039 0.188003
C 0 2.478200 -3.334098 0.592467
C 0 3.798487 -2.567816 0.744869
C 0 4.975937 -3.466957 1.135377
S 0 -1.197541 2.335806 -0.299605
O 0 -0.698811 1.173062 -1.192788
H 0 -3.163875 -1.812972 -2.302678
H 0 -0.896852 -1.781941 -1.296042
H 0 -3.052682 0.365623 2.302678
H 0 -4.335912 3.715108 0.804701
H 0 -5.735138 2.992717 -0.054172
H 0 -5.902323 3.513326 1.650004
H 0 -0.724719 1.111777 1.739555
H 0 -1.865283 2.461361 1.991488
H 0 0.772175 2.762360 1.096060
H 0 -0.035456 4.248617 0.541888
H 0 0.767987 3.461609 -1.887099
H 0 2.961058 4.377535 -0.738665
H 0 0.868665 1.014936 -1.170437
H 0 -5.445425 -1.938154 -1.462997
H 0 -2.957448 -4.444075 -2.082925
H 0 -3.998602 -4.371219 0.193920
H 0 -3.276215 -3.212412 1.315849
H 0 -2.352889 -4.641144 0.803512
H 0 -0.268081 -3.688819 1.011541
H 0 0.096007 -4.021875 -0.680047
H 0 1.178820 -1.638553 0.928985
H 0 1.530154 -1.942123 -0.765259
H 0 2.242715 -3.841621 1.539822
H 0 2.604576 -4.129974 -0.156530
H 0 3.672980 -1.779402 1.500557
H 0 4.027861 -2.051427 -0.197981
H 0 5.148791 -4.243129 0.379474
H 0 4.788462 -3.971987 2.091214
H 0 5.902323 -2.890254 1.239004
Free Energy (PCM/B3LYP/6-31G*) = -1832.836103
Number of imaginary frequencies = 0

3-S*S*-c2
B3LYP/6-31G* geometry
O 0 -0.079311 -1.491702 -0.732081
C 0 -0.481655 0.570268 0.433425
C 0 0.128055 -1.416360 1.752608
C 0 0.053238 0.139083 1.762376
C 0 0.719301 -1.963263 0.423430
C 0 -0.514852 -0.242578 -0.644751
C 0 -1.221382 0.400988 -1.814240
N 0 -1.695972 1.649881 -1.199418
C 0 -1.065106 1.855696 0.055723
O 0 -1.686211 2.749502 -2.078987
C 0 -2.957460 3.415327 -2.057904
C 0 -2.342925 -0.450374 -2.422083
C 0 -4.762399 -1.783838 -2.347875
C 0 -5.365111 -2.991610 -1.623062
C 0 -6.186512 -2.601278 -0.379177
O 0 -4.405313 -3.987310 -1.293867
O 0 -6.825633 -1.574498 -0.287992
O 0 -6.172649 -3.531219 0.582440
O 0 -1.059584 2.918294 0.661696
O 0 1.341393 0.699469 2.047156
O 0 -1.198451 -1.925259 1.907059
C 0 0.908793 -1.944758 2.955490
C 0 2.194323 -1.675728 0.134434
C 0 2.705129 -2.335565 -1.154403
C 0 4.194122 -2.064191 -1.404742
C 0 4.722416 -2.706997 -2.693498
C 0 6.208574 -2.427778 -2.940933
S 0 -3.673025 -0.816908 -1.199985
O 0 -3.110801 -1.878879 -0.218701
H 0 -0.653212 0.413558 2.557990
H 0 0.536413 -3.041093 0.413467
H 0 -0.506761 0.634889 -2.614467
H 0 -3.765631 2.737655 -2.353142
H 0 -3.160285 3.831857 -1.067117
H 0 -2.862086 4.222089 -2.788518
H 0 -1.946598 -1.404303 -2.782776
H 0 -2.819901 0.083432 -3.250201
H 0 -4.177461 -2.134834 -3.200506

H 0 -5.543080 -1.100220 -2.688560
H 0 -6.074165 -3.458862 -2.318102
H 0 -3.681993 -3.497866 -0.832575
H 0 -5.536773 -4.222089 0.286069
H 0 1.249132 1.663392 1.965162
H 0 -1.742382 -1.747437 1.110648
H 0 0.363701 -1.682868 3.868003
H 0 1.908541 -1.513125 3.014665
H 0 0.986950 -3.036051 2.907107
H 0 2.355818 -0.592881 0.101228
H 0 2.778938 -2.048344 0.983853
H 0 2.120570 -1.973763 -2.009632
H 0 2.535840 -3.421065 -1.100291
H 0 4.362608 -0.977810 -1.446642
H 0 4.781270 -2.430833 -0.549642
H 0 4.134011 -2.341466 -3.547086
H 0 4.556277 -3.792799 -2.650931
H 0 6.825633 -2.813267 -2.119763
H 0 6.400783 -1.350595 -3.021539
H 0 6.555223 -2.898809 -3.868003
Free Energy (PCM/B3LYP/6-31G*) = -1832.835624
Number of imaginary frequencies = 0

3-S*S*-c3
B3LYP/6-31G* geometry
O 0 0.890915 -1.363066 -0.686792
C 0 3.023119 -1.408668 0.385225
C 0 1.438429 -3.234984 0.872321
C 0 2.697675 -2.478555 1.378998
C 0 0.389773 -2.166384 0.442240
C 0 2.149430 -0.966313 -0.539557
C 0 2.767085 0.077116 -1.440564
N 0 4.047923 0.310180 -0.747818
C 0 4.293595 -0.734066 0.180317
O 0 5.113010 0.608107 -1.619622
C 0 5.777534 1.808638 -1.198210
C 0 1.925977 1.337272 -1.661828
C 0 0.508483 3.564090 -0.831497
C 0 -0.722605 3.685155 0.071127
C 0 -0.376057 4.174165 1.490094
O 0 -1.500596 2.496448 0.131038
O 0 0.535784 4.933620 1.740888
O 0 -1.220169 3.728349 2.428874
O 0 5.383252 -0.978316 0.691385
O 0 3.777147 -3.391895 1.564083
O 0 0.826297 -3.916950 1.971351
C 0 1.772165 -4.222864 -0.251041
C 0 -0.968201 -2.710851 0.021582
C 0 -2.032900 -1.618574 -0.154851
C 0 -3.384993 -2.179304 -0.613771
C 0 -4.462079 -1.101636 -0.791586
C 0 -5.805890 -1.666606 -1.263817
S 0 1.701349 2.319390 -0.119178
O 0 0.863180 1.458834 0.851063
H 0 2.429717 -2.030869 2.349215
H 0 0.268923 -1.459366 1.272603
H 0 2.970527 -0.357939 -2.428874
H 0 6.233858 1.674524 -0.213095
H 0 6.554159 1.976081 -1.948124
H 0 5.086345 2.657961 -1.179481
H 0 0.938201 1.058709 -2.042193
H 0 2.415118 2.003737 -2.379948
H 0 0.198089 3.252299 -1.830459
H 0 1.041401 4.515387 -0.889924
H 0 -1.367441 4.458927 -0.364701
H 0 -0.854719 1.798159 0.404856
H 0 -1.818415 3.090444 1.977360
H 0 4.589567 -2.852625 1.598004
H 0 1.503326 -4.507109 2.343449
H 0 2.526608 -4.933620 0.095966
H 0 2.169932 -3.718172 -1.136686
H 0 0.875950 -4.778137 -0.539722
H 0 -0.856769 -3.273635 -0.913288
H 0 -1.287658 -3.424090 0.789004
H 0 -1.680386 -0.876132 -0.882621
H 0 -2.162857 -1.082650 0.796642
H 0 -3.250653 -2.716999 -1.564105
H 0 -3.735520 -2.927054 0.113014
H 0 -4.106402 -0.351218 -1.511827
H 0 -4.601869 -0.569389 0.159852
H 0 -6.202521 -2.396029 -0.546694
H 0 -5.702782 -2.175729 -2.230241
H 0 -6.554159 -0.874414 -1.382158
Free Energy (PCM/B3LYP/6-31G*) = -1832.835265
Number of imaginary frequencies = 0

3-S*S*-c4
B3LYP/6-31G* geometry
O 0 -2.054372 -1.209845 -0.340612
C 0 -1.977450 -3.502235 0.322889
C 0 -4.185167 -2.413617 0.187627
C 0 -3.387015 -3.590120 0.817679
C 0 -3.342148 -1.115924 0.373289
C 0 -1.454403 -2.389938 -0.224841
C 0 -0.035972 -2.611198 -0.701859
N 0 0.221992 -3.963275 -0.172015
C 0 -0.998194 -4.571238 0.209164
O 0 1.045437 -4.738799 -1.017088
C 0 2.029321 -5.446027 -0.240155
C 0 0.911222 -1.521146 -0.193409
C 0 3.085725 0.132013 -0.525485
C 0 4.623042 0.287259 -0.474178
C 0 5.191970 0.037156 0.940818
O 0 4.957899 1.597609 -0.866341
O 0 5.867961 0.904576 1.471576
O 0 4.930287 -1.112831 1.531989
O 0 -1.158109 -5.774107 0.403591
O 0 -3.999460 -4.832908 0.484188
O 0 -5.366797 -2.180783 0.959521
C 0 -4.548115 -2.684141 -1.276600
C 0 -4.008089 0.169598 -0.097410
C 0 -3.238137 1.437673 0.298386
C 0 -3.924847 2.721441 -0.184422
C 0 -3.167242 3.998228 0.201906
C 0 -3.851985 5.277433 -0.290311
S 0 2.585473 -1.593009 -0.962165
O 0 3.472115 -2.513051 -0.091638
H 0 -3.421815 -3.448196 1.909551
H 0 -3.087778 -1.035426 1.437960
H 0 -0.011065 -2.649722 -1.799325
H 0 2.638013 -5.969002 -0.981974
H 0 2.646223 -4.745136 0.326138
H 0 1.545523 -6.170633 0.420300
H 0 1.047652 -1.575890 0.890872
H 0 0.499470 -0.542813 -0.461689
H 0 2.611279 0.422265 0.416531
H 0 2.698702 0.764811 -1.328537
H 0 5.094386 -0.439432 -1.154357
H 0 5.492510 1.948667 -0.123334
H 0 4.358348 -1.717102 0.949380
H 0 -3.312534 -5.518439 0.590315
H 0 -5.867961 -3.013849 0.959507
H 0 -5.114403 -3.616230 -1.346837
H 0 -3.661031 -2.784044 -1.909551
H 0 -5.164462 -1.870038 -1.666073
H 0 -4.129460 0.134188 -1.186866
H 0 -5.013497 0.191579 0.336683
H 0 -2.221139 1.392389 -0.112136
H 0 -3.130660 1.470099 1.392281
H 0 -4.037115 2.684286 -1.278186
H 0 -4.944629 2.765951 0.225777
H 0 -2.146904 3.949469 -0.204379
H 0 -3.058857 4.037827 1.295088
H 0 -4.862439 5.371296 0.126619
H 0 -3.943479 5.281520 -1.383607
H 0 -3.287054 6.170633 0.000431
Free Energy (PCM/B3LYP/6-31G*) = -1832.834698
Number of imaginary frequencies = 0

3-S*S*-c5
B3LYP/6-31G* geometry
O 0 0.339294 -1.164636 0.336781
C 0 -0.998223 -2.823147 -0.747047
C 0 1.395327 -2.708849 -1.324500

C 0 -0.003392 -3.176760 -1.807916
C 0 1.251695 -1.243051 -0.816605
C 0 -0.766942 -1.889605 0.195209
C 0 -1.925467 -1.760253 1.154067
N 0 -2.744417 -2.913680 0.726973
C 0 -2.304838 -3.428279 -0.507043
O 0 -4.124455 -2.813421 0.920593
C 0 -4.561137 -3.759353 1.912490
C 0 -2.660158 -0.413078 1.095810
C 0 -4.255232 1.469755 -0.142685
C 0 -4.366085 2.498306 -1.292445
C 0 -3.287238 3.601233 -1.199443
O 0 -5.634453 3.106319 -1.234021
O 0 -3.636379 4.770445 -1.148817
O 0 -2.018102 3.242734 -1.181307
O 0 -2.898381 -4.263124 -1.183534
O 0 0.024531 -4.573872 -2.087567
O 0 2.272462 -2.614596 -2.450667
C 0 1.982886 -3.650112 -0.266613
C 0 2.549746 -0.568276 -0.395314
C 0 2.392725 0.933042 -0.114661
C 0 3.716012 1.606112 0.270622
C 0 3.573800 3.106808 0.554852
C 0 4.898597 3.777711 0.931825
S 0 -3.175869 0.045173 -0.613113
O 0 -1.934509 0.658524 -1.306702
H 0 -0.220951 -2.623092 -2.734800
H 0 0.769580 -0.660167 -1.611306
H 0 -1.590088 -1.896363 2.189845
H 0 -5.636151 -3.590400 2.005439
H 0 -4.368616 -4.782591 1.577439
H 0 -4.068977 -3.577940 2.873498
H 0 -2.007891 0.385806 1.460515
H 0 -3.567842 -0.459098 1.703046
H 0 -3.888349 1.924038 0.782170
H 0 -5.245256 1.042942 0.038142
H 0 -4.238639 1.989361 -2.260419
H 0 -5.445119 4.066453 -1.174271
H 0 -1.907321 2.232984 -1.231879
H 0 -0.903301 -4.847406 -2.208081
H 0 2.273219 -3.490109 -2.873498
H 0 2.034313 -4.666111 -0.666337
H 0 1.374180 -3.677805 0.642456
H 0 2.992946 -3.328501 -0.000158
H 0 2.948142 -1.072996 0.493376
H 0 3.273188 -0.723319 -1.203176
H 0 1.659627 1.081542 0.688640
H 0 1.979319 1.426409 -1.006279
H 0 4.134204 1.107536 1.157784
H 0 4.448460 1.457300 -0.536697
H 0 2.845164 3.253532 1.364772
H 0 3.150644 3.603581 -0.329785
H 0 5.636151 3.677976 0.125744
H 0 5.330105 3.324366 1.833018
H 0 4.763050 4.847406 1.129058
Free Energy (PCM/B3LYP/6-31G*) = -1832.834616
Number of imaginary frequencies = 0

3-S*S*-c6
B3LYP/6-31G* geometry
O 0 1.723712 -1.178342 -0.147909
C 0 1.560323 -3.541196 -0.455609
C 0 3.722066 -2.622033 0.293016
C 0 3.028550 -3.827726 -0.401687
C 0 3.177419 -1.318135 -0.363390
C 0 1.042592 -2.308301 -0.295413
C 0 -0.469184 -2.331925 -0.307318
N 0 -0.715653 -3.725439 -0.720659
C 0 0.464334 -4.492396 -0.549395
O 0 -1.887421 -4.261452 -0.138324
C 0 -2.624537 -5.018783 -1.114165
C 0 -1.076647 -1.290146 -1.248803
C 0 -3.332809 -0.936027 0.384440
C 0 -4.711036 -0.240191 0.485172
C 0 -4.597296 1.297933 0.385709
O 0 -5.293868 -0.567536 1.723942
O 0 -5.037056 1.985782 1.293934
O 0 -4.027356 1.820698 -0.682701
O 0 0.508044 -5.718766 -0.508185
O 0 3.317888 -5.030605 0.305359
O 0 5.113843 -2.627590 -0.036946
C 0 3.531866 -2.647251 1.813711
C 0 3.814696 -0.024872 0.125193
C 0 3.422596 1.203187 -0.708815
C 0 4.068783 2.496151 -0.195774
C 0 3.693002 3.733300 -1.021272
C 0 4.333324 5.023235 -0.498110
S 0 -2.915151 -1.365571 -1.365014
O 0 -3.304506 -0.134462 -2.216623
H 0 3.446790 -3.892356 -1.418625
H 0 3.309537 -1.412365 -1.448822
H 0 -0.842336 -2.194532 0.715733
H 0 -2.047475 -5.887935 -1.439262
H 0 -3.526159 -5.342299 -0.589403
H 0 -2.892355 -4.394257 -1.971824
H 0 -0.731061 -1.447356 -2.274289
H 0 -0.818003 -0.274652 -0.933535
H 0 -2.536507 -0.326287 0.821196
H 0 -3.388346 -1.889348 0.915945
H 0 -5.360970 -0.580863 -0.335381
H 0 -5.467956 0.297485 2.151366
H 0 -3.720981 1.105890 -1.338288
H 0 2.634087 -5.677067 0.047004
H 0 5.467956 -3.482824 0.260272
H 0 3.901202 -3.594914 2.213500
H 0 2.479722 -2.551841 2.099246
H 0 4.093333 -1.830570 2.274289
H 0 3.540955 0.136153 1.174949
H 0 4.900424 -0.166144 0.094935
H 0 2.330653 1.315660 -0.706215
H 0 3.715803 1.038376 -1.755771
H 0 3.775573 2.657622 0.852195
H 0 5.162822 2.380503 -0.192346
H 0 2.599359 3.844058 -1.028675
H 0 3.990797 3.574386 -2.067394
H 0 5.428249 4.954811 -0.510517
H 0 4.026054 5.226227 0.535363
H 0 4.046235 5.887935 -1.107484
Free Energy (PCM/B3LYP/6-31G*) = -1832.834481
Number of imaginary frequencies = 0

3-S*S*-c7
B3LYP/6-31G* geometry
O 0 -0.161245 -1.591339 -0.753021
C 0 -0.797516 -0.015306 0.947310
C 0 -0.130496 -2.283030 1.647832
C 0 -0.322342 -0.812255 2.122049
C 0 0.585923 -2.361171 0.268826
C 0 -0.697451 -0.455843 -0.325324
C 0 -1.327679 0.503873 -1.307381
N 0 -1.588127 1.651415 -0.418810
C 0 -1.479872 1.283263 0.940592
O 0 -2.671016 2.468356 -0.761710
C 0 -2.210061 3.781167 -1.121599
C 0 -2.576154 -0.044348 -2.013717
C 0 -5.149727 -1.060697 -2.077606
C 0 -5.809084 -2.419578 -1.821401
C 0 -6.411160 -2.540971 -0.408238
O 0 -4.953658 -3.521613 -2.094685
O 0 -6.890964 -1.609479 0.202487
O 0 -6.403973 -3.790749 0.068896
O 0 -1.859669 1.965191 1.882232
O 0 0.905260 -0.309496 2.666194
O 0 -1.418012 -2.888926 1.533461
C 0 0.612484 -3.113763 2.694214
C 0 2.054511 -1.935457 0.207548
C 0 2.692876 -2.146815 -1.172982
C 0 4.176544 -1.758164 -1.203777
C 0 4.830227 -1.962302 -2.576530
C 0 6.310635 -1.568150 -2.603373
S 0 -3.806242 -0.748125 -0.837865
O 0 -3.292916 -2.164019 -0.461391
H 0 -1.094565 -0.829482 2.903141
H 0 0.478378 -3.390213 -0.084557

H 0 -0.611962 0.781429 -2.090927
H 0 -1.717738 4.261987 -0.271221
H 0 -1.527895 3.739152 -1.977143
H 0 -3.114129 4.331527 -1.391644
H 0 -2.295620 -0.846388 -2.702968
H 0 -3.077559 0.758711 -2.559826
H 0 -4.725133 -1.053215 -3.083530
H 0 -5.869521 -0.246504 -1.970565
H 0 -6.653370 -2.499554 -2.517876
H 0 -4.126302 -3.329678 -1.590670
H 0 -5.907245 -4.331527 -0.586890
H 0 0.742472 0.610289 2.932559
H 0 -1.950684 -2.473293 0.821364
H 0 -0.005404 -3.171702 3.596031
H 0 1.573074 -2.672672 2.961302
H 0 0.770832 -4.132378 2.324336
H 0 2.146091 -0.889056 0.518336
H 0 2.604988 -2.526187 0.949173
H 0 2.147894 -1.562031 -1.924645
H 0 2.586242 -3.202055 -1.464691
H 0 4.282658 -0.704542 -0.905363
H 0 4.723872 -2.344789 -0.450981
H 0 4.281598 -1.377506 -3.328519
H 0 4.726055 -3.015522 -2.873505
H 0 6.890964 -2.161133 -1.885524
H 0 6.443122 -0.510726 -2.342327
H 0 6.748595 -1.724113 -3.596031
Free Energy (PCM/B3LYP/6-31G*) = -1832.834198
Number of imaginary frequencies = 0

4-3*E*-c1
B3LYP/6-31G* geometry
O 0 2.074472 -1.399076 -0.195201
C 0 2.909888 -3.611922 -0.603416
C 0 4.523813 -1.873430 0.039872
C 0 4.353168 -3.240938 -0.676520
C 0 3.424753 -0.913306 -0.510442
C 0 1.919921 -2.719620 -0.363188
C 0 0.631473 -3.408976 -0.290178
N 0 0.954151 -4.766122 -0.515643
C 0 2.323181 -4.946336 -0.657441
S 0 -1.020443 -1.245922 0.201606
C 0 -2.808864 -1.361153 0.596414
C 0 -3.720943 -1.604262 -0.621647
C 0 -5.184761 -1.491785 -0.194671
O 0 -3.511262 -2.872351 -1.204131
O 0 -5.940022 -2.444385 -0.202481
O 0 -5.520630 -0.258167 0.196627
O 0 2.909969 -6.030178 -0.778854
O 0 5.195263 -4.225897 -0.078147
O 0 5.759364 -1.278055 -0.372706
C 0 3.504645 0.519927 -0.000774
C 0 2.568617 1.487841 -0.738405
C 0 2.686531 2.929113 -0.226608
C 0 1.766147 3.913528 -0.959437
C 0 1.890723 5.351638 -0.445512
C 0 -0.613191 -2.935608 -0.070737
C 0 4.483690 -2.012462 1.565543
H 0 4.667822 -3.088509 -1.722020
H 0 3.503900 -0.920697 -1.605833
H 0 0.306768 -5.532300 -0.392877
H 0 -3.044813 -0.395271 1.051077
H 0 -2.961617 -2.140872 1.348126
H 0 -3.524097 -0.829229 -1.372416
H 0 -4.284242 -3.414466 -0.952810
H 0 -6.457672 -0.268658 0.475048
H 0 4.805229 -5.090271 -0.313824
H 0 6.457672 -1.922464 -0.167146
H 0 3.280044 0.531505 1.072931
H 0 4.543302 0.848541 -0.115166
H 0 1.530363 1.147200 -0.635028
H 0 2.798112 1.463688 -1.813779
H 0 2.458457 2.952892 0.849436
H 0 3.729246 3.266045 -0.324827
H 0 0.724313 3.577471 -0.859640
H 0 1.993166 3.888524 -2.034766
H 0 1.223481 6.030178 -0.989502
H 0 2.915353 5.725878 -0.562684
H 0 1.635147 5.415183 0.619593
H 0 -1.458937 -3.614112 -0.084439
H 0 3.511549 -2.368991 1.919072
H 0 4.693188 -1.047784 2.034766
H 0 5.241970 -2.730576 1.888213
Free Energy (PCM/B3LYP/6-31G*) = -1642.022747
Number of imaginary frequencies = 0

4-3*E*-c2
B3LYP/6-31G* geometry
O 0 1.257502 -1.417736 -0.483605
C 0 2.414817 -3.511639 -0.673993
C 0 3.582438 -1.647146 0.422680
C 0 3.770770 -2.990682 -0.333439
C 0 2.578737 -0.780739 -0.398929
C 0 1.304452 -2.737379 -0.710950
C 0 0.132639 -3.557364 -1.020374
N 0 0.652578 -4.863934 -1.153659
C 0 2.018419 -4.895805 -0.907996
S 0 -1.816214 -1.595917 -0.988724
C 0 -3.567050 -1.858108 -1.480528
C 0 -4.457296 -2.519975 -0.417442
C 0 -4.433620 -1.770052 0.911925
O 0 -4.087540 -3.863132 -0.198943
O 0 -4.140690 -2.312253 1.959832
O 0 -4.776330 -0.483664 0.791006
O 0 2.732292 -5.907087 -0.873021
O 0 4.519518 -3.909892 0.461185
O 0 4.809365 -0.909401 0.381662
C 0 2.352709 0.626739 0.138339
C 0 1.557708 1.531196 -0.814006
C 0 1.358164 2.945576 -0.254653
C 0 0.562229 3.864731 -1.189947
C 0 0.365119 5.274935 -0.624128
C 0 -1.166014 -3.221601 -1.169030
C 0 3.137396 -1.859132 1.873730
H 0 4.342953 -2.758244 -1.246701
H 0 2.959113 -0.727649 -1.427851
H 0 0.086060 -5.696356 -1.237432
H 0 -3.596073 -2.453741 -2.396051
H 0 -3.946412 -0.859967 -1.713168
H 0 -5.490455 -2.474636 -0.796885
H 0 -3.903406 -3.941857 0.757216
H 0 -4.739058 -0.071711 1.676606
H 0 4.306281 -4.798460 0.115039
H 0 5.490455 -1.486490 0.766652
H 0 1.837031 0.562188 1.104337
H 0 3.338615 1.064024 0.329982
H 0 0.578871 1.079596 -1.020106
H 0 2.081623 1.591427 -1.779150
H 0 0.842886 2.883165 0.715462
H 0 2.339810 3.398208 -0.049799
H 0 -0.418288 3.411371 -1.393639
H 0 1.076527 3.927577 -2.159503
H 0 -0.205588 5.907087 -1.314214
H 0 1.329022 5.765957 -0.441079
H 0 -0.177678 5.246423 0.328962
H 0 -1.900252 -3.988675 -1.390319
H 0 2.154229 -2.335316 1.935465
H 0 3.094015 -0.899932 2.396051
H 0 3.855521 -2.504437 2.386353
Free Energy (PCM/B3LYP/6-31G*) = -1642.022506
Number of imaginary frequencies = 0

4-3*E*-c3
B3LYP/6-31G* geometry
O 0 2.165395 -1.399427 -0.588288
C 0 2.909529 -3.669873 -0.814719
C 0 4.411981 -2.071196 0.293690
C 0 4.341695 -3.416607 -0.479624
C 0 3.584085 -1.023689 -0.511226
C 0 1.962647 -2.701820 -0.826437
C 0 0.654848 -3.284742 -1.130242
N 0 0.923036 -4.658558 -1.315454
C 0 2.260234 -4.950451 -1.072123
S 0 -0.900272 -0.995402 -1.033661

C 0 -2.733239 -1.017745 -1.076256
C 0 -3.381552 -1.499118 0.240404
C 0 -4.901822 -1.482351 0.084203
O 0 -3.019762 -0.697142 1.338993
O 0 -5.609431 -0.689272 0.673834
O 0 -5.346866 -2.401130 -0.780700
O 0 2.769997 -6.077925 -1.060050
O 0 4.908758 -4.468041 0.301126
O 0 5.754831 -1.575591 0.252516
C 0 3.631270 0.397914 0.034326
C 0 3.049667 1.441971 -0.929480
C 0 3.112904 2.868069 -0.368227
C 0 2.551803 3.924944 -1.328209
C 0 2.612957 5.347297 -0.761717
C 0 -0.561726 -2.708872 -1.236261
C 0 3.941358 -2.214903 1.744801
H 0 4.942496 -3.284996 -1.394433
H 0 3.959269 -1.035149 -1.543363
H 0 0.208648 -5.371855 -1.366193
H 0 -3.067292 -1.622435 -1.923527
H 0 -3.018208 0.022016 -1.262829
H 0 -3.069539 -2.531316 0.439689
H 0 -3.775133 -0.098995 1.495991
H 0 -6.317619 -2.309643 -0.851781
H 0 4.543493 -5.298608 -0.060786
H 0 6.317619 -2.274823 0.626031
H 0 3.093983 0.432419 0.990042
H 0 4.679986 0.632012 0.246650
H 0 2.007768 1.187668 -1.163195
H 0 3.600172 1.400845 -1.880700
H 0 2.558915 2.910349 0.581251
H 0 4.156350 3.117453 -0.124166
H 0 1.510448 3.673137 -1.574381
H 0 3.107756 3.884059 -2.275669
H 0 2.206216 6.077925 -1.470459
H 0 3.646310 5.639678 -0.537148
H 0 2.036687 5.427771 0.168382
H 0 -1.416429 -3.331729 -1.487196
H 0 2.890059 -2.511411 1.806739
H 0 4.068956 -1.267912 2.275669
H 0 4.535759 -2.981486 2.248694
Free Energy (PCM/B3LYP/6-31G*) = -1642.021980
Number of imaginary frequencies = 0

4-3*E*-c4
B3LYP/6-31G* geometry
O 0 1.134086 -1.356592 0.133047
C 0 2.268761 -3.395332 -0.425344
C 0 3.626307 -1.368898 -0.111357
C 0 3.580672 -2.774018 -0.771821
C 0 2.284465 -0.648800 -0.445646
C 0 1.194074 -2.687633 -0.003703
C 0 0.086426 -3.595406 0.302235
N 0 0.605349 -4.878535 0.024746
C 0 1.936122 -4.814691 -0.368923
S 0 -1.796317 -1.759441 1.168360
C 0 -3.584652 -2.134666 1.359259
C 0 -4.421023 -1.922743 0.082095
C 0 -4.007109 -2.855943 -1.050633
O 0 -4.347014 -0.587838 -0.359826
O 0 -3.581076 -2.446537 -2.112490
O 0 -4.157945 -4.149916 -0.740971
O 0 2.677166 -5.779414 -0.595879
O 0 4.685471 -3.564729 -0.335123
O 0 4.628552 -0.577249 -0.759018
C 0 2.191013 0.797306 0.023654
C 0 0.976391 1.546415 -0.542997
C 0 0.900975 3.002852 -0.068194
C 0 -0.302928 3.765228 -0.636526
C 0 -0.378695 5.218659 -0.157709
C 0 -1.165070 -3.345787 0.742670
C 0 3.901048 -1.448626 1.394237
H 0 3.662748 -2.611912 -1.859084
H 0 2.153313 -0.692358 -1.535214
H 0 0.146533 -5.743305 0.275406
H 0 -3.943371 -1.429134 2.112490
H 0 -3.709982 -3.145682 1.755130
H 0 -5.461825 -2.164312 0.343767
H 0 -3.895952 -0.613278 -1.225106
H 0 -3.866955 -4.683868 -1.506502
H 0 4.432902 -4.493567 -0.501716
H 0 5.461825 -1.072627 -0.685588
H 0 2.163592 0.817325 1.120168
H 0 3.114484 1.300045 -0.283436
H 0 0.055314 1.021865 -0.258461
H 0 1.019443 1.523622 -1.641793
H 0 0.857897 3.025637 1.030903
H 0 1.827018 3.526814 -0.348215
H 0 -1.227286 3.239269 -0.358469
H 0 -0.258375 3.743602 -1.734743
H 0 -1.247887 5.734404 -0.582086
H 0 0.517730 5.779414 -0.450521
H 0 -0.459953 5.271894 0.935131
H 0 -1.847214 -4.178637 0.889597
H 0 3.101119 -1.968839 1.929643
H 0 4.003383 -0.443183 1.810657
H 0 4.831484 -1.995144 1.568898
Free Energy (PCM/B3LYP/6-31G*) = -1642.021946
Number of imaginary frequencies = 0

4-3*E*-c5
B3LYP/6-31G* geometry
O 0 1.970702 -1.255216 -0.361541
C 0 3.553972 -3.022966 -0.719610
C 0 4.402177 -0.865811 0.100993
C 0 4.780055 -2.175767 -0.643507
C 0 3.089507 -0.320018 -0.539913
C 0 2.298874 -2.537599 -0.568437
C 0 1.327642 -3.629877 -0.642824
N 0 2.119438 -4.781746 -0.839759
C 0 3.473255 -4.473753 -0.851571
S 0 -1.007817 -2.190378 -0.282954
C 0 -2.682942 -2.926191 -0.501946
C 0 -3.714637 -2.028793 0.204546
C 0 -5.118437 -2.571473 -0.061413
O 0 -3.642326 -0.687773 -0.226110
O 0 -5.915907 -1.996308 -0.774705
O 0 -5.344393 -3.742643 0.544640
O 0 4.406406 -5.283292 -0.932090
O 0 5.853299 -2.836575 0.026076
O 0 5.383417 0.137209 -0.185736
C 0 2.624236 1.028292 -0.005570
C 0 1.472229 1.645946 -0.810935
C 0 1.040955 3.014564 -0.268972
C 0 -0.111234 3.647031 -1.060017
C 0 -0.538924 5.013100 -0.513588
C 0 -0.019982 -3.618011 -0.557933
C 0 4.284336 -1.078031 1.614229
H 0 5.114703 -1.878690 -1.651075
H 0 3.259501 -0.249392 -1.622583
H 0 1.767015 -5.728350 -0.815982
H 0 -2.694582 -3.926819 -0.062903
H 0 -2.908398 -2.996418 -1.570101
H 0 -3.527694 -2.050086 1.284023
H 0 -4.317764 -0.586940 -0.923293
H 0 -6.242654 -4.043815 0.303432
H 0 5.816365 -3.768666 -0.264356
H 0 6.242654 -0.235727 0.074547
H 0 2.323499 0.914585 1.043153
H 0 3.489670 1.699703 -0.019971
H 0 0.612479 0.963823 -0.805352
H 0 1.779642 1.750401 -1.861757
H 0 0.742051 2.910510 0.784688
H 0 1.903407 3.697697 -0.275371
H 0 -0.972029 2.963454 -1.052656
H 0 0.187101 3.750769 -2.113001
H 0 -1.363112 5.437604 -1.098433
H 0 0.292609 5.728350 -0.540780
H 0 -0.874256 4.935055 0.528124
H 0 -0.560747 -4.555909 -0.651222
H 0 3.479583 -1.774344 1.868685
H 0 4.092013 -0.124694 2.113001
H 0 5.219789 -1.491707 1.999831
Free Energy (PCM/B3LYP/6-31G*) = -1642.021600

Number of imaginary frequencies = 0

4-3*E*-c6
B3LYP/6-31G* geometry
O 0 1.150904 -1.400207 0.232985
C 0 1.509724 -3.709554 -0.309862
C 0 3.485251 -2.290316 0.049151
C 0 2.964411 -3.590466 -0.622397
C 0 2.492174 -1.143256 -0.311506
C 0 0.744679 -2.668139 0.090882
C 0 -0.617575 -3.128694 0.378329
N 0 -0.576359 -4.512376 0.113907
C 0 0.696373 -4.921798 -0.264560
S 0 -1.729728 -0.729934 1.190261
C 0 -3.526145 -0.442730 1.456496
C 0 -4.346319 -0.287579 0.167892
C 0 -4.651827 -1.637728 -0.510870
O 0 -3.779552 0.620206 -0.770519
O 0 -5.008173 -2.625552 0.097974
O 0 -4.533105 -1.617294 -1.841932
O 0 1.051897 -6.084421 -0.487960
O 0 3.710574 -4.716703 -0.163495
O 0 4.717578 -1.903444 -0.568718
C 0 2.903082 0.243026 0.167321
C 0 2.046750 1.374070 -0.419732
C 0 2.495554 2.762427 0.053576
C 0 1.654920 3.905631 -0.529349
C 0 2.110437 5.290194 -0.056826
C 0 -1.704640 -2.451343 0.800522
C 0 3.676698 -2.458893 1.560189
H 0 3.123741 -3.470546 -1.706445
H 0 2.381395 -1.138335 -1.404057
H 0 -1.326133 -5.156763 0.322674
H 0 -3.562556 0.491350 2.021888
H 0 -3.946495 -1.238311 2.074850
H 0 -5.320600 0.130033 0.450882
H 0 -2.807569 0.523565 -0.722286
H 0 -4.225023 -0.710963 -2.074850
H 0 3.158612 -5.500379 -0.350350
H 0 5.320600 -2.660943 -0.480566
H 0 2.858836 0.272681 1.262982
H 0 3.951464 0.386215 -0.115765
H 0 0.994627 1.218209 -0.148942
H 0 2.094768 1.330962 -1.517660
H 0 2.451791 2.804378 1.152063
H 0 3.551268 2.914904 -0.215800
H 0 0.600650 3.754227 -0.257401
H 0 1.696393 3.861459 -1.627010
H 0 1.491567 6.084421 -0.490121
H 0 3.151327 5.483544 -0.344572
H 0 2.047512 5.376169 1.035120
H 0 -2.641278 -2.979153 0.951371
H 0 2.733885 -2.676384 2.070937
H 0 4.103525 -1.547890 1.987503
H 0 4.361050 -3.289190 1.752814
Free Energy (PCM/B3LYP/6-31G*) = -1642.021418
Number of imaginary frequencies = 0

4-3*E*-c7
B3LYP/6-31G* geometry
O 0 0.681002 -0.660443 0.285080
C 0 2.770650 -1.726937 -0.214438
C 0 2.803556 0.656826 0.401287
C 0 3.578552 -0.476756 -0.327322
C 0 1.324988 0.605128 -0.090167
C 0 1.450719 -1.737625 0.086804
C 0 0.965828 -3.114948 0.174623
N 0 2.103167 -3.900066 -0.112748
C 0 3.232166 -3.108566 -0.295744
S 0 -1.648091 -2.594446 0.892001
C 0 -2.984134 -3.864795 0.848240
C 0 -4.342334 -3.161079 0.767826
C 0 -4.518560 -2.352481 -0.517974
O 0 -4.516656 -2.306103 1.877092
O 0 -4.836511 -1.178523 -0.507804
O 0 -4.323719 -3.077292 -1.623254
O 0 4.387823 -3.516123 -0.467202
O 0 4.883478 -0.624806 0.231854
O 0 3.288478 1.925094 -0.053428
C 0 0.419443 1.716637 0.425101
C 0 -0.950240 1.744691 -0.269225
C 0 -1.889234 2.817688 0.294907
C 0 -3.264414 2.832921 -0.385672
C 0 -4.211362 3.890801 0.190164
C 0 -0.267621 -3.589481 0.451982
C 0 2.926904 0.554381 1.925035
H 0 3.672752 -0.164468 -1.380307
H 0 1.345037 0.625153 -1.188215
H 0 2.153318 -4.899138 0.031209
H 0 -2.946594 -4.466805 1.759407
H 0 -2.843748 -4.511400 -0.020171
H 0 -5.115571 -3.946054 0.759430
H 0 -4.823242 -1.452611 1.513962
H 0 -4.468949 -2.499822 -2.398433
H 0 5.183665 -1.518874 -0.021960
H 0 4.245331 1.932370 0.118240
H 0 0.284470 1.595080 1.506903
H 0 0.940320 2.667745 0.270504
H 0 -1.427502 0.761381 -0.172672
H 0 -0.805704 1.914905 -1.346139
H 0 -2.022259 2.652432 1.374452
H 0 -1.420687 3.807914 0.192873
H 0 -3.723939 1.838459 -0.292583
H 0 -3.132391 3.007563 -1.463032
H 0 -5.183665 3.875194 -0.315816
H 0 -3.793445 4.899138 0.078775
H 0 -4.389127 3.722675 1.259691
H 0 -0.435842 -4.662994 0.453978
H 0 2.482785 -0.368310 2.310220
H 0 2.429586 1.405015 2.398433
H 0 3.982318 0.563337 2.209767
Free Energy (PCM/B3LYP/6-31G*) = -1642.021069
Number of imaginary frequencies = 0

4-3*E*-c8
B3LYP/6-31G* geometry
O 0 2.297250 -1.395515 -0.144715
C 0 3.171567 -3.590544 -0.567258
C 0 4.762709 -1.811473 0.020507
C 0 4.603144 -3.186701 -0.683434
C 0 3.626028 -0.880177 -0.502067
C 0 2.168304 -2.719884 -0.303773
C 0 0.897831 -3.437185 -0.197426
N 0 1.244655 -4.787878 -0.427373
C 0 2.614046 -4.937898 -0.600722
S 0 -0.784508 -1.307628 0.332408
C 0 -2.574309 -1.449095 0.704557
C 0 -3.470790 -1.679077 -0.511973
C 0 -4.931537 -1.380300 -0.141486
O 0 -3.275619 -3.001798 -0.971614
O 0 -5.283806 -0.444591 0.545497
O 0 -5.780920 -2.247386 -0.719871
O 0 3.221978 -6.009002 -0.730933
O 0 5.484758 -4.148726 -0.104988
O 0 5.971557 -1.190340 -0.431493
C 0 3.687989 0.557385 -0.002185
C 0 2.706289 1.499197 -0.713711
C 0 2.811529 2.946891 -0.217376
C 0 1.842166 3.904813 -0.921623
C 0 1.955862 5.349957 -0.425154
C 0 -0.351543 -2.988538 0.046540
C 0 4.770867 -1.942218 1.547436
H 0 4.883935 -3.033177 -1.738383
H 0 3.672852 -0.891953 -1.599275
H 0 0.621347 -5.568092 -0.272242
H 0 -2.837141 -0.494743 1.164823
H 0 -2.730210 -2.240168 1.443477
H 0 -3.209737 -0.944483 -1.290710
H 0 -3.857057 -3.148636 -1.735182
H 0 -6.690203 -1.970249 -0.493160
H 0 5.107657 -5.022987 -0.324672
H 0 6.690203 -1.817465 -0.243325
H 0 3.497915 0.569881 1.078123
H 0 4.714468 0.909383 -0.151516

H 0 1.679897 1.137710 -0.570939
H 0 2.898046 1.471035 -1.796365
H 0 2.624249 2.974502 0.866412
H 0 3.842012 3.305642 -0.358007
H 0 0.812559 3.547699 -0.777933
H 0 2.027239 3.874696 -2.004836
H 0 1.252859 6.009002 -0.947655
H 0 2.966631 5.744906 -0.586471
H 0 1.741646 5.417871 0.648759
H 0 -1.181832 -3.686219 0.055399
H 0 3.816825 -2.314950 1.932248
H 0 4.975799 -0.970900 2.004836
H 0 5.552307 -2.643651 1.850954
Free Energy (PCM/B3LYP/6-31G*) = -1642.020932
Number of imaginary frequencies = 0

4-3*E*-c9
B3LYP/6-31G* geometry
O 0 0.944322 -1.334901 -1.263126
C 0 2.444983 -2.264352 0.378955
C 0 3.285747 -0.525296 -1.158676
C 0 3.574797 -1.314587 0.152250
C 0 1.789357 -0.123724 -1.282399
C 0 1.272439 -2.213386 -0.302711
C 0 0.385188 -3.293580 0.129012
N 0 1.133277 -3.979189 1.105880
C 0 2.371744 -3.384712 1.324560
S 0 -1.772241 -2.801816 -1.534177
C 0 -3.447314 -3.506813 -1.277513
C 0 -4.212123 -2.941392 -0.067728
C 0 -4.294542 -1.417693 -0.103665
O 0 -3.629150 -3.348321 1.148945
O 0 -3.861407 -0.722077 0.795495
O 0 -4.884543 -0.953042 -1.208395
O 0 3.206498 -3.748585 2.155070
O 0 3.750658 -0.404180 1.248337
O 0 3.603048 -1.384297 -2.265502
C 0 1.255005 0.886302 -0.264580
C 0 -0.211896 1.275889 -0.496349
C 0 -0.697699 2.350400 0.485235
C 0 -2.169346 2.736889 0.286646
C 0 -2.643647 3.817854 1.263776
C 0 -0.866215 -3.614647 -0.263305
C 0 4.206484 0.682279 -1.308172
H 0 4.511114 -1.865477 -0.015991
H 0 1.640331 0.253061 -2.297859
H 0 0.778947 -4.738374 1.670489
H 0 -3.372352 -4.592696 -1.176140
H 0 -3.990784 -3.287025 -2.199930
H 0 -5.240526 -3.328858 -0.132299
H 0 -3.365186 -2.529992 1.611939
H 0 -4.892311 0.023501 -1.165404
H 0 3.831188 -0.951895 2.047232
H 0 2.935421 -2.089105 -2.303799
H 0 1.392533 0.496354 0.749626
H 0 1.880634 1.784583 -0.334648
H 0 -0.848498 0.386917 -0.406494
H 0 -0.333934 1.641303 -1.526799
H 0 -0.554436 1.992228 1.515663
H 0 -0.069293 3.248016 0.384756
H 0 -2.795762 1.841143 0.398004
H 0 -2.314410 3.088195 -0.745177
H 0 -3.697193 4.072187 1.099008
H 0 -2.056986 4.738374 1.152386
H 0 -2.540278 3.483847 2.303799
H 0 -1.375661 -4.448712 0.207568
H 0 4.135858 1.352698 -0.450919
H 0 3.962445 1.234470 -2.221847
H 0 5.240526 0.331218 -1.386647
Free Energy (PCM/B3LYP/6-31G*) = -1642.020830
Number of imaginary frequencies = 0

4-3*E*-c10
B3LYP/6-31G* geometry
O 0 1.245483 -1.417012 -0.491338
C 0 2.446991 -3.485722 -0.682340
C 0 3.566672 -1.602090 0.432932
C 0 3.788765 -2.939197 -0.325678
C 0 2.553033 -0.753471 -0.394872
C 0 1.321619 -2.733837 -0.725145
C 0 0.170016 -3.574593 -1.053856
N 0 0.717846 -4.868481 -1.197493
C 0 2.081190 -4.875430 -0.932886
S 0 -1.814876 -1.651129 -1.014388
C 0 -3.562238 -1.940744 -1.503858
C 0 -4.446796 -2.550953 -0.414061
C 0 -4.516852 -1.643201 0.826495
O 0 -3.987223 -3.839427 -0.001432
O 0 -4.742350 -0.454079 0.758031
O 0 -4.331945 -2.277916 1.988262
O 0 2.813801 -5.872869 -0.896872
O 0 4.547383 -3.846056 0.473851
O 0 4.778830 -0.839870 0.405265
C 0 2.294310 0.648375 0.142283
C 0 1.489307 1.537325 -0.816354
C 0 1.255715 2.947089 -0.258621
C 0 0.447266 3.848456 -1.200563
C 0 0.214153 5.253986 -0.636856
C 0 -1.133264 -3.261670 -1.211079
C 0 3.112676 -1.827140 1.879252
H 0 4.365555 -2.692497 -1.232285
H 0 2.940185 -0.690964 -1.420735
H 0 0.168003 -5.712077 -1.281613
H 0 -3.586952 -2.559845 -2.403577
H 0 -3.948873 -0.950861 -1.757219
H 0 -5.467782 -2.614154 -0.811652
H 0 -4.587029 -4.519798 -0.345038
H 0 -4.146596 -3.217995 1.760039
H 0 4.358779 -4.737106 0.120238
H 0 5.467782 -1.403903 0.795589
H 0 1.771892 0.572576 1.103788
H 0 3.269586 1.105277 0.342395
H 0 0.521681 1.066083 -1.030930
H 0 2.020147 1.608617 -1.776973
H 0 0.734331 2.873842 0.707408
H 0 2.225871 3.420685 -0.046478
H 0 -0.521242 3.373314 -1.411784
H 0 0.967801 3.922454 -2.166016
H 0 -0.365416 5.872869 -1.331585
H 0 1.165275 5.766776 -0.446637
H 0 -0.335223 5.213378 0.311990
H 0 -1.845788 -4.042663 -1.453655
H 0 2.138635 -2.322882 1.930782
H 0 3.045250 -0.870413 2.403577
H 0 3.838729 -2.459345 2.396985
Free Energy (PCM/B3LYP/6-31G*) = -1642.020763
Number of imaginary frequencies = 0

4-3*E*-c11
B3LYP/6-31G* geometry
O 0 -1.226846 -1.918366 0.442928
C 0 -2.508120 -3.583280 -0.739982
C 0 -3.392129 -2.709711 1.387924
C 0 -3.738138 -3.503651 0.099896
C 0 -2.472244 -1.487325 1.093687
C 0 -1.394580 -2.844173 -0.505754
C 0 -0.334113 -3.214624 -1.444010
N 0 -0.911965 -4.226958 -2.233801
C 0 -2.237331 -4.466781 -1.877523
S 0 1.661157 -1.510305 -0.557902
C 0 3.299388 -1.342906 -1.390834
C 0 4.370920 -0.993622 -0.356390
C 0 4.597704 -2.101376 0.671413
O 0 4.021816 0.197481 0.316667
O 0 4.594837 -1.887716 1.868312
O 0 4.836504 -3.292576 0.113639
O 0 -2.993699 -5.264767 -2.435014
O 0 -4.835451 -2.870661 -0.573568
O 0 -2.638259 -3.656894 2.161135
C 0 -3.095308 -0.342508 0.288163
C 0 -2.168111 0.868832 0.117837
C 0 -2.850183 2.034383 -0.610202
C 0 -1.934735 3.249902 -0.805067
C 0 -2.623349 4.414511 -1.524295

C 0 0.928649 -2.752665 -1.566637
C 0 -4.647999 -2.294680 2.158971
H 0 -4.042082 -4.509631 0.422972
H 0 -2.119208 -1.100939 2.055872
H 0 -0.484500 -4.621815 -3.059617
H 0 3.246108 -0.544162 -2.134168
H 0 3.550018 -2.280133 -1.891546
H 0 5.322241 -0.867642 -0.899394
H 0 4.180259 0.026587 1.265224
H 0 4.993485 -3.939521 0.829329
H 0 -4.941093 -3.338898 -1.418297
H 0 -2.412011 -3.234343 3.006285
H 0 -3.421087 -0.718633 -0.687490
H 0 -4.003705 -0.024742 0.813405
H 0 -1.268352 0.571146 -0.434629
H 0 -1.825802 1.207956 1.106819
H 0 -3.208944 1.690266 -1.591697
H 0 -3.745484 2.341159 -0.049122
H 0 -1.044000 2.944284 -1.372093
H 0 -1.570398 3.589070 0.175154
H 0 -1.943172 5.264767 -1.650770
H 0 -3.496571 4.766400 -0.960993
H 0 -2.971212 4.114215 -2.520544
H 0 1.568774 -3.175281 -2.335815
H 0 -5.322241 -1.684345 1.555999
H 0 -4.375592 -1.730676 3.059617
H 0 -5.184744 -3.196313 2.470588
Free Energy (PCM/B3LYP/6-31G*) = -1642.020711
Number of imaginary frequencies = 0

4-3*Z*-c1
B3LYP/6-31G* geometry
O 0 1.811819 -1.427858 0.157214
C 0 2.224287 -3.703792 -0.482361
C 0 4.165336 -2.240042 -0.116632
C 0 3.666844 -3.529919 -0.824479
C 0 3.129703 -1.110921 -0.408674
C 0 1.440002 -2.699543 -0.027354
C 0 0.096277 -3.209275 0.265057
N 0 0.167934 -4.577493 -0.033508
C 0 1.441036 -4.936017 -0.474858
S 0 -2.500439 -3.202264 1.167409
C 0 -3.607710 -1.755213 0.955526
C 0 -3.932408 -1.435510 -0.519420
C 0 -4.830465 -0.200225 -0.577166
O 0 -4.574523 -2.505885 -1.169924
O 0 -5.995660 -0.256109 -0.918725
O 0 -4.205369 0.917057 -0.188363
O 0 1.810927 -6.076800 -0.768844
O 0 4.458559 -4.648327 -0.425403
O 0 5.370055 -1.792751 -0.748171
C 0 3.510626 0.268251 0.113453
C 0 2.590214 1.388966 -0.390667
C 0 3.012794 2.773808 0.115920
C 0 2.108721 3.907648 -0.384374
C 0 2.540525 5.289033 0.118802
C 0 -0.952576 -2.499202 0.727669
C 0 4.399671 -2.460209 1.381749
H 0 3.796887 -3.365966 -1.906629
H 0 2.993092 -1.066819 -1.497524
H 0 -0.586425 -5.239019 0.084427
H 0 -3.157291 -0.892143 1.453407
H 0 -4.526342 -2.021622 1.486347
H 0 -2.999249 -1.206418 -1.048310
H 0 -5.521829 -2.272046 -1.201992
H 0 -4.846706 1.654156 -0.222208
H 0 3.933242 -5.441708 -0.642999
H 0 5.995660 -2.535800 -0.707140
H 0 3.509053 0.251383 1.210198
H 0 4.541106 0.462338 -0.203048
H 0 1.557659 1.184724 -0.079378
H 0 2.588774 1.388864 -1.490508
H 0 3.018061 2.773099 1.216060
H 0 4.049410 2.973126 -0.193969
H 0 1.073828 3.710143 -0.070750
H 0 2.100279 3.904712 -1.483657
H 0 1.876648 6.076800 -0.255691
H 0 3.560026 5.528023 -0.208559
H 0 2.525995 5.333391 1.214946
H 0 -0.819925 -1.435183 0.899961
H 0 3.475869 -2.721599 1.906629
H 0 4.813915 -1.555127 1.833375
H 0 5.109653 -3.278803 1.525635
Free Energy (PCM/B3LYP/6-31G*) = -1642.025760
Number of imaginary frequencies = 0

4-3*Z*-c2
B3LYP/6-31G* geometry
O 0 0.629381 -0.700540 -0.693239
C 0 -0.148731 -2.955573 -0.953650
C 0 1.968114 -2.597609 0.245721
C 0 1.123645 -3.627260 -0.554649
C 0 1.979795 -1.264480 -0.563567
C 0 -0.308225 -1.611785 -0.974006
C 0 -1.690075 -1.279703 -1.334175
N 0 -2.309933 -2.522109 -1.543051
C 0 -1.435706 -3.572678 -1.258417
S 0 -3.862527 0.260068 -2.003870
C 0 -4.219134 1.886324 -1.224518
C 0 -4.825813 1.801799 0.190047
C 0 -3.881322 1.140739 1.188718
O 0 -6.055135 1.115923 0.182804
O 0 -4.158235 0.106272 1.763410
O 0 -2.735548 1.814383 1.345198
O 0 -1.726330 -4.772695 -1.250091
O 0 0.896601 -4.796670 0.231118
O 0 3.335964 -3.020462 0.258684
C 0 2.859853 -0.163957 0.014200
C 0 3.036948 1.035553 -0.927737
C 0 3.946466 2.121328 -0.339575
C 0 4.143176 3.322582 -1.273296
C 0 5.056938 4.401791 -0.683731
C 0 -2.223618 -0.046941 -1.447553
C 0 1.452537 -2.419148 1.677876
H 0 1.718397 -3.899811 -1.441672
H 0 2.310418 -1.503969 -1.583161
H 0 -3.310633 -2.650030 -1.613153
H 0 -4.954401 2.359940 -1.879128
H 0 -3.311791 2.495906 -1.230640
H 0 -4.987988 2.837555 0.523101
H 0 -5.909480 0.295534 0.691235
H 0 -2.177325 1.325749 1.981754
H 0 0.125556 -5.244120 -0.166978
H 0 3.344674 -3.912358 0.645353
H 0 2.435101 0.172345 0.968100
H 0 3.835102 -0.610025 0.237176
H 0 2.055134 1.467292 -1.161353
H 0 3.457296 0.686749 -1.882302
H 0 3.527033 2.469873 0.615979
H 0 4.927201 1.683836 -0.100440
H 0 3.163166 3.760960 -1.509718
H 0 4.559193 2.972854 -2.228899
H 0 5.178020 5.244120 -1.374637
H 0 6.055135 4.000649 -0.468422
H 0 4.649301 4.796400 0.255360
H 0 -1.596371 0.813023 -1.235105
H 0 0.433536 -2.021486 1.699209
H 0 2.105692 -1.737536 2.228899
H 0 1.445529 -3.385885 2.187971
Free Energy (PCM/B3LYP/6-31G*) = -1642.025120
Number of imaginary frequencies = 0

4-3*Z*-c3
B3LYP/6-31G* geometry
O 0 2.179261 -1.383300 -0.285317
C 0 3.203068 -3.522301 -0.653412
C 0 4.640290 -1.671194 0.087888
C 0 4.616546 -3.043106 -0.640612
C 0 3.503386 -0.793400 -0.519861
C 0 2.137442 -2.707576 -0.472031
C 0 0.900294 -3.493183 -0.487524
N 0 1.329311 -4.817512 -0.673314
C 0 2.718430 -4.894937 -0.753788
S 0 -1.781846 -4.044795 -0.300254

C 0 -3.120554 -2.876084 -0.768581
C 0 -3.570895 -1.943964 0.371683
C 0 -4.778132 -1.125177 -0.086917
O 0 -2.548677 -1.061577 0.781531
O 0 -4.726261 0.079275 -0.242481
O 0 -5.861867 -1.875388 -0.311566
O 0 3.381577 -5.932030 -0.862809
O 0 5.496111 -3.962011 0.007070
O 0 5.847246 -0.979667 -0.251990
C 0 3.440353 0.636592 0.001747
C 0 2.472749 1.531045 -0.786334
C 0 2.423282 2.967652 -0.251456
C 0 1.468780 3.876648 -1.036339
C 0 1.417384 5.308766 -0.494184
C 0 -0.354577 -3.020532 -0.350194
C 0 4.524814 -1.825604 1.608109
H 0 4.979420 -2.861472 -1.665509
H 0 3.646664 -0.782572 -1.608766
H 0 0.729574 -5.630337 -0.668392
H 0 -3.949884 -3.519591 -1.074121
H 0 -2.795853 -2.295049 -1.635831
H 0 -3.876638 -2.555188 1.229451
H 0 -2.784573 -0.187285 0.415119
H 0 -6.580469 -1.290766 -0.624087
H 0 5.207388 -4.852096 -0.271629
H 0 6.580469 -1.569831 -0.008560
H 0 3.151445 0.619749 1.059863
H 0 4.453908 1.049094 -0.047954
H 0 1.465471 1.095854 -0.757340
H 0 2.774244 1.545896 -1.843914
H 0 2.120332 2.951808 0.806045
H 0 3.434917 3.399658 -0.272478
H 0 0.459361 3.441610 -1.018367
H 0 1.774010 3.895501 -2.092236
H 0 0.728094 5.932030 -1.075543
H 0 2.406711 5.781668 -0.531059
H 0 1.081708 5.324753 0.550206
H 0 -0.512189 -1.958448 -0.204122
H 0 3.571200 -2.273998 1.902485
H 0 4.616258 -0.849889 2.092236
H 0 5.326258 -2.473016 1.973174
Free Energy (PCM/B3LYP/6-31G*) = -1642.024884
Number of imaginary frequencies = 0

4-3*Z*-c4
B3LYP/6-31G* geometry
O 0 1.972108 -1.399061 -0.226598
C 0 2.328784 -3.710484 -0.764089
C 0 4.288908 -2.347913 -0.175106
C 0 3.811324 -3.626729 -0.917167
C 0 3.367796 -1.171899 -0.622753
C 0 1.552499 -2.652952 -0.428441
C 0 0.157630 -3.080254 -0.296824
N 0 0.178735 -4.453593 -0.598335
C 0 1.483200 -4.897284 -0.821150
S 0 -2.555267 -2.885043 0.073007
C 0 -3.378985 -1.604501 1.106114
C 0 -3.699715 -0.289192 0.382033
C 0 -4.541357 -0.501406 -0.873904
O 0 -2.523503 0.409784 0.038049
O 0 -4.198691 -0.086413 -1.963884
O 0 -5.676629 -1.165103 -0.634840
O 0 1.826613 -6.070261 -1.003050
O 0 4.477122 -4.777868 -0.398808
O 0 5.589949 -1.986205 -0.651489
C 0 3.757348 0.198935 -0.084890
C 0 2.975527 1.351996 -0.730243
C 0 3.394961 2.726082 -0.193057
C 0 2.629383 3.890169 -0.834977
C 0 3.054257 5.260464 -0.296986
C 0 -0.896609 -2.304075 0.023121
C 0 4.313133 -2.538476 1.345376
H 0 4.090227 -3.497773 -1.975821
H 0 3.375124 -1.151304 -1.720915
H 0 -0.582111 -5.089401 -0.399908
H 0 -2.752020 -1.396483 1.975821
H 0 -4.302872 -2.072812 1.454202
H 0 -4.304454 0.314867 1.077383
H 0 -2.579519 0.577594 -0.922860
H 0 -6.161181 -1.258840 -1.478561
H 0 3.921259 -5.543161 -0.641479
H 0 6.161181 -2.759374 -0.506621
H 0 3.611468 0.214544 1.002197
H 0 4.829778 0.327818 -0.266726
H 0 1.900753 1.205969 -0.562005
H 0 3.125630 1.325453 -1.819476
H 0 3.246264 2.752161 0.896721
H 0 4.473707 2.867043 -0.357140
H 0 1.551869 3.749477 -0.668959
H 0 2.776451 3.862394 -1.924052
H 0 2.491357 6.070261 -0.775442
H 0 4.120862 5.443054 -0.477827
H 0 2.885397 5.330572 0.784845
H 0 -0.743599 -1.250452 0.224201
H 0 3.314579 -2.727503 1.750724
H 0 4.722629 -1.647042 1.827626
H 0 4.944832 -3.393962 1.598343
Free Energy (PCM/B3LYP/6-31G*) = -1642.024712
Number of imaginary frequencies = 0

4-3*Z*-c5
B3LYP/6-31G* geometry
O 0 1.193774 -0.469531 -0.181786
C 0 -0.005824 -2.481043 -0.696764
C 0 2.342733 -2.687291 0.001521
C 0 1.182115 -3.382201 -0.763332
C 0 2.419047 -1.212778 -0.501240
C 0 0.072046 -1.162812 -0.406978
C 0 -1.278428 -0.584488 -0.357612
N 0 -2.136717 -1.652535 -0.655280
C 0 -1.417923 -2.837299 -0.800711
S 0 -3.085364 1.582765 -0.048251
C 0 -4.146373 0.847295 -1.372836
C 0 -5.337899 0.020978 -0.862128
C 0 -6.221665 0.804514 0.105853
O 0 -4.933383 -1.176312 -0.226820
O 0 -6.456952 0.409856 1.231098
O 0 -6.688785 1.939299 -0.420170
O 0 -1.903607 -3.963697 -0.956726
O 0 0.917066 -4.668564 -0.204862
O 0 3.587557 -3.268319 -0.402817
C 0 3.581756 -0.397466 0.049822
C 0 3.748031 0.965268 -0.637506
C 0 4.935297 1.766952 -0.089432
C 0 5.111907 3.131194 -0.768309
C 0 6.299941 3.928384 -0.219885
C 0 -1.556457 0.709990 -0.098593
C 0 2.175142 -2.790569 1.521144
H 0 1.522764 -3.506364 -1.804355
H 0 2.480590 -1.245123 -1.597482
H 0 -3.143252 -1.627946 -0.477635
H 0 -3.522729 0.233259 -2.024571
H 0 -4.521036 1.689824 -1.958833
H 0 -5.950286 -0.228370 -1.740549
H 0 -5.252497 -1.122586 0.696478
H 0 -7.240433 2.387206 0.251252
H 0 0.005652 -4.895172 -0.473491
H 0 3.516511 -4.222124 -0.228701
H 0 3.442676 -0.254554 1.128453
H 0 4.490221 -0.995391 -0.080315
H 0 2.826662 1.549820 -0.519029
H 0 3.880717 0.811015 -1.718391
H 0 4.806311 1.915429 0.993037
H 0 5.857787 1.179550 -0.209056
H 0 4.189772 3.717056 -0.646670
H 0 5.238558 2.982744 -1.850147
H 0 6.401194 4.895172 -0.726439
H 0 7.240433 3.380037 -0.355823
H 0 6.183562 4.124523 0.853318
H 0 -0.717264 1.351841 0.151305
H 0 1.273916 -2.277240 1.869509
H 0 3.041864 -2.354439 2.024571
H 0 2.095358 -3.841623 1.810921
Free Energy (PCM/B3LYP/6-31G*) = -1642.024351

Number of imaginary frequencies = 0

4-3*Z*-c6
B3LYP/6-31G* geometry

O 0	1.744873	-1.400019	-0.220197
C 0	2.126132	-3.708363	-0.755106
C 0	4.073051	-2.322161	-0.177735
C 0	3.606648	-3.606822	-0.916863
C 0	3.136637	-1.156390	-0.620637
C 0	1.339436	-2.659162	-0.418507
C 0	-0.049955	-3.102024	-0.279050
N 0	-0.013907	-4.476173	-0.572901
C 0	1.293825	-4.905159	-0.804847
S 0	-2.764413	-2.925539	0.101703
C 0	-3.592899	-1.637373	1.122436
C 0	-3.973603	-0.363576	0.364917
C 0	-4.946430	-0.668678	-0.788186
O 0	-2.830788	0.306065	-0.174325
O 0	-5.948259	-1.335167	-0.641094
O 0	-4.606081	-0.123501	-1.959950
O 0	1.649428	-6.074147	-0.988028
O 0	4.289682	-4.749362	-0.402115
O 0	5.367371	-1.945544	-0.660647
C 0	3.511925	0.217698	-0.080726
C 0	2.715011	1.363439	-0.720635
C 0	3.113188	2.739873	-0.173337
C 0	2.331352	3.897087	-0.808090
C 0	2.732982	5.269443	-0.257659
C 0	-1.110383	-2.334024	0.040159
C 0	4.107170	-2.512298	1.342651
H 0	3.877560	-3.474739	-1.977189
H 0	3.140150	-1.133713	-1.718733
H 0	-0.769672	-5.119624	-0.380316
H 0	-2.957344	-1.397233	1.977189
H 0	-4.503823	-2.114915	1.490654
H 0	-4.511180	0.289932	1.063618
H 0	-2.678163	1.129139	0.315628
H 0	-3.751867	0.342606	-1.807384
H 0	3.742380	-5.521776	-0.641536
H 0	5.948259	-2.711974	-0.518318
H 0	3.368654	0.228642	1.006779
H 0	4.582313	0.359579	-0.264796
H 0	1.642088	1.200710	-0.555728
H 0	2.867613	1.345953	-1.809662
H 0	2.962536	2.756025	0.916388
H 0	4.189919	2.898102	-0.334546
H 0	1.255650	3.738143	-0.646448
H 0	2.481797	3.880498	-1.896900
H 0	2.158718	6.074147	-0.731173
H 0	3.797036	5.470421	-0.433826
H 0	2.560033	5.328066	0.824219
H 0	-0.960851	-1.277469	0.226856
H 0	3.112910	-2.712675	1.753108
H 0	4.509099	-1.616282	1.822733
H 0	4.749799	-3.360552	1.592337

Free Energy (PCM/B3LYP/6-31G*) = -1642.024232
Number of imaginary frequencies = 0

4-3*Z*-c7
B3LYP/6-31G* geometry

O 0	2.113924	-1.286310	-0.243899
C 0	2.152872	-3.665840	-0.553087
C 0	4.176268	-2.585147	0.332672
C 0	3.628910	-3.835803	-0.408919
C 0	3.577074	-1.324562	-0.363075
C 0	1.526768	-2.470223	-0.453503
C 0	0.078702	-2.652580	-0.589892
N 0	-0.085945	-4.035877	-0.757116
C 0	1.138285	-4.701673	-0.721708
S 0	-2.588186	-1.999262	-0.624282
C 0	-3.204915	-0.373392	-1.244403
C 0	-4.607333	-0.108191	-0.695256
C 0	-4.634160	0.072079	0.822069
O 0	-5.471729	-1.166298	-1.050473
O 0	-5.400936	-0.545349	1.535394
O 0	-3.767830	0.993194	1.255408
O 0	1.300406	-5.923959	-0.795600
O 0	3.971795	-5.022606	0.305931
O 0	5.586085	-2.482808	0.104133
C 0	4.092684	0.010168	0.159231
C 0	3.666252	1.208361	-0.700840
C 0	4.195108	2.543161	-0.161244
C 0	3.782309	3.750961	-1.012171
C 0	4.310002	5.081633	-0.465720
C 0	-0.862369	-1.687616	-0.553999
C 0	3.881920	-2.632606	1.835780
H 0	4.120463	-3.855989	-1.395292
H 0	3.795697	-1.409645	-1.436133
H 0	-0.973760	-4.510763	-0.838728
H 0	-3.241362	-0.400464	-2.335610
H 0	-2.515264	0.412811	-0.930060
H 0	-4.955758	0.842101	-1.132537
H 0	-5.975546	-1.384519	-0.242461
H 0	-3.859540	1.067625	2.225688
H 0	3.366740	-5.716445	-0.018636
H 0	5.975546	-3.323333	0.399048
H 0	3.744478	0.151241	1.189791
H 0	5.185584	-0.053328	0.195593
H 0	2.570790	1.246251	-0.759308
H 0	4.027126	1.062158	-1.729381
H 0	3.836380	2.686823	0.868844
H 0	5.292745	2.501562	-0.098850
H 0	2.685543	3.790309	-1.075570
H 0	4.142467	3.608699	-2.040952
H 0	3.998629	5.923959	-1.094253
H 0	5.406277	5.084762	-0.422417
H 0	3.938244	5.267354	0.549683
H 0	-0.540876	-0.659238	-0.422505
H 0	2.807616	-2.620808	2.043070
H 0	4.343296	-1.776986	2.335610
H 0	4.295145	-3.550577	2.261695

Free Energy (PCM/B3LYP/6-31G*) = -1642.024172
Number of imaginary frequencies = 0

4-3*Z*-c8
B3LYP/6-31G* geometry

O 0	2.267619	-1.335270	-0.393308
C 0	2.580106	-3.694674	-0.699459
C 0	4.457355	-2.390500	0.208294
C 0	4.063988	-3.697293	-0.535012
C 0	3.726862	-1.207944	-0.498231
C 0	1.820445	-2.578474	-0.601686
C 0	0.403336	-2.926520	-0.737338
N 0	0.398516	-4.316460	-0.940792
C 0	1.689966	-4.839652	-0.859512
S 0	-2.308725	-2.563386	-0.974586
C 0	-3.200149	-1.244569	-0.043833
C 0	-4.599581	-1.052585	-0.653429
C 0	-5.379689	-0.042859	0.187681
O 0	-5.321977	-2.261993	-0.721001
O 0	-6.327673	-0.362410	0.876835
O 0	-4.884174	1.196744	0.100615
O 0	1.988256	-6.037799	-0.893888
O 0	4.527416	-4.836854	0.188593
O 0	5.849206	-2.129905	-0.004692
C 0	4.083528	0.178368	0.022186
C 0	3.530677	1.317720	-0.845858
C 0	3.917484	2.705245	-0.319013
C 0	3.379858	3.855262	-1.180262
C 0	3.769646	5.238850	-0.649952
C 0	-0.645165	-2.080354	-0.689462
C 0	4.153351	-2.467166	1.708367
H 0	4.567901	-3.664868	-1.514789
H 0	3.963352	-1.271361	-1.569007
H 0	-0.428245	-4.895209	-0.877257
H 0	-2.625432	-0.317703	-0.114469
H 0	-3.276242	-1.541278	1.006162
H 0	-4.495584	-0.658729	-1.670705
H 0	-5.897877	-2.281571	0.066828
H 0	-5.407723	1.777736	0.687278
H 0	3.991360	-5.590964	-0.123528
H 0	6.327673	-2.916716	0.306925
H 0	3.714885	0.282987	1.050088
H 0	5.176332	0.239285	0.065540

H 0 2.437240 1.239376 -0.901507
H 0 3.902876 1.201969 -1.874278
H 0 3.546994 2.820343 0.710525
H 0 5.013537 2.779065 -0.258670
H 0 2.284691 3.780774 -1.239873
H 0 3.749932 3.739898 -2.208933
H 0 3.371224 6.037799 -1.285935
H 0 4.859910 5.355186 -0.611639
H 0 3.384272 5.396851 0.365098
H 0 -0.451625 -1.027274 -0.510244
H 0 3.082932 -2.581375 1.903850
H 0 4.505078 -1.561435 2.208933
H 0 4.667170 -3.328496 2.142865
Free Energy (PCM/B3LYP/6-31G*) = -1642.023823
Number of imaginary frequencies = 0

4-3*Z*-c9
B3LYP/6-31G* geometry
O 0 -1.984530 -1.820143 0.858697
C 0 -2.565481 -3.607905 -0.652832
C 0 -4.123500 -3.039302 1.173571
C 0 -3.965046 -3.809611 -0.170689
C 0 -3.424311 -1.651604 1.140285
C 0 -1.723107 -2.676201 -0.139143
C 0 -0.421306 -2.747750 -0.805727
N 0 -0.555206 -3.797572 -1.724914
C 0 -1.851194 -4.322727 -1.717116
S 0 2.205077 -2.163459 -1.350773
C 0 2.987114 -0.521497 -1.069058
C 0 3.566522 -0.308625 0.336706
C 0 4.581852 -1.382173 0.720594
O 0 2.548779 -0.263293 1.312580
O 0 4.478357 -2.042359 1.736094
O 0 5.580604 -1.493732 -0.160660
O 0 -2.266774 -5.200387 -2.472250
O 0 -4.941066 -3.351326 -1.118524
O 0 -3.498858 -3.822468 2.202989
C 0 -4.005482 -0.603127 0.189850
C 0 -3.303941 0.759121 0.284074
C 0 -3.937816 1.814509 -0.631007
C 0 -3.252038 3.184154 -0.546535
C 0 -3.886602 4.233898 -1.464653
C 0 0.648956 -1.966765 -0.556683
C 0 -5.587022 -2.919464 1.589253
H 0 -4.145622 -4.870385 0.053508
H 0 -3.423032 -1.268999 2.164624
H 0 0.110827 -4.008660 -2.455355
H 0 2.253073 0.258935 -1.280925
H 0 3.783351 -0.453693 -1.814391
H 0 4.106249 0.651746 0.313800
H 0 2.785808 -0.934003 1.982301
H 0 6.189484 -2.191348 0.152317
H 0 -4.742682 -3.803563 -1.955609
H 0 -2.537282 -3.789647 2.068360
H 0 -3.975751 -0.981503 -0.837590
H 0 -5.065950 -0.479287 0.440573
H 0 -2.242956 0.644678 0.028470
H 0 -3.335153 1.112820 1.325200
H 0 -3.908639 1.458337 -1.671555
H 0 -5.002425 1.926296 -0.376895
H 0 -2.188008 3.071830 -0.798921
H 0 -3.282874 3.540179 0.493087
H 0 -3.376437 5.200387 -1.379771
H 0 -4.943278 4.391346 -1.214895
H 0 -3.837968 3.922033 -2.515418
H 0 0.584075 -1.196285 0.202203
H 0 -6.189484 -2.448035 0.812261
H 0 -5.671771 -2.341406 2.515418
H 0 -5.985476 -3.922311 1.773878
Free Energy (PCM/B3LYP/6-31G*) = -1642.023746
Number of imaginary frequencies = 0

4-3*Z*-c10
B3LYP/6-31G* geometry
O 0 0.652724 -0.568253 -0.174220
C 0 0.489219 -2.897844 -0.721581
C 0 2.696735 -2.000966 -0.101695
C 0 1.954297 -3.136743 -0.862008
C 0 2.073979 -0.643022 -0.554912
C 0 -0.033859 -1.696095 -0.384332
C 0 -1.489046 -1.803354 -0.257432
N 0 -1.771819 -3.146810 -0.562386
C 0 -0.597321 -3.867904 -0.785491
S 0 -4.089037 -0.999881 0.120167
C 0 -4.599834 0.461942 1.114207
C 0 -4.584407 1.802755 0.366027
C 0 -5.418560 1.772458 -0.912186
O 0 -3.268384 2.202231 0.050307
O 0 -4.959376 2.078822 -1.995296
O 0 -6.683654 1.396768 -0.701002
O 0 -0.521619 -5.087150 -0.971726
O 0 2.350691 -4.408812 -0.349383
O 0 4.055769 -1.967662 -0.549200
C 0 2.690071 0.642657 -0.000941
C 0 4.007719 1.087125 -0.653764
C 0 4.456297 2.468114 -0.157860
C 0 5.770613 2.944206 -0.789992
C 0 6.213034 4.324224 -0.292534
C 0 -2.342449 -0.810943 0.063520
C 0 2.644724 -2.199726 1.417383
H 0 2.266944 -3.063494 -1.916447
H 0 2.097394 -0.615815 -1.652398
H 0 -2.656507 -3.599057 -0.374794
H 0 -3.958227 0.532150 1.995296
H 0 -5.615127 0.233829 1.447777
H 0 -5.049106 2.543088 1.036934
H 0 -3.258970 2.365060 -0.913087
H 0 -7.153292 1.406451 -1.558214
H 0 1.643067 -5.033421 -0.599417
H 0 4.412096 -2.860841 -0.404089
H 0 1.937964 1.425753 -0.163331
H 0 2.814890 0.559399 1.085126
H 0 3.879384 1.118505 -1.745627
H 0 4.790262 0.348455 -0.455168
H 0 3.666361 3.205433 -0.365207
H 0 4.570077 2.443035 0.936271
H 0 5.658512 2.967824 -1.883354
H 0 6.559989 2.208550 -0.580208
H 0 7.153292 4.636159 -0.761739
H 0 6.366363 4.322593 0.793835
H 0 5.457754 5.087150 -0.518928
H 0 -1.955761 0.181320 0.262583
H 0 1.623776 -2.138765 1.805345
H 0 3.253427 -1.441725 1.917024
H 0 3.041955 -3.186299 1.669479
Free Energy (PCM/B3LYP/6-31G*) = -1642.023580
Number of imaginary frequencies = 0

4-3*Z*-c11
B3LYP/6-31G* geometry
O 0 -1.052124 0.089025 -0.569495
C 0 0.400857 1.940119 -1.037926
C 0 -1.814443 2.429407 -0.094430
C 0 -0.634710 3.008250 -0.922296
C 0 -2.171537 1.032402 -0.690936
C 0 0.139382 0.627011 -0.854023
C 0 1.384278 -0.147563 -0.974044
N 0 2.380390 0.809908 -1.190609
C 0 1.838619 2.090968 -1.253866
S 0 2.921375 -2.512580 -0.940717
C 0 4.207753 -1.637404 0.066118
C 0 5.411114 -1.168588 -0.763424
C 0 6.518387 -0.611907 0.133649
O 0 5.048560 -0.165482 -1.697693
O 0 6.980848 0.500814 -0.027824
O 0 6.919704 -1.476214 1.068407
O 0 2.471796 3.137127 -1.431618
O 0 -0.130022 4.190202 -0.301864
O 0 -2.983312 3.224481 -0.322193
C 0 -3.382967 0.355609 -0.063421
C 0 -3.856297 -0.889856 -0.826924
C 0 -5.076479 -1.551605 -0.175100
C 0 -5.576357 -2.790541 -0.929703
C 0 -6.792116 -3.448325 -0.269008

C 0 1.486794 -1.488088 -0.864222
C 0 -1.492534 2.371881 1.403030
H 0 -1.042885 3.268329 -1.912582
H 0 -2.341583 1.170813 -1.767055
H 0 3.352195 0.584363 -1.415174
H 0 4.546605 -2.360620 0.810515
H 0 3.738310 -0.800143 0.586642
H 0 5.834785 -2.032313 -1.295640
H 0 5.692435 0.561940 -1.574137
H 0 7.635667 -1.058635 1.586944
H 0 0.763314 4.326990 -0.671000
H 0 -2.743539 4.138375 -0.093153
H 0 -3.145380 0.086337 0.973093
H 0 -4.187736 1.097761 -0.025413
H 0 -3.035826 -1.616330 -0.889390
H 0 -4.102268 -0.609141 -1.861464
H 0 -4.827473 -1.833491 0.858804
H 0 -5.892736 -0.817468 -0.102698
H 0 -4.759541 -3.522451 -1.004768
H 0 -5.828551 -2.507980 -1.961725
H 0 -7.126726 -4.326990 -0.832485
H 0 -7.635667 -2.749470 -0.208199
H 0 -6.558969 -3.775312 0.751967
H 0 0.567178 -2.050174 -0.736106
H 0 -0.659849 1.694952 1.616414
H 0 -2.368920 2.033518 1.961725
H 0 -1.215121 3.367750 1.758268
Free Energy (PCM/B3LYP/6-31G*) = -1642.023468
Number of imaginary frequencies = 0

5-3*R*-c1
B3LYP/6-31G* geometry
O 0 -1.413341 0.407573 -0.030300
C 0 -3.623934 -0.332632 -0.542766
C 0 -1.920752 -2.048478 -0.077623
C 0 -3.283821 -1.771789 -0.772285
C 0 -0.935245 -0.912124 -0.486214
C 0 -2.717645 0.592296 -0.190645
C 0 -3.373879 1.945296 0.033659
O 0 -4.781905 1.636238 -0.226719
C 0 -4.928513 0.308011 -0.560285
C 0 -2.914150 3.029611 -0.925994
O 0 -6.018569 -0.178104 -0.802917
O 0 -4.271275 -2.660107 -0.257050
O 0 -1.344214 -3.231947 -0.636591
C 0 -2.067370 -2.192601 1.441656
C 0 0.487375 -1.064191 0.033623
C 0 1.471794 -0.057016 -0.577960
C 0 2.903948 -0.241073 -0.060634
C 0 3.900982 0.757295 -0.662672
C 0 5.331328 0.564658 -0.148320
O 0 -3.226880 2.428765 1.332066
H 0 -3.140480 -1.967314 -1.846357
H 0 -0.926652 -0.861870 -1.582496
H 0 -1.850781 3.230716 -0.771002
H 0 -3.483658 3.942843 -0.733917
H 0 -3.073189 2.714087 -1.959733
H 0 -5.133650 -2.327390 -0.564590
H 0 -1.996098 -3.942843 -0.514052
H 0 -2.787870 -2.982610 1.667862
H 0 -2.422685 -1.270581 1.911847
H 0 -1.105110 -2.460590 1.884900
H 0 0.484160 -0.964187 1.125783
H 0 0.808217 -2.086527 -0.194136
H 0 1.463641 -0.161857 -1.672614
H 0 1.133246 0.964453 -0.361515
H 0 2.910762 -0.143582 1.035149
H 0 3.240332 -1.265717 -0.278037
H 0 3.566326 1.780606 -0.440992
H 0 3.890133 0.663198 -1.757867
H 0 5.380261 0.686120 0.940991
H 0 6.018569 1.291721 -0.596160
H 0 5.705351 -0.438856 -0.386529
H 0 -3.480764 1.729604 1.959733
Free Energy (PCM/B3LYP/6-31G*) = -997.782624
Number of imaginary frequencies = 0

5-3*R*-c2
B3LYP/6-31G* geometry
O 0 -1.410565 0.410419 -0.010353
C 0 -3.621299 -0.331946 -0.518274
C 0 -1.920053 -2.044109 -0.042733
C 0 -3.280435 -1.771539 -0.743850
C 0 -0.933077 -0.911969 -0.458894
C 0 -2.714871 0.592208 -0.167178
C 0 -3.373706 1.939182 0.061684
O 0 -4.781779 1.634788 -0.208850
C 0 -4.928097 0.303340 -0.521564
C 0 -2.927789 3.034122 -0.896860
O 0 -6.019222 -0.191041 -0.745472
O 0 -4.270950 -2.656981 -0.229739
O 0 -1.342970 -3.233660 -0.588156
C 0 -2.072514 -2.170616 1.477539
C 0 0.489175 -1.062090 0.062438
C 0 1.473441 -0.054617 -0.548894
C 0 2.905492 -0.238572 -0.031193
C 0 3.902060 0.762194 -0.630013
C 0 5.332358 0.568980 -0.115710
O 0 -3.208981 2.277964 1.402893
H 0 -3.133211 -1.971179 -1.816766
H 0 -0.923844 -0.868124 -1.555477
H 0 -1.868936 3.253735 -0.735138
H 0 -3.513638 3.942206 -0.719236
H 0 -3.075444 2.723075 -1.934211
H 0 -5.133949 -2.306437 -0.515948
H 0 -1.996665 -3.942206 -0.461840
H 0 -2.796380 -2.955817 1.709757
H 0 -2.426873 -1.241485 1.934211
H 0 -1.112766 -2.436608 1.927499
H 0 0.484949 -0.961153 1.154482
H 0 0.811020 -2.084375 -0.164156
H 0 1.465613 -0.159025 -1.643610
H 0 1.134628 0.966685 -0.332114
H 0 2.911531 -0.143745 1.064826
H 0 3.242924 -1.262387 -0.250862
H 0 3.566654 1.784618 -0.405352
H 0 3.891657 0.671389 -1.725495
H 0 5.380693 0.686728 0.974038
H 0 6.019222 1.298135 -0.560730
H 0 5.707304 -0.433407 -0.357182
H 0 -3.512534 3.193613 1.528778
Free Energy (PCM/B3LYP/6-31G*) = -997.782218
Number of imaginary frequencies = 0

5-3*R*-c3
B3LYP/6-31G* geometry
O 0 1.083878 -0.542907 -0.088959
C 0 3.277626 0.390962 -0.208161
C 0 1.388998 1.939907 0.095790
C 0 2.856145 1.814095 -0.401120
C 0 0.569737 0.769662 -0.527333
C 0 2.408377 -0.620355 -0.057307
C 0 3.133370 -1.935586 0.181618
O 0 4.533703 -1.508201 0.157147
C 0 4.618429 -0.151141 -0.065756
C 0 2.907168 -2.978818 -0.899301
O 0 5.688969 0.427959 -0.107825
O 0 3.680445 2.735242 0.307068
O 0 0.806459 3.118365 -0.467591
C 0 1.299035 1.978849 1.625833
C 0 -0.920225 0.781986 -0.214870
C 0 -1.723364 -0.229441 -1.047669
C 0 -3.240109 -0.170283 -0.801698
C 0 -3.684791 -0.614940 0.599275
C 0 -5.208036 -0.606869 0.767650
O 0 2.838801 -2.520814 1.411597
H 0 2.853970 2.072739 -1.471450
H 0 0.722993 0.800474 -1.613610
H 0 1.852157 -3.265048 -0.918138
H 0 3.514029 -3.860914 -0.678232
H 0 3.188957 -2.580747 -1.876768
H 0 4.603136 2.490915 0.112548
H 0 1.376950 3.860914 -0.205753
H 0 1.919022 2.792164 2.011079

H 0 1.645129 1.047140 2.083755
H 0 0.264767 2.152833 1.932860
H 0 -1.058697 0.593444 0.855136
H 0 -1.283837 1.796543 -0.412887
H 0 -1.529466 -0.039662 -2.112514
H 0 -1.360364 -1.245202 -0.843073
H 0 -3.598712 0.852754 -0.989278
H 0 -3.737445 -0.808206 -1.545837
H 0 -3.235948 0.036249 1.360952
H 0 -3.299120 -1.625821 0.794531
H 0 -5.618009 0.397678 0.604604
H 0 -5.501534 -0.927509 1.773860
H 0 -5.688969 -1.281035 0.047955
H 0 2.952884 -1.855293 2.112514
Free Energy (PCM/B3LYP/6-31G*) = -997.782109
Number of imaginary frequencies = 0

5-3*R*-c4
B3LYP/6-31G* geometry
O 0 1.874163 -0.505798 -1.306912
C 0 3.497691 -0.199114 0.429325
C 0 2.511965 1.803823 -0.612303
C 0 3.307679 1.271840 0.617562
C 0 1.361226 0.851925 -1.031187
C 0 2.814012 -0.921496 -0.475743
C 0 3.246739 -2.377254 -0.459631
O 0 4.262348 -2.374957 0.585308
C 0 4.419662 -1.101985 1.105396
C 0 3.858393 -2.867012 -1.761245
O 0 5.224445 -0.876381 1.986715
O 0 2.605904 1.593637 1.823174
O 0 3.393922 1.772922 -1.743842
C 0 1.997489 3.227088 -0.384151
C 0 0.178475 0.736605 -0.067369
C 0 -0.918292 -0.216887 -0.564068
C 0 -2.138370 -0.246511 0.364995
C 0 -3.238818 -1.204982 -0.108502
C 0 -4.457545 -1.229372 0.819685
O 0 2.210473 -3.249938 -0.117627
H 0 4.283930 1.780593 0.619676
H 0 1.016548 1.181098 -2.014596
H 0 3.110853 -2.827205 -2.558059
H 0 4.191707 -3.900535 -1.634078
H 0 4.711418 -2.243034 -2.037930
H 0 3.115651 1.214060 2.558059
H 0 4.100321 2.421396 -1.586647
H 0 2.849599 3.900535 -0.233367
H 0 1.360141 3.298580 0.498534
H 0 1.441574 3.572131 -1.262029
H 0 0.537279 0.426719 0.920225
H 0 -0.246091 1.739732 0.058334
H 0 -1.235338 0.085677 -1.572889
H 0 -0.506830 -1.229465 -0.659973
H 0 -1.818543 -0.534431 1.377498
H 0 -2.554723 0.767925 0.453620
H 0 -2.822783 -2.218871 -0.193888
H 0 -3.555639 -0.918978 -1.121542
H 0 -4.177677 -1.545304 1.832381
H 0 -5.224445 -1.922094 0.454288
H 0 -4.915441 -0.235446 0.897763
H 0 1.796101 -2.933918 0.704280
Free Energy (PCM/B3LYP/6-31G*) = -997.780978
Number of imaginary frequencies = 0

5-3*R*-c5
B3LYP/6-31G* geometry
O 0 1.896211 -0.456216 -1.333625
C 0 3.510296 -0.188548 0.420216
C 0 2.550324 1.836171 -0.606667
C 0 3.337980 1.285986 0.620979
C 0 1.381846 0.901406 -1.027370
C 0 2.814834 -0.899591 -0.485290
C 0 3.213842 -2.365300 -0.464612
O 0 4.224316 -2.382151 0.585718
C 0 4.406329 -1.114824 1.107071
C 0 3.819572 -2.874360 -1.761474
O 0 5.206106 -0.904941 1.995310
O 0 2.635425 1.593490 1.829562
O 0 3.465324 1.930945 -1.708082
C 0 2.059179 3.261448 -0.367662
C 0 0.206791 0.767291 -0.057597
C 0 -0.896295 -0.174014 -0.563989
C 0 -2.112961 -0.212536 0.369268
C 0 -3.218953 -1.159300 -0.114822
C 0 -4.435041 -1.192149 0.816558
O 0 2.155683 -3.210660 -0.124499
H 0 4.314354 1.789208 0.619432
H 0 1.029534 1.245531 -2.002898
H 0 3.077518 -2.817941 -2.562366
H 0 4.124848 -3.915761 -1.629021
H 0 4.690691 -2.274452 -2.035156
H 0 3.156970 1.225893 2.562366
H 0 3.741801 1.035200 -1.962406
H 0 2.926598 3.915761 -0.235036
H 0 1.436798 3.328937 0.524947
H 0 1.491748 3.616248 -1.234210
H 0 0.571435 0.439466 0.921950
H 0 -0.212789 1.769906 0.089210
H 0 -1.216042 0.144923 -1.566840
H 0 -0.489601 -1.186879 -0.676568
H 0 -1.790740 -0.516900 1.376160
H 0 -2.524661 0.802191 0.474389
H 0 -2.807538 -2.173588 -0.216346
H 0 -3.537515 -0.857056 -1.122581
H 0 -4.153674 -1.524539 1.823550
H 0 -5.206106 -1.875853 0.443033
H 0 -4.888210 -0.197470 0.910889
H 0 1.752559 -2.890368 0.701408
Free Energy (PCM/B3LYP/6-31G*) = -997.780935
Number of imaginary frequencies = 0

5-3*R*-c6
B3LYP/6-31G* geometry
O 0 1.883216 -0.496674 -1.308189
C 0 3.502886 -0.196219 0.433392
C 0 2.525545 1.810670 -0.603108
C 0 3.317986 1.276009 0.622023
C 0 1.368774 0.856926 -1.022724
C 0 2.821543 -0.915514 -0.475590
C 0 3.251011 -2.372163 -0.460607
O 0 4.263058 -2.373871 0.587478
C 0 4.420897 -1.102195 1.111078
C 0 3.865426 -2.861925 -1.760910
O 0 5.222800 -0.880428 1.995811
O 0 2.610820 1.598815 1.823099
O 0 3.508529 1.801617 -1.649064
C 0 2.016539 3.235326 -0.371454
C 0 0.185401 0.734385 -0.058710
C 0 -0.912539 -0.216099 -0.558595
C 0 -2.132859 -0.247277 0.370158
C 0 -3.234986 -1.201968 -0.107050
C 0 -4.454693 -1.226011 0.819868
O 0 2.211403 -3.242881 -0.123084
H 0 4.291680 1.784798 0.615040
H 0 1.007958 1.185648 -2.002731
H 0 3.120440 -2.819399 -2.559994
H 0 4.195623 -3.896475 -1.633923
H 0 4.720962 -2.239891 -2.034175
H 0 3.122905 1.226901 2.559994
H 0 3.096247 2.170568 -2.447757
H 0 2.876098 3.896475 -0.223531
H 0 1.372945 3.305175 0.506843
H 0 1.456991 3.590567 -1.245281
H 0 0.546517 0.419156 0.926314
H 0 -0.238266 1.737130 0.073849
H 0 -1.229472 0.090276 -1.566381
H 0 -0.502110 -1.228795 -0.658073
H 0 -1.813770 -0.539371 1.381680
H 0 -2.547404 0.767589 0.462321
H 0 -2.821204 -2.216571 -0.194827
H 0 -3.550173 -0.912428 -1.119607
H 0 -4.176546 -1.545133 1.832032
H 0 -5.222800 -1.916050 0.451942
H 0 -4.910407 -0.231243 0.900014

H 0 1.796577 -2.927852 0.698959
Free Energy (PCM/B3LYP/6-31G*) = -997.780848
Number of imaginary frequencies = 0

5-3*R*-c7
B3LYP/6-31G* geometry
O 0 1.895521 -0.401078 -1.330476
C 0 3.514619 -0.118699 0.416046
C 0 2.543339 1.897642 -0.615852
C 0 3.335249 1.355445 0.612144
C 0 1.377790 0.956271 -1.030645
C 0 2.818378 -0.835475 -0.483273
C 0 3.228719 -2.294840 -0.458505
O 0 4.255609 -2.301845 0.577942
C 0 4.414781 -1.037153 1.107728
C 0 3.842348 -2.809105 -1.753218
O 0 5.206128 -0.818368 2.002083
O 0 2.632622 1.663388 1.820567
O 0 3.455765 1.992143 -1.719626
C 0 2.046761 3.321826 -0.381564
C 0 0.203690 0.823149 -0.059477
C 0 -0.897763 -0.121463 -0.562927
C 0 -2.115963 -0.157751 0.368364
C 0 -3.219257 -1.108393 -0.114389
C 0 -4.437307 -1.140583 0.814403
O 0 2.121440 -3.035337 -0.042896
H 0 4.309040 1.863921 0.608349
H 0 1.023608 1.294757 -2.007518
H 0 3.096786 -2.776402 -2.552363
H 0 4.176506 -3.843313 -1.617904
H 0 4.704421 -2.201718 -2.040831
H 0 3.147320 1.284241 2.552363
H 0 3.747068 1.097438 -1.960777
H 0 2.911701 3.980129 -0.252548
H 0 1.425233 3.389971 0.511599
H 0 1.476585 3.671296 -1.248511
H 0 0.569704 0.498056 0.920320
H 0 -0.217354 1.825604 0.084624
H 0 -1.216618 0.192531 -1.567732
H 0 -0.489004 -1.133851 -0.669133
H 0 -1.795013 -0.457885 1.376887
H 0 -2.529739 0.856653 0.468815
H 0 -2.805502 -2.122172 -0.211351
H 0 -3.536363 -0.810502 -1.123951
H 0 -4.157401 -1.468872 1.823133
H 0 -5.206128 -1.827246 0.441600
H 0 -4.892838 -0.146563 0.904285
H 0 2.326360 -3.980129 -0.149814
Free Energy (PCM/B3LYP/6-31G*) = -997.780693
Number of imaginary frequencies = 0

5-3*R*-c8
B3LYP/6-31G* geometry
O 0 1.872632 -0.432895 -1.296863
C 0 3.501400 -0.090796 0.427563
C 0 2.487697 1.890862 -0.626217
C 0 3.291276 1.378218 0.606130
C 0 1.346638 0.922542 -1.034398
C 0 2.822343 -0.828152 -0.467798
C 0 3.286843 -2.271129 -0.450278
O 0 4.319029 -2.245704 0.579629
C 0 4.440177 -0.973852 1.106402
C 0 3.912451 -2.752796 -1.752022
O 0 5.236703 -0.729188 1.990846
O 0 2.585566 1.699407 1.809780
O 0 3.367471 1.860578 -1.759732
C 0 1.958908 3.310693 -0.409316
C 0 0.165002 0.805390 -0.069620
C 0 -0.930527 -0.150080 -0.564932
C 0 -2.151352 -0.178752 0.363193
C 0 -3.250694 -1.138738 -0.109847
C 0 -4.470782 -1.162025 0.816582
O 0 2.212337 -3.059562 -0.034103
H 0 4.260548 1.900380 0.604606
H 0 0.998594 1.238538 -2.021020
H 0 3.160126 -2.748683 -2.545497
H 0 4.292360 -3.772073 -1.623788
H 0 4.744342 -2.106442 -2.043420
H 0 3.090220 1.314682 2.545497
H 0 4.067448 2.517422 -1.608630
H 0 2.804780 3.994058 -0.268114
H 0 1.324357 3.383846 0.475235
H 0 1.396151 3.642086 -1.288085
H 0 0.525659 0.496712 0.917543
H 0 -0.261671 1.807688 0.055671
H 0 -1.247326 0.150387 -1.574544
H 0 -0.518280 -1.162317 -0.657609
H 0 -1.832117 -0.464982 1.376310
H 0 -2.568548 0.835558 0.449798
H 0 -2.833899 -2.152529 -0.192531
H 0 -3.566293 -0.854994 -1.123948
H 0 -4.192113 -1.475593 1.830331
H 0 -5.236703 -1.856060 0.451564
H 0 -4.929488 -0.168245 0.891883
H 0 2.458389 -3.994058 -0.144214
Free Energy (PCM/B3LYP/6-31G*) = -997.780553
Number of imaginary frequencies = 0

5-3*R*-c9
B3LYP/6-31G* geometry
O 0 1.880693 -0.426505 -1.296906
C 0 3.506844 -0.087861 0.431415
C 0 2.498827 1.896009 -0.617923
C 0 3.300054 1.382323 0.609825
C 0 1.352289 0.924520 -1.025315
C 0 2.830189 -0.823296 -0.467066
C 0 3.293432 -2.266596 -0.450660
O 0 4.323143 -2.243479 0.581615
C 0 4.443501 -0.972643 1.111293
C 0 3.921608 -2.747546 -1.751432
O 0 5.237609 -0.730405 1.998431
O 0 2.588382 1.703904 1.808808
O 0 3.478689 1.888815 -1.667133
C 0 1.974244 3.316955 -0.398362
C 0 0.170563 0.799751 -0.059776
C 0 -0.926318 -0.152423 -0.558476
C 0 -2.147602 -0.182819 0.369034
C 0 -3.248875 -1.138165 -0.108901
C 0 -4.470289 -1.161506 0.815773
O 0 2.217167 -3.054597 -0.037942
H 0 4.266051 1.905727 0.599128
H 0 0.987488 1.239255 -2.008498
H 0 3.171113 -2.742161 -2.546657
H 0 4.300298 -3.767299 -1.623341
H 0 4.754797 -2.101720 -2.040263
H 0 3.095223 1.326732 2.546657
H 0 3.060032 2.248215 -2.466876
H 0 2.826875 3.989190 -0.260535
H 0 1.333579 3.388184 0.481960
H 0 1.407071 3.657414 -1.273157
H 0 0.533986 0.485201 0.924483
H 0 -0.255116 1.801632 0.073032
H 0 -1.242857 0.152162 -1.567025
H 0 -0.515258 -1.164829 -0.654969
H 0 -1.829535 -0.474170 1.381039
H 0 -2.562594 0.832031 0.459863
H 0 -2.834709 -2.152763 -0.194795
H 0 -3.562367 -0.849822 -1.122370
H 0 -4.193881 -1.479466 1.828765
H 0 -5.237609 -1.852109 0.447198
H 0 -4.926379 -0.166747 0.894015
H 0 2.462910 -3.989190 -0.147912
Free Energy (PCM/B3LYP/6-31G*) = -997.780411
Number of imaginary frequencies = 0

5-3*R*-c10
B3LYP/6-31G* geometry
O 0 2.182089 -0.941974 -0.012958
C 0 3.886489 0.660587 -0.482453
C 0 1.637434 1.497961 0.083700
C 0 2.976554 1.836657 -0.633370
C 0 1.182561 0.083217 -0.391490
C 0 3.443974 -0.569975 -0.183733
C 0 4.599633 -1.547794 -0.039861

O 0 5.752453 -0.678472 -0.284758
C 0 5.337562 0.610582 -0.540712
C 0 4.595466 -2.674500 -1.059337
O 0 6.129390 1.509801 -0.758081
O 0 3.516665 3.032446 -0.078109
O 0 0.634523 2.398947 -0.391575
C 0 1.762529 1.600167 1.608648
C 0 -0.144784 -0.458495 0.138736
C 0 -1.406940 0.149450 -0.491239
C 0 -2.683247 -0.567955 -0.032050
C 0 -3.961597 0.021549 -0.641661
C 0 -5.235117 -0.696261 -0.182847
O 0 4.695844 -2.117616 1.228146
H 0 2.735123 2.003551 -1.694644
H 0 1.166508 0.087868 -1.488473
H 0 3.707284 -3.295704 -0.916095
H 0 5.489032 -3.288234 -0.917636
H 0 4.591108 -2.269919 -2.074156
H 0 4.427053 3.111505 -0.415024
H 0 0.977071 3.295704 -0.234701
H 0 2.129424 2.593306 1.879328
H 0 2.458686 0.862257 2.018790
H 0 0.784969 1.452283 2.074156
H 0 -0.131389 -1.536012 -0.070569
H 0 -0.179225 -0.358825 1.229893
H 0 -1.469514 1.213572 -0.244826
H 0 -1.330907 0.088779 -1.586612
H 0 -2.617126 -1.635433 -0.290248
H 0 -2.752368 -0.523204 1.065136
H 0 -3.892188 -0.020927 -1.737971
H 0 -4.027801 1.087452 -0.381400
H 0 -5.212268 -1.757883 -0.458875
H 0 -6.129390 -0.253043 -0.636055
H 0 -5.350415 -0.640153 0.906792
H 0 4.667429 -1.409969 1.895584
Free Energy (PCM/B3LYP/6-31G*) = -997.780297
Number of imaginary frequencies = 0

5-3*R*-c11
B3LYP/6-31G* geometry
O 0 1.397583 -0.262215 -0.033131
C 0 3.631832 0.450269 -0.544922
C 0 1.916728 2.175046 -0.106605
C 0 3.292551 1.889980 -0.780139
C 0 0.925772 1.047048 -0.517649
C 0 2.702096 -0.456244 -0.192541
C 0 3.313591 -1.828000 0.024940
O 0 4.711199 -1.579730 -0.282289
C 0 4.911600 -0.255240 -0.641968
C 0 2.773770 -2.907966 -0.898138
O 0 6.010123 0.133433 -0.972834
O 0 4.321415 2.732236 -0.281126
O 0 1.488330 3.400332 -0.713633
C 0 2.040102 2.330972 1.410527
C 0 -0.506566 1.208176 -0.027716
C 0 -1.475097 0.168555 -0.610457
C 0 -2.916942 0.366235 -0.126432
C 0 -3.896383 -0.667537 -0.696740
C 0 -5.335873 -0.463963 -0.213145
O 0 3.182395 -2.285672 1.338824
H 0 3.158818 2.078302 -1.856941
H 0 0.934776 0.981636 -1.613606
H 0 1.709753 -3.063665 -0.701444
H 0 3.313354 -3.840396 -0.711606
H 0 2.907089 -2.616253 -1.942554
H 0 4.040061 3.640829 -0.484821
H 0 0.865165 3.840396 -0.113438
H 0 2.802079 3.077453 1.643952
H 0 2.322646 1.391228 1.893570
H 0 1.087671 2.655075 1.843639
H 0 -0.525683 1.159328 1.067814
H 0 -0.839707 2.213746 -0.312473
H 0 -1.447119 0.223652 -1.708252
H 0 -1.133154 -0.838809 -0.340920
H 0 -2.941769 0.320529 0.972449
H 0 -3.257075 1.376678 -0.397869
H 0 -3.556890 -1.676446 -0.422679
H 0 -3.869228 -0.624126 -1.794789
H 0 -5.400286 -0.535644 0.879775
H 0 -6.010123 -1.217059 -0.637002
H 0 -5.714681 0.524091 -0.502877
H 0 3.503892 -1.593311 1.942554
Free Energy (PCM/B3LYP/6-31G*) = -997.780252
Number of imaginary frequencies = 0

5-3*S*-c1
B3LYP/6-31G* geometry
O 0 1.412259 -0.657214 -0.198677
C 0 3.623585 0.100165 -0.681326
C 0 1.918711 1.800481 -0.175519
C 0 3.281570 1.543400 -0.878310
C 0 0.932580 0.675722 -0.613637
C 0 2.717526 -0.835168 -0.357306
C 0 3.375189 -2.192642 -0.166991
O 0 4.782969 -1.875777 -0.419462
C 0 4.929934 -0.535612 -0.701581
C 0 3.237239 -2.766761 1.232943
O 0 6.022578 -0.035821 -0.901909
O 0 4.269125 2.419810 -0.343721
O 0 1.341462 2.998688 -0.701488
C 0 2.067057 1.901962 1.347017
C 0 -0.487671 0.811954 -0.082940
C 0 -1.474857 -0.179567 -0.715223
C 0 -2.904111 -0.008213 -0.185424
C 0 -3.904934 -0.992111 -0.804681
C 0 -5.332260 -0.810300 -0.278020
O 0 2.934435 -3.156787 -1.071634
H 0 3.136469 1.764115 -1.947305
H 0 0.919021 0.657445 -1.710791
H 0 2.183052 -2.959586 1.449290
H 0 3.633828 -2.066723 1.971963
H 0 3.792244 -3.706904 1.290158
H 0 5.133530 2.079048 -0.637235
H 0 1.991633 3.706904 -0.556425
H 0 2.793827 2.679771 1.594270
H 0 2.415268 0.964306 1.791013
H 0 1.107077 2.165260 1.797951
H 0 -0.478449 0.683122 1.006148
H 0 -0.810616 1.839649 -0.281871
H 0 -1.472650 -0.046881 -1.806877
H 0 -1.135305 -1.206166 -0.526545
H 0 -2.904338 -0.131543 0.907772
H 0 -3.241673 1.021261 -0.376542
H 0 -3.569770 -2.020384 -0.608250
H 0 -3.900249 -0.872999 -1.897479
H 0 -5.375132 -0.956192 0.808543
H 0 -6.022578 -1.526667 -0.738192
H 0 -5.706806 0.198564 -0.491492
H 0 2.977377 -2.789422 -1.971963
Free Energy (PCM/B3LYP/6-31G*) = -997.783130
Number of imaginary frequencies = 0

5-3*S*-c2
B3LYP/6-31G* geometry
O 0 1.412699 -0.539537 -0.209900
C 0 3.623103 0.218019 -0.695124
C 0 1.918536 1.917786 -0.186131
C 0 3.280387 1.661115 -0.890519
C 0 0.932781 0.792919 -0.624626
C 0 2.717075 -0.716533 -0.372421
C 0 3.375564 -2.070430 -0.187441
O 0 4.786274 -1.753774 -0.429298
C 0 4.929066 -0.418376 -0.727290
C 0 3.252665 -2.654631 1.212838
O 0 6.019262 0.081993 -0.941556
O 0 4.268766 2.537860 -0.357190
O 0 1.340528 3.116546 -0.710377
C 0 2.068086 2.018420 1.336347
C 0 -0.487949 0.928561 -0.095117
C 0 -1.473308 -0.064792 -0.727398
C 0 -2.903867 0.106250 -0.201111
C 0 -3.902115 -0.880138 -0.820600
C 0 -5.331019 -0.698763 -0.298108
O 0 2.898498 -2.921026 -1.182399

H 0 3.134080 1.882410 -1.959202
H 0 0.920099 0.774793 -1.721764
H 0 2.202597 -2.867358 1.431336
H 0 3.640807 -1.956878 1.959202
H 0 3.827123 -3.585150 1.274446
H 0 5.132000 2.198863 -0.656209
H 0 1.993965 3.823116 -0.572089
H 0 2.794322 2.796810 1.583351
H 0 2.417631 1.080783 1.779297
H 0 1.108182 2.280462 1.788214
H 0 -0.479700 0.801142 0.994177
H 0 -0.811699 1.955689 -0.295676
H 0 -1.468848 0.065764 -1.819287
H 0 -1.133256 -1.090763 -0.536272
H 0 -2.906560 -0.014658 0.892376
H 0 -3.242134 1.134932 -0.395265
H 0 -3.566298 -1.907599 -0.620999
H 0 -3.894697 -0.763571 -1.913653
H 0 -5.376605 -0.842089 0.788698
H 0 -6.019262 -1.417058 -0.758390
H 0 -5.706208 0.309137 -0.514989
H 0 3.206601 -3.823116 -0.988929
Free Energy (PCM/B3LYP/6-31G*) = -997.782655
Number of imaginary frequencies = 0

5-3*S*-c3
B3LYP/6-31G* geometry
O 0 1.083389 -0.709659 -0.200383
C 0 3.279305 0.221492 -0.294870
C 0 1.398787 1.765198 0.065232
C 0 2.862264 1.650955 -0.446302
C 0 0.571459 0.618954 -0.590835
C 0 2.407441 -0.791244 -0.171278
C 0 3.128472 -2.113461 0.034256
O 0 4.530970 -1.690311 0.015025
C 0 4.617846 -0.326327 -0.156148
C 0 2.831430 -2.790648 1.361594
O 0 5.687933 0.255746 -0.155746
O 0 3.695874 2.546805 0.282740
O 0 0.815072 2.962881 -0.454620
C 0 1.322027 1.753440 1.596283
C 0 -0.916541 0.624602 -0.269035
C 0 -1.726663 -0.354421 -1.133273
C 0 -3.241742 -0.302901 -0.875624
C 0 -3.678088 -0.801062 0.509779
C 0 -5.200087 -0.796334 0.689284
O 0 2.895847 -3.038377 -0.981630
H 0 2.852929 1.941185 -1.508517
H 0 0.717670 0.685931 -1.676443
H 0 1.773655 -3.063268 1.406814
H 0 3.065933 -2.120072 2.191585
H 0 3.438615 -3.695959 1.444779
H 0 4.616204 2.282780 0.101609
H 0 1.385656 3.695959 -0.167623
H 0 1.940947 2.557705 2.001833
H 0 1.679493 0.809737 2.019610
H 0 0.289794 1.910687 1.918871
H 0 -1.049276 0.398065 0.794352
H 0 -1.278221 1.646592 -0.428303
H 0 -1.539353 -0.125547 -2.191585
H 0 -1.363586 -1.377345 -0.968715
H 0 -3.599959 0.727113 -1.021304
H 0 -3.744696 -0.910924 -1.640711
H 0 -3.222080 -0.181540 1.293316
H 0 -3.293829 -1.819853 0.662441
H 0 -5.608755 0.215057 0.571888
H 0 -5.487072 -1.158608 1.683169
H 0 -5.687933 -1.438483 -0.054636
H 0 3.034235 -2.604053 -1.841490
Free Energy (PCM/B3LYP/6-31G*) = -997.781846
Number of imaginary frequencies = 0

5-3*S*-c4
B3LYP/6-31G* geometry
O 0 1.883754 -0.260338 -1.596562
C 0 3.527348 0.040458 0.124731
C 0 2.503666 2.047241 -0.871951
C 0 3.316614 1.507315 0.342203
C 0 1.350004 1.090354 -1.284937
C 0 2.833921 -0.683788 -0.772490
C 0 3.292174 -2.134050 -0.784253
O 0 4.330162 -2.124649 0.239379
C 0 4.468127 -0.861638 0.782986
C 0 2.210917 -3.128734 -0.395330
O 0 5.264872 -0.637941 1.670718
O 0 2.613442 1.781529 1.558260
O 0 3.402736 2.169516 -1.983837
C 0 1.986884 3.461202 -0.618428
C 0 0.181880 0.936336 -0.310240
C 0 -0.947779 0.046429 -0.848836
C 0 -2.144961 -0.032316 0.106849
C 0 -3.277249 -0.927827 -0.412511
C 0 -4.474163 -1.000979 0.541059
O 0 3.825950 -2.542333 -2.006890
H 0 4.278544 2.037599 0.339267
H 0 0.987131 1.430392 -2.258140
H 0 1.408005 -3.108570 -1.137005
H 0 1.800636 -2.881226 0.586451
H 0 2.644057 -4.132077 -0.363779
H 0 3.143587 1.410117 2.282910
H 0 3.699115 1.282349 -2.244848
H 0 2.843815 4.132077 -0.501349
H 0 1.382052 3.513633 0.287085
H 0 1.394981 3.807562 -1.471908
H 0 0.550593 0.556126 0.648822
H 0 -0.216096 1.938498 -0.111319
H 0 -1.283243 0.434716 -1.821696
H 0 -0.564094 -0.964339 -1.035670
H 0 -1.806957 -0.406296 1.084689
H 0 -2.536167 0.980178 0.286012
H 0 -2.885867 -1.940032 -0.587582
H 0 -3.611276 -0.556526 -1.391817
H 0 -4.178070 -1.402648 1.518157
H 0 -5.264872 -1.646181 0.141038
H 0 -4.907462 -0.007190 0.709303
H 0 4.507855 -1.905378 -2.282910
Free Energy (PCM/B3LYP/6-31G*) = -997.781566
Number of imaginary frequencies = 0

5-3*S*-c5
B3LYP/6-31G* geometry
O 0 1.856347 -0.291778 -1.551218
C 0 3.504225 0.062646 0.153879
C 0 2.457197 2.036284 -0.876884
C 0 3.270022 1.525896 0.349418
C 0 1.321690 1.060552 -1.282963
C 0 2.827770 -0.678460 -0.741997
C 0 3.340372 -2.109336 -0.769895
O 0 4.383592 -2.072189 0.247203
C 0 4.484823 -0.804670 0.792751
C 0 2.306086 -3.155384 -0.389080
O 0 5.289840 -0.557564 1.668136
O 0 2.557968 1.822710 1.555159
O 0 3.331729 2.015763 -2.014747
C 0 1.920038 3.451861 -0.652976
C 0 0.142324 0.932842 -0.317217
C 0 -0.975487 0.019878 -0.841505
C 0 -2.181260 -0.039884 0.104687
C 0 -3.302640 -0.958019 -0.398583
C 0 -4.507222 -1.012587 0.546567
O 0 3.883397 -2.475243 -2.003120
H 0 4.230724 2.063428 0.352766
H 0 0.969803 1.377324 -2.268053
H 0 1.494689 -3.156322 -1.121763
H 0 1.896605 -2.942406 0.601207
H 0 2.780287 -4.140575 -0.379367
H 0 3.070173 1.443851 2.288614
H 0 4.027096 2.677995 -1.865810
H 0 2.762791 4.140575 -0.519253
H 0 1.294336 3.519475 0.238261
H 0 1.346181 3.781212 -1.525287
H 0 0.502158 0.582455 0.656422
H 0 -0.264811 1.937366 -0.152717
H 0 -1.303985 0.377692 -1.828360

H 0 -0.582620 -0.992868 -0.995333
H 0 -1.849918 -0.383085 1.096005
H 0 -2.581212 0.974501 0.251014
H 0 -2.902705 -1.971969 -0.541235
H 0 -3.630717 -0.617217 -1.390889
H 0 -4.216664 -1.383820 1.537267
H 0 -5.289840 -1.674855 0.158705
H 0 -4.949068 -0.017615 0.682504
H 0 4.507523 -1.785245 -2.288614
Free Energy (PCM/B3LYP/6-31G*) = -997.781261
Number of imaginary frequencies = 0

5-3*S*-c6
B3LYP/6-31G* geometry
O 0 1.864969 -0.286126 -1.335835
C 0 3.509705 0.067101 0.373410
C 0 2.467703 2.041307 -0.654858
C 0 3.277675 1.531521 0.567860
C 0 1.327075 1.061721 -1.059486
C 0 2.836702 -0.672843 -0.525784
C 0 3.350702 -2.103039 -0.555404
O 0 4.390376 -2.067392 0.465071
C 0 4.489019 -0.800873 1.013801
C 0 2.316151 -3.151105 -0.180955
O 0 5.290946 -0.555544 1.892357
O 0 2.558502 1.829263 1.768088
O 0 3.442714 2.042833 -1.708660
C 0 1.934792 3.458392 -0.430221
C 0 0.147407 0.926459 -0.093318
C 0 -0.971325 0.016177 -0.620368
C 0 -2.177927 -0.044639 0.324763
C 0 -3.300819 -0.958647 -0.182664
C 0 -4.507287 -1.012192 0.760153
O 0 3.898395 -2.465219 -1.787884
H 0 4.234795 2.070885 0.562462
H 0 0.958603 1.376144 -2.041452
H 0 1.507265 -3.150927 -0.916413
H 0 1.902981 -2.941040 0.808430
H 0 2.791235 -4.135877 -0.172090
H 0 3.071459 1.457480 2.504345
H 0 3.019345 2.405728 -2.504345
H 0 2.784456 4.135877 -0.299903
H 0 1.302242 3.525060 0.456258
H 0 1.357315 3.795985 -1.299322
H 0 0.509900 0.571131 0.877498
H 0 -0.259131 1.930225 0.077922
H 0 -1.299266 0.377072 -1.606378
H 0 -0.579448 -0.996578 -0.776926
H 0 -1.848123 -0.391822 1.315182
H 0 -2.575893 0.970087 0.474113
H 0 -2.903341 -1.973306 -0.327071
H 0 -3.626214 -0.614544 -1.174727
H 0 -4.219551 -1.386406 1.750547
H 0 -5.290946 -1.671489 0.369344
H 0 -4.946724 -0.016345 0.897493
H 0 4.525239 -1.775256 -2.067501
Free Energy (PCM/B3LYP/6-31G*) = -997.781227
Number of imaginary frequencies = 0
